# Late-Stage Molecular Editing Enabled by Ketone Chain-Walking Isomerization

**DOI:** 10.1021/jacs.3c05680

**Published:** 2023-08-28

**Authors:** Yannick Brägger, Ori Green, Benjamin N. Bhawal, Bill Morandi

**Affiliations:** † ETH Zürich, Vladimir-Prelog-Weg 3, HCI, 8093 Zürich, Switzerland; ‡ School of Chemistry, University of Edinburgh, Edinburgh EH9 3FJ, U.K.

## Abstract

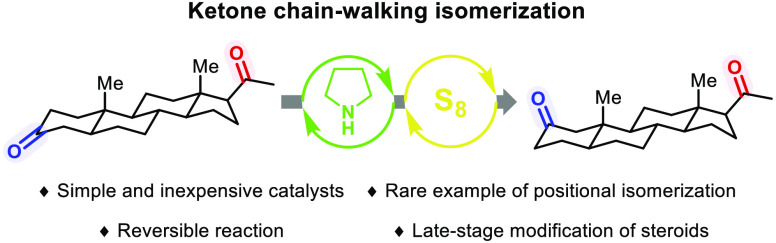

Herein, a method for the isomerization of ketones in a manner akin to the chain-walking reaction of alkenes is described. Widely available and inexpensive pyrrolidine and elemental sulfur are deployed as catalysts to achieve this reversible transformation. Key to the utility of this approach was the elucidation of a stereochemical model to determine the thermodynamically favored product of the reaction and the kinetic selectivity observed. With the distinct selectivity profile of our ketone chain-walking process, the isomerization of various steroids was demonstrated to rapidly access novel steroids with “unnatural” oxidation patterns.

The relative arrangement of functional groups within a molecule imparts its physical and biological properties.^1^ Thus, controlling the position and orientation of functional groups is a fundamental goal in organic synthesis. Typically, this is realized through the deployment of transformations that selectively introduce functional groups with the desired arrangement.^2^ Alternatively, isomerization reactions offer the opportunity to “correct” either the position or the orientation of functional groups within a molecule. This approach is particularly attractive to facilitate the editing of complex molecules that feature a high density of functionality (Scheme 1A).^3,4^ Aside from the atom economy of this approach,^5^ it is also a step-economical strategy,^6^ enabling access to complex chemical entities through a single synthetic procedure rather than a lengthy *de novo* synthesis. Applying this to inexpensive and readily available biomass feedstocks, such as sugars and steroids, also represents a sustainable approach to access analogues of these privileged scaffolds. In 2020, the Wendlandt group provided a compelling demonstration of the utility of this approach (Scheme 1B).^7^ Using a photocatalytic reaction manifold, they accessed rare monosaccharides of biological importance from more readily available monosaccharides through selective epimerization.

**Scheme 1:**
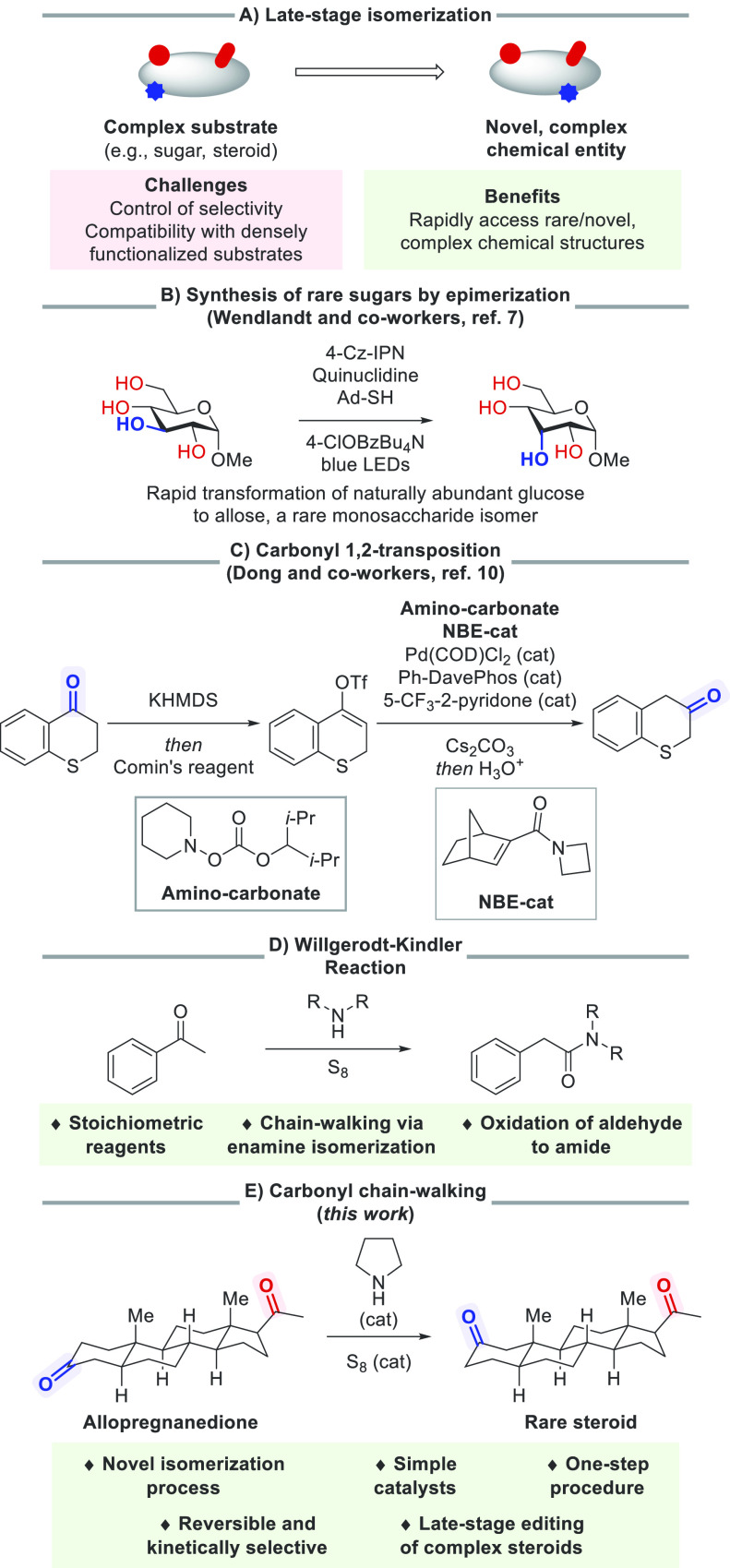
Context of This Work

While previous examples clearly show the feasibility and synthetic utility of late-stage epimerization reactions, amending the location of functional groups is arguably even more challenging, as it requires reversible cleavage and transposition of strong bonds. Alkene chain-walking is a rare example and proceeds via a series of β-hydride eliminations and hydride insertions.^8^ This process has greatly impacted organic synthesis by enabling the synthesis of unusual building blocks and unlocking cascade reactions. Achieving a directly analogous process with ketones, another ubiquitous and versatile functional group, would be highly desirable. However, transition-metal-mediated elementary steps to manipulate the C=O bond of ketones for a chain-walking process are lacking.^9^ To circumvent this challenge, the Dong group designed a Catellani-type process to realize a carbonyl 1,2-transposition (Scheme 1C).^10^ This elegant process is kinetically controlled but is irreversible and limited to 1,2-transpositions. Thus, a reversible carbonyl chain-walking process akin to alkene chain-walking would open new synthetic opportunities.^11^

Given our group’s interest in reversible catalytic reactions^12,13^ and molecular editing processes,^14^ we sought to develop such a process. Cognizant of the challenges of achieving this through transition-metal catalysis, we looked for a mechanistically distinct approach to perform the desired transformation. We took inspiration from the Willgerodt–Kindler reaction (Scheme 1D),^15^ wherein aliphatic ketones are transformed to either an amide or a thioamide, typically by refluxing the ketone with elemental sulfur^16^ in a solution of the amine, with the carbonyl group migrating down the aliphatic chain to the terminal position. Notably, the carbonyl group is able to “walk” along a variety of chain lengths, suggesting that a chain-walking process is operative. We hypothesized that, for cyclic ketones, wherein the carbonyl group is unable to migrate to a terminal position and undergo subsequent oxidation, a carbonyl chain-walking process could be realized using substoichiometric amounts of an amine and elemental sulfur. Previously, the isomerization of cyclic ketones has been demonstrated using stoichiometric quantities of both amine and sulfur.^17^ Herein, we report a simple procedure for the isomerization of cyclic ketones and demonstrate its application in the late-stage editing of complex steroids to rapidly access unnatural isomers of this biologically prevalent class of natural products (Scheme 1E).

At the outset of this work, we were conscious that, as the reaction would be reversible, a suitable driving force to favor product formation would be necessary. Thus, we targeted synthetically relevant ring systems in which certain isomers would be favored thermodynamically. We were intrigued by geminally disubstituted cyclohexanones, as one of the substituents must occupy an axial position. Consequently, 3,3-geminally disubstituted cyclohexanones are more thermodynamically stable than their 2,2- and 4,4-disubstituted counterparts due to a reduction in the number of 1,3-diaxial interactions (Scheme 2A). Furthermore, this structural pattern is frequently embedded in naturally occurring steroids, offering the prospect to apply our method in the late-stage isomerization of bioactive compounds. To evaluate this hypothesis, DFT calculations were performed to determine the relative ground-state energies of 4,4-, 3,3-, and 2,2-dimethylcyclohexanone (Scheme 2B). This showed that, as expected, the 3,3-dimethylcyclohexanone isomer should be favored.

**Scheme 2:**
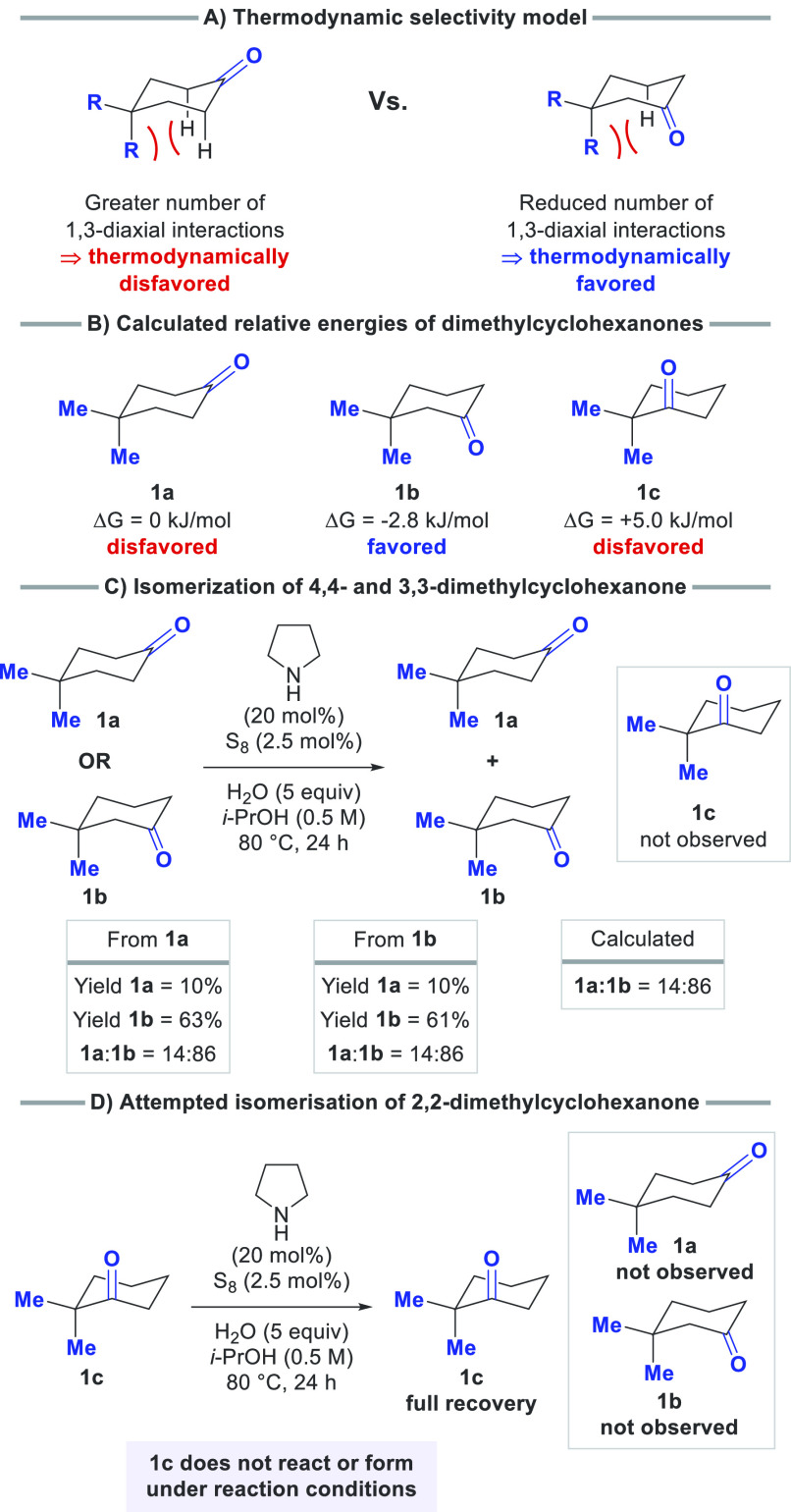
Model for Predicting the Thermodynamic Selectivity of the Reversible Isomerization of Geminally Substituted Dimethylcyclohexanones and Theoretical and Experimental Verification Ground-state energy calculations were performed at the B3LYP/def2-QZVPP level of theory. All yields were determined by GC-FID with an internal standard.

Subsequently, we assessed reaction conditions to facilitate the isomerization of dimethylcyclohexanones. Critically, the isomerization can be performed with substoichiometric amounts of pyrrolidine and sulfur, in contrast to the Willgerodt–Kindler reaction and previous work.^17^ We also observed pyrrolidine to be markedly superior in relation to other amines (see the Supporting Information for further information). Under the optimized conditions, reactions with 4,4- and 3,3-dimethylcyclohexanone (**1a** and **1b**, respectively) yielded a nearly identical mixture of isomers, indicative that an equilibrium is achieved, with **1b** being the major product, thereby verifying its greater thermodynamic stability (Scheme 2C). Notably, formation of 2,2-dimethylcyclohexanone **1c** was not observed, and no isomerization of 2,2-dimethylcyclohexanone occurred when it was submitted to the optimized reaction conditions (Scheme 2D). Together, these results imply that the ketone chain-walking process is reversible, thermodynamically selective for the 3,3-disubstituted isomer, and not applicable to the formation or reaction of sterically encumbered ketones such as 2,2-dimethylcyclohexanone.

The selectivity of the reaction was further probed through the isomerization of a range of mono-substituted cyclohexanones (Scheme 3). With 2-substituted cyclohexanones **3c** and **4c**, limited reactivity was observed, and with 2-*tert*-butylcyclohexanone **2c**, no isomerization was observed at all. Conversely, isomerization of 4-substituted cyclohexanones **2a**, **3a**, and **4a** or 3-substituted cyclohexanones **2b**, **3b**, and **4b** gave limited or no formation of the 2-substituted isomers. With 4-*tert*-butylcyclohexanone **2a** and 3-*tert*-butylcyclohexanone **2b**, no formation of 2-*tert*-butylcyclohexanone **2c** was observed, and the reaction exhibited selectivity for the 3-substituted isomer. The origin of this selectivity remains unclear. It should also be noted that the isomerization of 4-methylcyclohexanone **3a** yielded a mixture of all three isomers, showcasing the potential for longer range walking of the carbonyl group.

**Scheme 3:**
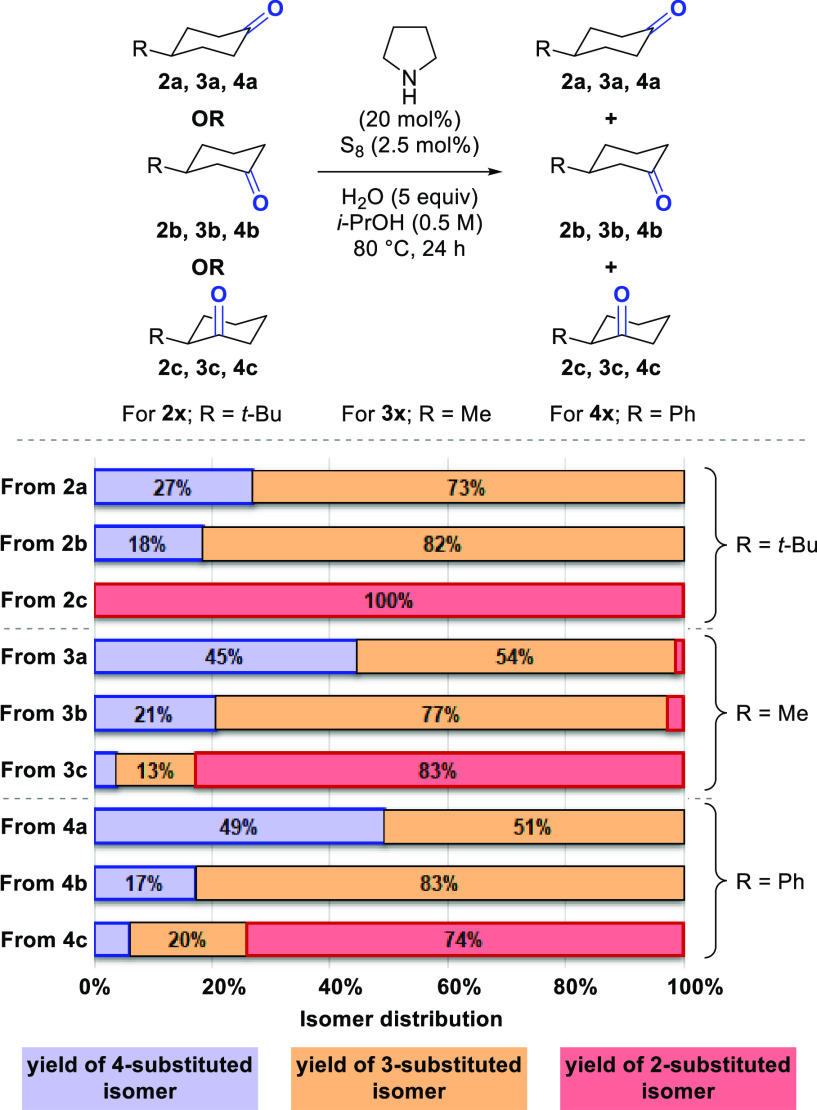
Isomerization of Mono-substituted Cyclohexanones Isomer distributions were determined by GC-FID with an internal standard.

Collectively, these results provide further insights into the kinetic selectivity of our isomerization process (Scheme 4). Ketones bearing bulky α-substituents (e.g., *tert*-butyl) or α,α-disubstituted ketones neither form nor react under these conditions. However, with less bulky substituents (e.g., methyl), the α-substituted isomer will participate in the reaction. However, this reversible process is considerably depressed in comparison to the interconversion of ketone isomers bearing no substituents at the α-position. This selectivity profile is reminiscent of that observed in enamine catalysis, which is typically ineffective with bulkier cycloketones.^18^

**Scheme 4:**
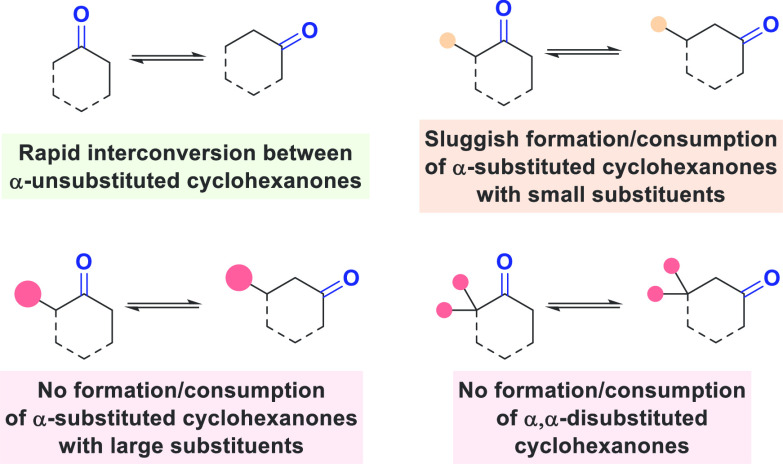
Kinetic Selectivity of Ketone Isomerization

With a greater understanding of the isomerization process, we sought to apply this methodology to more complex targets, as we anticipated our mild reaction conditions to be compatible with a range of substrates. Various derivatives of the Wieland–Miescher ketone, which has been extensively used as a key intermediate in natural product synthesis,^19,20^ were investigated (Scheme 5A). Isomerization with keto-alcohol **5a** proceeded smoothly, affording the 2-oxo-isomer **5b** as the major product, with no other isomers detected. Notably, reaction with diketone **6a** led to the formation of only one new isomer, **6b**. This result underlines the critical role of the selectivity of this transformation; otherwise, a mixture of 16 different regioisomers could be formed. Due to the selectivity profile, the desired product could be obtained in 44% yield. Similarly, reaction with the cis-fused diketone **7a** also afforded a single isomeric product, **7b**, albeit with reduced selectivity. Further derivatives were subjected to the reaction conditions to evaluate the functional group tolerance of the procedure, and substrates incorporating esters (**8a**), silyl ethers (**9a**), alkenes (**10a**), carbamates (**11a**), and imides (**12a**) were all tolerated. While the separation of isomers can potentially present a challenge, we found this could be readily accomplished using preparative HPLC. Combined yields of the reactant and product isomers in these reactions were typically around 80%. However, attempts to identify any byproducts formed were thwarted by the complex mixtures obtained. Derivatives of the Hajos–Parrish ketones, **13a** and **14a**, were also subjected to the reaction conditions (Scheme 5B). With the 6,5-fused ring system, two new isomeric products were formed, with the 4-oxo-isomers **13c** and **14c** being formed as minor products, in addition to the 2-oxo-isomers **13b** and **14b**. Previous syntheses of these ketone isomers have relied on multistep *de novo* synthesis.^21^ For instance, the groups of Wijnberg and de Groot undertook a 7-step synthesis to realize formal isomerization of the Wieland–Miescher ketone derivative **5a**.^22^ This synthetic sequence uses several stoichiometric reagents and utilizes protecting group logic to circumvent selectivity issues. With our method, the same isomerization takes just one single step and avoids the use of protecting groups and stoichiometric reagents, thus constituting a powerful atom- and step-economic upgrade.

**Scheme 5:**
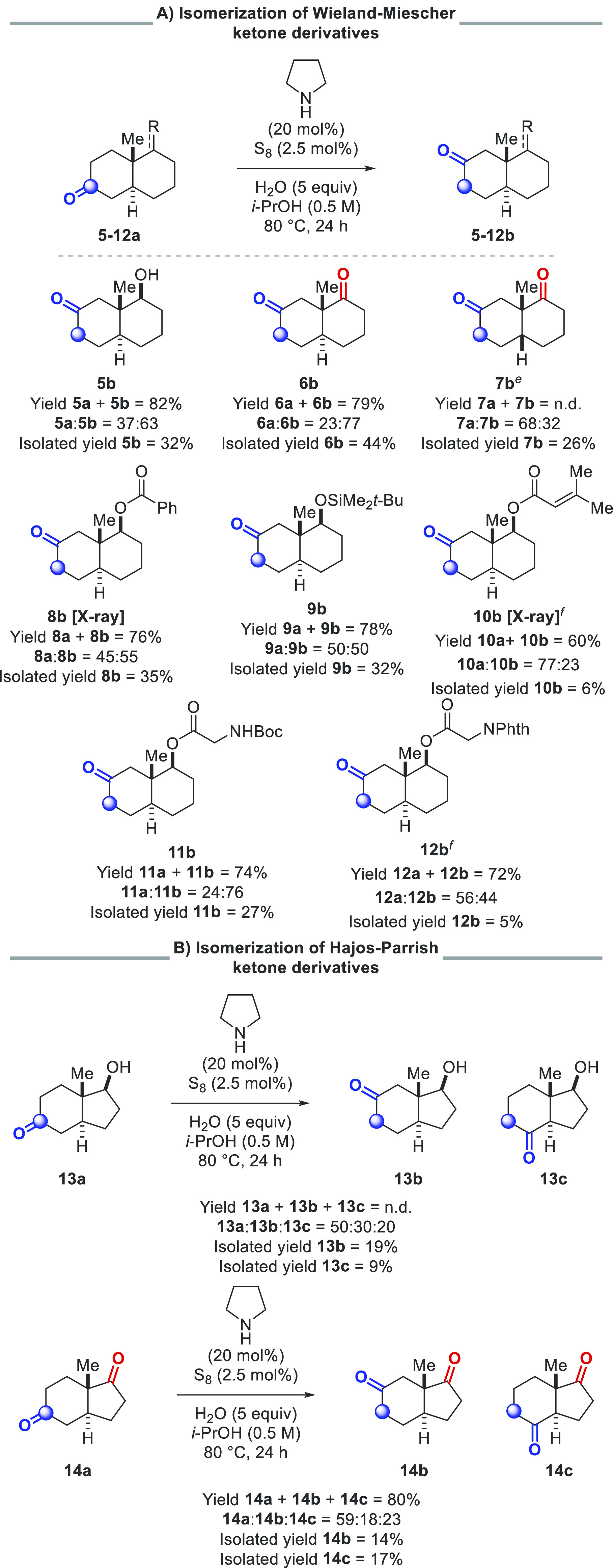
Isomerization of Bicyclic Ketones by Carbonyl Chain-Walking^,^^,^^,^ Reactions were performed on 1.0 mmol scale. Unless otherwise stated, products were initially isolated as a mixture of isomers by flash column chromatography. Combined yields of these isomeric mixtures are reported. Isomer ratios were determined by ^1^H NMR spectroscopy. Unless otherwise stated, isolated yields following purification by preparative HPLC are reported. Isomer **7b** was directly isolated by flash column chromatography. Only a small sample was purified by HPLC.

We next performed the isomerization of naturally occurring steroids (Scheme 6A).^23^ As expected, 3-oxo-steroids smoothly underwent isomerization to afford 2-keto-steroids as the major product. Due to the kinetic selectivity of the reaction, neither the 1- nor 4-keto-steroid isomers were detected in any of the crude reaction mixtures. Thus, the isomerization of androstanolone **15a** and mestanolone **16a** afforded exclusively their respective 2-oxo-isomers, **15b** and **16b**. In the isomerization of the diketosteroids, androstanedione **17a** and allopregnanedione **18a**, only isomerization of the A-ring ketone was observed, with the other ketone remaining untouched. This selectivity is particularly imperative in the case of **18a**, because if the other ketone were able to undergo reaction, this would likely lead to the undesired occurrence of a Willgerodt–Kindler rearrangement, forming an amide species at the terminal position and ultimately inhibiting the desired isomerization process. Furthermore, the synthesis of **18b** has previously been reported in an 8-step sequence starting from pregnenolone (Scheme 6B).^24,25^ The isomerization is achieved through several synthetic steps, and further steps are necessitated by the protecting group strategy and redox manipulations employed to navigate the challenge of achieving selective isomerization. Thus, this truly highlights the synthetic virtues of our new ketone isomerization, especially given its distinct selectivity profile. It should also be noted that the isomerization of diketones **17a** and **18a** would be challenging with the protocol from Dong’s group, as this would require selective triflate formation.^10^

**Scheme 6:**
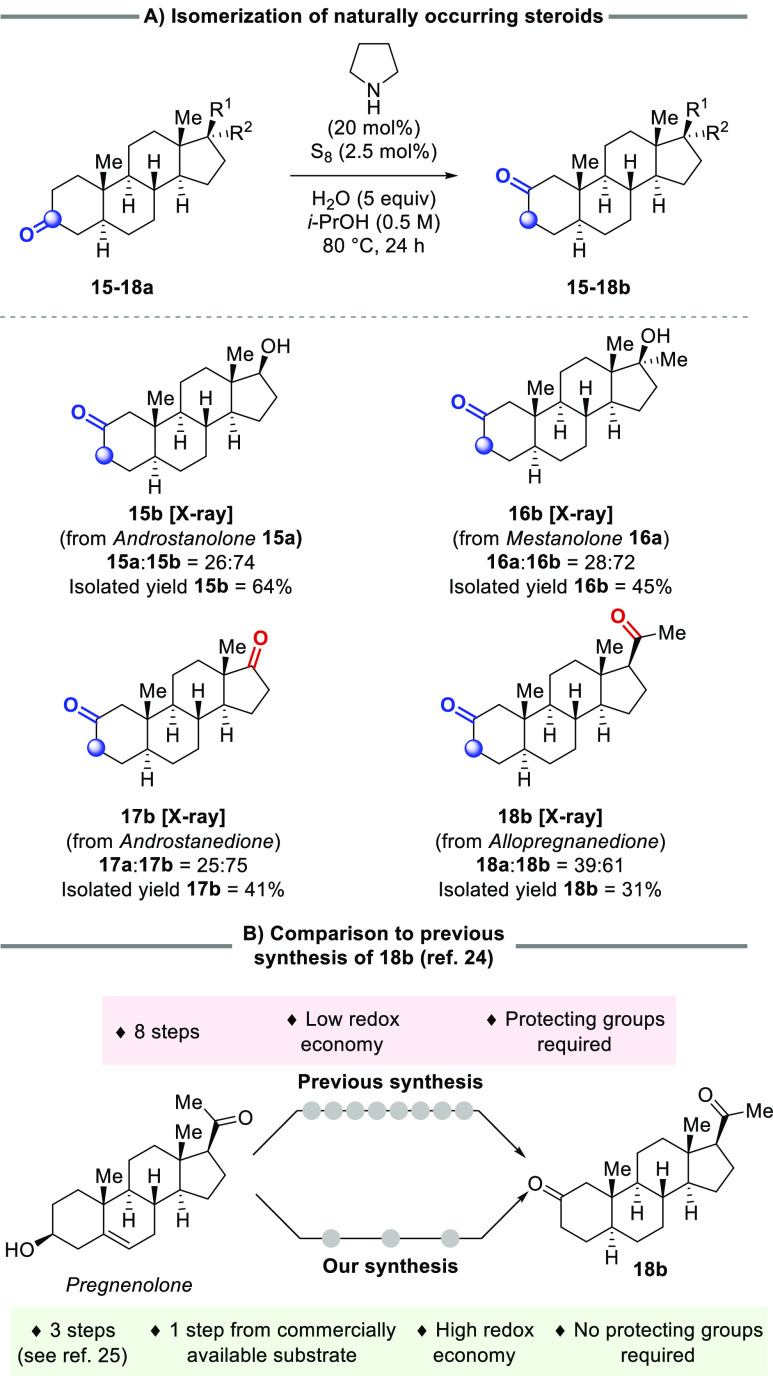
Isomerization of 3-Oxo-Steroids by Carbonyl Chain-Walking^,^^,^ Reactions were performed on 1.0 mmol scale. Isolated yields following purification by preparative HPLC are reported. Isomer ratios were determined by quantitative ^13^C NMR spectroscopy.

Having established a protocol to achieve the chain-walking isomerization of ketones, preliminary mechanistic experiments were performed. Based on studies on the Willgerodt–Kindler reaction^17b,26^ and related transformations,^16,27^ we surmised that the reaction likely proceeds through the formation of an enamine intermediate. To test this hypothesis, 4,4-dimethylcyclohexanone **1a** was submitted to the standard reaction conditions but using D_2_O as additive and CD_3_OD as solvent (Scheme 7). This led to deuterium incorporation detected at all methylene carbons in both ***d*****-1a** and ***d*****-1b**. Since this includes both of the methylene carbons adjacent to the quaternary carbon, this further affirms the reversibility of the system. Subsequently, this reaction was repeated but without the addition of S_8_. No isomerization was observed, and deuterium incorporation was only detected alpha to the ketone, but not at the beta methylene carbons. This result reflects the fact that S_8_ is necessary to isomerize the putative enamine intermediate.

**Scheme 7:**
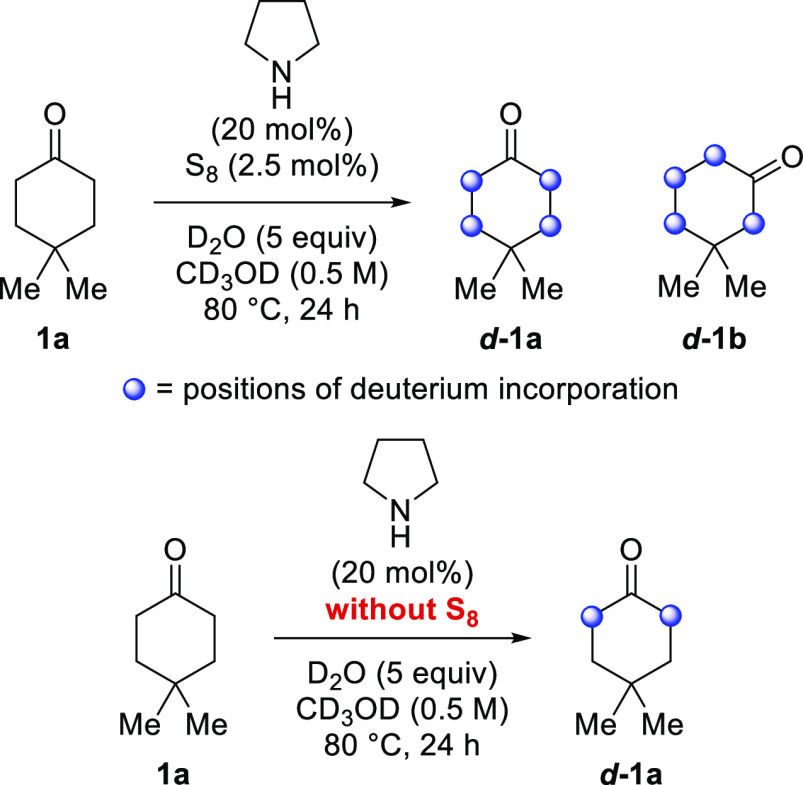
Deuterium Labeling Positions of deuterium incorporation were determined by ^1^H and ^2^H NMR spectroscopy.

In conclusion, we report a novel and simple process for the isomerization of cyclic ketones. Based on well-established concepts in the stereochemistry of cyclohexanes, a model to control this reversible process was devised. Moreover, we found that the process was not amenable to the formation of sterically hindered ketones. Thus, the selective isomerization of 3-oxo-steroids to their 2-oxo-steroid analogues was executed. This process is a rare example of a polar group chain-walking reaction that parallels the venerable alkene chain-walking reaction, which has been a key platform in homogeneous catalysis. We thus expect that our new carbonyl chain-walking will open a wealth of new opportunities for organic synthesis. In a wider context, this work provides compelling motivation for the discovery of new isomerization processes and underlines their value for the late-stage editing of natural products and other complex molecular architectures.

## Supporting Information Available

Experimental procedures and characterization data for all compounds (PDF)
