# Supplementary material for: Late-Stage Molecular Editing Enabled by Ketone Chain-Walking Isomerization
Source: J Am Chem Soc. 2023 Aug 28;145(36):19496–502. doi: 10.1021/jacs.3c05680 (PMC10510328; doi:10.1021/jacs.3c05680)
Supplement: Supplementary file 1 — ja3c05680_si_001.pdf [file ja3c05680_si_001.pdf]

Late-stage molecular editing enabled by ketone chain-walking isomerization

Yannick Brägger[a], Ori Green[a], Benjamin N. Bhawal[a,b]*, Bill Morandi[a]*

[a]*ETH Zürich, Vladimir-Prelog-Weg 3, HCI, 8093 Zürich, Switzerland*

[b]*School of Chemistry, University of Edinburgh, Edinburgh EH9 3FJ, U.K.*

Supporting information

Table of Contents

1. GENERAL INFORMATION 4

2. CURRENT MECHANISTIC HYPOTHESIS 6

3. REACTION OPTIMIZATION........ 7

General procedure for investigating amine catalysts 7
General procedure for investigating temperature/sulfur loading effect 9
General procedure for investigating solvent effect........10
General procedure for investigating the influence of water content and reaction concentration 11

4. ISOMERISATION OF DIMETHYL-SUBSTITUTED AND MONO-SUBSTITUTED CYCLOHEXANONES 12

*Isomerization of 4,4-dimethylcyclohexanone **1a** 12*
*Isomerization of 3,3-dimethylcyclohexanone **1b** 12*
*Isomerization of 2,2-dimethylcyclohexanone **1c**........13*
*Isomerization of 4-tert-butylcyclohexanone **2a** 13*
*Isomerization of 3-tert-butylcyclohexanone **2b** 14*
*Isomerization of 2-tert-butylcyclohexanone **2c**........14*
*Isomerization of 4-methylcyclohexanone **3a** 15*
*Isomerization of 3-methylcyclohexanone **3b** 15*
*Isomerization of 2-methylcyclohexanone **3c**........16*
*Isomerization of 4-phenylcyclohexanone **4a**........16*
*Isomerization of 3-phenylcyclohexanone **4b**........17*
*Isomerization of 2-phenylcyclohexanone **4c** 17*

5. SYNTHESIS AND CHARACTERIZATION OF SUBSTRATES........ 18

*(±)-3-tert-butylcyclohexanone **2b** 18*
*(±)-3-phenylcyclohexanone **4b**........19*
*(±)-(4aS,5S)-5-hydroxy-4a-methyl-4,4a,5,6,7,8-hexahydronaphthalen-2(3H)-one **S4**........20*
*(±)-(4aS,5S)-5-hydroxy-4a-methyloctahydronaphthalen-2(1H)-one **5a**........21*
*(±)-(4aS,8aS)-8a-methylhexahydronaphthalene-1,6(2H,5H)-dione **6a**........22*
*(±)-(4aR,8aS)-8a-methylhexahydronaphthalene-1,6(2H,5H)-dione **7a**........23*
*(±)-(1S,4aS,8aS)-8a-methyl-6-oxodecahydronaphthalen-1-yl benzoate **8a**........24*
*(±)-(4aS,5S,8aS)-5-((tert-butyldimethylsilyl)oxy)-4a-methyloctahydronaphthalen-2(1H)-one **9a**........25*
*(±)-(1S,4aS,8aS)-8a-methyl-6-oxodecahydronaphthalen-1-yl 3-methylbut-2-enoate **10a** 26*

(±)-(1S,4aS,8aS)-8a-methyl-6-oxodecahydronaphthalen-1-yl (tert-butoxycarbonyl)glycinate **11a**....................27
(±)-(1S,4aS,8aS)-8a-methyl-6-oxodecahydronaphthalen-1-yl 2-(1,3-dioxoisoindolin-2-yl)acetate **12a** 28
(±)-(1S,7aS)-1-hydroxy-7a-methyl-1,2,3,6,7,7a-hexahydro-5H-inden-5-one **S7** 29
(±)-(1S,3aS,7aS)-1-hydroxy-7a-methyloctahydro-5H-inden-5-one **13a** 31
(±)-(3aS,7aS)-7a-methylhexahydro-1H-indene-1,5(4H)-dione **14a** 32
Androstanedione **17a**..33

6. ISOMERIZATION OF CYCLIC KETONES AND CHARACTERIZATION OF PRODUCTS... 34

General procedure for the isomerisation of cycloalkanones 34
(±)-(4aS,5S,8aS)-5-hydroxy-4a-methyloctahydronaphthalen-2(1H)-one **5b** 35
(±)-(4aS,8aS)-8a-methylhexahydronaphthalene-1,6(2H,5H)-dione **6b** 36
(±)-(4aR,8aS)-8a-methyloctahydronaphthalene-1,7-dione **7b**..37
(±)-(1S,4aS,8aS)-8a-methyl-7-oxodecahydronaphthalen-1-yl benzoate **8b**...38
(±)-(4aS,8S,8aS)-8-((tert-butyldimethylsilyl)oxy)-8a-methyloctahydronaphthalen-2(1H)-one **9b** 39
(±)-(1S,4aS,8aS)-8a-methyl-7-oxodecahydronaphthalen-1-yl 3-methylbut-2-enoate **10b** 40
(±)-(1S,4aS,8aS)-8a-methyl-7-oxodecahydronaphthalen-1-yl (tert-butoxycarbonyl)glycinate **11b**....................41
(±)-(1S,4aS,8aS)-8a-methyl-7-oxodecahydronaphthalen-1-yl 2-(1,3-dioxoisoindolin-2-yl)acetate **12b** 42
(±)-(3S,3aS,7aS)-3-hydroxy-3a-methyloctahydro-5H-inden-5-one **13b**, (±)-(1S,3aR,7aS)-1-hydroxy-7a-methyloctahydro-4H-inden-4-one **13c**...43
(±)-(3aR,7aS)-7a-methylhexahydro-1H-indene-1,6(2H)-dione **14b** & 44
(±)-(3aR,7aS)-7a-methylhexahydro-1H-indene-1,4(2H)-dione **14c** 44
(5S,8R,9S,10S,13S,14S,17S)-17-hydroxy-10,13-dimethylhexadecahydro-2H-cyclopenta[a]phenanthren-2-one **15b** (from Androstanolone)...45
(5S,8R,9S,10S,13S,14S,17S)-17-hydroxy-10,13,17-trimethylhexadecahydro-2H-cyclopenta[a]phenanthren-2-one **16b** (from Mestanolone) 46
(5S,8R,9S,10S,13S,14S)-10,13-dimethyltetradecahydro-1H-cyclopenta[a]phenanthrene-2,17-dione **17b** (from Androstanedione) 47
(5S,8R,9S,10S,13S,14S,17S)-17-hydroxy-10,13,17-trimethylhexadecahydro-2H-cyclopenta[a]phenanthren-2-one **18b** (from Allopregnanedione)..48

7. DEUTERIUM LABELING STUDIES.. 49

Standard reaction conditions in methanol..49
Standard reaction conditions in methanol without sulfur 52

8. COMPUTATIONAL DETAILS 55

4,4-dimethylcyclohexanone **1a** (B3LYP) 57
4,4-dimethylcyclohexanone **1a** (PBE0)...58
3,3-dimethylcyclohexanone **1b** (B3LYP) 59
3,3-dimethylcyclohexanone **1b** (PBE0)...60
2,2-dimethylcyclohexanone **1c** (B3LYP)..61
2,2-dimethylcyclohexanone **1c** (PBE0) 62

9. CRYSTALLOGRAPHIC DATA.. 63

(±)-(1S,4aS,8aS)-8a-methyl-7-oxodecahydronaphthalen-1-yl benzoate **8b**...63
(±)-(1S,4aS,8aS)-8a-methyl-7-oxodecahydronaphthalen-1-yl 3-methylbut-2-enoate **10b** 64
(5S,8R,9S,10S,13S,14S,17S)-17-hydroxy-10,13-dimethylhexadecahydro-2H-cyclopenta[a]phenanthren-2-one **15b** 65
(5S,8R,9S,10S,13S,14S,17S)-17-hydroxy-10,13,17-trimethylhexadecahydro-2H-cyclopenta[a]phenanthren-2-one **16b** 66

(5S,8R,9S,10S,13S,14S)-10,13-dimethyltetradecahydro-1H-cyclopenta[a]phenanthrene-2,17-dione ***17b***.........*67*
(5S,8R,9S,10S,13S,14S,17S)-17-acetyl-10,13-dimethylhexadecahydro-2H-cyclopenta[a]phenanthren-2-one ***18b***
..68

10. NMR SPECTRA..69

1. General information

All reagents, unless otherwise stated, were used as supplied from commercial sources without further purification. Solvents were dried using a LC Technology Solutions solvent purification system under an atmosphere of N_2 (H_2O content < 10 ppm, as determined by Karl-Fischer titration) and stored over molecular sieves. Methyl vinyl ketone was distilled before use.

All glassware were dried for at least one hour in an oven set at 120 °C prior to use.

NMR: ^1H and ^{13}C NMR spectra were recorded on a Bruker AV III 400 MHz Ascend, AV-NEO 500 MHz or AV III 300 MHz and are reported in parts per million (ppm). 2D NMR spectra, affiliated ^1H and ^{13}C NMR spectra, as well as ^2H NMR spectra were recorded on a Bruker AV III HD 600. ^1H NMR spectra are calibrated with respect to the corresponding solvent residual peak ($CHCl_3$: 7.26 ppm). ^{13}C NMR spectra were recorded with complete proton decoupling and the spectra are calibrated with respect to the corresponding solvent residual peak ($^{13}CDCl_3$: 77.16 ppm, t). Multiplet signals are reported as follows: s = singlet, d = doublet, t = triplet, q = quartet, quint = quintet, sept = septet, m = multiplet, br = broad, or combinations thereof. ^{13}C signals are singlets unless otherwise stated. For selected compounds the structural assignment of carbon and proton signals was proposed based on analysis of the 2D NMR spectra

High resolution mass spectrometry (HRMS): HRMS data were obtained by the mass spectrometry service in the Laboratory für Organisch Chemie at ETH Zürich on VG-TRIBRIB for electron impact ionization (EI), a Varian IonSpec Spectrometer for electrospray ionization (ESI) or an IonSpec Ultima Fourier Transform Mass Spectrometer for matrix-assisted laser desorption/ionization (MALDI) and are reported as (m/z).

Reverse-phase high pressure liquid chromatography (RP-HPLC): Analytical RP-HPLC was performed on an Agilent Infinity II 1260 using C18 column (5 µm, 250 x 4.6 mm), with a flow of 1 mL/min. Preparative RP-HPLC was performed on an Agilent Infinity II 1260 with C18 column (7 µm, 250 x 21 mm) with a flow of 20 mL/min using a gradient of MeCN in H_2O with 0.1% TFA or formic acid.

Gas chromatography (GC): GC were recorded on a Shimadzu GC-2025 (capillary column: Macherey-Nagel OPTIMA 5, 30.0 m × 0.25 × 0.25 µm; carrier gas: H_2). To determine GC yields, calibration curves using dodecane as an internal standard were generated.

Chromatography: Analytical thin layer chromatography was performed using silica gel 60 F254 coated aluminium sheets (Merck). Visualization was achieved by ultraviolet fluorescence (λ = 254 nm) and/or staining with potassium permanganate ($KMnO_4$). Automated flash column chromatography was performed on a Biotage Isolera One system with Sfär columns or manually using silica gel 60 (pore size = 60 Å, mesh: 40-63 µm from Sigma-Aldrich or SiliCycle).

X-Ray analysis: Single crystalline samples were measured on a Riguka Oxford Diffraction XtaLAB Synergy-S Dualflex kappa diffractometer equipped with a Dectris Pilatus 300 HPAD detector and using microfocus sealed tube Cu-Kα radiation with mirror optics (λ = 1.54178 Å). All measurements were carried out at 100 K (unless otherwise noted) using an Oxford Cryosystems Cryostream 800 sample cryostat. Data collected on the Rigaku instrument were integrated using CrysAlisPro and corrected for absorption effects using a combination of empirical (ABSPACK) and numerical corrections.[1] The structures were solved using SHELXT[2] or SHELXS[3] and refined by full-matrix least-squares analysis (SHELXL)[4] using the program package OLEX2[5]. Unless otherwise indicated below, all non-hydrogen atoms were refined anisotropically and hydrogen atoms were constrained to ideal geometries and refined with fixed isotropic displacement parameters (in terms of a riding model).

Optical Rotation: Optical rotations were recorded on a Jasco P-2000 polarimeter using a 1.5-mL cell with 100 mm length (l). α_D^T values are reported in deg mL g^{-1} dm^{-1}. Concentrations I are quoted in g/100 mL (i.e. 10 mg/mL) with the appropriate number of significant figures; D refers to the D line of sodium (589 nm); temperatures (T) are given in degrees Celsius (°C). The formula used to calculate the specific rotation α_D^T from the optical rotation α is as follows: α_D^T = [α (°) × 100] / [l (dm) × c (g/100 mL)].

References

1. Rigaku, "CrysAlis^{Pro}" (2016); https://rigaku.com/products/crystallography/crysalis
2. G. M. Sheldrick, SHELXT - integrated space-group and crystal-structure determination. *Acta Crystallogr. A Found. Adv.* **71**, 3–8 (2015). doi.org/10.1107/S2053273314026370 Medline
3. G. M. Sheldrick, A short history of SHELX. *Acta Crystallogr. A* **64**, 112–122 (2008). doi:10.1107/S0108767307043930 Medline
4. M. Sheldrick, Crystal structure refinement with SHELXL. Acta Crystallogr. C Struct. Chem. 71, 3–8 (2015). doi:10.1107/S2053229614024218 Medline
5. O. V. Dolomanov, L. J. Bourhis, R. J. Gildea, J. A. K. Howard, H. Puschmann, OLEX2: A complete structure solution, refinement and analysis program. *J. Appl. Cryst.* **42**, 339–341 (2009). doi:10.1107/S0021889808042726

2. Current mechanistic hypothesis

Scheme S1 shows our current *tentative* hypothesis for the mechanism of the ketone chain-walking isomerization to help the reader. This is largely based on the proposed mechanism for the Willgerodt-Kindler reaction (Priebbenow, D. L.; Bolm, C. *Chem. Soc. Rev.* **2013**, *42*, 7870–7880. DOI: 10.1039/C3CS60154D). However, we would like to stress that the mechanism of the Willgerodt-Kindler reaction is still poorly understood and alternative mechanisms have been suggested. Further investigations are required to understand the mechanism of the ketone chain-walking isomerization.

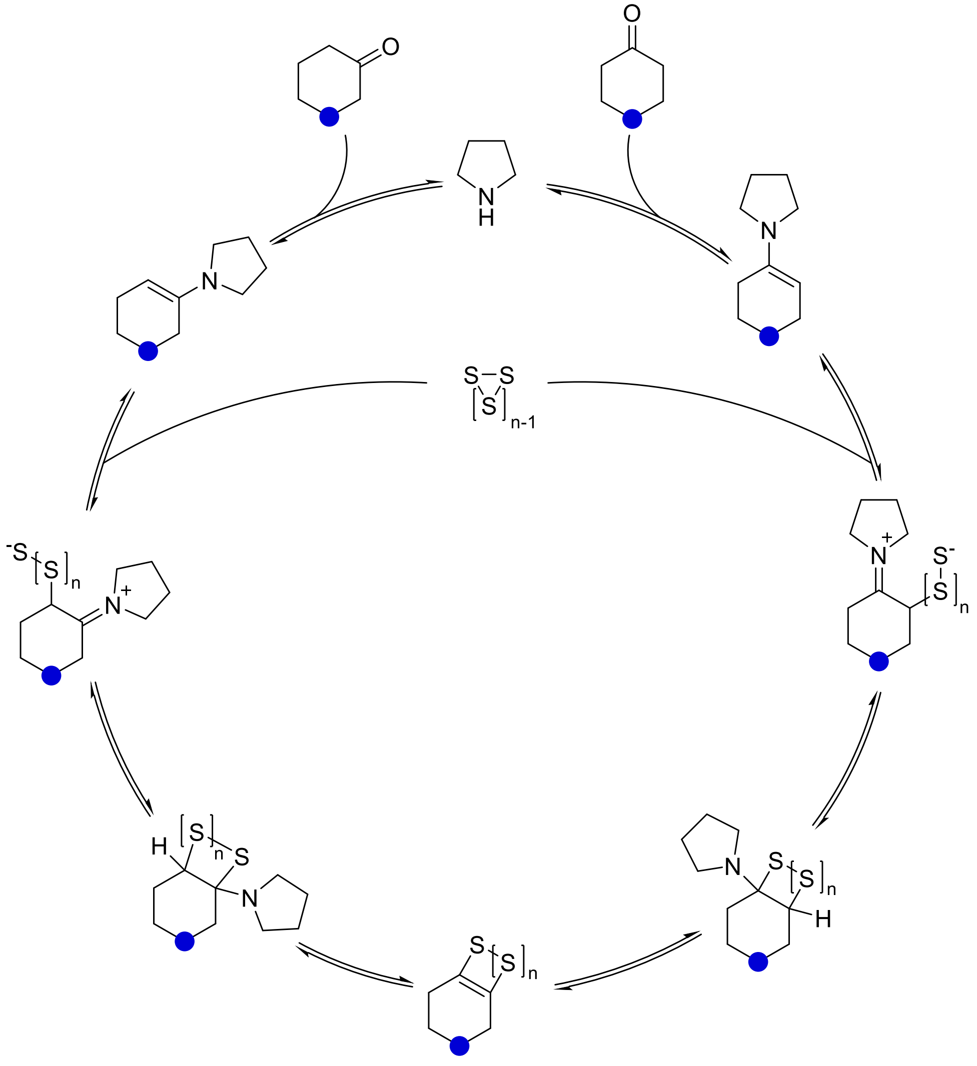

Scheme S1. Tentative mechanistic proposal for the ketone chain-walking isomerization

3. Reaction optimization

General procedure for investigating amine catalysts

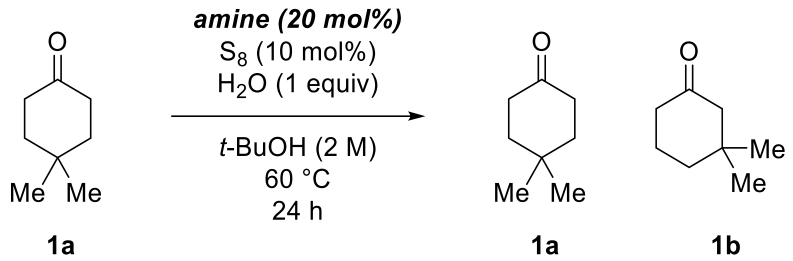

To an oven-dried screw-cap vial was added 4,4-dimethylcyclohexanone **1a** (63 mg, 0.50 mmol), S_8 (6.4 mg, 25 µmol, 5 mol%, 40 mol% S), *t*-BuOH (0.25 mL), H_2O (9 µL, 0.5 mmol, 1 equiv) and the indicated amine (0.10 mmol, 20 mol%). The vial was flushed with N_2, sealed and then heated at 60 °C for 24 hours. After cooling to room temperature, the reaction was diluted with MTBE (4 mL) and dodecane (50 µL) was added as an internal standard. An aliquot (0.5 mL) of the crude mixture was filtered through a plug of silica and then analyzed by GC-FID analysis.

Figure S1. Amine catalysts tested

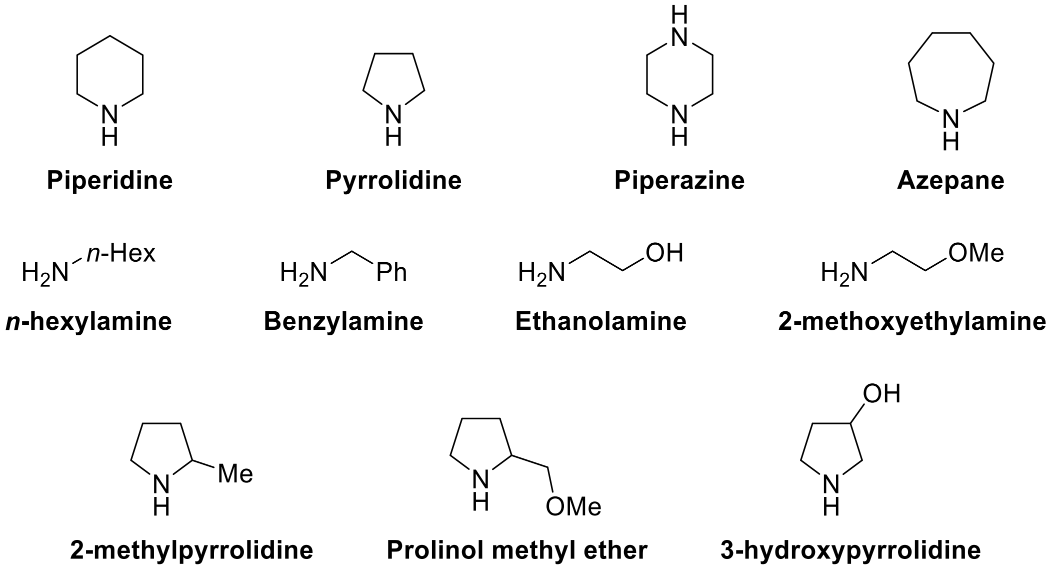

Table S1: Results of amine catalyst testing

Amine	Yield 1a	Yield 1b	1a:1b	Total Yield
Piperidine	40%	26%	1:0.65	66%
**Pyrrolidine**	**9%**	**54%**	**1:6.0**	**63%**
Piperazine	70%	8%	1:0.11	78%
Azepane	37%	29%	1:0.78	66%
N-hexylamine	36%	17%	1:0.47	53%
Benzylamine	50%	8%	1:0.16	58%
Ethanolamine	43%	8%	1:0.19	51%
2-methoxyethylamine	42%	19%	1:0.45	61%
2-methylpyrrolidine	25%	40%	1:1.6	65%
Prolinol methyl ether	48%	15%	1:0.31	63%
3-hydroxypyrrolidine	14%	43%	1:3.1	57%

Figure S2. Amine catalysts that gave negligible conversion to 1b (<5% GC yield of 1b)

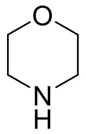

Morpholine

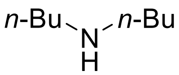

Dibutylamine

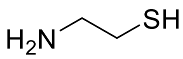

Cysteamine

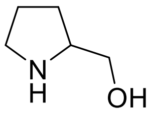

Prolinol

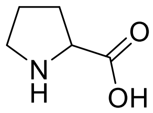

Proline

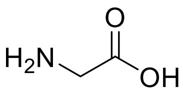

Glycine

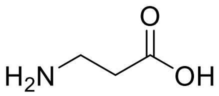

β-alanine

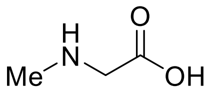

Sarcosine

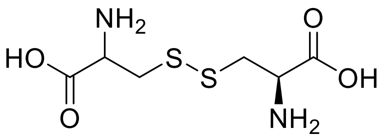

Cystine

General procedure for investigating temperature/sulfur loading effect

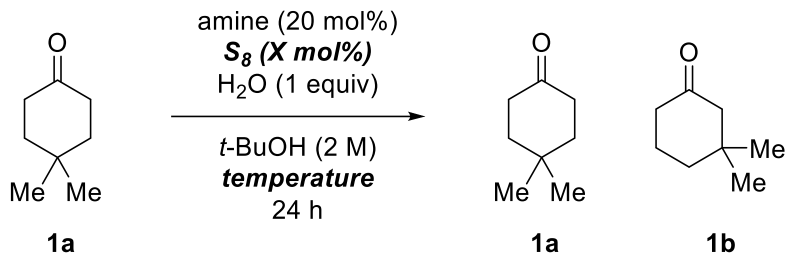

To an oven-dried screw-cap vial was added 4,4-dimethylcyclohexanone **1a** (126 mg, 1.0 mmol), the indicated amount of S_8, *t*-BuOH (0.5 mL), H_2O (18 µL, 1.0 mmol, 1 equiv) and pyrrolidine (16 µL, 0.10 mmol, 20 mol%). The vial was flushed with N_2, sealed and then heated at the indicated temperature for 24 hours. After cooling to room temperature, the reaction was diluted with MTBE (4 mL) and dodecane (50 µL) was added as an internal standard. An aliquot (0.5 mL) of the crude mixture was filtered through a plug of silica and then analyzed by GC-FID analysis.

Table S2: Results of temperature/sulfur loading testing

Temperature	S_8 loading	Yield 1a	Yield 1b	1a:1b	Total Yield
60 °C	1 mol%	56%	18%	1:0.32	74%
60 °C	2.5 mol%	13%	60%	1:4.6	73%
60 °C	5 mol%	9%	54%	1:6.0	63%
60 °C	10 mol%	7%	45%	1:6.4	52%
80 °C	1 mol%	38%	37%	1:0.97	75%
**80 °C**	**2.5 mol%**	**11%**	**60%**	**1:5.5**	**71%**
80 °C	5 mol%	9%	50%	1:5.6	59%
80 °C	10 mol%	5%	34%	1:6.8	39%

General procedure for investigating solvent effect

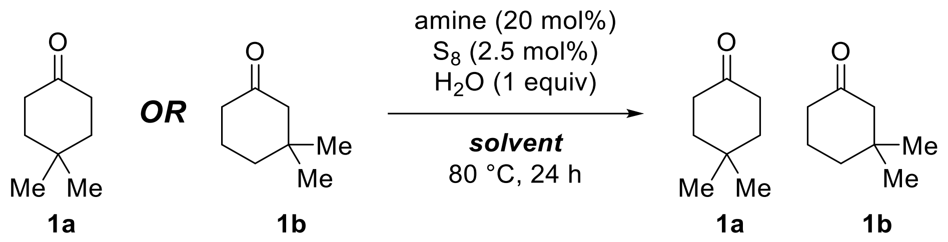

To an oven-dried screw-cap vial was added 4,4-dimethylcyclohexanone **1a** (63 mg, 0.50 mmol) or 3,3-dimethylcyclohexanone **1b** (69 µL, 0.50 mmol), S_8 (3.2 mg, 12.5 µmol, 2.5 mol%, 20 mol% S), the indicated solvent (0.25 mL), H_2O (9 µL, 0.5 mmol, 1 equiv) and pyrrolidine (8 µL, 0.10 mmol, 20 mol%). The vial was flushed with N_2, sealed and then heated at 80 °C for 24 hours. After cooling to room temperature, the reaction was diluted with MTBE (4 mL) and dodecane (50 µL) was added as an internal standard. An aliquot (0.5 mL) of the crude mixture was filtered through a plug of silica and then analyzed by GC-FID analysis.

Table S3: Results of solvent effect investigation

	From 1a				From 1b			
**Solvent**	**Yield 1a**	**Yield 1b**	**1a:1b**	**Total Yield**	**Yield 1a**	**Yield 1b**	**1a:1b**	**Total Yield**
t-BuOH	11%	60%	1:5.5	71%	9%	57%	1:6.3	66%
MeOH	11%	54%	1:4.9	65%	*Reaction not performed*			
EtOH	11%	55%	1:5.0	66%	9%	56%	1:6.2	65%
**i-PrOH**	**10%**	**58%**	**1:5.8**	**68%**	**9%**	**56%**	**1:6.2**	**65%**
n-BuOH	22%	44%	1:2.0	66%	*Reaction not performed*			
Dioxane	14%	53%	1:3.8	67%	10%	52%	1:5.2	62%
H_2O*	56%	5%	1:0.1	61%	*Reaction not performed*			
n-Heptane	67%	1%	1:0.01	68%	*Reaction not performed*			
PhMe	61%	4%	1:0.07	65%	*Reaction not performed*			
DCE	80%	0%	-	80%	*Reaction not performed*			
MeCN	22%	53%	1:2.4	75%	10%	65%	1:6.5	75%
DMF	11%	55%	1:5.0	66%	10%	51%	1:5.1	61%

* 0.25 mL of water in total was added

General procedure for investigating the influence of water content and reaction concentration

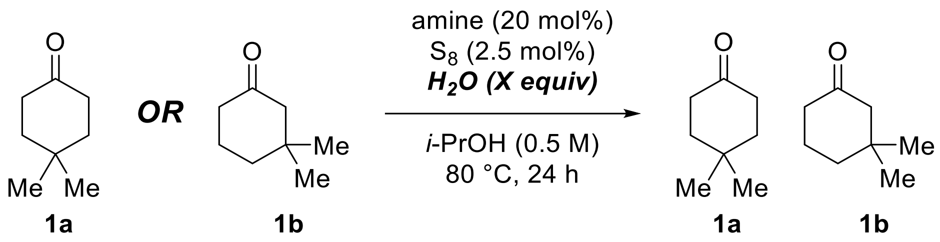

To an oven-dried, screw-cap vial was added 4,4-dimethylcyclohexanone **1a** (63 mg, 0.50 mmol), or 3,3-dimethylcyclohexanone **1b** (63 mg, 0.50 mmol), S_8 (3.2 mg, 0.013 mmol, 2.5 mol%, 20 mol% S), the indicated amount of *i*-PrOH, and the indicated amount of H_2O (X equiv) and pyrrolidine (8 µL, 0.10 mmol, 20 mol%). The vial was flushed with N_2, sealed and then heated at 80 °C for 24 hours. After cooling to room temperature, the reaction was diluted with MTBE (2.5 mL) and dodecane (50 µL) was added as an internal standard. An aliquot (0.1 mL) of the crude mixture was filtered through a plug of silica and then analyzed by GC-FID analysis.

Table S4: Results of water content and reaction concentration testing

		From 1a				From 1b			
**Amount *i*-PrOH (concentration)**	**Equivalents H_2O**	**Yield 1a**	**Yield 1b**	**1a:1b**	**Total Yield**	**Yield 1a**	**Yield 1b**	**1a:1b**	**Total Yield**
1.0 mL (0.5 M)	2	10%	60%	1:6.0	70%	9%	57%	1:6.3	66%
1.0 mL (0.5 M)	3	10%	60%	1:6.0	70%	9%	60%	1:6.7	69%
**1.0 mL (0.5 M)**	**5**	**10%**	**63%**	**1:6.3**	**73%**	**10%**	**61%**	**1:6.1**	**71%**
1.0 mL (0.5 M)	7.5	12%	64%	1:5.3	76%	10%	62%	1:6.2	72%
2.0 mL (0.25 M)	2	9%	58%	1:6.4	67%	8%	52%	1:6.5	60%
2.0 mL (0.25 M)	3	9%	57%	1:6.3	66%	9%	57%	1:6.3	66%
2.0 mL (0.25 M)	5	10%	59%	1:5.9	69%	9%	59%	1:6.6	68%
2.0 mL (0.25 M)	7.5	10%	60%	1:6.0	70%	9%	60%	1:6.7	69%

4. Isomerisation of dimethyl-substituted and mono-substituted cyclohexanones

*Isomerization of 4,4-dimethylcyclohexanone **1a***

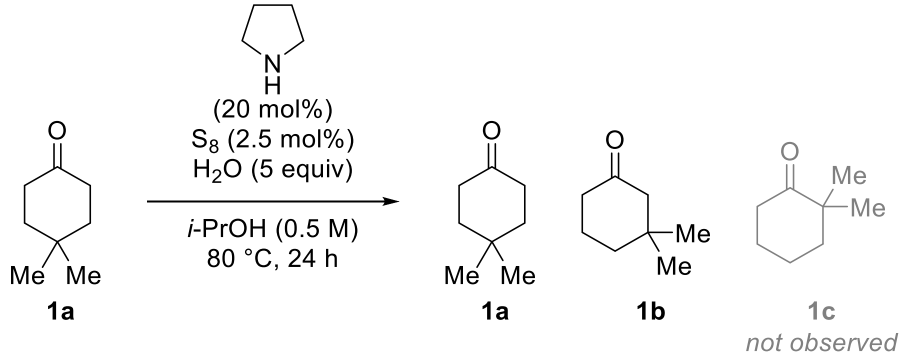

To an oven-dried screw-cap vial was added 4,4-dimethylcyclohexanone **1a** (63 mg, 0.50 mmol), S_8 (3.2 mg, 0.013 mmol, 2.5 mol%, 20 mol% S), *i*-PrOH (1.0 mL), and H_2O (45 µL, 2.5 mmol, 5 equiv) and pyrrolidine (8 µL, 0.05 mmol, 20 mol%). The vial was flushed with N_2, sealed and then heated at 80 °C for 24 hours. After cooling to room temperature, the reaction was diluted with MTBE (2.5 mL) and dodecane (50 µL) was added as an internal standard. An aliquot (0.1 mL) of the crude mixture was filtered through a plug of silica and then analyzed by GC-FID analysis.

GC yield **1a** = 10%; GC yield **1b** = 63%; GC yield **1c** = 0%

*Isomerization of 3,3-dimethylcyclohexanone **1b***

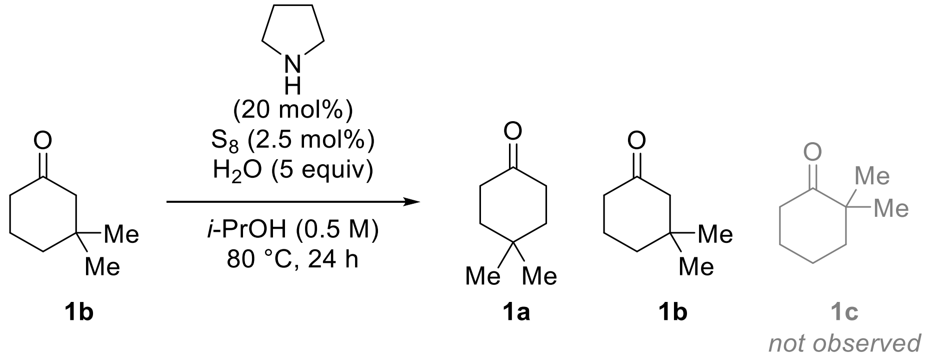

To an oven-dried screw-cap vial was added 3,3-dimethylcyclohexanone **1b** (63 mg, 0.50 mmol), S_8 (3.2 mg, 0.013 mmol, 2.5 mol%, 20 mol% S), *i*-PrOH (1.0 mL), and H_2O (45 µL, 2.5 mmol, 5 equiv) and pyrrolidine (8 µL, 0.05 mmol, 20 mol%). The vial was flushed with N_2, sealed and then heated at 80 °C for 24 hours. After cooling to room temperature, the reaction was diluted with MTBE (2.5 mL) and dodecane (50 µL) was added as an internal standard. An aliquot (0.1 mL) of the crude mixture was filtered through a plug of silica and then analyzed by GC-FID analysis.

GC yield **1a** = 10%; GC yield **1b** = 61%; GC yield **1c** = 0%

*Isomerization of 2,2-dimethylcyclohexanone **1c***

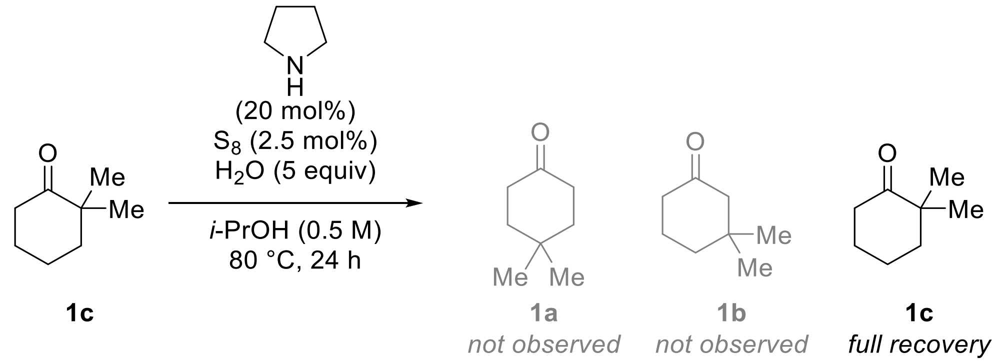

To an oven-dried screw-cap vial was added 2,2-dimethylcyclohexanone **1c** (69 µL, 0.50 mmol), S_8 (3.2 mg, 0.013 mmol, 2.5 mol%, 20 mol% S), *i*-PrOH (1.0 mL), and H_2O (45 µL, 2.5 mmol, 5 equiv) and pyrrolidine (8 µL, 0.05 mmol, 20 mol%). The vial was flushed with N_2, sealed and then heated at 80 °C for 24 hours. After cooling to room temperature, the reaction was diluted with MTBE (2.5 mL) and dodecane (50 µL) was added as an internal standard. An aliquot (0.1 mL) of the crude mixture was filtered through a plug of silica and then analyzed by GC-FID analysis.

GC yield **1a** = 0%; GC yield **1b** = 0%; GC yield **1c** = 100%

*Isomerization of 4-tert-butylcyclohexanone **2a***

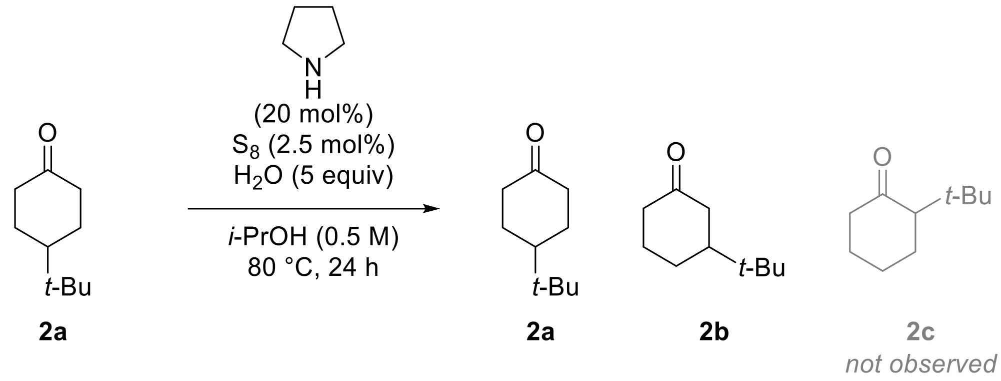

To an oven-dried screw-cap vial was added 4-*tert*-butylcyclohexanone **2a** (154 mg, 1.00 mmol), S_8 (6.4 mg, 0.025 mmol, 2.5 mol%, 20 mol% S), *i*-PrOH (2.0 mL), and H_2O (90 µL, 5.0 mmol, 5 equiv) and pyrrolidine (16 µL, 0.10 mmol, 20 mol%). The vial was flushed with N_2, sealed and then heated at 80 °C for 24 hours. After cooling to room temperature, the reaction was diluted with MTBE (2.5 mL) and dodecane (100 µL) was added as an internal standard. An aliquot (0.1 mL) of the crude mixture was filtered through a plug of silica and then analyzed by GC-FID analysis.

GC yield **2a** = 21%; GC yield **2b** = 57%; GC yield **2c** = 0%

*Isomerization of 3-tert-butylcyclohexanone **2b***

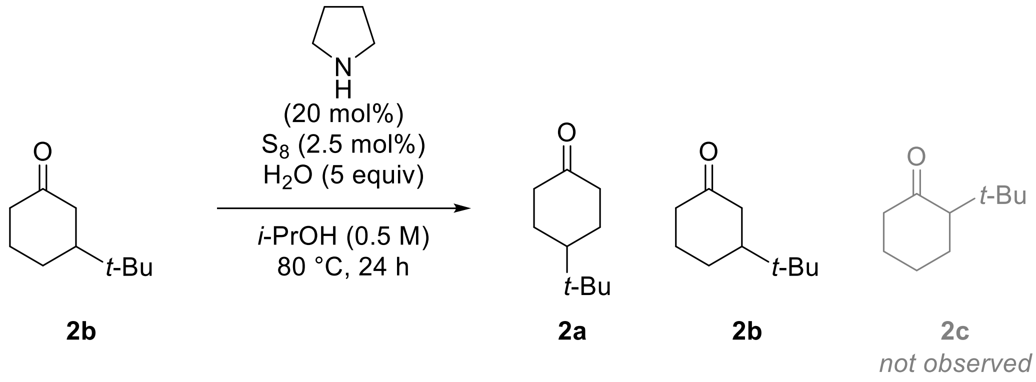

To an oven-dried screw-cap vial was added 3-*tert*-butylcyclohexanone **2b** (154 mg, 1.00 mmol), S_8 (6.4 mg, 0.025 mmol, 2.5 mol%, 20 mol% S), *i*-PrOH (2.0 mL), and H_2O (90 µL, 5.0 mmol, 5 equiv) and pyrrolidine (16 µL, 0.10 mmol, 20 mol%). The vial was flushed with N_2, sealed and then heated at 80 °C for 24 hours. After cooling to room temperature, the reaction was diluted with MTBE (2.5 mL) and dodecane (100 µL) was added as an internal standard. An aliquot (0.1 mL) of the crude mixture was filtered through a plug of silica and then analyzed by GC-FID analysis.

GC yield **2a** = 14%; GC yield **2b** = 62%; GC yield **2c** = 0%

*Isomerization of 2-tert-butylcyclohexanone **2c***

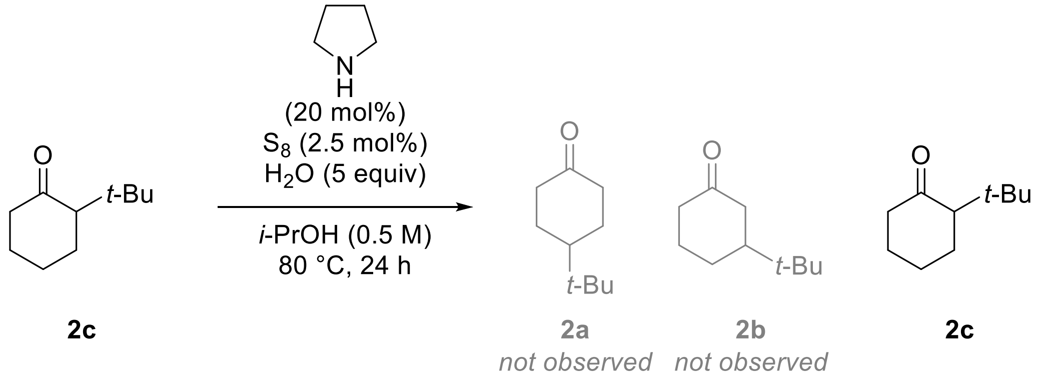

To an oven-dried screw-cap vial was added 2-*tert*-butylcyclohexanone **2c** (172 µL, 1.00 mmol), S_8 (6.4 mg, 0.025 mmol, 2.5 mol%, 20 mol% S), *i*-PrOH (2.0 mL), and H_2O (90 µL, 5.0 mmol, 5 equiv) and pyrrolidine (16 µL, 0.10 mmol, 20 mol%). The vial was flushed with N_2, sealed and then heated at 80 °C for 24 hours. After cooling to room temperature, the reaction was diluted with MTBE (2.5 mL) and dodecane (100 µL) was added as an internal standard. An aliquot (0.1 mL) of the crude mixture was filtered through a plug of silica and then analyzed by GC-FID analysis.

GC yield **2a** = 0%; GC yield **2b** = 0%; GC yield **2c** = 100%

Isomerization of 4-methylcyclohexanone ***3a***

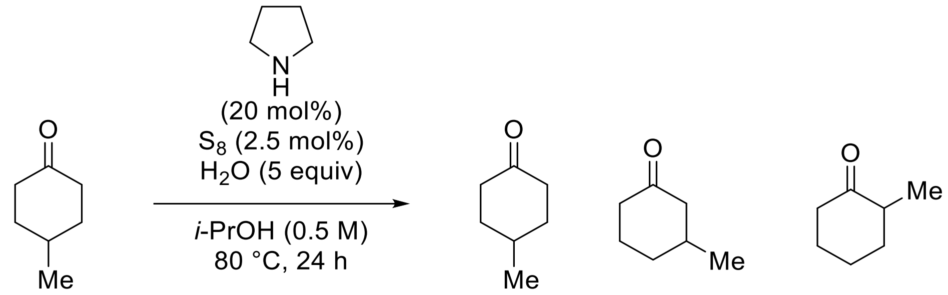

3a **3a** **3b** **3c**

To an oven-dried screw-cap vial was added 4-methylcyclohexanone **3a** (61 µL, 0.50 mmol), S_8 (3.2 mg, 0.012 mmol, 2.5 mol%, 20 mol% S), *i*-PrOH (1.0 mL), and H_2O (45 µL, 2.5 mmol, 5 equiv) and pyrrolidine (8 µL, 0.10 mmol, 20 mol%). The vial was flushed with N_2, sealed and then heated at 80 °C for 24 hours. After cooling to room temperature, the reaction was diluted with MTBE (2.5 mL) and dodecane (50 µL) was added as an internal standard. An aliquot (0.1 mL) of the crude mixture was filtered through a plug of silica and then analyzed by GC-FID analysis.

GC yield **3a** = 33%; GC yield **3b** = 40%; GC yield **3c** = 1%

Isomerization of 3-methylcyclohexanone ***3b***

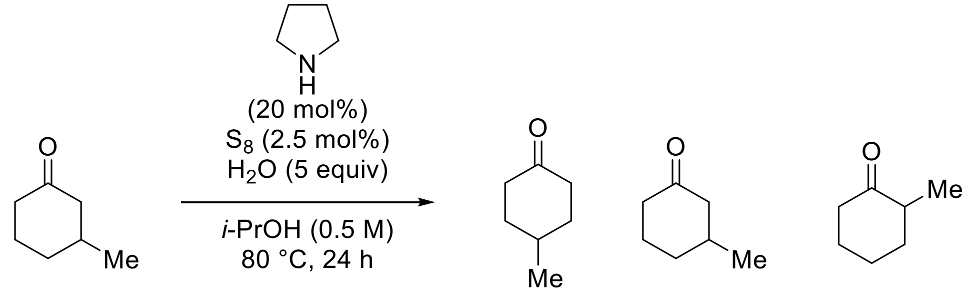

3b **3a** **3b** **3c**

To an oven-dried screw-cap vial was added 3-methylcyclohexanone **3b** (61 µL, 0.50 mmol), S_8 (3.2 mg, 0.012 mmol, 2.5 mol%, 20 mol% S), *i*-PrOH (1.0 mL), and H_2O (45 µL, 2.5 mmol, 5 equiv) and pyrrolidine (8 µL, 0.10 mmol, 20 mol%). The vial was flushed with N_2, sealed and then heated at 80 °C for 24 hours. After cooling to room temperature, the reaction was diluted with MTBE (2.5 mL) and dodecane (50 µL) was added as an internal standard. An aliquot (0.1 mL) of the crude mixture was filtered through a plug of silica and then analyzed by GC-FID analysis.

GC yield **3a** = 15%; GC yield **3b** = 56%; GC yield **3c** = 2%

*Isomerization of 2-methylcyclohexanone **3c***

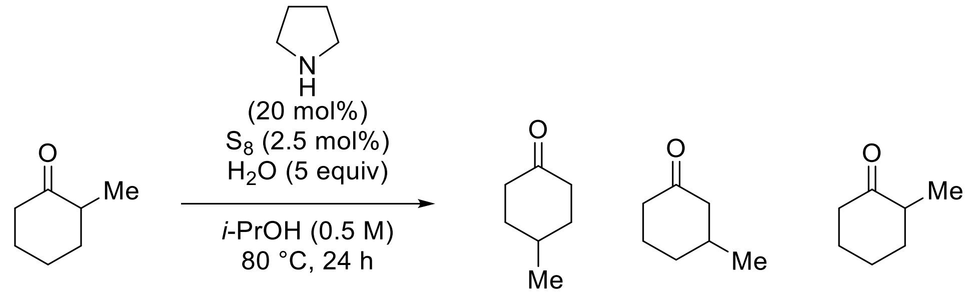

3c **3a** **3b** **3c**

To an oven-dried screw-cap vial was added 2-methylcyclohexanone **3c** (61 µL, 0.50 mmol), S_8 (3.2 mg, 0.012 mmol, 2.5 mol%, 20 mol% S), *i*-PrOH (1.0 mL), and H_2O (45 µL, 2.5 mmol, 5 equiv) and pyrrolidine (8 µL, 0.10 mmol, 20 mol%). The vial was flushed with N_2, sealed and then heated at 80 °C for 24 hours. After cooling to room temperature, the reaction was diluted with MTBE (2.5 mL) and dodecane (50 µL) was added as an internal standard. An aliquot (0.1 mL) of the crude mixture was filtered through a plug of silica and then analyzed by GC-FID analysis.

GC yield **3a** = 3%; GC yield **3b** = 11%; GC yield **3c** = 68%

*Isomerization of 4-phenylcyclohexanone **4a***

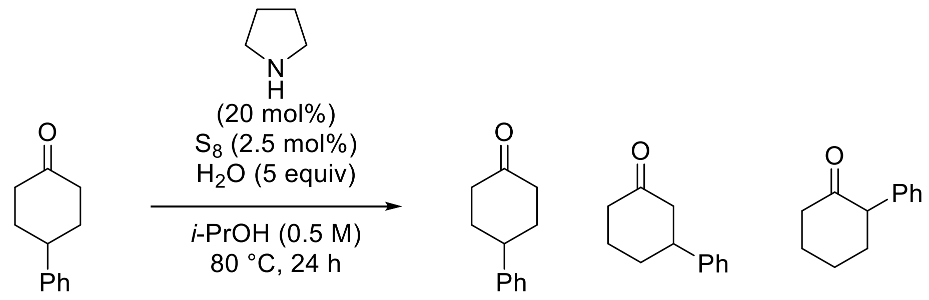

4a **4a** **4b** **4c**

To an oven-dried screw-cap vial was added 4-phenylcyclohexanone **4a** (174 mg, 1.00 mmol), S_8 (6.4 mg, 0.025 mmol, 2.5 mol%, 20 mol% S), *i*-PrOH (2.0 mL), and H_2O (90 µL, 5.0 mmol, 5 equiv) and pyrrolidine (16 µL, 0.10 mmol, 20 mol%). The vial was flushed with N_2, sealed and then heated at 80 °C for 24 hours. After cooling to room temperature, the reaction was diluted with MTBE (2.5 mL) and dodecane (100 µL) was added as an internal standard. An aliquot (0.1 mL) of the crude mixture was filtered through a plug of silica and then analyzed by GC-FID analysis.

GC yield **4a** = 30%; GC yield **4b** = 31%; GC yield **4c** < 1% (*trace amounts detected)*

Isomerization of 3-phenylcyclohexanone ***4b***

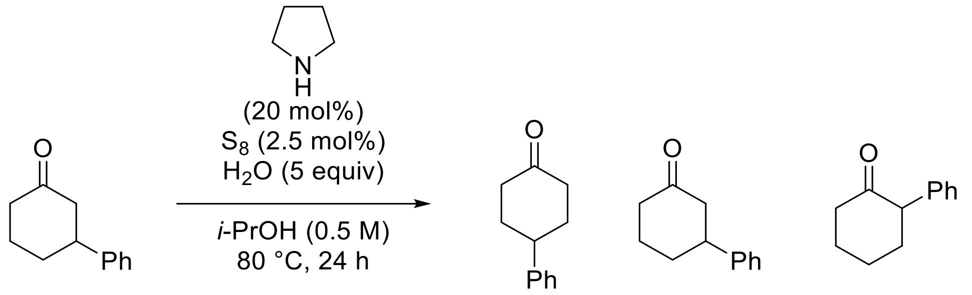

4b **4a** **4b** **4c**

To an oven-dried screw-cap vial was added 3-phenylcyclohexanone **4b** (174 mg, 1.00 mmol), S_8 (6.4 mg, 0.025 mmol, 2.5 mol%, 20 mol% S), *i*-PrOH (2.0 mL), and H_2O (90 µL, 5.0 mmol, 5 equiv) and pyrrolidine (16 µL, 0.10 mmol, 20 mol%). The vial was flushed with N_2, sealed and then heated at 80 °C for 24 hours. After cooling to room temperature, the reaction was diluted with MTBE (2.5 mL) and dodecane (100 µL) was added as an internal standard. An aliquot (0.1 mL) of the crude mixture was filtered through a plug of silica and then analyzed by GC-FID analysis.

GC yield **4a** = 11%; GC yield **4b** = 53%; GC yield **4c** < 1% (*trace amounts detected)*

Isomerization of 2-phenylcyclohexanone ***4c***

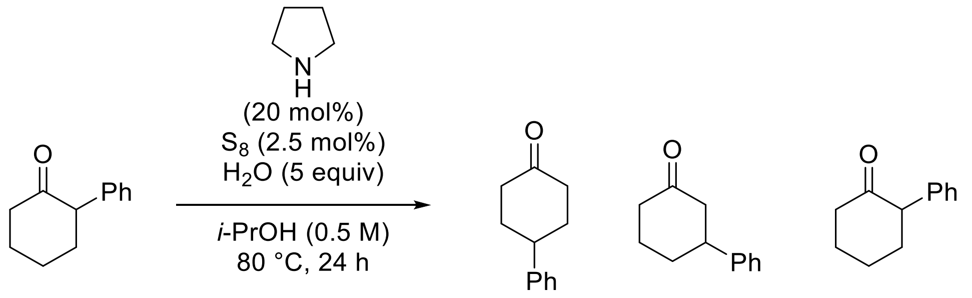

4c **4a** **4b** **4c**

To an oven-dried screw-cap vial was added 2-phenylcyclohexanone **4c** (174 mg, 1.00 mmol), S_8 (6.4 mg, 0.025 mmol, 2.5 mol%, 20 mol% S), *i*-PrOH (2.0 mL), and H_2O (90 µL, 5.0 mmol, 5 equiv) and pyrrolidine (16 µL, 0.10 mmol, 20 mol%). The vial was flushed with N_2, sealed and then heated at 80 °C for 24 hours. After cooling to room temperature, the reaction was diluted with MTBE (2.5 mL) and dodecane (100 µL) was added as an internal standard. An aliquot (0.1 mL) of the crude mixture was filtered through a plug of silica and then analyzed by GC-FID analysis.

GC yield **4a** = 4%; GC yield **4b** = 13%; GC yield **4c** = 49%

5. Synthesis and characterization of substrates

*(±)-3-tert-butylcyclohexanone **2b***

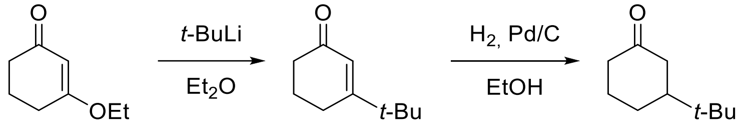

S1 **2b**

To a solution of 3-ethoxy-2-cyclohexen-1-one (1.40 mL, 9.6 mmol, 1 equiv) in anhydrous Et_2O (15 mL) was added dropwise *tert*-butyllithium (5.8 mL, 1.9 M in pentane, 11 mmol, 1.1 equiv) at -78 °C under an atmosphere of N_2. The reaction mixture was stirred at -78 °C for 30 minutes and then quenched by addition of water (1 mL). After warming to room temperature, saturated NH_4Cl aqueous solution was added and the layers were separated. The aqueous phase was extracted with Et_2O and the combined organic layers were washed with brine and dried over Na_2SO_4. After removal of the solvents under reduced pressure, the residue was purified by automated flash column chromatography (SiO_2, 0–20% EtOAc in hexanes) to afford the product **S1** as a pale golden-yellow oil (1.15 g, 7.6 mmol, 79% yield).

^{1}H NMR (400 MHz, $CDCl_3$) δ 5.95 (s, 1H), 2.40 – 2.31 (m, 4H), 2.02 – 1.91 (m, 2H), 1.12 (s, 9H).

^{13}C NMR (101 MHz, $CDCl_3$) δ 200.8, 173.8, 123.0, 37.5, 36.8, 28.3, 25.9, 23.3.

Consistent with reported data:
Coote, S. C.; O'Brien, P.; Whitwood, A. C. Stereoselective Aziridination of Cyclic Allylic Alcohols Using Chloramine-T. *Org. Biomol. Chem.* **2008**, *6*, 4299.
https://doi.org/10.1039/b811137e

A suspension of **S1** (1.15 g, 7.6 mmol) and 10% Pd/C (58 mg) in EtOH (15 mL) was stirred for 18 hours at room temperature under a H_2 atmosphere. After filtration through celite, the solvents were removed under reduced pressure and the residue was purified by automated flash column chromatography (SiO_2, 0–5% EtOAc in hexanes) to afford the product **2b** as a colourless oil (453 mg, 2.94 mmol, 39% yield).

^{1}H NMR (400 MHz, $CDCl_3$) δ 2.44 (ddt, *J* = 13.6, 3.7, 2.2 Hz, 1H), 2.35 (ddtd, *J* = 14.1, 4.6, 2.3, 1.5 Hz, 1H), 2.22 (tdd, *J* = 14.1, 6.4, 1.1 Hz, 1H), 2.15 – 2.00 (m, 2H), 1.98 – 1.89 (m, 1H), 1.63 – 1.43 (m, 2H), 1.31 (tdd, *J* = 12.9, 11.9, 3.5 Hz, 1H), 0.89 (s, 9H).

^{13}C NMR (101 MHz, $CDCl_3$) δ 213.1, 49.4, 43.7, 41.4, 32.7, 27.2, 26.1, 25.7.

Consistent with reported data:
Shen, D.; Miao, C.; Wang, S.; Xia, C.; Sun, W. Efficient Benzylic and Aliphatic C–H Oxidation with Selectivity for Methylenic Sites Catalyzed by a Bioinspired Manganese Complex. *Org. Lett.* **2014**, *16*, 1108–1111. https://doi.org/10.1021/ol4037083

*(±)-3-phenylcyclohexanone **4b***

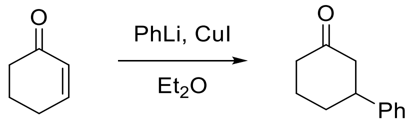

4b

To a solution of copper(I) iodide (1.97 g, 10.3 mmol, 1 equiv) in anhydrous Et_2O (5 mL) was added phenyllithium (10 mL, 1.9 M in dibutyl ether, 19 mmol, 1.8 equiv) under a N_2 atmosphere at 0 °C. The reaction mixture was slowly warmed to room temperature and, after stirring for 10 minutes at room temperature, the solution was cooled to 0 °C and a solution of cyclohexenone (1.00 mL, 10.3 mmol) was added dropwise. After stirring for 15 minutes at 0 °C, the reaction then quenched by addition of saturated NH_4Cl aqueous solution and NH_4OH solution. The layers were separated and the aqueous phase was extracted with EtOAc. The combined organic layers were washed with brine and dried over Na_2SO_4. After removal of the solvents under reduced pressure, the residue was purified by automated flash column chromatography (SiO_2, 0–10% EtOAc in hexanes) to afford the product **4b** as a golden-yellow oil (1.32 g, 7.60 mmol, 74% yield).

^{1}H NMR (400 MHz, $CDCl_3$) δ 7.37 – 7.30 (m, 2H), 7.26 – 7.20 (m, 3H), 3.02 (tt, *J* = 11.7, 4.0 Hz, 1H), 2.64 – 2.52 (m, 2H), 2.51 – 2.33 (m, 2H), 2.21 – 2.05 (m, 2H), 1.92 – 1.71 (m, 2H).

^{13}C NMR (101 MHz, $CDCl_3$) δ 211.0, 144.4, 128.7, 126.7, 126.6, 49.0, 44.8, 41.2, 32.8, 25.6.

Consistent with reported data:
Khiar, N.; Salvador, Á.; Valdivia, V.; Chelouan, A.; Alcudia, A.; Álvarez, E.; Fernández, I. Flexible C2-Symmetric Bis-Sulfoxides as Ligands in Enantioselective 1,4-Addition of Boronic Acids to Electron-Deficient Alkenes. *J. Org. Chem.* **2013**, *78*, 6510–6521.
https://doi.org/10.1021/jo400700m

(±)-(4aS,5S)-5-hydroxy-4a-methyl-4,4a,5,6,7,8-hexahydronaphthalen-2(3H)-one ***S4***

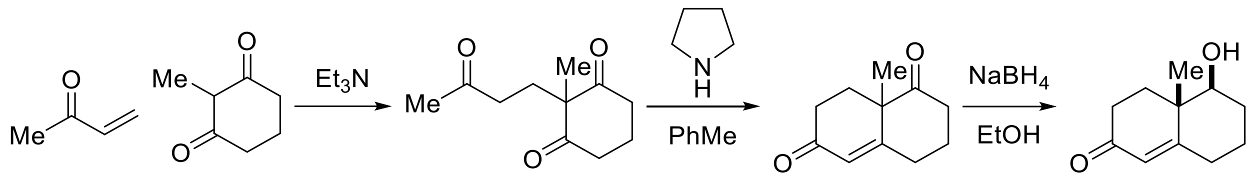

S2 **S3** **S4**

A solution of 2-methyl-cyclohexane-1,3-dione (25 g, 198 mmol) and Et_3N (0.27 mL, 1.9 mmol, 0.01 equiv) was cooled to 0 °C under a N_2 atmosphere and freshly distilled methyl vinyl ketone (19 mL, 230 mmol, 1.16 equiv) was added. The solution was stirred for 16 hours at 25 °C then diluted with EtOAc and activated charcoal (5 g) was added. The suspension was then heated at 45 °C for 30 minutes. After cooling to room temperature, the suspension was filtered and the filtrate was concentrated under reduce pressure to afford the product **S2** as a pale yellow oil (37.8 g, 193 mmol, 97% yield). The crude product was used in the next step without further purification.

^{1}H NMR (500 MHz, $CDCl_3$) δ 2.77 – 2.57 (m, 4H), 2.37 – 2.30 (m, 2H), 2.10 (s, 3H), 2.08 – 1.96 (m, 3H), 1.90 (dtt, *J* = 14.1, 8.8, 5.4 Hz, 1H), 1.23 (s, 3H).

^{13}C NMR (126 MHz, $CDCl_3$) δ 210.2, 207.7, 64.5, 38.5, 37.9, 30.1, 29.7, 20.2, 17.7.

HRMS (ESI) m/z: $[M + Na]^+$ Calculated for $C_{11}H_{16}NaO_3$ 219.0992; Found 219.0990.

A solution of **S2** (37.8 g, 193 mmol) and pyrrolidine (1.5 mL, 18 mmol, 0.09 equiv) in PhMe (100 mL) was heated at reflux with a Dean-Stark apparatus for 3 h. After cooling to room temperature, the organic solution was washed with 10% aqueous HCl solution and water. The combined aqueous layers were extracted with Et_2O and then the combined organic layers were washed with water and dried over Na_2SO_4. The solvents were removed under reduced pressure to afford the product **S3** as a brown oil (24.4 g, 136 mmol, 70% yield). The crude product was used in the next step without further purification.

^{1}H NMR (500 MHz, $CDCl_3$) δ 5.86 (d, *J* = 2.0 Hz, 1H), 2.78 – 2.66 (m, 2H), 2.54 – 2.42 (m, 4H), 2.20 – 2.07 (m, 3H), 1.71 (qt, *J* = 13.3, 4.4 Hz, 1H), 1.45 (s, 3H).

^{13}C NMR (126 MHz, $CDCl_3$) δ 211.2, 198.5, 165.9, 126.0, 50.8, 37.8, 33.8, 31.9, 29.9, 23.4, 23.1.

HRMS (ESI) m/z: $[M + H]^+$ Calculated for $C_{11}H_{15}O_2$ 179.1067; Found 179.1068.

A solution of **S3** (24.4 g, 137 mmol) in EtOH (50 mL) was cooled to -10 °C and a suspension of $NaBH_4$ (1.55 g, 41 mmol, 0.30 equiv) suspended in EtOH (20 mL) was added dropwise over 1 h. The reaction was subsequently allowed to warm up to 25 °C, stirred for 1 h and then quenched by addition of AcOH (7.8 mL, 137 mmol, 1 equiv) and the crude reaction mixture was concentrated under reduced pressure. The residue was suspended in EtOAc (50 mL) and, after filtration, was washed with water. After removal of the solvents, the residue was purified by vacuum distillation (180 °C at 5 mbar) to afford the product **S4** as a transparent oil that slowly solidified to a white wax upon standing at room temperature (20.2 g, 112 mmol, 82% yield).

^{1}H NMR (500 MHz, $CDCl_3$) δ 5.82 – 5.76 (m, 1H), 3.43 (dd, *J* = 11.6, 4.3 Hz, 1H), 2.51 – 2.27 (m, 3H), 2.26 – 2.13 (m, 2H), 1.93 – 1.77 (m, 4H), 1.70 (tdd, *J* = 12.8, 11.5, 4.3 Hz, 1H), 1.50 – 1.34 (m, 1H), 1.20 (d, *J* = 0.7 Hz, 3H).

^{13}**C NMR** (126 MHz, $CDCl_3$) δ 199.7, 168.5, 125.7, 78.4, 41.7, 34.4, 33.9, 32.2, 30.4, 23.3, 15.4.

HRMS (ESI) m/z: $[M + Na]^+$ Calculated for $C_{11}H_{16}NaO_2$ 203.1043; Found 203.1041.

Consistent with reported data:
Letort, A.; Long, D.-L.; Prunet, J. Study of Cascade Ring-Closing Metathesis Reactions En Route to an Advanced Intermediate of Taxol. *J. Org. Chem.* **2016**, *81*, 12318–12331. https://doi.org/10.1021/acs.joc.6b02264

(±)-(4aS,5S)-5-hydroxy-4a-methyloctahydronaphthalen-2(1H)-one ***5a***

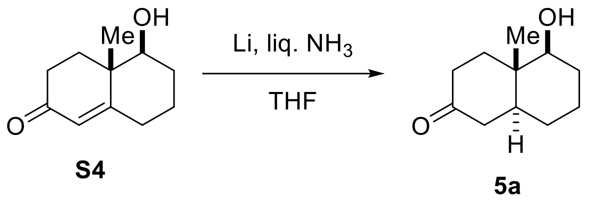

To freshly distilled NH_3 (approximately 50 mL, dried over Na) at -78 °C was added Li (104 mg, 15.0 mmol, 3 equiv) under a N_2 atmosphere. After stirring for 30 minutes at -78 °C, a solution of **S4** (901 mg, 5.00 mmol) in anhydrous THF (13 mL) was added dropwise. The reaction mixture was stirred for 1 hour at -78 °C and then warmed to room temperature and NH_4Cl was added until the blue colour faded. After evaporation of the NH_3, the residue was acidified by addition of 10% aqueous HCl solution until a pH of 4 was reached. The aqueous solution was extracted with Et_2O and the combined organic layers were dried over Na_2SO_4. After removal of the solvents under reduced pressure, the residue was purified by automated flash column chromatography (SiO_2, 20–60% EtOAc in hexanes) to afford the product **5a** as a colourless oil that slowly crystallized to a white wax upon standing (434 mg, 2.38 mmol, 48% yield).

^{1}**H NMR** (400 MHz, $CDCl_3$) δ 3.34 – 3.24 (m, 1H), 2.47 – 2.12 (m, 5H), 1.82 – 1.70 (m, 2H), 1.63 – 1.25 (m, 7H), 1.04 (d, *J* = 0.8 Hz, 3H).

^{13}**C NMR** (101 MHz, $CDCl_3$) δ 211.5, 78.9, 44.1, 44.0, 38.6, 38.0, 37.3, 30.6, 28.3, 24.4, 9.2.

HRMS (ESI) m/z: $[M + Na]^+$ Calculated for $C_{11}H_{18}NaO_2$ 205.1199; Found 205.1201.

Consistent with reported data:
Coates, R. M.; Muskopf, J. W.; Senter, P. A. Synthesis of Stereoisomeric 9a-Methylhydrodicyclopenta[a,d]Cyclooctan-1-Ones Related to the Ophiobolins and Ceroplastins via Annelative Ring Expansion of Hydrindene Precursors. *J. Org. Chem.*, **1985**, *50*, 3541–3557. https://doi.org/10.1021/jo00219a022

(±)-(4aS,8aS)-8a-methylhexahydronaphthalene-1,6(2H,5H)-dione ***6a***

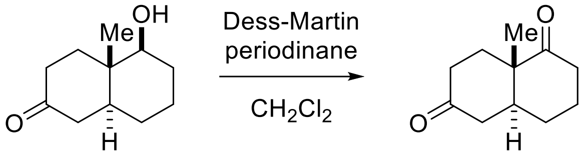

5a **6a**

A solution of **5a** (501 mg, 2.75 mmol) in CH_2Cl_2 (30 mL) was cooled to 0 °C and Dess-Martin periodinane (2.32 g, 5.47 mmol, 2 equiv) was added portionwise. The reaction mixture was stirred for 30 minutes at room temperature then quenched by addition of saturated aqueous $NaHCO_3$ solution. The layers were separated and the organic phase was washed with saturated aqueous $NaHCO_3$ solution and saturated aqueous $Na_2S_2O_3$ solution. The combined aqueous layers were extracted with CH_2Cl_2 and then the combined organic layers were washed with brine and dried over $MgSO_4$. After removal of the solvents under reduced pressure, the residue was purified by automated flash column chromatography (SiO_2, 30% EtOAc in hexanes) to afford the product **6a** as a transparent oil (294 mg, 59% yield).

^{1}H NMR (400 MHz, $CDCl_3$) δ 2.76 – 2.64 (m, 1H), 2.46 – 2.23 (m, 5H), 2.12 – 2.01 (m, 2H), 1.99 – 1.80 (m, 2H), 1.79 – 1.61 (m, 2H), 1.59 – 1.50 (m, 1H), 1.32 (d, *J* = 0.8 Hz, 3H).

^{13}C NMR (101 MHz, $CDCl_3$) δ 214.1, 209.7, 47.5, 45.2, 43.5, 37.6, 37.5, 32.4, 27.8, 26.1, 15.1.

HRMS (ESI) m/z: $[M + Na]^+$ Calculated for $C_{11}H_{16}NaO_2$ 203.1043; Found 203.1042.

Consistent with reported data:
Heathcock, C. H.; Ratcliffe, R.; Van, J. Synthesis of Hydroazulenes by Solvolytic Rearrangement of 9-Methyl-1-Declyl Tosylates. *J. Org. Chem.* **1972**, *37*, 1796–1807.
https://doi.org/10.1021/jo00976a029

*(±)-(4aR,8aS)-8a-methylhexahydronaphthalene-1,6(2H,5H)-dione **7a***

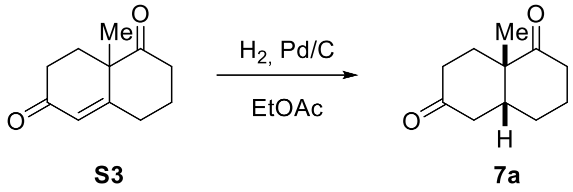

A suspension of **S3** (891 mg, 5.00 mmol) and 10% Pd/C (89 mg) in EtOAc (12 mL) was stirred under a H_2 atmosphere for 16 hours at room temperature. The reaction was then filtered through a plug of celite. After removal of the solvents under reduced pressure, the residue was purified by automated flash column chromatography (SiO_2, 0–50% EtOAc in hexanes) to afford **7a** as white crystalline needles (704 mg, 3.9 mmol, 78% yield).

^{1}H NMR (400 MHz, $CDCl_3$) δ 2.62 – 2.35 (m, 4H), 2.33 – 2.23 (m, 4H), 2.15 – 2.05 (m, 1H), 2.04 – 1.86 (m, 2H), 1.58 – 1.48 (m, 1H), 1.47 – 1.38 (m, 1H), 1.35 (s, 3H).

^{13}C NMR (101 MHz, $CDCl_3$) δ 214.2, 211.3, 48.7, 46.2, 43.9, 38.5, 37.6, 33.8, 26.8, 24.1, 23.1.

Consistent with reported data:

Kraus, G. A.; Gottschalk, P. Iodotrimethylsilane-Mediated Additions of Dienol Silyl Ethers to .Alpha.,.Beta.-Unsaturated Ketones. *J. Org. Chem.* **1984**, *49*, 1153–1154. https://doi.org/10.1021/jo00180a048

*(±)-(1S,4aS,8aS)-8a-methyl-6-oxodecahydronaphthalen-1-yl benzoate **8a***

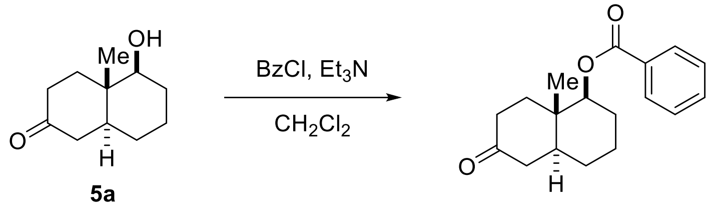

To a solution of **5a** (911 mg, 5.00 mmol) in anhydrous CH_2Cl_2 (50 mL) was added benzoyl chloride (1.16 mL, 10.0 mmol, 2 equiv) and Et_3N (3.48 mL, 25.0 mmol, 5 equiv). The reaction was flushed with N_2 and then heated at 40 °C for 48 h. After cooling to room temperature, pentane (50 mL) was added and the precipitate formed was removed by filtration. After removal of the solvents under reduced pressure, the residue was purified by automated flash column chromatography (SiO_2, 0–40% EtOAc in hexanes) to afford the product **8a** as a white amorphous powder (900 mg, 3.14 mmol, 63% yield).

^{1}H NMR (400 MHz, $CDCl_3$) δ 8.09 – 8.01 (m, 2H), 7.62 – 7.54 (m, 1H), 7.50 – 7.42 (m, 2H), 4.81 (dd, *J* = 11.6, 4.6 Hz, 1H), 2.47 – 2.18 (m, 4H), 2.06 – 1.91 (m, 2H), 1.85 (dtd, *J* = 13.2, 4.6, 2.0 Hz, 1H), 1.79 – 1.66 (m, 2H), 1.60 – 1.46 (m, 2H), 1.43 – 1.30 (m, 2H), 1.27 (d, *J* = 0.7 Hz, 3H).

^{13}C NMR (101 MHz, $CDCl_3$) δ 210.7, 166.1, 133.1, 130.6, 129.7, 128.5, 80.9, 43.9, 43.8, 38.0, 37.7, 37.0, 28.1, 27.1, 24.0, 10.5.

HRMS (ESI) m/z: $[M + Na]^+$ Calculated for $C_{18}H_{22}NaO_3$ 309.1461; Found 309.1465.

Compound previously unknown.

(±)-(4aS,5S,8aS)-5-((tert-butyldimethylsilyl)oxy)-4a-methyloctahydronaphthalen-2(1H)-one ***9a***

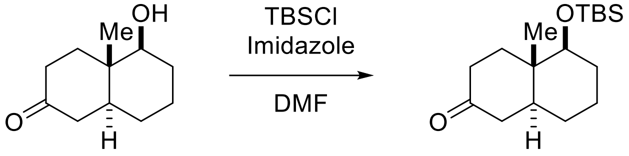

5a **9a**

To a solution of **5a** (520 mg, 2.85 mmol) in anhydrous DMF (10 mL) was added imidazole (486 mg, 7.13 mmol, 2.5 equiv) and TBSCl (645 mg, 4.28 mmol, 1.5 equiv) under a N_2 atmosphere. The reaction mixture was stirred at room temperature for 24 hours then diluted with Et_2O. The organic solution was washed with water and brine then dried over Na_2SO_4. After removal of the solvents under reduced pressure, the residue was purified by automated flash column chromatography (SiO_2, 5–30% EtOAc in hexanes) to afford the product **9a** as colourless white crystalline prisms (370 mg, 1.25 mmol, 44% yield).

^{1}H NMR (400 MHz, $CDCl_3$) δ 3.22 (dd, *J* = 10.9, 4.9 Hz, 1H), 2.41 (dddd, *J* = 15.6, 13.6, 6.6, 0.8 Hz, 1H), 2.35 – 2.21 (m, 2H), 2.19 – 2.09 (m, 2H), 1.78 – 1.66 (m, 1H), 1.65 – 1.44 (m, 3H), 1.38 – 1.21 (m, 4H), 1.01 (d, *J* = 0.8 Hz, 3H), 0.89 (s, 9H), 0.04 (s, 3H), 0.02 (s, 3H).

^{13}C NMR (101 MHz, $CDCl_3$) δ 212.0, 79.4, 44.3, 44.1, 39.2, 38.3, 37.9, 31.0, 28.4, 26.0, 24.3, 18.2, 9.5, -3.8, -4.7.

HRMS (ESI) m/z: $[M + Na]^+$ Calculated for $C_{17}H_{32}NaO_2Si$ 319.2064; Found 319.2063.

Compound previously unknown.

*(±)-(1S,4aS,8aS)-8a-methyl-6-oxodecahydronaphthalen-1-yl 3-methylbut-2-enoate **10a***

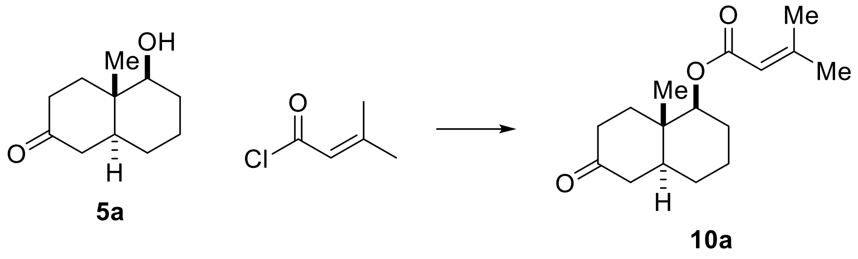

A solution of **5a** (1.00 g, 5.49 mmol) in anhydrous CH_2Cl_2 (50 mL) was cooled to 0 °C and 3-methylbut-2-enoyl chloride (0.92 mL, 8.230 mmol, 1.5 equiv) was added. To this solution Et_3N (3.8 mL, 27 mmol, 3 equiv) was added dropwise and the resulting reaction mixture was warmed to room temperature. After stirring at room temperature for 16 hours, pentane (50 mL) was added and the suspension formed was filtered. The filtrate was concentrated under reduced pressure and the residue was purified by automated flash column chromatography (SiO_2, 0–40% EtOAc in hexanes) to afford the product **10a** as white amorphous solid (224 mg, 0.85 mmol, 15% yield).

^{1}H NMR (400 MHz, $CDCl_3$) δ 5.66 (apparent p, *J* = 1.3 Hz, 1H), 4.57 (dd, *J* = 11.5, 4.4 Hz, 1H), 2.42 – 2.16 (m, 4H), 2.15 (d, *J* = 1.3 Hz, 3H), 1.93 (ddd, *J* = 13.4, 6.4, 2.4 Hz, 1H), 1.88 (d, *J* = 1.4 Hz, 3H), 1.79 (dddd, *J* = 13.2, 9.7, 4.6, 2.3 Hz, 2H), 1.70 – 1.52 (m, 2H), 1.52 – 1.36 (m, 2H), 1.36 – 1.20 (m, 2H), 1.11 (d, *J* = 0.7 Hz, 3H).

^{13}C NMR (101 MHz, $CDCl_3$) δ 210.9, 166.3, 156.9, 116.3, 79.2, 43.9, 43.8, 37.7, 37.6, 36.8, 28.1, 27.5, 27.1, 24.0, 20.4, 10.3.

HRMS (ESI) m/z: $[M + Na]^+$ Calculated for $C_{16}H_{24}NaO_3$ 287.1618; Found 287.1619.

Compound previously unknown.

(±)-(1S,4aS,8aS)-8a-methyl-6-oxodecahydronaphthalen-1-yl (tert-butoxycarbonyl)glycinate ***11a***

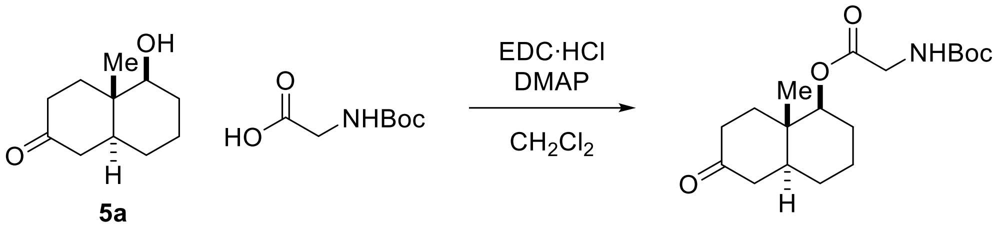

11a

A solution of **5a** (290 mg, 1.59 mmol) and Boc-Gly-OH (280 mg, 1.60 mmol, 1 equiv) in anhydrous CH_2Cl_2 (20 mL) was cooled to 0 °C and EDC hydrochloride (408 mg, 2.13 mmol, 1.3 equiv) and DMAP (100 mg, 0.82 mmol, 0.5 equiv) were added. The reaction mixture was stirred at room temperature for 16 hours and then concentrated under reduced pressure. The residue was dissolved in EtOAc and water and then the layers were separated. The organic layer was washed with brine then dried over Na_2SO_4. After removal of the solvents under reduced pressure, the residue was purified by automated flash column chromatography (SiO_2, 0–40% EtOAc in hexanes) to afford the product **11a** as a colourless oil (507 mg, 1.49 mmol, 94% yield).

^{1}H NMR (400 MHz, $CDCl_3$) δ 4.98 (s, 1H), 4.62 (dd, *J* = 11.6, 4.3 Hz, 1H), 3.99 – 3.80 (m, 2H), 2.44 – 2.14 (m, 4H), 1.93 (ddd, *J* = 13.4, 6.2, 2.6 Hz, 1H), 1.80 (dd, *J* = 11.8, 2.8 Hz, 2H), 1.70 – 1.57 (m, 2H), 1.45 (s, 10H), 1.40 – 1.19 (m, 3H), 1.11 (s, 3H).

^{13}C NMR (101 MHz, $CDCl_3$) δ 210.5, 170.0, 155.8, 81.4, 80.1, 43.8, 43.7, 42.7, 37.7, 37.6, 36.7, 28.4, 27.9, 26.9, 23.8, 10.1.

HRMS (ESI) m/z: $[M + Na]^+$ Calculated for $C_{18}H_{29}NaNO_5$ 362.1938; Found 362.1937.

Compound previously unknown.

(±)-(1S,4aS,8aS)-8a-methyl-6-oxodecahydronaphthalen-1-yl 2-(1,3-dioxoisoindolin-2-yl)acetate ***12a***

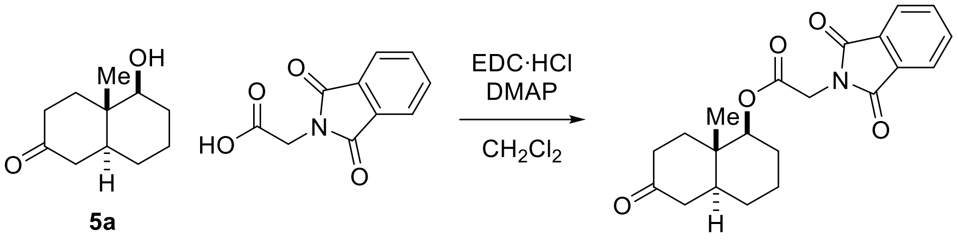

12a

A solution of **5a** (500 mg, 2.74 mmol), *N*-Phthaloylglycine (619 mg, 3.02 mmol, 1.1 equiv) in anhydrous CH_2Cl_2 (20 mL) was cooled to 0 °C and EDC hydrochloride (684 mg, 3.57 mmol, 1.3 equiv) and DMAP (67 mg, 0.55 mmol, 0.2 equiv) were added. The reaction mixture was stirred at room temperature for 16 hours. The solvents were then removed under reduced pressure, the residue was dissolved in EtOAc and washed with water and saturated aqueous NaCl solution, then dried over Na_2SO_4. After removal of the solvents under reduced pressure, the residue was purified by automated flash column chromatography (SiO_2, 5–30% EtOAc in hexanes) to afford the product **12a** as a white amorphous powder (710 mg, 1.92 mmol, 70% yield).

^{1}H NMR (400 MHz, $CDCl_3$) δ 7.89 (dd, J = 5.5, 3.1 Hz, 2H), 7.79 – 7.72 (m, 2H), 4.63 (dd, *J* = 11.6, 4.5 Hz, 1H), 4.44 (d, *J* = 2.2 Hz, 2H), 2.39 – 2.31 (m, 2H), 2.27 – 2.12 (m, 2H), 1.99 – 1.90 (m, 1H), 1.80 (ddtd, *J* = 14.4, 8.2, 4.3, 2.5 Hz, 2H), 1.68 – 1.54 (m, 2H), 1.51 – 1.16 (m, 4H), 1.01 (d, *J* = 0.7 Hz, 3H).

^{13}C NMR (101 MHz, $CDCl_3$) δ 210.4, 167.6, 166.9, 134.5, 132.1, 123.8, 82.1, 43.8, 43.7, 39.2, 37.7, 37.7, 36.7, 27.9, 26.9, 23.8, 10.1.

HRMS (ESI) m/z: $[M + H]^+$ Calculated for $C_{21}H_{24}NO_5$ 370.1649; Found 370.1654.

Compound previously unknown.

*(±)-(1S,7aS)-1-hydroxy-7a-methyl-1,2,3,6,7,7a-hexahydro-5H-inden-5-one **S7***

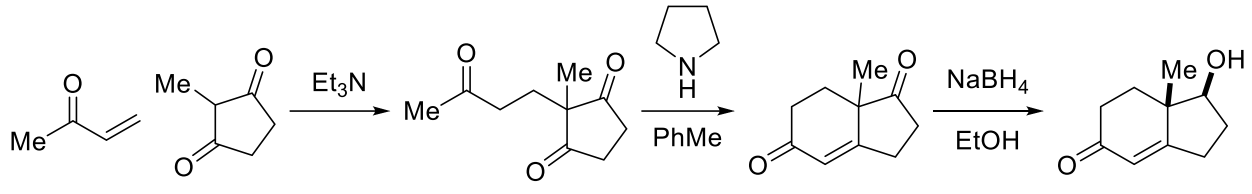

A solution of 2-methyl-cyclopentane-1,3-dione (25 g, 200 mmol) and Et_3N (0.31 mL, 2.22 mmol, 0.01 equiv) was cooled to 0 °C under a N_2 atmosphere and freshly distilled methyl vinyl ketone (23 mL, 279 mmol, 1.2 equiv) was added. The solution was stirred for 16 hours at 25 °C then diluted with EtOAc and activated charcoal (5 g) was added. The suspension was then heated at 45 °C for 30 minutes. After cooling to room temperature, the suspension was filtered and the filtrate was concentrated under reduced pressure to afford the product **S5** as a pale yellow oil (36.1 g, 198 mmol, 99% yield). The crude product was used in the next step without further purification.

^{1}H NMR (400 MHz, $CDCl_3$) δ 2.91 – 2.65 (m, 4H), 2.44 (t, *J* = 7.2 Hz, 2H), 2.08 (s, 3H), 1.87 (t, *J* = 7.2 Hz, 2H), 1.09 (s, 3H).

^{13}C NMR (101 MHz, $CDCl_3$) δ 215.9, 208.0, 55.2, 37.5, 34.8, 30.1, 27.9, 19.2.

HRMS (ESI) m/z: $[M + Na]^+$ Calculated for $C_{10}H_{14}NaO_3$ 205.0835; Found 205.0835.

A solution of **S5** (36.1 g, 198 mmol) and pyrrolidine (1.8 mL, 22 mmol, 0.11 equiv) in PhMe (100 mL) was heated at reflux with a Dean-Stark apparatus for 6 h. After cooling to room temperature, the organic solution was washed with 10% aqueous HCl solution and water. The combined aqueous layers were extracted with Et_2O and then the combined organic layers were washed with water and dried over Na_2SO_4. The solvents were removed under reduced pressure to afford the product **S6** as a brown oil. The crude product was used in the next step without further purification.

^{1}H NMR (400 MHz, $CDCl_3$) δ 6.00 – 5.95 (m, 1H), 3.03 – 2.88 (m, 1H), 2.86 – 2.69 (m, 2H), 2.60 – 2.36 (m, 3H), 2.11 (ddd, *J* = 13.6, 5.1, 2.3 Hz, 1H), 1.85 (td, *J* = 13.5, 5.7 Hz, 1H), 1.32 (s, 3H).

^{13}C NMR (101 MHz, $CDCl_3$) δ 216.7, 198.3, 169.9, 124.1, 48.9, 36.0, 33.1, 29.4, 27.0, 20.7.

HRMS (ESI) m/z: $[M + H]^+$ Calculated for $C_{10}H_{13}O_2$ 165.0910; Found 165.0912.

A solution of **S6** in EtOH (50 mL) was cooled to -10 °C and a suspension of $NaBH_4$ (2.49 g, 65.8 mmol, 0.33 equiv) suspended in EtOH (20 mL) was added dropwise over 1 h. The reaction was subsequently allowed to warm up to 25 °C, stirred for 1 h and then quenched by addition of AcOH (13.2 mL, 231 mmol, 1.2 equiv) and the crude reaction mixture was concentrated under reduced pressure. The residue was suspended in EtOAc (50 mL) and, after filtration, was washed with water. After removal of the solvents, the residue was purified by vacuum distillation (180 °C at 5 mbar) to afford the product **S7** as a transparent oil that slowly solidified to a white wax upon standing at room temperature (25.0 g, 150 mmol, 76% yield over two steps).

^{1}H NMR (400 MHz, $CDCl_3$) δ 5.85 – 5.74 (m, 1H), 3.83 (t, *J* = 9.1 Hz, 1H), 2.75 – 2.63 (m, 1H), 2.56 – 2.31 (m, 4H), 2.17 – 2.05 (m, 2H), 1.87 – 1.70 (m, 2H), 1.13 (s, 3H).

^{13}**C NMR** (101 MHz, $CDCl_3$) δ 199.5, 175.5, 123.4, 80.6, 45.2, 34.1, 33.3, 29.1, 26.5, 15.1.

HRMS (ESI) m/z: $[M + H]^+$ Calculated for $C_{10}H_{15}O_2$ 167.1067; Found 167.1067.

Consistent with reported data:
Enev, V. S.; Petrov, O. S.; Neh, H.; Nickisch, K. Studies towards a Total Synthesis of the Antiprogestine Onapristone. *Tetrahedron* **1997**, *53*, 13709–13718.
https://doi.org/10.1016/s0040-4020(97)00934-4

(±)-(1S,3aS,7aS)-1-hydroxy-7a-methyloctahydro-5H-inden-5-one ***13a***

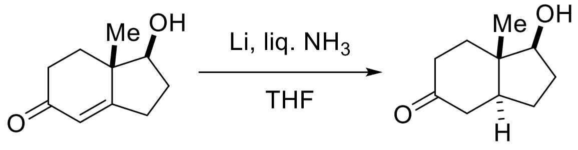

S7 **13a**

To freshly distilled NH_3 (approximately 300 mL, dried over Na) at -78 °C under a N_2 atmosphere was added Li (3.13 g, 451 mmol, 3 equiv) under a N_2 atmosphere. After stirring for 30 minutes at -78 °C, a solution of **S7** (25 g, 150 mmol) in anhydrous THF (50 mL) was added dropwise. The reaction mixture was stirred for 1 hour at -78 °C and then warmed to room temperature and NH_4Cl was added until the blue colour faded. After evaporation of the NH_3, the residue was acidified by addition of 10% aqueous HCl solution until a pH of 4 was reached. The aqueous solution was extracted with Et_2O and the combined organic layers were dried over Na_2SO_4. After removal of the solvents under reduced pressure, the residue was purified by automated flash column chromatography (SiO_2, 10–50% EtOAc in hexanes) to afford the product **13a** as a colourless oil that slowly crystallized to a white wax upon standing (5.3 g, 32 mmol, 21% yield).

^{1}H NMR (400 MHz, $CDCl_3$) δ 3.85 (dd, *J* = 6.2, 4.3 Hz, 1H), 2.51 – 2.36 (m, 2H), 2.31 – 2.18 (m, 3H), 2.13 (dddd, *J* = 14.2, 9.7, 6.3, 3.4 Hz, 1H), 1.99 – 1.88 (m, 1H), 1.73 – 1.52 (m, 4H), 1.31 – 1.20 (m, 1H), 1.18 (s, 3H).

^{13}C NMR (101 MHz, $CDCl_3$) δ 213.0, 80.1, 43.9, 43.3, 42.1, 37.0, 32.2, 32.2, 28.5, 19.5.

HRMS (ESI) m/z: $[M + Na]^+$ Calculated for $C_{10}H_{16}NaO_2$ 191.1043; Found 191.1041.

Consistent with reported data:
Hajos, Z. G.; Parrish, D. R. Stereocontrolled Total Synthesis of 19-Norsteroids. *J. Org. Chem.* **1973**, *38*, 3244–3249.
https://doi.org/10.1021/jo00959a003

(±)-(3aS,7aS)-7a-methylhexahydro-1H-indene-1,5(4H)-dione ***14a***

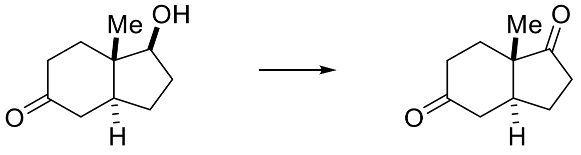

13a **14a**

A solution of **13a** (1.00 g, 5.94 mmol) in CH_2Cl_2 (20 mL) was cooled to 0 °C and Dess-Martin periodinane (5.04 g, 11.9 mmol, 2 equiv) was added portionwise. The reaction mixture was stirred for 16 hours at room temperature then quenched by addition of saturated aqueous $NaHCO_3$ solution. The layers were separated and the organic phase was washed with saturated aqueous $NaHCO_3$ solution and saturated aqueous $Na_2S_2O_3$ solution. The combined aqueous layers were extracted with CH_2Cl_2 and then the combined organic layers were washed with brine and dried over $MgSO_4$. After removal of the solvents under reduced pressure, the residue was purified by automated flash column chromatography (SiO_2, 5–30% EtOAc in hexanes) to afford the product **14a** as transparent prisms (820 mg, 83% yield).

^{1}H NMR (400 MHz, $CDCl_3$) δ 2.59 (ddd, *J* = 14.9, 6.3, 1.1 Hz, 1H), 2.50 – 2.18 (m, 6H), 2.12 (dddd, *J* = 13.5, 9.1, 6.8, 4.4 Hz, 1H), 2.02 (ddd, *J* = 14.1, 10.6, 4.9 Hz, 1H), 1.70 – 1.54 (m, 2H), 1.24 (s, 3H).

^{13}C NMR (101 MHz, $CDCl_3$) δ 220.4, 210.8, 47.4, 44.8, 42.0, 37.2, 35.3, 30.0, 25.3, 20.8.

HRMS (ESI) m/z: $[M + Na]^+$ Calculated for $C_{10}H_{14}NaO_2$ 189.0886; Found 189.0890.

Consistent with reported data:

Hajos, Z. G.; Parrish, D. R. Stereocontrolled Total Synthesis of 19-Norsteroids. *J. Org. Chem*. **1973**, *38*, 3244–3249. https://doi.org/10.1021/jo00959a003

Androstanedione ***17a***

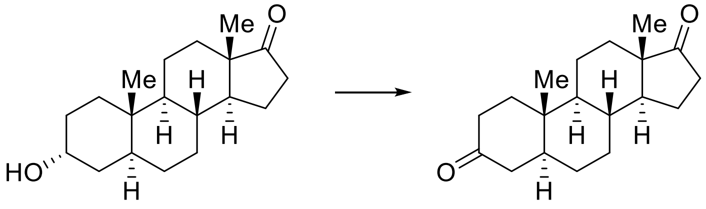

To a suspension of Androsterone (1.45 g, 5.00 mmol) and celite (3.2 g) in CH_2Cl_2 (30 mL) was added PCC (1.62 g, 7.50 mmol, 1.5 equiv) at 0 °C. The reaction was allowed to warm to room temperature, stirred for 16 hours and then filtered through a plug of celite and silica. After removal of the solvents under reduced pressure, the residue was purified by automated flash column chromatography (SiO_2, 0–30% EtOAc in hexanes) to afford the product **17a** as a white amorphous powder (yield = 1.27 g, 4.43 mmol, 88% yield).

^{1}H NMR (500 MHz, $CDCl_3$) δ 2.50 – 2.18 (m, 4H), 2.14 – 1.87 (m, 4H), 1.81 (ddt, *J* = 12.8, 6.4, 3.4 Hz, 2H), 1.73 – 1.62 (m, 1H), 1.62 – 1.43 (m, 3H), 1.43 – 1.16 (m, 6H), 1.02 (m, 4H), 0.86 (s, 3H), 0.78 (ddd, *J* = 12.2, 10.4, 4.1 Hz, 1H).

^{13}C NMR (126 MHz, $CDCl_3$) δ 221.0, 211.6, 54.0, 51.3, 47.8, 46.7, 44.7, 38.5, 38.2, 35.9, 35.0, 31.6, 30.6, 28.7, 21.9, 20.8, 13.9, 11.5.

R_f (25% EtOAc in hexanes) = 0.19.

Consistent with reported data:

Hamada, S.; Sugimoto, K.; Elboray, E. E.; Kawabata, T.; Furuta, T. Chemoselective Oxidation of p-Methoxybenzyl Ethers by an Electronically Tuned Nitroxyl Radical Catalyst. *Org. Lett*. **2020**, *22*, 5486–5490. https://doi.org/10.1021/acs.orglett.0c01839

6. Isomerization of cyclic ketones and characterization of products

General procedure for the isomerisation of cycloalkanones

To an oven-dried, screw-cap vial was added the indicated substrate (1.00 mmol), S_8 (6.4 mg, 0.025 mmol, 2.5 mol%, 20 mol% S), *i*-PrOH (2 mL), H_2O (90 µL, 5 mmol, 5 equiv) and pyrrolidine (16 µL, 0.20 mmol, 20 mol%). The vial was flushed with N_2, sealed and then heated at 80 °C for 24 hours. After cooling to room temperature, the reaction mixture was diluted with EtOAc and filtered through a plug of silica, eluting with EtOAc and CH_2Cl_2. The solvents were removed under reduced pressure and the residue was purified as described below.

(±)-(4aS,5S,8aS)-5-hydroxy-4a-methyloctahydronaphthalen-2(1H)-one ***5b***

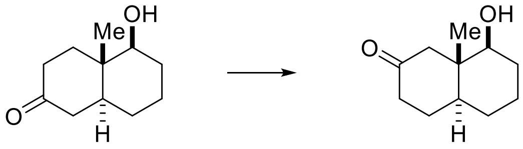

5a **5b**

Prepared according to the general procedure with **5a** (198 mg, 1.09 mmol). The crude reaction mixture was purified by automated flash column chromatography (SiO_2, 15–50% EtOAc in hexanes) to give a mixture of regioisomers **5a** and **5b** (163 mg, 82% yield). The regioisomers were then separated by preparative reverse-phase HPLC (Agilent ZORBAX Eclipse XDB-C18 Prep HT 21.2 × 250 mm column, flow rate = 20 mL/min, gradient = 10–90% MeCN in H_2O (containing 0.1% TFA) over 20 minutes) to afford the product **5b** as a colourless oil (63 mg, 0.35 mmol, 32% yield).

^{1}H NMR (400 MHz, $CDCl_3$) δ 3.50 – 3.30 (m, 1H), 2.57 (dd, *J* = 13.4, 2.0 Hz, 1H), 2.42 – 2.24 (m, 2H), 2.08 (dq, *J* = 13.5, 1.1 Hz, 1H), 1.85 – 1.52 (m, 6H), 1.48 – 1.33 (m, 3H), 1.23 – 1.11 (m, 1H), 0.76 (d, *J* = 1.1 Hz, 3H).

^{13}C NMR (101 MHz, $CDCl_3$) δ 211.8, 78.3, 52.7, 43.1, 42.6, 41.5, 30.1, 28.7, 27.3, 24.1, 10.4.

HRMS (ESI) m/z: $[M + Na]^+$ Calculated for $C_{11}H_{18}NaO_2$ 205.1199; Found 205.1204.

Consistent with reported data:

Orru, R. V. A.; Wijnberg, J. B. P. A.; Bouwman, C. T.; de Groot, A. Rearrangement vs Homofragmentation: Chemical Consequences of Different .Sigma.-Relays on the Heterolysis of Sulfonate Esters Induced by Through-Bond Interactions. *J. Org. Chem.* **1994**, *59*, 374–382. https://doi.org/10.1021/jo00081a016

*(±)-(4aS,8aS)-8a-methylhexahydronaphthalene-1,6(2H,5H)-dione **6b***

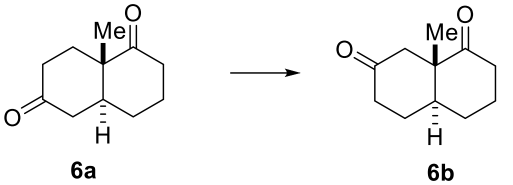

Prepared according to the general procedure with **6a** (180 mg, 1.00 mmol). The crude reaction mixture was purified by automated flash column chromatography (SiO_2, 5–40% EtOAc in hexanes) to give a mixture of regioisomers **6a** and **6b** (142 mg, 79% yield). The regioisomers were then separated by preparative reverse-phase HPLC (Agilent ZORBAX Eclipse XDB-C18 Prep HT 21.2 × 250 mm column, flow rate = 20 mL/min, gradient = 30–35% MeCN in H_2O (containing 0.1% TFA) over 20 minutes) to afford the product **6b** as a colourless oil (79 mg, 0.44 mmol, 44% yield).

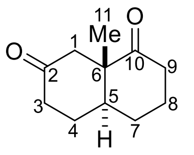

6b

^{1}H NMR (600 MHz, $CDCl_3$) δ 2.59 (apparent td, *J* = 13.9, 6.7 Hz, 1H, H9), 2.51 (apparent dp, *J* = 14.3, 1.1 Hz, 1H, H1), 2.42 – 2.35 (m, 2H, H1, H3), 2.32 – 2.24 (m, 2H, H3, H9), 2.14 – 2.08 (m, 1H, H8), 1.99 – 1.89 (m, 2H, H5, H4), 1.80 – 1.62 (m, 4H, H4, H7, H7, H8), 1.05 (d, *J* = 1.0 Hz, 3H, H11).

^{13}C NMR (150 MHz, $CDCl_3$) δ 213.3 (C10), 211.4 (C2), 51.2 (C6), 48.9 (C1), 44.9 (C5), 41.2 (C3), 36.8 (C9), 28.1 (C4), 27.1 (C7), 26.2 (C8), 16.6 (C11).

HRMS (ESI) m/z: $[M + Na]^+$ Calculated for $C_{11}H_{16}NaO_2$ 203.1043; Found 203.1045.

Compound previously unknown.

(±)-(4aR,8aS)-8a-methyloctahydronaphthalene-1,7-dione ***7b***

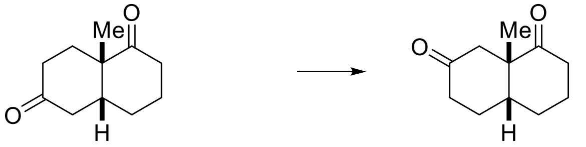

7a **7b**

Prepared according to the general procedure with **7a** (180 mg, 1.00 mmol). The crude reaction mixture was purified by automated flash column chromatography (SiO_2, 0–30% EtOAc in hexanes) to afford the product **7b** as a white amorphous powder (47 mg, 0.26 mmol, 26% yield).

^{1}H NMR (400 MHz, $CDCl_3$) δ 2.85 (dd, *J* = 14.5, 1.7 Hz, 1H), 2.54 (dddd, *J* = 14.7, 8.9, 6.0, 0.8 Hz, 1H), 2.48 – 2.33 (m, 2H), 2.28 (dddd, *J* = 14.9, 9.0, 5.7, 1.5 Hz, 1H), 2.15 – 1.95 (m, 3H), 1.95 – 1.70 (m, 5H), 1.27 (s, 3H).

^{13}C NMR (101 MHz, $CDCl_3$) δ 212.9, 208.7, 52.9, 47.5, 43.6, 39.4, 37.2, 28.2, 26.0, 24.1, 23.8.

Consistent with reported data:

Ando, S.; Minor, K. P.; Overman, L. E. Ring-Enlarging Cyclohexane Annulations. *J. Org. Chem.* **1997**, *62*, 6379–6387. https://doi.org/10.1021/jo970399i

*(±)-(1S,4aS,8aS)-8a-methyl-7-oxodecahydronaphthalen-1-yl benzoate **8b***

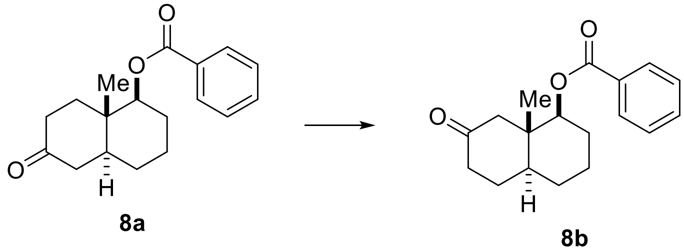

Prepared according to the general procedure with **8a** (286 mg, 1.00 mmol). The crude reaction mixture was purified by automated flash column chromatography (SiO_2, 0–35% EtOAc in hexanes) to give a mixture of regioisomers **8a** and **8b** (218 mg, 76% yield). The regioisomers were then separated by preparative reverse-phase HPLC (Agilent ZORBAX Eclipse XDB-C18 Prep HT 21.2 × 250 mm column, flow rate = 20 mL/min, gradient = 50–70% MeCN in H_2O (containing 0.1% TFA) over 20 minutes) to afford the product **8b** as a white amorphous powder (100 mg, 0.35 mmol, 35% yield).

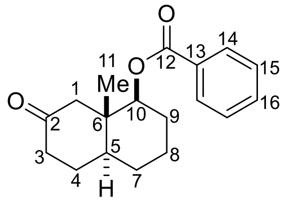

8b

^{1}H NMR (600 MHz, $CDCl_3$) δ 8.04 – 7.96 (m, 2H, H14), 7.58 – 7.54 (m, 1H, H16), 7.47 – 7.42 (m, 2H, H15), 4.92 (dd, *J* = 11.2, 4.5 Hz, 1H, H10), 2.43 – 2.31 (m, 3H, H1, H3, H3), 2.21 – 2.16 (m, 1H, H1), 1.96 – 1.91 (m, 1H, H9), 1.91 – 1.83 (m, 2H, H4 & H8), 1.79 (tt, *J* = 12.2, 3.4 Hz, 1H, H5), 1.72 – 1.65 (m, 1H, H4), 1.65 – 1.50 (m, 3H, H7, H8, H9), 1.32 – 1.24 (m, 1H, H7), 1.00 (d, *J* = 1.0 Hz, 3H, H11).

^{13}C NMR (150 MHz, $CDCl_3$) δ 210.8 (C2), 166.2 (C12), 133.2 (C16), 130.4 (C13), 129.7 (C14), 128.5 (C15), 80.2 (C10), 52.6 (C1), 42.9 (C5), 42.3 (C6), 41.4 (C3), 28.5 (C4), 27.2 (C7), 26.6 (C9), 23.9 (C8), 11.9 (C11).

HRMS (ESI) m/z: $[M + Na]^+$ Calculated for $C_{18}H_{22}NaO_3$ 309.1461; Found 309.1466.

Compound previously unknown.

(±)-(4aS,8S,8aS)-8-((tert-butyldimethylsilyl)oxy)-8a-methyloctahydronaphthalen-2(1H)-one ***9b***

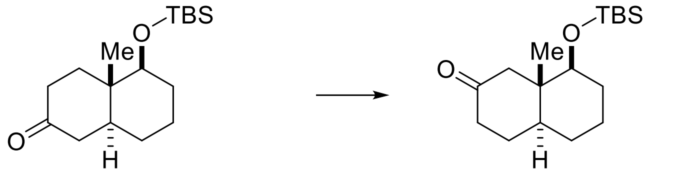

9a **9b**

Prepared according to the general procedure with **9a** (297 mg, 1.00 mmol). The crude reaction mixture was purified by automated flash column chromatography (SiO_2, 0–10% EtOAc in hexanes) to give a mixture of regioisomers **9a** and **9b** (232 mg, 78% yield). The regioisomers were then separated by preparative reverse-phase HPLC (Agilent ZORBAX Eclipse XDB-C18 Prep HT 21.2 × 250 mm column, flow rate = 20 mL/min, gradient = 60–70% MeCN in H_2O over 20 minutes) to afford the product **9b** as a white amorphous powder (95 mg, 0.32 mmol, 32% yield).

^{1}H NMR (400 MHz, $CDCl_3$) δ 3.35 (dd, *J* = 10.8, 4.3 Hz, 1H), 2.51 (dd, *J* = 13.5, 2.2 Hz, 1H), 2.41 – 2.23 (m, 2H), 1.95 (dt, *J* = 13.6, 1.1 Hz, 1H), 1.83 – 1.68 (m, 2H), 1.68 – 1.50 (m, 3H), 1.47 – 1.12 (m, 4H), 0.87 (s, 9H), 0.75 (d, *J* = 1.0 Hz, 3H), 0.03 (s, 3H), 0.02 (s, 3H).

^{13}C NMR (101 MHz, $CDCl_3$) δ 212.1, 78.9, 53.3, 43.5, 42.7, 41.5, 30.4, 28.7, 27.3, 25.9, 23.9, 18.0, 10.6, -4.0, -4.8.

HRMS (ESI) m/z: $[M + Na]^+$ Calculated for $C_{17}H_{32}NaO_2Si$ 319.2064; Found 319.2069.

Consistent with literature data:

Orru, R. V. A.; Wijnberg, J. B. P. A.; Bouwman, C. T.; de Groot, A. Rearrangement vs Homofragmentation: Chemical Consequences of Different .Sigma.-Relays on the Heterolysis of Sulfonate Esters Induced by Through-Bond Interactions. *J. Org. Chem.* **1994**, *59*, 374–382. https://doi.org/10.1021/jo00081a016

*(±)-(1S,4aS,8aS)-8a-methyl-7-oxodecahydronaphthalen-1-yl 3-methylbut-2-enoate **10b***

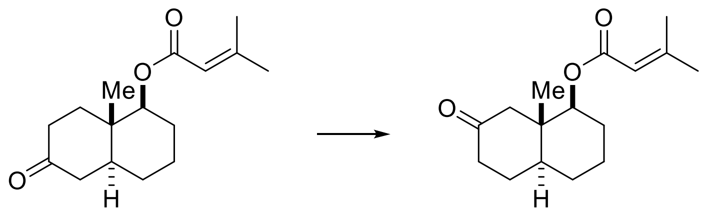

10a **10b**

Prepared according to the general procedure with **10a** (264 mg, 1.00 mmol). The crude reaction mixture was purified by automated flash column chromatography (SiO_2, 0–40% EtOAc in hexanes) to give a mixture of regioisomers **10a** and **10b** (159 mg, 60% yield). The regioisomers were then separated by preparative reverse-phase HPLC (Agilent ZORBAX Eclipse XDB-C18 Prep HT 21.2 × 250 mm column, flow rate = 20 mL/min, gradient = 50–70% MeCN in H_2O (containing 0.1% TFA) over 20 minutes), to afford the product **10b** as a white amorphous powder (17 mg, 0.06 mmol, 6% yield).

^{1}H NMR (400 MHz, $CDCl_3$) δ 5.64 (hept, *J* = 1.3 Hz, 1H), 4.75 – 4.66 (m, 1H), 2.36 – 2.26 (m, 3H), 2.14 (d, *J* = 1.3 Hz, 3H), 2.10 (dt, *J* = 13.6, 1.1 Hz, 1H), 1.88 (d, *J* = 1.3 Hz, 3H), 1.85 – 1.74 (m, 3H), 1.73 – 1.59 (m, 2H), 1.48 (tdd, *J* = 8.3, 3.6, 2.0 Hz, 3H), 1.27 – 1.18 (m, 1H), 0.85 (d, *J* = 1.1 Hz, 3H).

^{13}C NMR (101 MHz, $CDCl_3$) δ 211.1, 166.4, 157.2, 116.2, 78.4, 52.5, 42.8, 42.0, 41.4, 28.5, 27.6, 27.2, 26.7, 23.9, 20.4, 11.7.

HRMS (ESI) m/z: $[M + Na]^+$ calculated for $C_{16}H_{24}NaO_3$ 287.1618; Found 287.1621.

Compound previously unknown.

(±)-(1S,4aS,8aS)-8a-methyl-7-oxodecahydronaphthalen-1-yl (tert-butoxycarbonyl)glycinate ***11b***

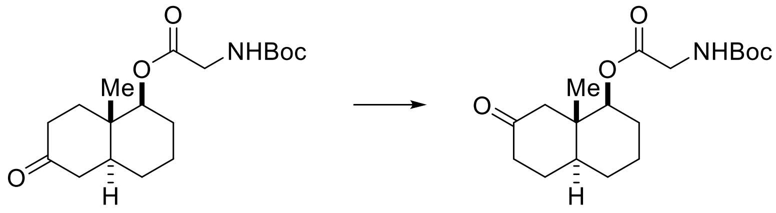

11a **11b**

Prepared according to the general procedure with **11a** (339 mg, 1.00 mmol). The crude reaction mixture was purified by automated flash column chromatography (SiO_2, 0–50% EtOAc in hexanes) to give a mixture of regioisomers **11a** and **11b** (252 mg, 74% yield). The regioisomers were then separated by preparative reverse-phase HPLC (Agilent ZORBAX Eclipse XDB-C18 Prep HT 21.2 × 250 mm column, flow rate = 20 mL/min, gradient = 50–70% MeCN in H_2O over 20 minutes) to afford the product **11b** as a white amorphous powder (90 mg, 0.27 mmol, 27% yield).

^{1}H NMR (400 MHz, $CDCl_3$) δ 5.06 – 4.95 (m, 1H), 4.77 – 4.65 (m, 1H), 3.98 – 3.74 (m, 2H), 2.39 – 2.20 (m, 3H), 2.07 (dt, *J* = 13.6, 1.0 Hz, 1H), 1.88 – 1.73 (m, 3H), 1.73 – 1.54 (m, 2H), 1.43 (m, 12H), 1.30 – 1.12 (m, 1H), 0.82 (d, *J* = 1.0 Hz, 3H).

^{13}C NMR (101 MHz, $CDCl_3$) δ 210.4, 170.1, 155.7, 80.7, 80.1, 52.3, 42.7, 42.6, 41.8, 41.3, 28.4, 28.3, 27.0, 26.4, 23.7, 11.5.

HRMS (ESI) m/z: $[M + NH_4]^+$ Calculated for $C_{18}H_{33}N_2O_5$ 357.2384; Found 357.2384.

Compound previously unknown.

*(±)-(1S,4aS,8aS)-8a-methyl-7-oxodecahydronaphthalen-1-yl 2-(1,3-dioxoisoindolin-2-yl)acetate **12b***

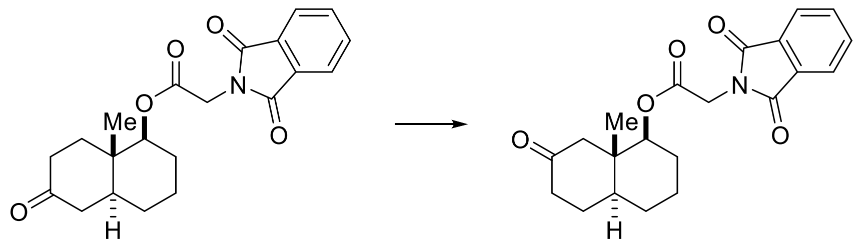

12a **12b**

Prepared according to the general procedure with **12a** (369 mg, 1.00 mmol). The crude reaction mixture was purified by automated flash column chromatography (SiO_2, 5–40% EtOAc in hexanes) to give a mixture of regioisomers **12a** and **12b** (267 mg, 72% yield). The regioisomers were then separated by preparative reverse-phase HPLC (Agilent ZORBAX Eclipse XDB-C18 Prep HT 21.2 × 250 mm column, flow rate = 20 mL/min, gradient = 50–55% MeCN in H_2O (containing 0.1% TFA) over 20 minutes) to afford the product **12b** as a white amorphous powder (20 mg, 0.05 mmol, 5% yield).

^{1}H NMR (400 MHz, $CDCl_3$) δ 7.87 (dd, *J* = 5.5, 3.0 Hz, 2H), 7.78 – 7.69 (m, 2H), 4.76 – 4.66 (m, 1H), 4.40 (s, 2H), 2.39 – 2.23 (m, 3H), 2.09 (dt, *J* = 13.4, 1.1 Hz, 1H), 1.87 – 1.74 (m, 3H), 1.72 – 1.36 (m, 5H), 1.22 – 1.10 (m, 1H), 0.76 (d, *J* = 1.0 Hz, 3H).

^{13}C NMR (101 MHz, $CDCl_3$) δ 210.2, 167.5, 167.0, 134.4, 132.1, 123.7, 81.6, 52.2, 42.7, 41.8, 41.3, 39.1, 28.4, 27.0, 26.3, 23.6, 11.5.

HRMS (ESI) m/z: $[M + NH_4]^+$ Calculated for $C_{21}H_{27}N_2O_5$ 387.1914; Found 387.1916.

Compound previously unknown.

(±)-(3S,3aS,7aS)-3-hydroxy-3a-methyloctahydro-5H-inden-5-one ***13b****, (±)-(1S,3aR,7aS)-1-hydroxy-7a-methyloctahydro-4H-inden-4-one* ***13c***

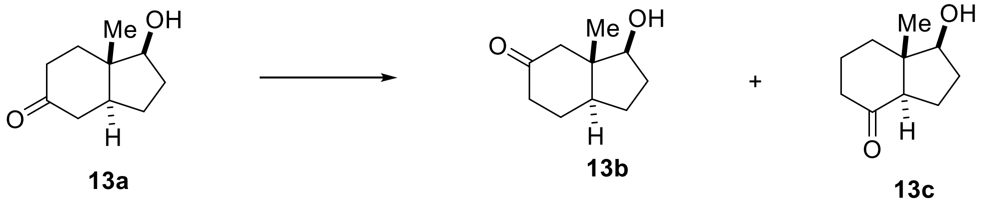

Prepared according to the general procedure with **13a** (168 mg, 1.00 mmol). The crude reaction mixture was purified by preparative reverse-phase HPLC (Agilent ZORBAX Eclipse XDB-C18 Prep HT 21.2 × 250 mm column, flow rate = 20 mL/min, gradient = 10–20% MeCN in H_2O (containing 0.1% TFA) over 20 minutes), to afford a fraction containing product regioisomers **13b** and **13c**. The regioisomers were then separated by automated flash column chromatography (SiO_2, 5–40% EtOAc in hexanes) to afford the product **13b** (32 mg, 0.19 mmol, 19% yield) and **13c** (15 mg, 0.09 mmol, 9% yield) as colourless oils.

13b:

^{1}H NMR (400 MHz, $CDCl_3$) δ 3.75 (t, *J* = 5.6 Hz, 1H), 2.43 – 2.32 (m, 1H), 2.28 – 2.10 (m, 3H), 2.09 – 1.90 (m, 4H), 1.79 (dq, *J* = 14.0, 6.0 Hz, 1H), 1.70 – 1.62 (m, 2H), 1.59 – 1.48 (m, 1H), 1.00 (d, *J* = 0.5 Hz, 3H).

^{13}C NMR (101 MHz, $CDCl_3$) δ 213.1, 80.9, 48.8, 48.4, 42.1, 37.5, 32.2, 26.9, 26.8, 21.8.

HRMS (ESI) m/z: $[M + Na]^+$ Calculated for $C_{10}H_{16}NaO_2$ 191.1043; Found 191.1044.

Compound previously unknown.

13c:

^{1}H NMR (400 MHz, $CDCl_3$) δ 3.83 – 3.77 (m, 1H), 2.43 (ddt, *J* = 10.5, 8.8, 4.6 Hz, 2H), 2.30 – 2.11 (m, 2H), 2.06 (ddt, *J* = 13.7, 10.3, 6.1 Hz, 1H), 1.96 – 1.84 (m, 3H), 1.70 – 1.54 (m, 3H), 1.50 (ddd, *J* = 14.0, 7.2, 4.6 Hz, 1H), 1.07 (s, 3H).

^{13}C NMR (101 MHz, $CDCl_3$) δ 213.5, 77.8, 57.1, 50.2, 38.9, 31.6, 31.2, 22.7, 21.9, 20.3.

HRMS (ESI) m/z: $[M + Na]^+$ Calculated for $C_{10}H_{16}NaO_2$ 191.1043; Found 191.1040.

Compound previously unknown.

(±)-(3aR,7aS)-7a-methylhexahydro-1H-indene-1,6(2H)-dione ***14b*** *&*
(±)-(3aR,7aS)-7a-methylhexahydro-1H-indene-1,4(2H)-dione ***14c***

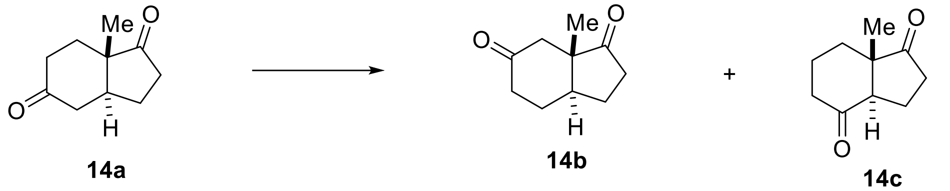

Prepared according to the general procedure with **14a** (166 mg, 1.00 mmol). The crude reaction mixture was purified by automated flash column chromatography (SiO_2, 0–40% EtOAc in hexanes) to afford a fraction of pure **14c** (29 mg, 0.17 mmol, 17% yield), a fraction containing regioisomers **14a** and **14b** (101 mg, 61% yield) and a mixed fraction of **14a**, **14b** and **14c** (4 mg, 2% yield). The regioisomers **14a** and **14b** were then separated by preparative reverse-phase HPLC (Agilent ZORBAX Eclipse XDB-C18 Prep HT 21.2 × 250 mm column, flow rate = 20 mL/min, gradient = 30–35% MeCN in H_2O (containing 0.1% TFA) over 20 minutes) to afford the product **14b** as a colourless oil (24 mg, 0.14 mmol, 14% yield).

14b:

^{1}H NMR (400 MHz, $CDCl_3$) δ 2.57 – 2.35 (m, 4H), 2.34 – 2.24 (m, 1H), 2.20 – 2.06 (m, 3H), 2.06 – 2.01 (m, 1H), 2.00 – 1.84 (m, 2H), 1.10 (s, 3H).

^{13}C NMR (101 MHz, $CDCl_3$) δ 219.3, 209.8, 51.8, 44.1, 42.0, 37.8, 35.2, 27.2, 23.5, 22.0.

HRMS (ESI) m/z: $[M + Na]^+$ Calculated for $C_{10}H_{15}NaO_2$ 189.0886; Found 189.0890.

Compound previously unknown.

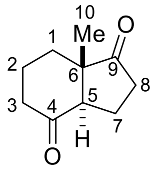

14c

14c:

^{1}H NMR (600 MHz, $CDCl_3$) δ 2.65 – 2.61 (m, 1H, H5), 2.48 – 2.41 (m, 2H, H3, H7), 2.37 – 2.21 (m, 3H, H3, H8, H8), 2.05 – 1.97 (m, 2H, H1, H7), 1.91 (apparent dtdd, *J* = 13.9, 7.7, 4.9, 3.6 Hz, 1H, H2), 1.69 – 1.61 (m, 1H, H2), 1.54 (apparent dddt, *J* = 13.8, 9.0, 3.5, 0.6 Hz, 1H, H1), 1.17 (s, 3H, H10).

^{13}C NMR (150 MHz, $CDCl_3$) δ 219.8 (C9), 211.3 (C4), 57.2 (C5), 53.2 (C6), 39.6 (C3), 36.2 (C8), 30.3 (C1), 23.4 (C10), 22.7 (C2), 20.6 (C7).

HRMS (ESI) m/z: $[M + H]^+$ Calculated for $C_{10}H_{15}O_2$ 167.1067; Found 167.1065.

Compound previously unknown.

*(5S,8R,9S,10S,13S,14S,17S)-17-hydroxy-10,13-dimethylhexadecahydro-2H-cyclopenta[a]phenanthren-2-one **15b** (from Androstanolone)*

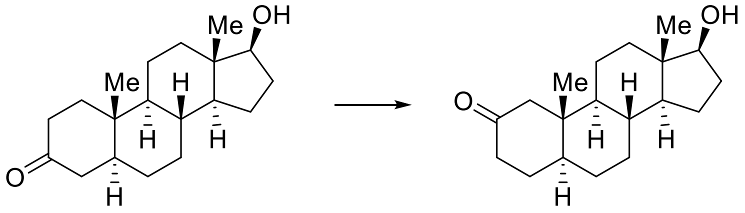

15a **15b**

Prepared according to the general procedure with androstanolone **15a** (291 mg, 1.00 mmol). The crude reaction mixture was purified by preparative reverse-phase HPLC (Agilent ZORBAX Eclipse XDB-C18 Prep HT 21.2 × 250 mm column, flow rate = 18 mL/min, gradient = 50–100% MeCN in H_2O (containing 0.1% formic acid) over 20 minutes) to afford the product **15b** as a white amorphous powder (188 mg, 0.64 mmol, 64% yield).

^{1}H NMR (500 MHz, $CDCl_3$) δ 3.64 (apparent t, *J* = 8.6 Hz, 1H), 2.40 (dd, *J* = 13.2, 2.1 Hz, 1H), 2.38 – 2.27 (m, 2H), 2.12 – 2.02 (m, 1H), 1.98 (apparent dt, *J* = 13.2, 1.0 Hz, 1H), 1.81 (ddd, *J* = 12.5, 4.1, 2.8 Hz, 1H), 1.77 – 1.71 (m, 2H), 1.64 – 1.55 (m, 3H), 1.53 – 1.39 (m, 3H), 1.35 – 1.20 (m, 5H), 1.08 – 1.01 (m, 1H), 1.00 – 0.86 (m, 3H), 0.76 (d, *J* = 1.0 Hz, 3H), 0.73 (d, *J* = 0.7 Hz, 3H).

^{13}C NMR (126 MHz, $CDCl_3$) δ 212.5, 82.0, 54.2, 54.2, 51.1, 45.4, 43.1, 41.5, 40.9, 36.7, 35.1, 31.4, 30.7, 29.4, 28.0, 23.5, 20.7, 12.7, 11.2.

HRMS (ESI) m/z: $[M + H]^+$ Calculated for $C_{19}H_{31}O_2$ 291.2319; Found 291.2317.

Optical rotation: $[\alpha]_{589}^{22.9}$ = +41.8.

Compound previously unknown.

(5S,8R,9S,10S,13S,14S,17S)-17-hydroxy-10,13,17-trimethylhexadecahydro-2H-cyclopenta[a]phenanthren-2-one ***16b*** *(from Mestanolone)*

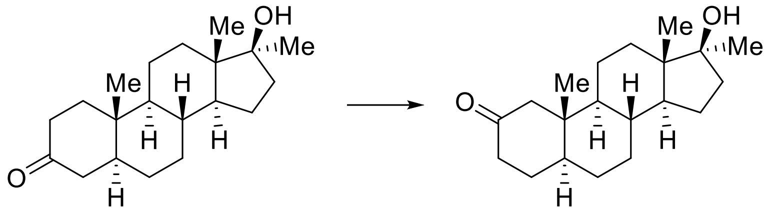

16a **16b**

Prepared according to the general procedure with mestanolone **16a** (305 mg, 1.00 mmol). The crude reaction mixture was purified by automated flash column chromatography (SiO_2, 10–40% EtOAc in hexanes) to give a mixture of the regioisomers **16a** and **16b**. The regioisomers were separated by preparative reverse-phase HPLC (Agilent ZORBAX Eclipse XDB-C18 Prep HT 21.2 × 250 mm column, flow rate = 18 mL/min, gradient = 70–85% MeCN in H_2O (containing 0.1% formic acid) over 20 minutes) to afford the product **16b** as a white amorphous powder (138 mg, 0.45 mmol, 45% yield).

^{1}H NMR (500 MHz, $CDCl_3$) δ 2.38 (dd, *J* = 13.3, 2.1 Hz, 1H), 2.36 – 2.24 (m, 2H), 1.96 (m, 1H), 1.84 – 1.68 (m, 4H), 1.64 – 1.45 (m, 6H), 1.38 – 1.13 (m, 10H), 0.97 – 0.84 (m, 2H), 0.82 (s, 3H), 0.75 (d, *J* = 1.0 Hz, 3H).

^{13}C NMR (126 MHz, $CDCl_3$) δ 212.5, 81.7, 54.2, 54.1, 50.7, 45.6, 45.4, 41.5, 40.8, 39.0, 35.9, 31.6, 31.6, 29.3, 28.0, 25.9, 23.4, 20.7, 14.0, 12.7.

HRMS (ESI) m/z: $[M]^+$ Calculated for $C_{20}H_{32}O_2$ 304.2397; Found 304.2398.

***R_f* (**50% EtOAc in hexanes) = 0.47.

Optical rotation: $[\alpha]_{589}^{23.1}$ = +16.4.

Compound previously unknown.

*(5S,8R,9S,10S,13S,14S)-10,13-dimethyltetradecahydro-1H-cyclopenta[a]phenanthrene-2,17-dione **17b** (from Androstanedione)*

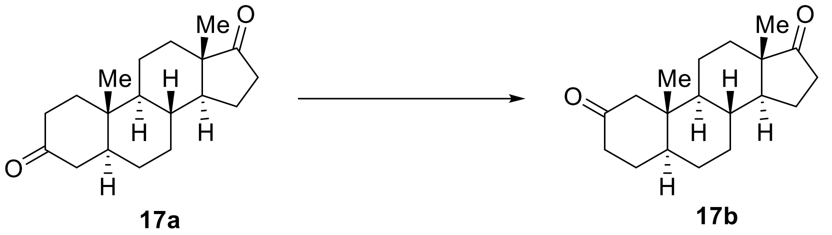

Prepared according to the general procedure with androstanedione **17a** (288 mg, 1.00 mmol). The crude reaction mixture was purified by automated flash column chromatography (SiO_2, 0–50% EtOAc in hexanes) to give a mixture of the regioisomers **17a** and **17b**. The regioisomers were separated by preparative reverse-phase HPLC (Agilent ZORBAX Eclipse XDB-C18 Prep HT 21.2 × 250 mm column, flow rate = 20 mL/min, gradient = 60–70% MeCN in H_2O (containing 0.1% TFA) over 20 minutes) to afford the product **17b** as a white amorphous powder (117 mg, 0.41 mmol, 41% yield).

^{1}H NMR (500 MHz, $CDCl_3$) δ 2.49 – 2.27 (m, 4H), 2.12 – 1.92 (m, 3H), 1.90 – 1.73 (m, 3H), 1.65 – 1.46 (m, 6H), 1.35 – 1.23 (m, 5H), 1.09 – 0.94 (m, 2H), 0.85 (d, *J* = 0.6 Hz, 3H), 0.77 (d, *J* = 1.0 Hz, 3H).

^{13}C NMR (126 MHz, $CDCl_3$) δ 221.0, 212.1, 54.2, 54.1, 51.4, 47.8, 45.4, 41.5, 40.9, 35.9, 34.6, 31.5, 30.7, 29.3, 27.8, 21.9, 20.4, 13.9, 12.7.

HRMS (ESI) m/z: $[M + H]^+$ Calculated for $C_{19}H_{29}O_2$ 289.2162; Found 289.2161.

Optical rotation: $[\alpha]_{589}^{23.2}$ = +99.9.

Compound previously unknown.

*(5S,8R,9S,10S,13S,14S,17S)-17-hydroxy-10,13,17-trimethylhexadecahydro-2H-cyclopenta[a]phenanthren-2-one **18b** (from Allopregnanedione)*

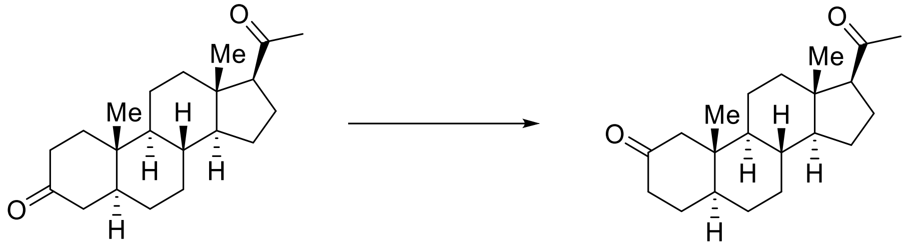

18a **18b**

Prepared according to the general procedure with allopregnanedione **18a** (317 mg, 1.00 mmol). The crude reaction mixture was purified by automated flash column chromatography (SiO_2, 10–30% EtOAc in hexanes) to give a mixture of regioisomers **18a** and **18b** (220 mg, 69% yield). The isomers were separated by preparative reverse-phase HPLC (Agilent ZORBAX Eclipse XDB-C18 Prep HT 21.2 × 250 mm column, flow rate = 20 mL/min, gradient = 55–60% MeCN in H_2O (containing 0.1% TFA) over 20 minutes) to afford the product **18b** as a white amorphous powder (98 mg, 0.31 mmol, 31% yield).

^{1}H NMR (500 MHz, $CDCl_3$) δ 2.52 (apparent t, *J* = 9.0 Hz, 1H), 2.44 – 2.25 (m, 3H), 2.21 – 2.12 (m, 1H), 2.11 (s, 3H), 2.06 – 1.93 (m, 2H), 1.79 – 1.55 (m, 6H), 1.55 – 1.47 (m, 2H), 1.47 – 1.10 (m, 6H), 1.05 – 0.88 (m, 2H), 0.74 (d, *J* = 1.0 Hz, 3H), 0.60 (s, 3H).

^{13}C NMR (126 MHz, $CDCl_3$) δ 212.4, 209.6, 63.9, 56.7, 54.2, 54.0, 45.3, 44.2, 41.5, 40.8, 39.0, 35.0, 31.8, 31.7, 29.3, 28.0, 24.6, 22.9, 21.1, 13.5, 12.7.

HRMS (ESI) m/z: $[M + Na]^+$ Calculated for $C_{21}H_{32}NaO_2$ 339.2295; Found 339.2292.

***R_f* (**20% EtOAc in hexanes) = 0.36

Optical rotation: $[\alpha]_{589}^{24.7}$ = +127.4.

Characterization data not previously reported.

7. Deuterium labeling studies

Standard reaction conditions in methanol

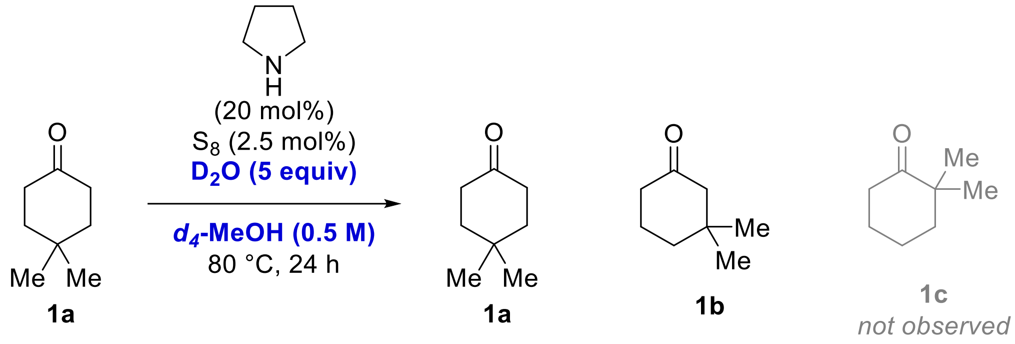

To an oven-dried screw-cap vial was added 4,4-dimethylcyclohexanone **1a** (126 mg, 1.00 mmol), S_8 (6.4 mg, 0.025 mmol, 2.5 mol%, 20 mol% S), *d*$_4$-MeOH (2.0 mL), D_2O (90 µL, 5 mmol, 5 equiv) and pyrrolidine (16 µL, 0.10 mmol, 20 mol%). The vial was flushed with N_2, sealed and then heated at 80 °C for 24 hours. After cooling to room temperature, the reaction was diluted with Et_2O (8 mL) and filtered through a plug of silica. The reaction mixture and the silica plug were washed with further 10 mL of Et_2O and the crude reaction mixture was concentrated under reduced pressure. The crude was then purified by distillation (50 °C at 12 mbar) to yield a mixture of isomers **1a** and **1b** as a transparent oil. The mixture was analyzed by ^1H and ^2H NMR spectroscopy.

Relative Isomer Distribution (**1a**:**1b**): 1:1.5.

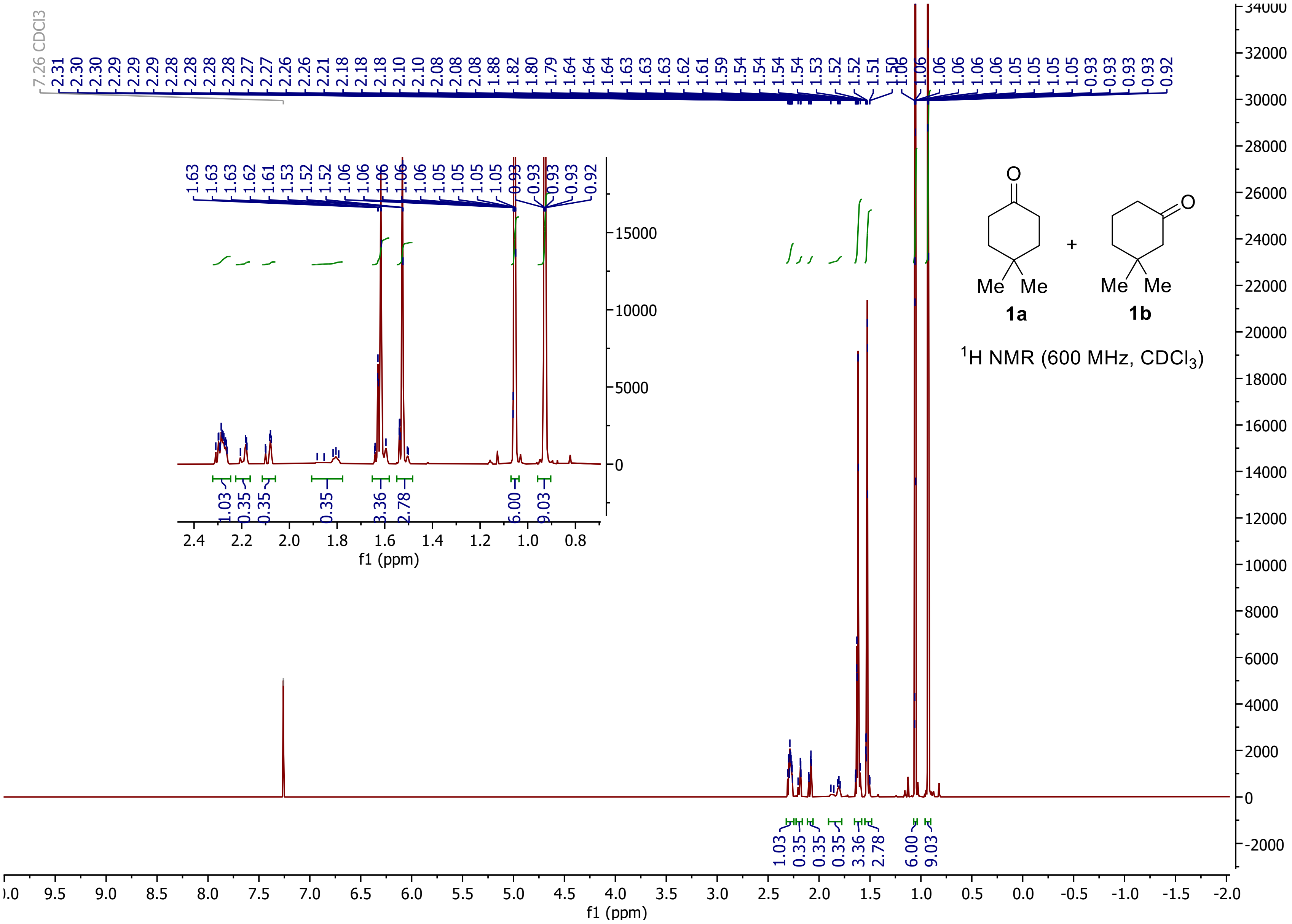

1a
1b
^{1}H NMR (600 MHz, CDCl$_3$)
f1 (ppm)

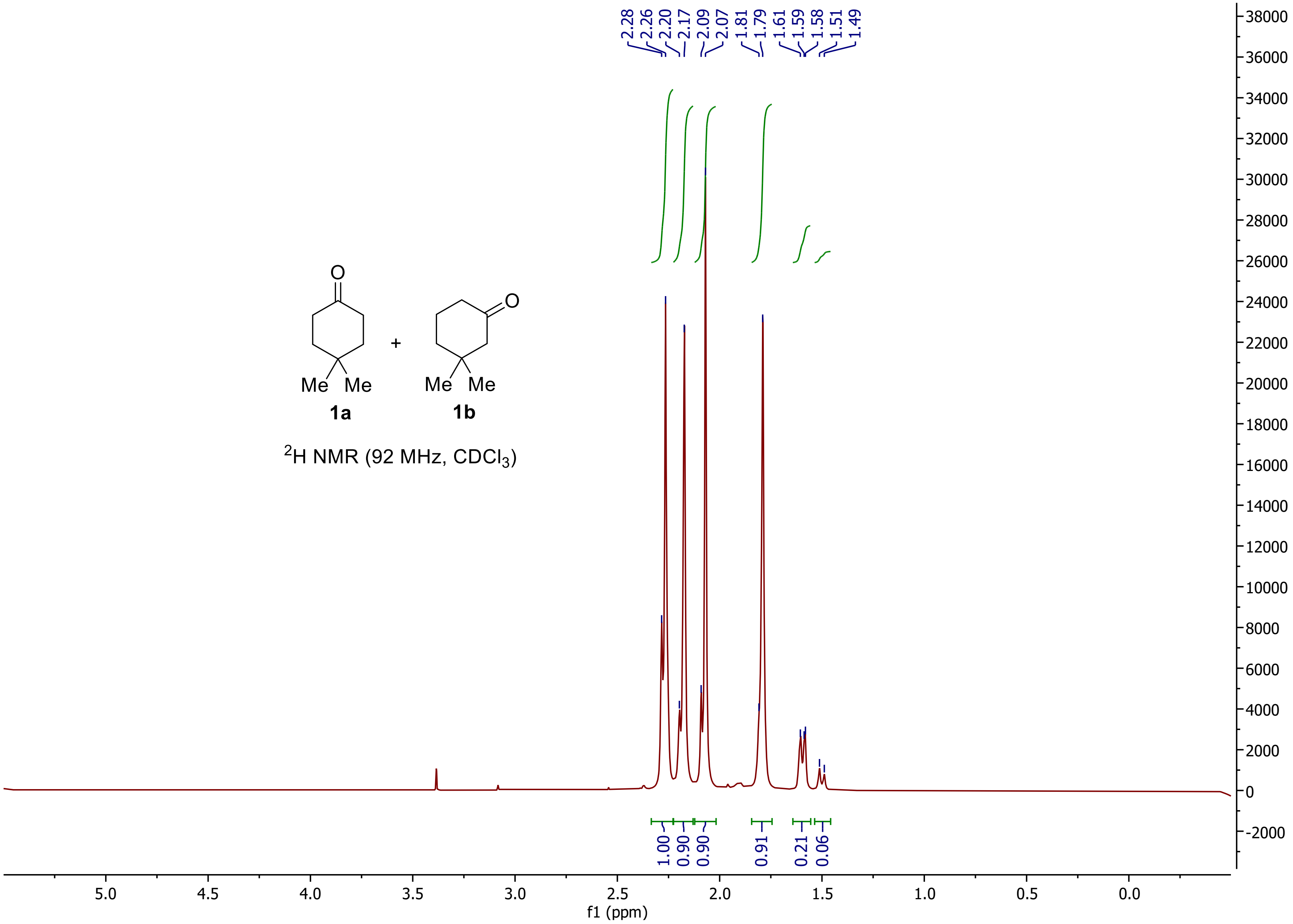

1a
1b
Me Me
O
²H NMR (92 MHz, CDCl₃)
2.28 2.26 2.20 2.17 2.09 2.07 1.81 1.79 1.61 1.59 1.58 1.51 1.49
1.00 0.90 0.90 0.91 0.21 0.06
f1 (ppm)

Standard reaction conditions in methanol without sulfur

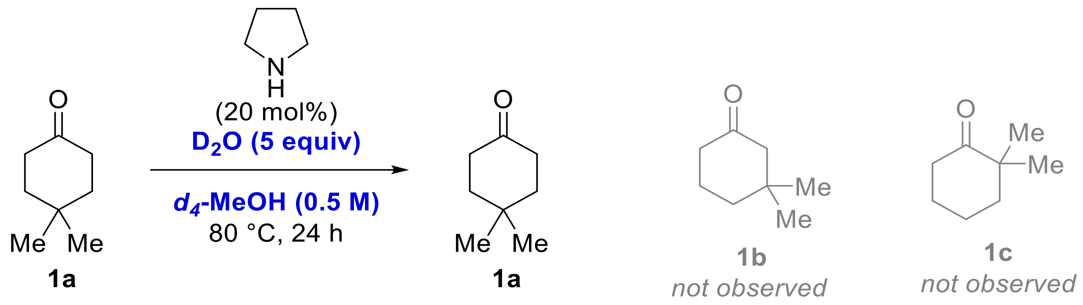

To an oven-dried screw-cap vial was added 4,4-dimethylcyclohexanone **1a** (126 mg, 1.00 mmol), *d*$_4$-MeOH (2.0 mL), D_2O (90 µL, 5 mmol, 5 equiv) and pyrrolidine (16 µL, 0.10 mmol, 20 mol%). The vial was flushed with N_2, sealed and then heated at 80 °C for 24 hours. After cooling to room temperature, the reaction was diluted with Et_2O (8 mL) and filtered through a plug of silica. The reaction mixture and the silica plug were washed with further 10 mL of Et_2O and the crude reaction mixture was concentrated under reduced pressure. The crude was then purified by distillation (50 °C at 12 mbar) to yield pure **1a** as a transparent oil. The product was analyzed by ^{1}H and ^{2}H NMR.

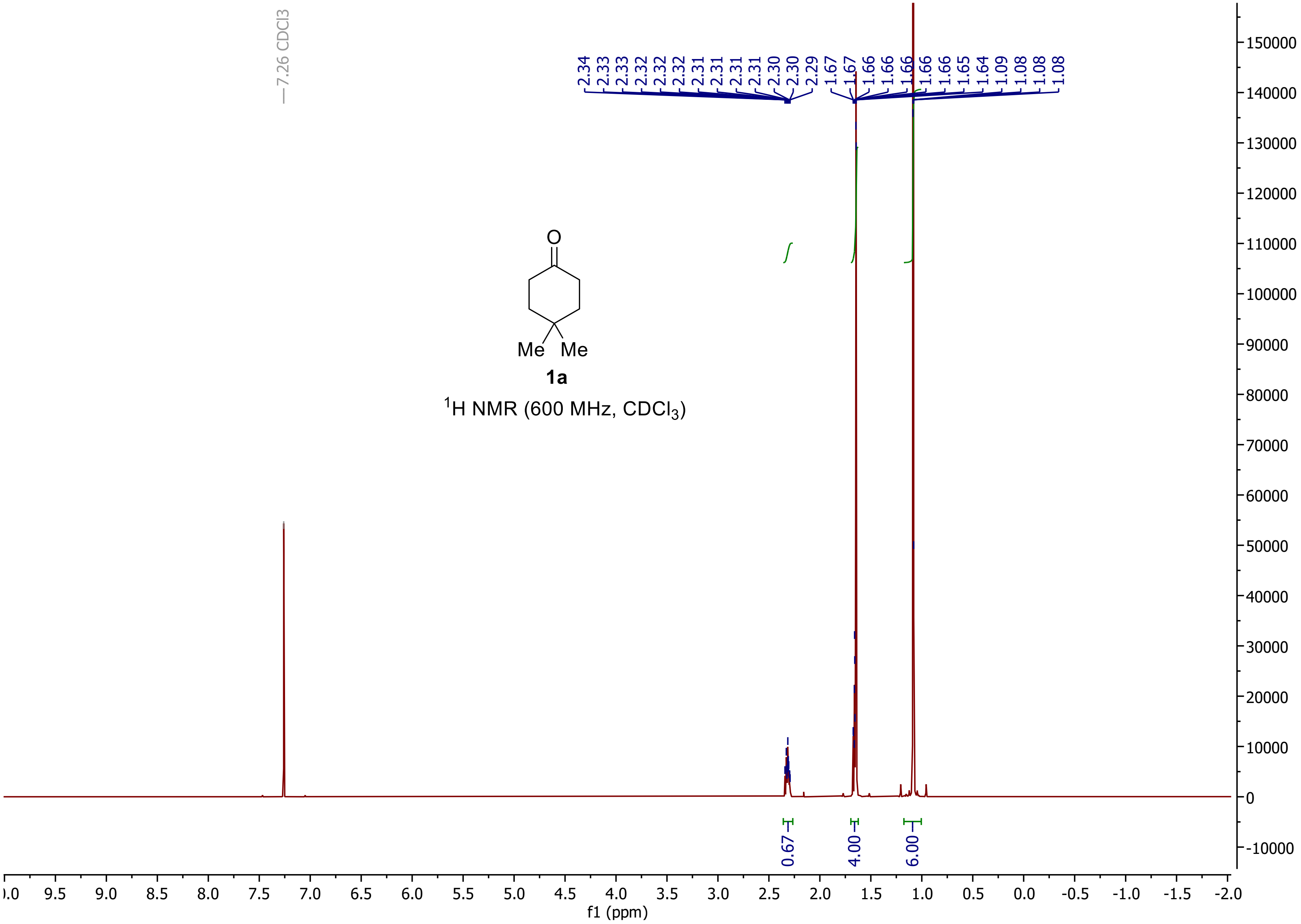

7.26 CDCl3
2.34
2.33
2.33
2.32
2.32
2.32
2.31
2.31
2.31
2.30
2.30
2.29
1.67
1.67
1.66
1.66
1.66
1.66
1.65
1.64
1.09
1.08
1.08
1.08
O
Me Me
1a
^{1}H NMR (600 MHz, $CDCl_3$)
0.67
4.00
6.00
f1 (ppm)

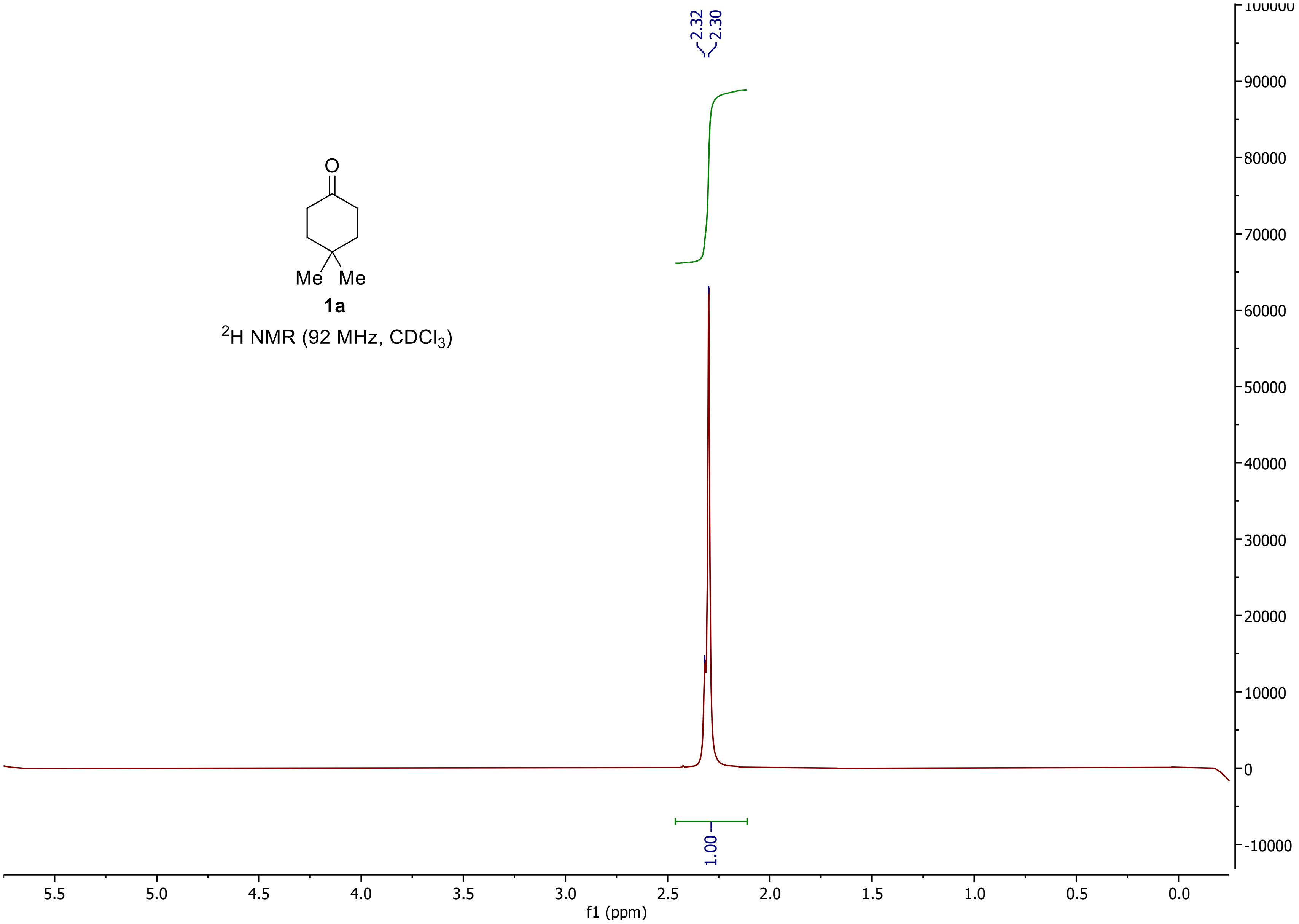

2.32
2.30
O
Me
Me
1a
^{2}H NMR (92 MHz, CDCl$_3$)
1.00
f1 (ppm)

8. Computational details

All calculations were run on the Euler cluster at ETH Zürich.

Initial conformer searches were performed with xTB version 6.4.0[1,2] and CREST version 2.11.[3]

The three lowest energy conformers were further optimized using by DFT using ORCA version 4.2.1.[4] Only the lowest energy conformers are reported.

Geometry and frequency optimizations were performed with both the PBE0[5] and B3LYP[6,7] functionals, using the def2-QZVPP basis set.[8] Both the DFT and COSX integration grid were set to 5 and optimization settings were set to *tight*. Grimme's atom-pairwise dispersion correction with the Becke-Johnson damping scheme was used[9,10] and the def2/J auxiliary basis set was chosen.[11] The cpcm solvation[12] was used for *iso*-propanol, setting the dielectric constant (epsilon) to 18.3 and the refraction index (refract) to 1.377.

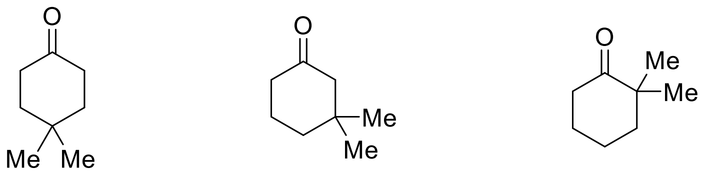

1a **1b** **1c**

	**1a**	**1b**	**1c**
**B3LYP**	Δ*G* = 0 kcal/mol	Δ*G* = -0.7 kcal/mol	Δ*G* = 1.2 kcal/mol
**PBE0**	Δ*G* = 0 kcal/mol	Δ*G* = -0.6 kcal/mol	Δ*G* = 1.3 kcal/mol

References

1. Bannwarth, C.; Ehlert, S.; Grimme, S. GFN2-xTB—An Accurate and Broadly Parametrized Self-Consistent Tight-Binding Quantum Chemical Method with Multipole Electrostatics and Density-Dependent Dispersion Contributions. *J. Chem. Theory Comput*. **2019**, *15*, 1652–1671. https://doi.org/10.1021/acs.jctc.8b01176

2. Bannwarth, C.; Caldeweyher, E.; Ehlert, S.; Hansen, A.; Pracht, P.; Seibert, J.; Spicher, S.; Grimme, S. Extended tight-binding quantum chemistry methods. *WIREs Computational Molecular Science* **2020**, 11. https://doi.org/10.1002/wcms.1493

3. Pracht, P.; Bohle, F; Grimme, S. Automated exploration of the low-energy chemical space with fast quantum chemical methods. *Phys. Chem. Chem. Phys.* **2020**, *22*, 7169–7192. https://doi.org/10.1039/C9CP06869D

4. Neese, F. Software Update: The ORCA Program System, Version 4.0. *Wiley Interdisciplinary Reviews: Computational Molecular Science* **2018**, 8, e1327. https://doi.org/10.1002/wcms.1327

5. Adamo, C.; Barone, V. Toward Reliable Density Functional Methods without Adjustable Parameters: The PBE0 Model. *J. Chem. Phys*. **1999**, *110*, 6158–6170. https://doi.org/10.1063/1.478522

6. Becke, A. D. Density-functional thermochemistry. III. The role of exact exchange. *J. Chem. Phys.* **1993**, *98*, 5648−5652. https://doi.org/10.1063/1.464913

7. Lee, C.; Yang, W.; Parr, R. G. Development of the Colle-Salvetti correlation-energy formula into a functional of the electron density. *Phys. Rev. B* **1988**, *37*, 785–789. https://doi.org/10.1002/qua.24876

8. Weigend, F.; Ahlrichs, R. Balanced Basis Sets of Split Valence, Triple Zeta Valence and Quadruple Zeta Valence Quality for H to Rn: Design and Assessment of Accuracy. *Phys. Chem. Chem. Phys.* **2005**, *7*, 3297–3305. https://doi.org/10.1039/B508541A.

9. Grimme, S.; Ehrlich, S.; Goerigk, L. Effect of the Damping Function in Dispersion Corrected Density Functional Theory. *J. Comput. Chem*. **2011**, *32*, 1456–1465. https://doi.org/10.1002/jcc.21759

10. Grimme, S., Antony, J., Ehrlich, S., & Krieg, H. A consistent and accurate ab initio parametrization of density functional dispersion correction (DFT-D) for the 94 elements H-Pu. *J. Chem. Phys.* **2010**, *132*, 154104. https://doi.org/10.1063/1.3382344

11. Weigend, F. Accurate Coulomb-Fitting Basis Sets for H to Rn. *Phys. Chem. Chem. Phys.* **2006**, 8, 1057. https://doi.org/10.1039/b515623h

12. Barone, V.; Cossi, M. Quantum Calculation of Molecular Energies and Energy Gradients in Solution by a Conductor Solvent Model. *J. Phys. Chem. A* **1998**, *102*, 1995–2001. https://doi.org/10.1021/jp9716997

*4,4-dimethylcyclohexanone **1a** (B3LYP)*

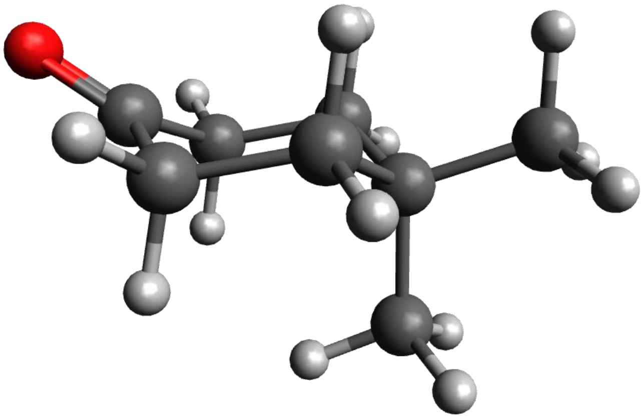

C	0.96535312546217	0.12010724005272	0.05530825006611
C	2.49896433532228	0.10241219981536	-0.01094617330586
C	3.01692216616644	1.12834672429739	-1.03579570218835
C	2.66980124626618	0.77650046848167	-2.49177876392560
C	3.05407070673186	0.45863654874110	1.37225280516189
C	3.01652819525667	-1.29087566109804	-0.41140225975662
C	2.66233681145287	-1.68856681574832	-1.85331847936120
C	3.08362831686289	-0.63030608107927	-2.84031883039608
O	3.71702191283302	-0.89376364831308	-3.84815726016789
H	0.61068616881223	1.10618658600722	0.35829300162317
H	0.60900495211216	-0.60619039034991	0.78693852461848
H	0.49966467147270	-0.11900089742184	-0.89954897892280
H	4.10363908684760	1.19303611200288	-0.94328245641964
H	2.62074752580033	2.11783717246679	-0.80277030155439
H	3.13295646659269	1.47102411497937	-3.19003241012039
H	1.58718421618076	0.83445981093036	-2.63534383345496
H	2.72198690413032	1.45178274239982	1.67878420476750
H	4.14500008393185	0.45123305740131	1.36790383661232
H	2.71395992076470	-0.25674170339511	2.12250408751436
H	2.62593923539079	-2.04366326106690	0.27485200402697
H	4.10397699271500	-1.30094160159167	-0.30554426002047
H	1.57889953911263	-1.80142642427007	-1.94946098696582
H	3.11874741978186	-2.63769629324175	-2.12717601783070

*4,4-dimethylcyclohexanone **1a** (PBE0)*

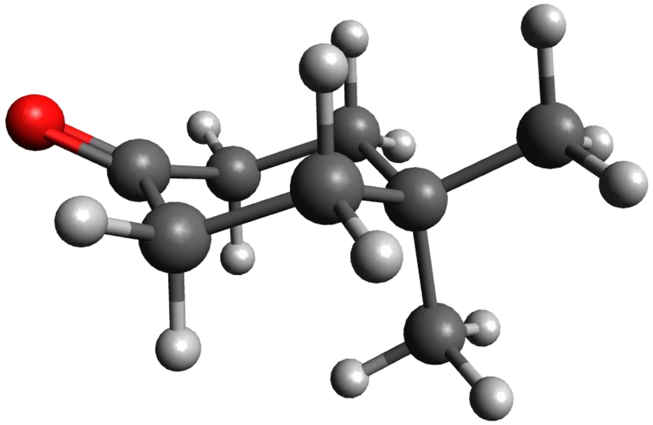

C	0.97375658748454	0.11764386962937	0.04748507354223
C	2.49935723401092	0.10130642344644	-0.01529369792153
C	3.01778102150971	1.12155361455564	-1.03343313786397
C	2.67133617360112	0.77325684884625	-2.48122230430541
C	3.04864341870098	0.45598657277402	1.36156813978825
C	3.01788158576711	-1.28339702193684	-0.41283804533336
C	2.66259577580487	-1.68040707348968	-1.84533424211849
C	3.07931982567657	-0.62822872598644	-2.83124847895693
O	3.69991414658194	-0.89299947360468	-3.84193551848047
H	0.61743216090413	1.10419937728386	0.35192639071563
H	0.61611622956138	-0.61037297935139	0.77878908352040
H	0.50756058080916	-0.12102315516364	-0.90932038737659
H	4.10638531340990	1.18258083017518	-0.93834323023632
H	2.62509693289508	2.11371317171897	-0.79759145353029
H	3.13197133728154	1.46808662377354	-3.18297405889989
H	1.58667030414774	0.82975716129498	-2.62422936169955
H	2.71543501740548	1.45035896664587	1.66765094429998
H	4.14100677192173	0.44839812199209	1.35915029136111
H	2.70686481758513	-0.26006143307731	2.11232361916992
H	2.63236728421076	-2.03730592961139	0.27782940261321
H	4.10737116584331	-1.28822144101776	-0.30803080465419
H	1.57695673279043	-1.79035403248087	-1.94097954757215
H	3.11519958209644	-2.63208031641618	-2.12198867606155

*3,3-dimethylcyclohexanone **1b** (B3LYP)*

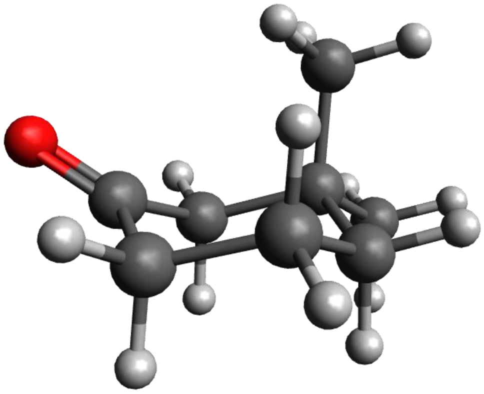

O	1.02693485434970	0.07040423160029	0.29696849118312
C	2.24079218536129	0.02921821609001	0.18977667913946
C	2.99878766373509	-1.26897363722232	0.09378344013254
C	3.92749308267597	-1.31575212808632	-1.14532659982225
C	3.07335148921275	-1.37130618728319	-2.41941742312267
C	4.79542603481810	-2.57415299584475	-1.06767072817624
C	4.82882974739801	-0.06711492810921	-1.13797488635089
C	4.05057214170049	1.24546983972308	-1.06482754516878
C	3.09138839531519	1.27191242059428	0.13758618894844
H	2.29568824301682	-2.10078455329777	0.08633720457541
H	3.61544084076714	-1.34933271514771	0.99396142511046
H	2.45928041871697	-2.27265443656077	-2.42549991861540
H	3.71047827476953	-1.38707961453614	-3.30435926788963
H	2.40238974079953	-0.51698339741056	-2.50637878229689
H	4.17708031830944	-3.47225947253435	-1.03346429163137
H	5.42403386071630	-2.55954862155798	-0.17624837050694
H	5.44516961194211	-2.64480678037582	-1.94140775620700
H	5.45639459544789	-0.07569661506103	-2.03131224200995
H	5.50122763363948	-0.13190066749593	-0.27803562099911
H	3.47878422289445	1.39635621655526	-1.98122352559451
H	4.74089619487382	2.08510003233681	-0.98643336069108
H	2.45067220749778	2.15131397910727	0.12754769561338
H	3.68189824204207	1.28876181451686	1.05910919437992

*3,3-dimethylcyclohexanone **1b** (PBE0)*

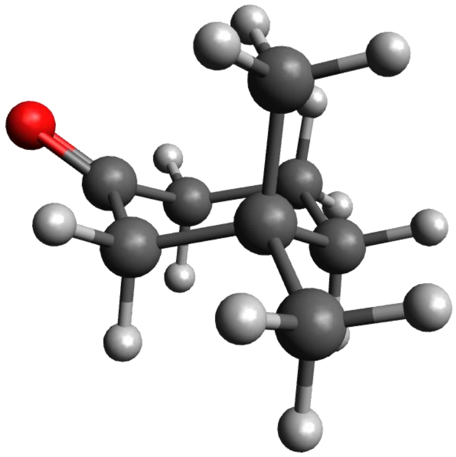

O	1.05974387429321	-0.04725814825170	0.12178138180721
C	2.27102824867180	-0.07855515647264	0.02740549169395
C	3.04534364492566	-1.36376542301302	0.01480159761331
C	4.01551567538321	-1.41942102548670	-1.16658337773536
C	4.86879210052199	-0.16429998684470	-1.24920736523191
C	4.04928364197240	1.12916422337604	-1.29833583813572
C	3.22570747420541	1.20899297960320	-2.58171679176460
C	4.98748595229225	2.32668732592968	-1.23015482930641
C	3.10296589158867	1.16536150242708	-0.08371585338389
H	2.35343323424485	-2.20524976567730	0.01932997552091
H	3.62192537033846	-1.39058184182779	0.94675208707971
H	3.44916321666877	-1.55344516160982	-2.09111466962833
H	4.65255489484486	-2.29929949997974	-1.06327425524126
H	5.51489548611991	-0.21036964920448	-2.13012336139848
H	5.52827277966354	-0.12090130527012	-0.37558575604639
H	3.87779627089475	1.17205477620017	-3.45675430960942
H	2.66407107251183	2.14490824188693	-2.61482129973401
H	2.50669049361617	0.39187995104095	-2.66518656320605
H	5.65231097667856	2.34155941814649	-2.09691221004460
H	5.60355869901776	2.28859399978109	-0.32907213201822
H	4.42563549530042	3.26324304768309	-1.21949749220255
H	2.45008957523092	2.03843517193772	-0.12205175399479
H	3.70745593101454	1.22901632562558	0.82818732496687

*2,2-dimethylcyclohexanone **1c** (B3LYP)*

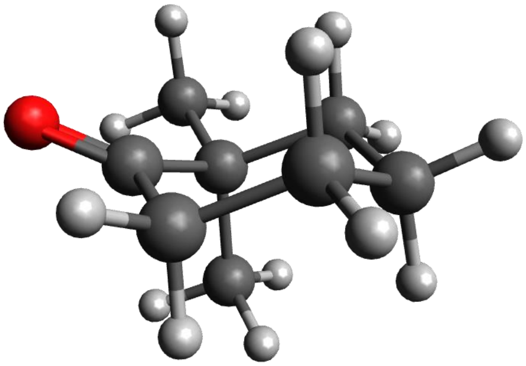

O	1.21154228619806	-0.01165606774283	0.21757377498378
C	2.41931488290888	-0.02656349131736	0.06188223004640
C	3.21686340019946	-1.33063424609287	0.03326972114325
C	4.01317622949554	-1.42674568759789	1.35265340958382
C	2.28284241358570	-2.53453307517808	-0.07894340733955
C	4.18141621851239	-1.30623936062337	-1.17868412190402
C	5.02512222782616	-0.03816806126756	-1.28414767894908
C	4.13482488460461	1.20058176484401	-1.32674902545840
C	3.20223293252153	1.25463823524649	-0.10684717872052
H	3.33966022073990	-1.42772261410254	2.20962785291998
H	4.71826275275046	-0.60824444462002	1.47546436502709
H	4.57548636484305	-2.36082686333831	1.35917702385748
H	1.61866716120903	-2.60300164973681	0.78098863768674
H	2.87277108256491	-3.44985878668791	-0.13045013130527
H	1.66844036371355	-2.47204549331133	-0.97662816858982
H	3.58659983909639	-1.40887350390288	-2.09032230639033
H	4.81898462672061	-2.19023413549610	-1.11790539595014
H	5.64066485394202	-0.08689284654995	-2.18351504126183
H	5.71363219372852	0.03224586231239	-0.43948083534642
H	3.52887791296583	1.17987901685310	-2.23607627868620
H	4.73321909477767	2.11101220273348	-1.36215701119429
H	2.49847646521878	2.08219720651338	-0.17225427061157
H	3.80248159187689	1.39990603906296	0.79514383645888

*2,2-dimethylcyclohexanone **1c** (PBE0)*

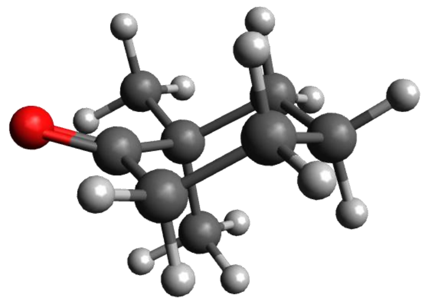

O	1.22024220253285	-0.01683063124099	0.21551532412190
C	2.42417126002522	-0.03003728835467	0.05933026292724
C	3.21911097802597	-1.32745525139367	0.03064763689052
C	4.01186225064011	-1.42083469314306	1.34203278772295
C	2.28893786489211	-2.52406244080791	-0.07956171332826
C	4.17732171355721	-1.30228594042538	-1.17482904517912
C	5.01848189167254	-0.04129376364824	-1.27604481210979
C	4.13168750844850	1.19059230051862	-1.32184397621128
C	3.20424704034955	1.24517721841811	-0.10854701291977
H	3.33909729835124	-1.42497681934155	2.20136364197417
H	4.71664268336366	-0.59975083069667	1.46591216633439
H	4.57756711426968	-2.35467825256338	1.34737381884924
H	1.62388799196572	-2.59073062252245	0.78161676369532
H	2.87871401843160	-3.44116700581915	-0.13017192429511
H	1.67349324225010	-2.46208244403407	-0.97825758947616
H	3.58127223909110	-1.40264365937214	-2.08813094909130
H	4.81287725064481	-2.19009678028421	-1.11601983049933
H	5.63984658767084	-0.08999064176387	-2.17290760593912
H	5.70483338026416	0.02924473786935	-0.42733507798266
H	3.52559423058819	1.16691987305165	-2.23285090465249
H	4.72915835781644	2.10309036002736	-1.36062022129043
H	2.49950975301322	2.07397302549023	-0.17062263265133
H	3.80500314213511	1.38813955003608	0.79557089311041

9. Crystallographic Data

(±)-(1S,4aS,8aS)-8a-methyl-7-oxodecahydronaphthalen-1-yl benzoate ***8b***

Crystals were grown by dissolution in MTBE and then leaving the solution standing at room temperature.

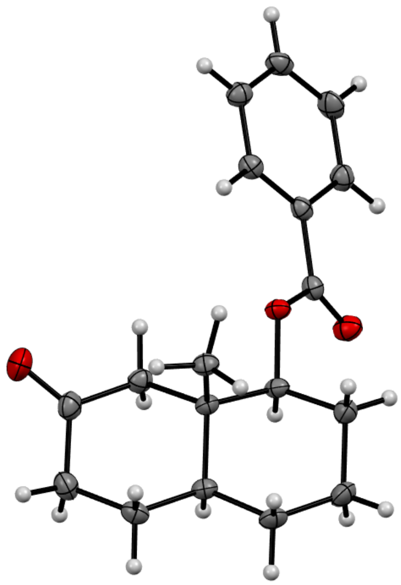

Identification code	bm220623_1_1
Empirical formula	$C_{18}H_{22}O_3$
Formula weight	286.35
Temperature [K]	100.0(1)
Crystal system	triclinic
Space group (number)	$P\bar{1}$ (2)
a [Å]	9.7985(3)
b [Å]	12.8775(3)
c [Å]	13.7370(6)
α [°]	64.984(3)
β [°]	78.541(3)
γ [°]	76.356(2)
Volume [Å^3]	1516.75(10)
Z	4
ρ_{calc} [gcm^{-3}]	1.254
μ [mm^{-1}]	0.671

F(000)	616
Crystal size [mm^3]	0.212×0.044×0.027
Radiation	Cu K_α (λ=1.54184 Å)
2θ range [°]	7.15 to 159.67
Index ranges	$-12 \le h \le 9$<br>$-16 \le k \le 16$<br>$-17 \le l \le 17$
Reflections collected	23863
Independent reflections	6155<br>R_{int} = 0.0332<br>R_{sigma} = 0.0309
Data / Restraints / Parameters	6155/0/381
Goodness-of-fit on F^2	1.041
Final R indexes [$I \ge 2\sigma(I)$]	R_1 = 0.0355<br>wR_2 = 0.0900
Final R indexes [all data]	R_1 = 0.0432<br>wR_2 = 0.0941
Largest peak/hole [eÅ^{-3}]	0.25/-0.22

(±)-(1S,4aS,8aS)-8a-methyl-7-oxodecahydronaphthalen-1-yl 3-methylbut-2-enoate ***10b***

Crystals were grown by dissolution in MeCN/H_2O (0.7:0.3) and then leaving the solution standing at room temperature.

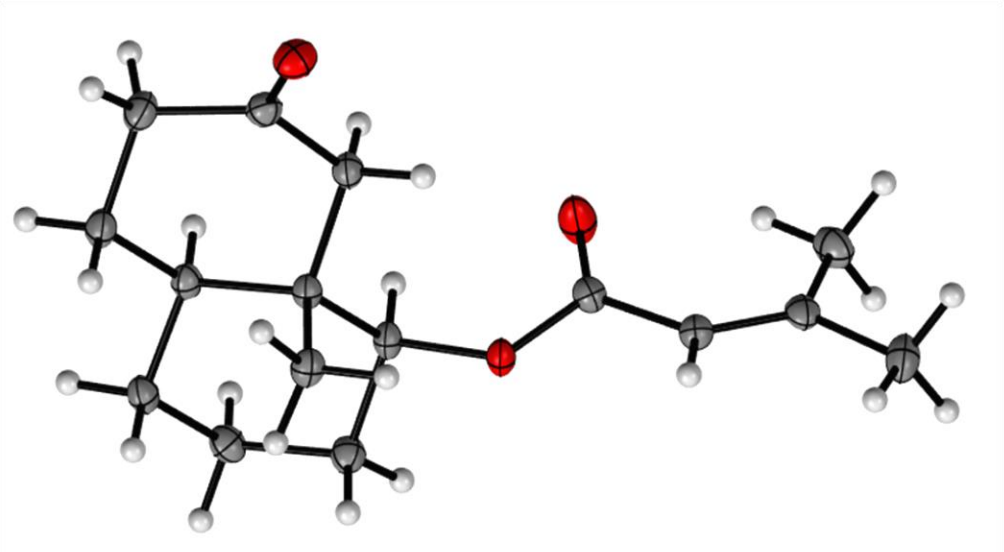

Identification code	bm150622_1_1
Empirical formula	$C_{16}H_{24}O_3$
Formula weight	264.35
Temperature [K]	100.0(1)
Crystal system	monoclinic
Space group (number)	$P2_1/n$
a [Å]	7.31590(10)
b [Å]	17.0092(2)
c [Å]	11.81780(10)
α [°]	90
β [°]	103.0590(10)
γ [°]	90
Volume [Å^3]	1432.55(3)
Z	4

ρ_{calc} [gcm^{-3}]	1.226
μ [mm^{-1}]	0.661
F(000)	576.0
Crystal size [mm^3]	0.238 × 0.167 × 0.121
Radiation	Cu Kα (λ = 1.54184)
2θ range [°]	9.276 to 160.276
Index ranges	$-7 \leq h \leq 9$, $-21 \leq k \leq 21$, $-14 \leq l \leq 15$
Reflections collected	29418
Independent reflections	3096 [R_{int} = 0.0378, R_{sigma} = 0.0169]
Data / Restraints / Parameters	3096/0/175
Goodness-of-fit on F^2	1.071
Final *R* indexes [$I \geq 2\sigma(I)$]	R_1 = 0.0368, wR_2 = 0.0968
Final *R* indexes [all data]	R_1 = 0.0393, wR_2 = 0.0985
Largest peak/hole [eÅ^{-3}]	0.30/-0.20

(5S,8R,9S,10S,13S,14S,17S)-17-hydroxy-10,13-dimethylhexadecahydro-2H-cyclopenta[a]phenanthren-2-one ***15b***

Crystals were grown by dissolution in acetone/hexane (1:1) and then leaving the solution standing at room temperature.

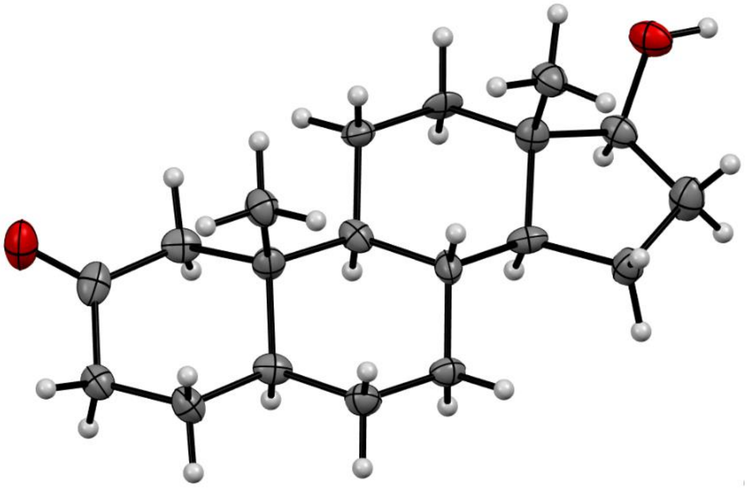

Identification code	bm200621_2_1
Empirical formula	$C_{19}H_{30}O_2$
Formula weight	290.43
Temperature [K]	100.0(1)
Crystal system	monoclinic
Space group (number)	$P2_1$
a [Å]	6.4100(2)
b [Å]	11.9462(4)
c [Å]	20.9908(7)
α [°]	90
β [°]	95.653(3)
γ [°]	90
Volume [Å^3]	1599.55(9)
Z	4

ρ_{calc} [gcm^{-3}]	1.206
μ [mm^{-1}]	0.584
F(000)	640.0
Crystal size [mm^3]	0.314 × 0.191 × 0.027
Radiation	Cu Kα (λ = 1.54184)
2θ range [°]	4.23 to 134.448
Index ranges	$-6 \leq h \leq 7$, $-14 \leq k \leq 14$, $-24 \leq l \leq 25$
Reflections collected	6101
Independent reflections	6101 [R_{int} = 0.0370, R_{sigma} = 0.0241]
Data / Restraints / Parameters	6101/3/390
Goodness-of-fit on F^2	1.062
Final *R* indexes [$I \geq 2\sigma(I)$]	R_1 = 0.0616, wR_2 = 0.1692
Final *R* indexes [all data]	R_1 = 0.0661, wR_2 = 0.1739
Largest peak/hole [eÅ^{-3}]	0.40/-0.30
Flack X parameter	-0.1(4)

(5S,8R,9S,10S,13S,14S,17S)-17-hydroxy-10,13,17-trimethylhexadecahydro-2H-cyclopenta[a]phenanthren-2-one ***16b***

Crystals were grown by dissolution in CH_2Cl_2/hexane (1:1) and then leaving the solution standing at room temperature.

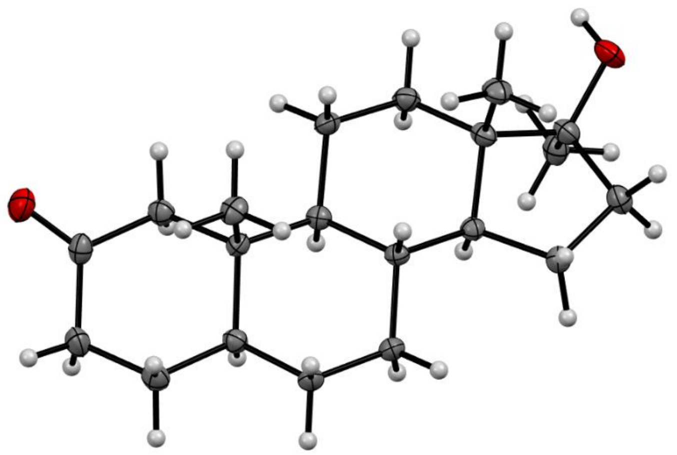

Identification code	bm280621_1_1
Empirical formula	$C_{20}H_{32}O_2$
Formula weight	304.45
Temperature [K]	100.0(1)
Crystal system	orthorhombic
Space group (number)	$P2_12_12_1$
a [Å]	6.49710(10)
b [Å]	11.98870(10)
c [Å]	21.5897(2)
α [°]	90
β [°]	90
γ [°]	90
Volume [Å^3]	1681.66(3)
Z	4

ρ_{calc} [gcm^{-3}]	1.203
μ [mm^{-1}]	0.577
F(000)	672.0
Crystal size [mm^3]	0.309 × 0.148 × 0.072
Radiation	Cu Kα (λ = 1.54184)
2θ range [°]	8.19 to 161.198
Index ranges	-8 ≤ h ≤ 7, -15 ≤ k ≤ 15, -27 ≤ l ≤ 27
Reflections collected	41362
Independent reflections	3618 [R_{int} = 0.0729, R_{sigma} = 0.0265]
Data / Restraints / Parameters	3618/1/205
Goodness-of-fit on F^2	1.058
Final *R* indexes [$I \geq 2\sigma(I)$]	R_1 = 0.0363, wR_2 = 0.0936
Final *R* indexes [all data]	R_1 = 0.0384, wR_2 = 0.0989
Largest peak/hole [eÅ^{-3}]	0.18/-0.18
Flack X parameter	0.20(11)

(5S,8R,9S,10S,13S,14S)-10,13-dimethyltetradecahydro-1H-cyclopenta[a]phenanthrene-2,17-dione ***17b***

Crystals were grown by dissolution in acetone and then leaving the solution standing at room temperature.

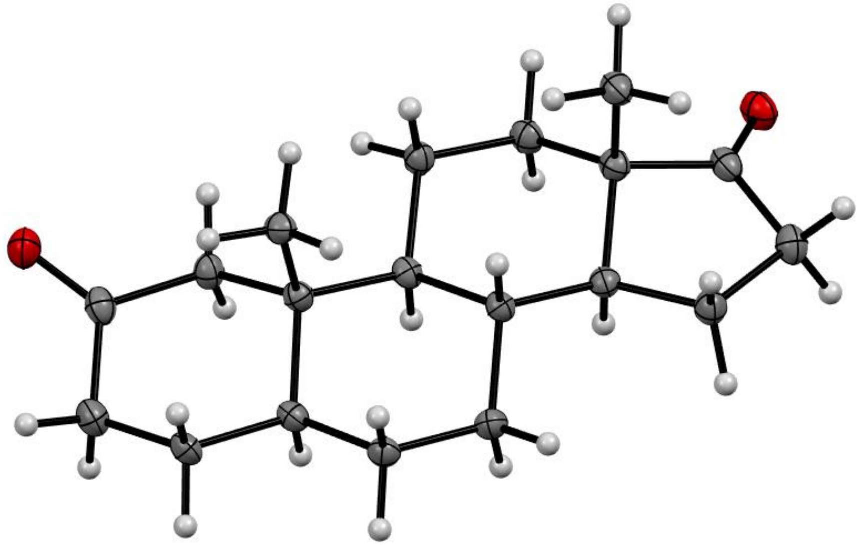

Identification code	bm200623_2_2
Empirical formula	$C_{19}H_{28}O_2$
Formula weight	288.41
Temperature [K]	100.0(1)
Crystal system	monoclinic
Space group (number)	$P2_1$ (4)
a [Å]	6.65720(10)
b [Å]	37.2520(4)
c [Å]	7.02540(10)
α [°]	90
β [°]	116.997(2)
γ [°]	90
Volume [Å^3]	1552.40(4)
Z	4
ρ_{calc} [gcm^{-3}]	1.234
μ [mm^{-1}]	0.602

F(000)	632
Crystal size [mm^3]	0.252×0.215×0.05
Radiation	Cu K_α (λ=1.54184 Å)
2θ range [°]	9.50 to 160.35
Index ranges	$-7 \leq h \leq 8$<br>$-46 \leq k \leq 46$<br>$-8 \leq l \leq 8$
Reflections collected	31521
Independent reflections	6346<br>R_{int} = 0.0421<br>R_{sigma} = 0.0287
Data / Restraints / Parameters	6346/1/383
Goodness-of-fit on F^2	1.061
Final R indexes [$I \geq 2\sigma(I)$]	R_1 = 0.0327<br>wR_2 = 0.0854
Final R indexes [all data]	R_1 = 0.0338<br>wR_2 = 0.0861
Largest peak/hole [eÅ^{-3}]	0.18/-0.23
Flack X parameter	-0.01(8)

(5S,8R,9S,10S,13S,14S,17S)-17-acetyl-10,13-dimethylhexadecahydro-2H-cyclopenta[a]phenanthren-2-one ***18b***

Crystals were grown by dissolution in ethanol and then leaving the solution standing at room temperature.

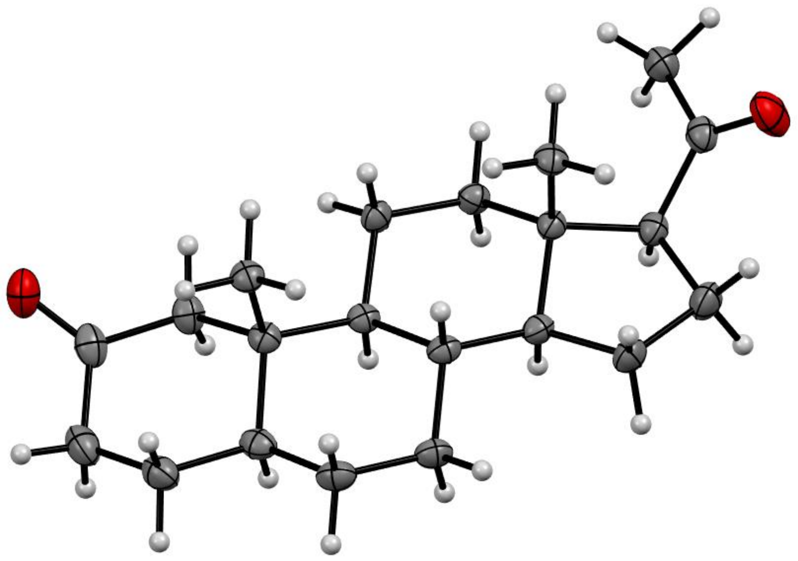

Identification code	bm190623_1_1
Empirical formula	$C_{21}H_{32}O_2$
Formula weight	316.46
Temperature [K]	100.0(1)
Crystal system	orthorhombic
Space group (number)	$P2_12_12_1$ (19)
a [Å]	7.27460(10)
b [Å]	11.40590(10)
c [Å]	21.0775(2)
α [°]	90
β [°]	90
γ [°]	90
Volume [Å^3]	1748.87(3)
Z	4
ρ_{calc} [gcm^{-3}]	1.202
μ [mm^{-1}]	0.576

F(000)	696
Crystal size [mm^3]	0.204×0.125×0.079
Radiation	Cu K_α (λ=1.54184 Å)
2θ range [°]	8.39 to 159.17
Index ranges	$-8 \le h \le 9$<br>$-14 \le k \le 14$<br>$-25 \le l \le 26$
Reflections collected	53391
Independent reflections	3695<br>R_{int} = 0.0447<br>R_{sigma} = 0.0162
Data / Restraints / Parameters	3695/0/211
Goodness-of-fit on F^2	1.037
Final R indexes [$I \geq 2\sigma(I)$]	R_1 = 0.0297<br>wR_2 = 0.0781
Final R indexes [all data]	R_1 = 0.0305<br>wR_2 = 0.0787
Largest peak/hole [eÅ^{-3}]	0.15/-0.16
Flack X parameter	-0.07(6)

10. NMR spectra

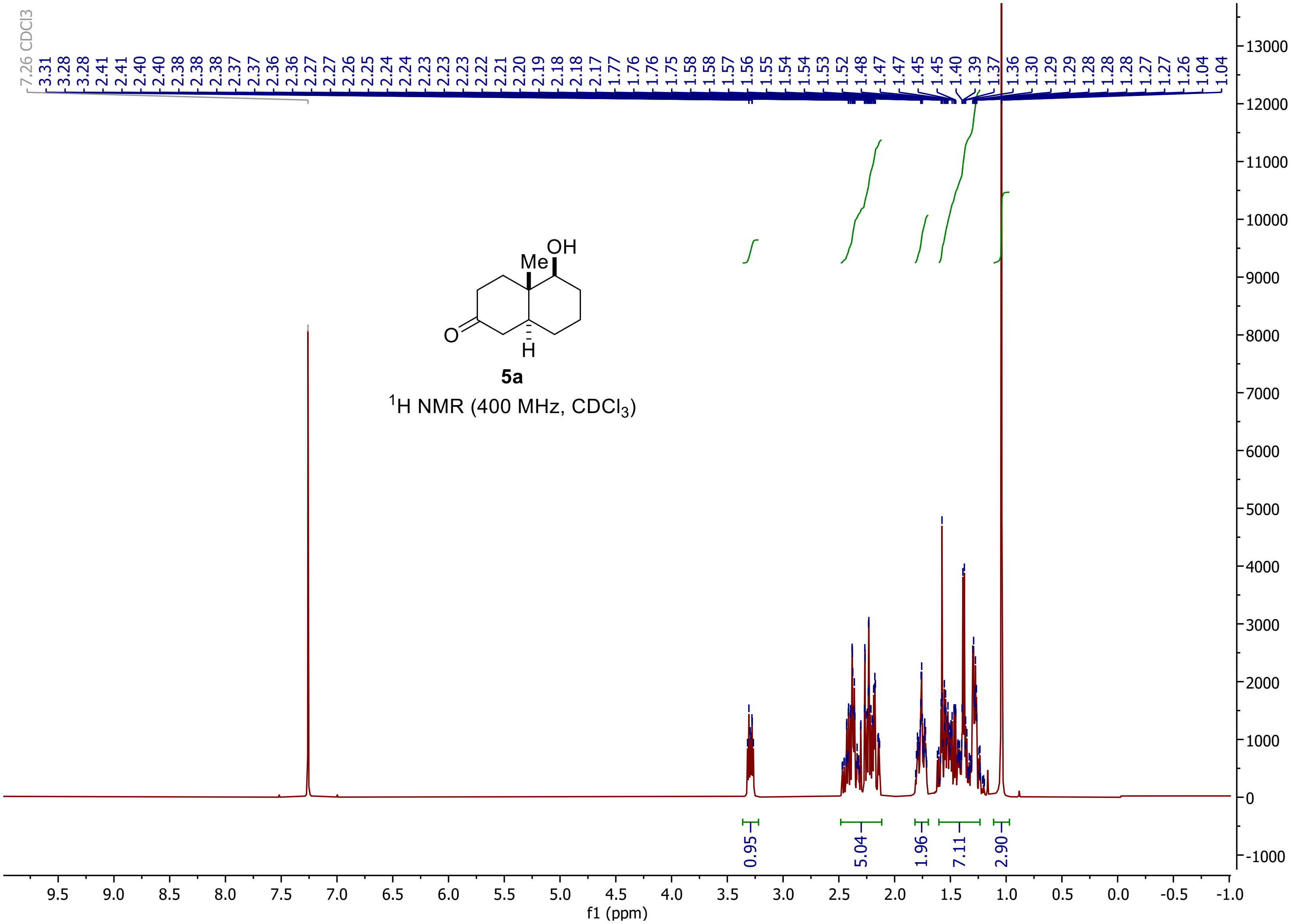

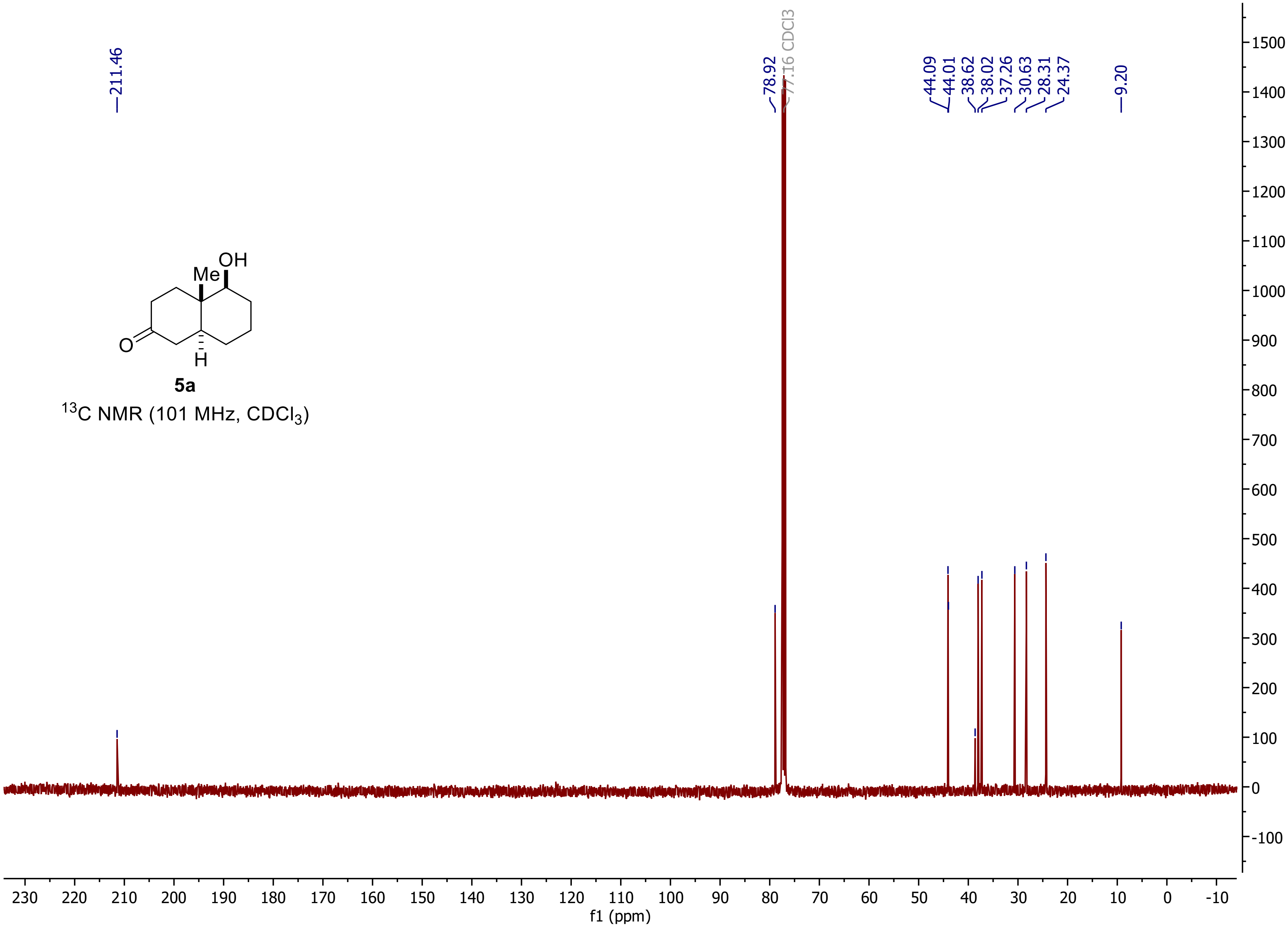

211.46
78.92
77.16 CDCl3
44.09
44.01
38.62
38.02
37.26
30.63
28.31
24.37
9.20
Me
OH
O
H
5a
^{13}C NMR (101 MHz, $CDCl_3$)
f1 (ppm)

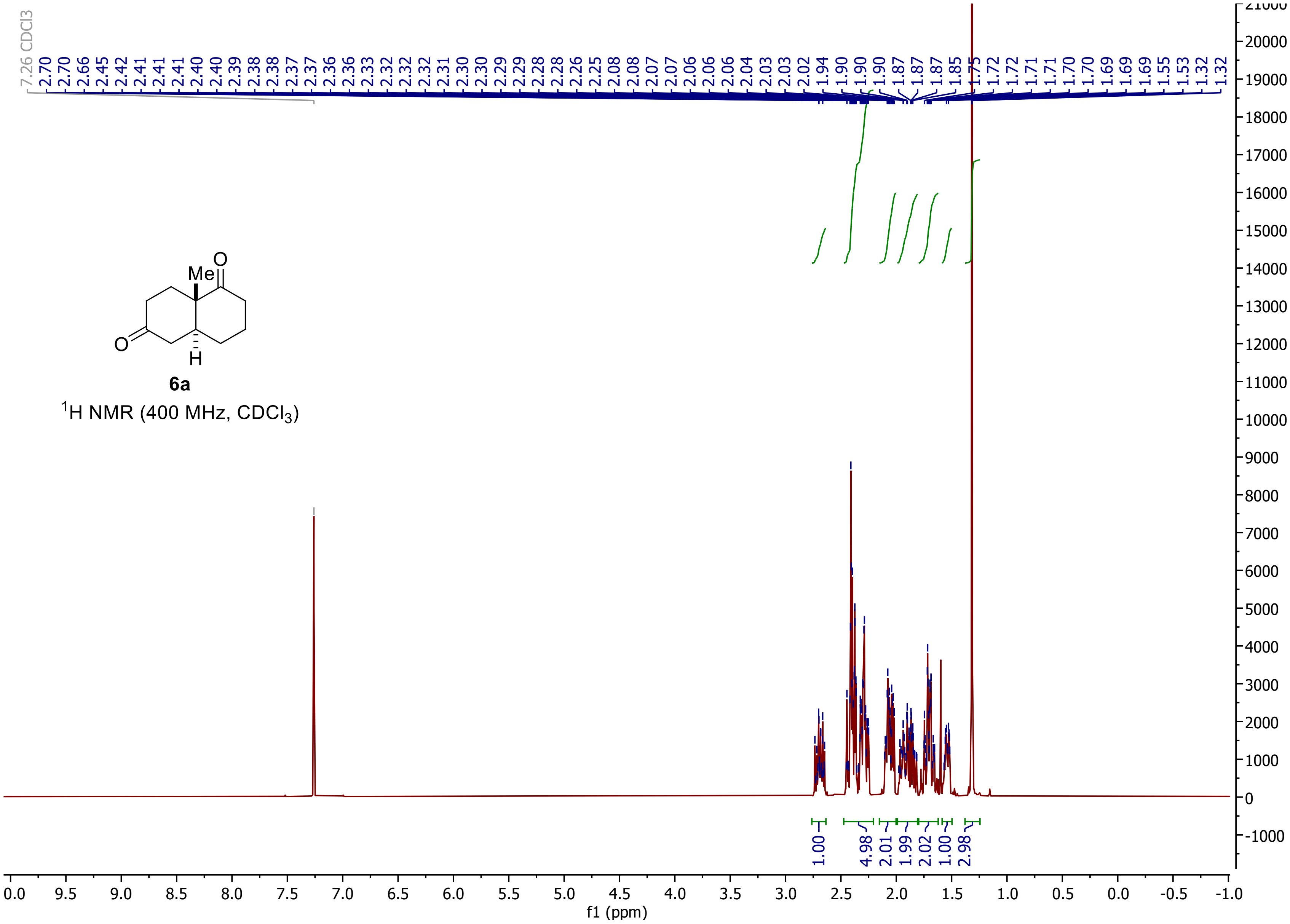
7.26 CDCl3
2.70 2.70 2.66 2.45 2.42 2.41 2.41 2.41 2.40 2.40 2.39 2.38 2.38 2.37 2.37 2.36 2.36 2.33 2.32 2.32 2.32 2.31 2.30 2.30 2.29 2.29 2.28 2.28 2.26 2.25 2.08 2.08 2.07 2.07 2.06 2.06 2.06 2.04 2.03 2.03 2.02 1.94 1.90 1.90 1.90 1.87 1.87 1.87 1.85 1.75 1.72 1.72 1.71 1.71 1.70 1.70 1.69 1.69 1.69 1.55 1.53 1.32 1.32
Me
O
O
H
6a
^{1}H NMR (400 MHz, $CDCl_3$)
1.00
4.98
2.01
1.99
2.02
1.00
2.98
f1 (ppm)

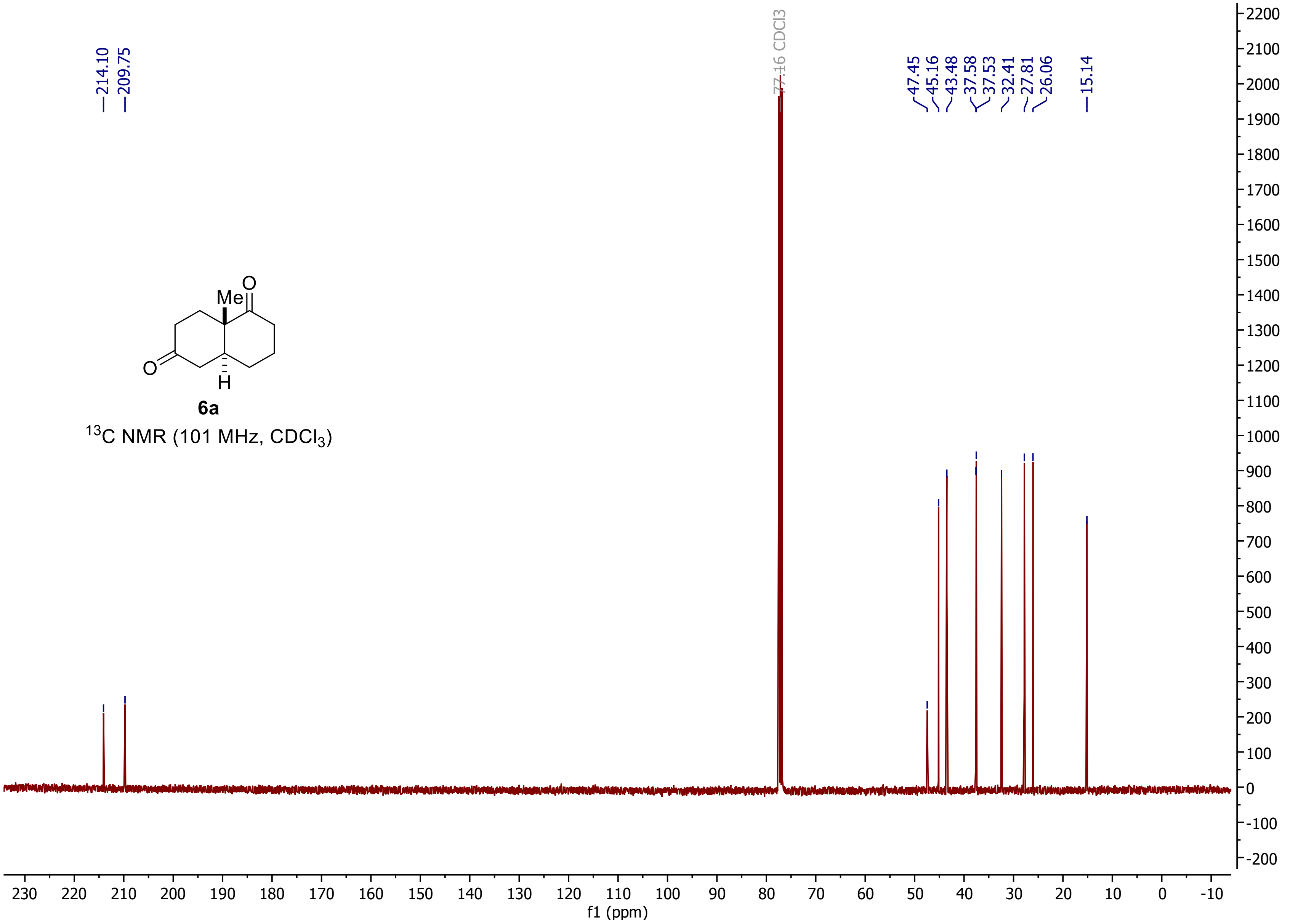

214.10
209.75
77.16 CDCl3
47.45
45.16
43.48
37.58
37.53
32.41
27.81
26.06
15.14
Me
O
O
H
6a
^{13}C NMR (101 MHz, $CDCl_3$)
f1 (ppm)

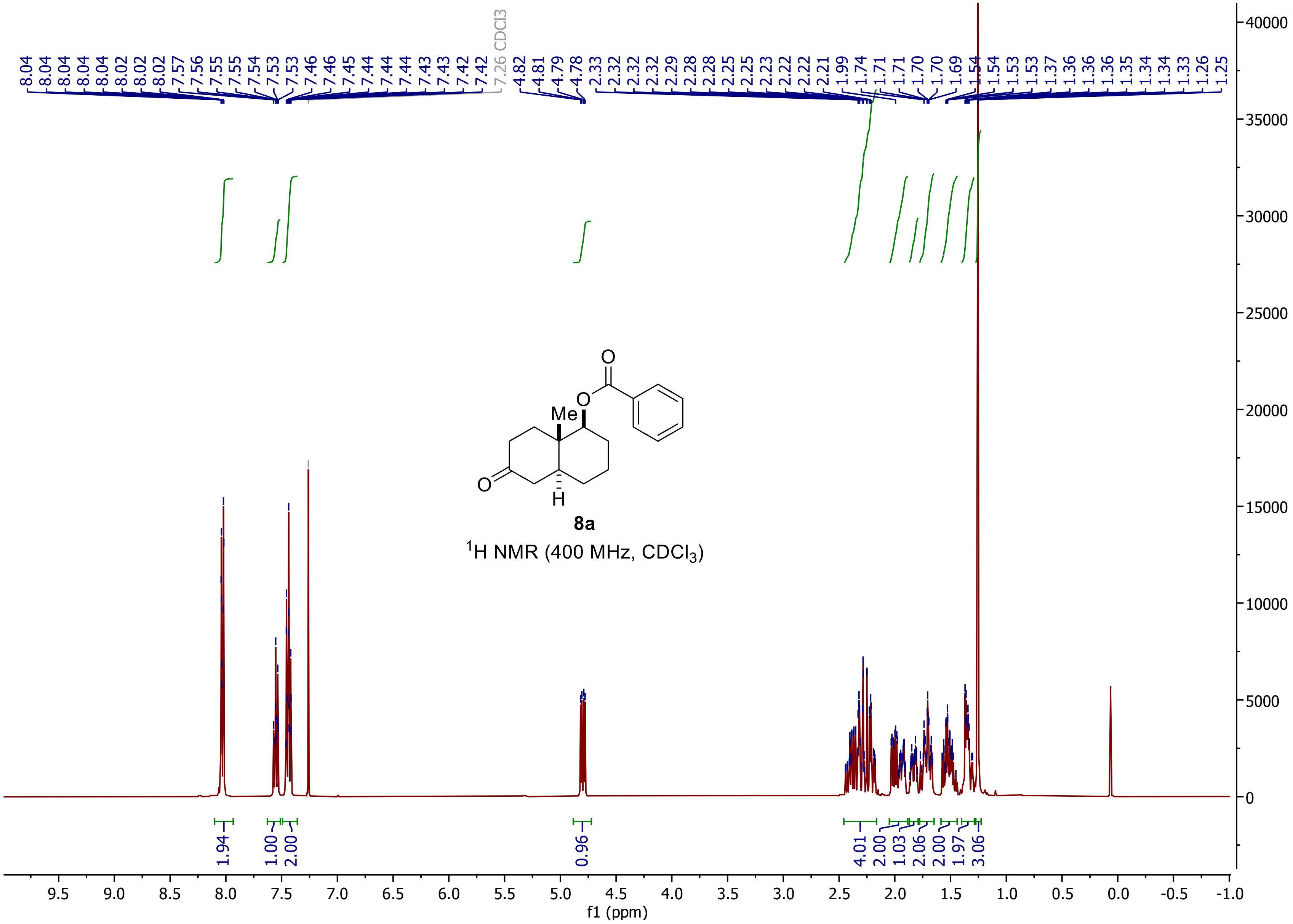

8a
^{1}H NMR (400 MHz, CDCl$_3$)
f1 (ppm)

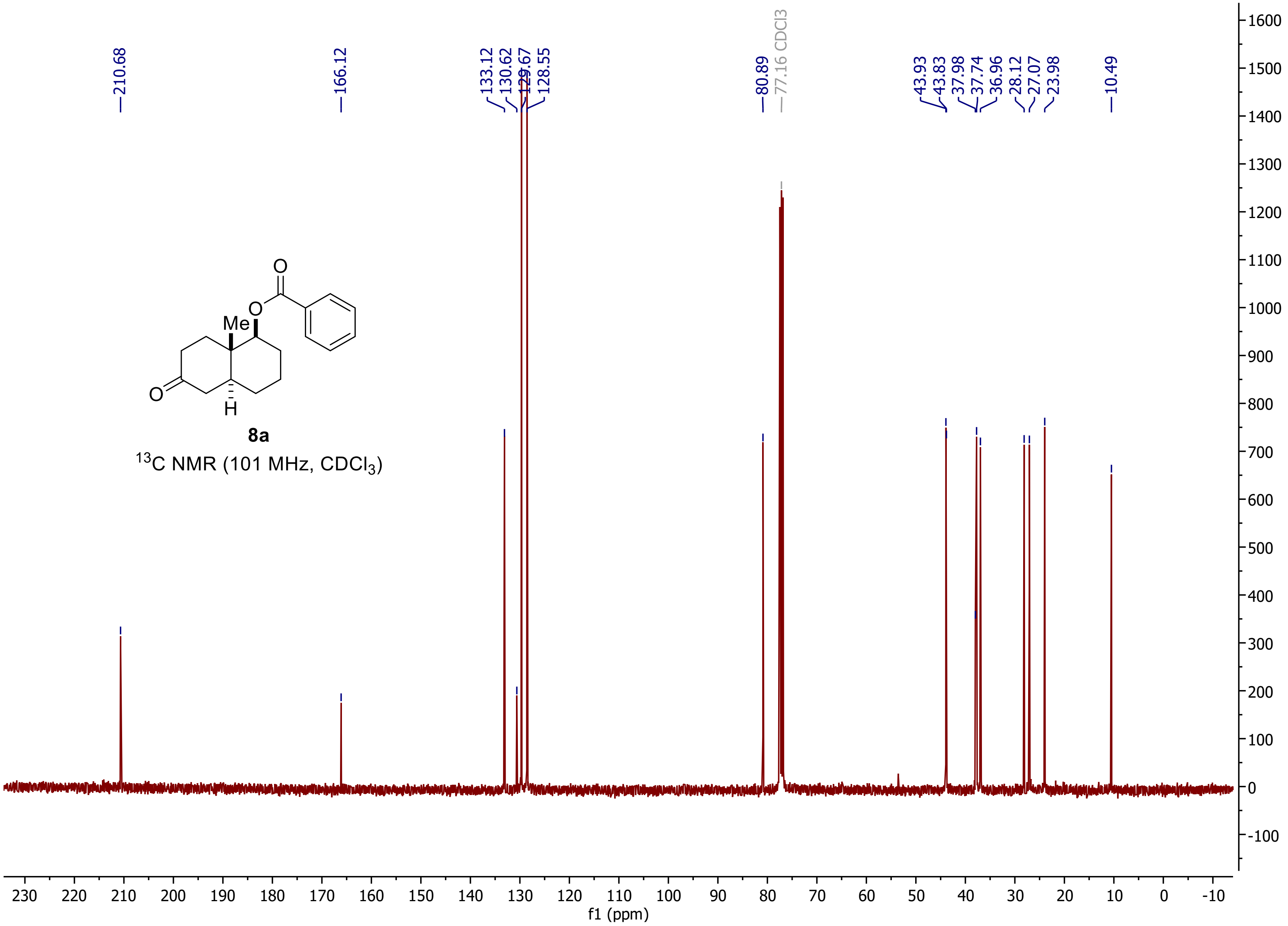
210.68
166.12
133.12
130.62
129.67
128.55
80.89
77.16 CDCl3
43.93
43.83
37.98
37.74
36.96
28.12
27.07
23.98
10.49
Me
O
H
8a
13C NMR (101 MHz, CDCl3)
f1 (ppm)

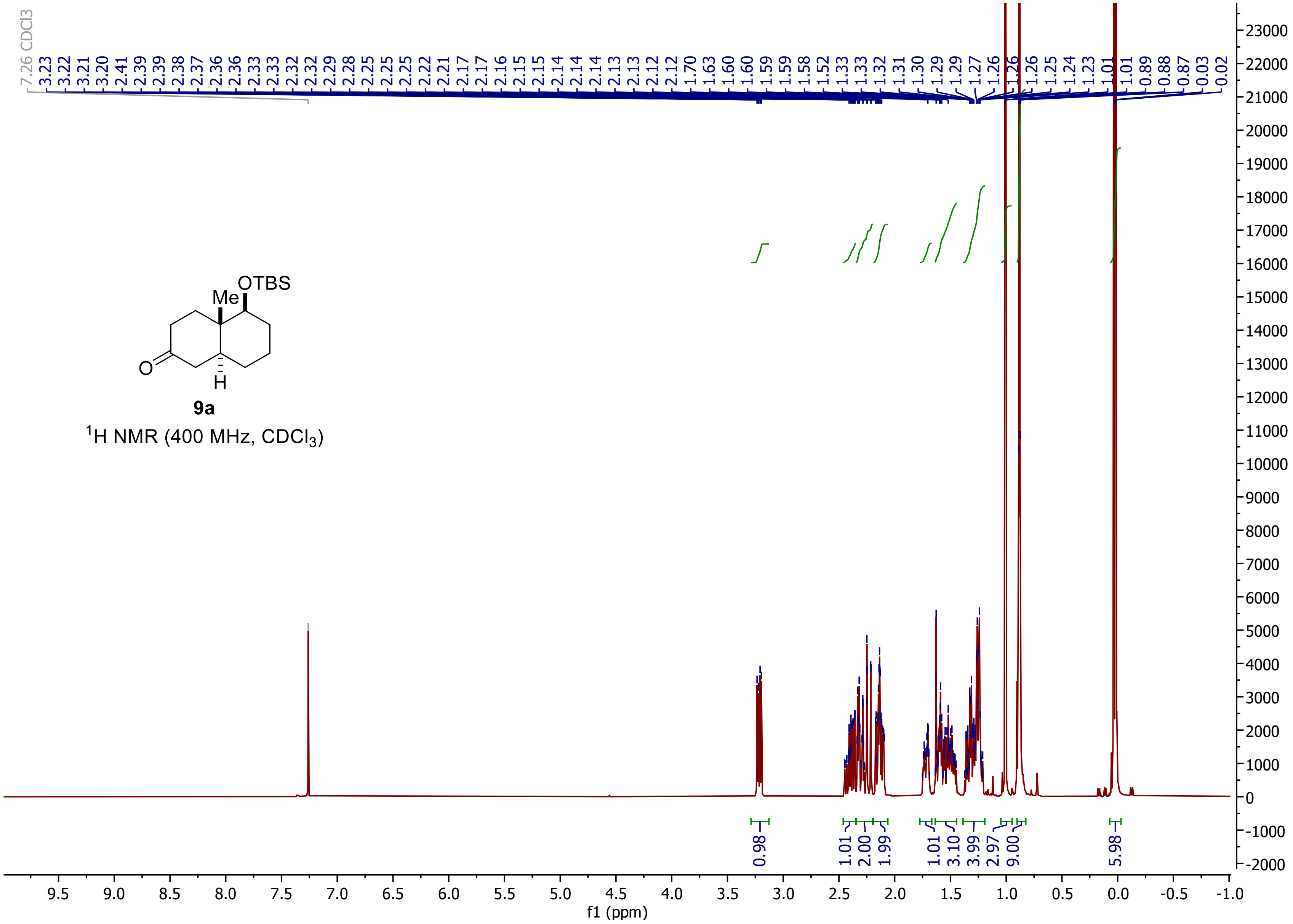
OTBS
Me
O
H
9a
^{1}H NMR (400 MHz, CDCl$_3$)
f1 (ppm)

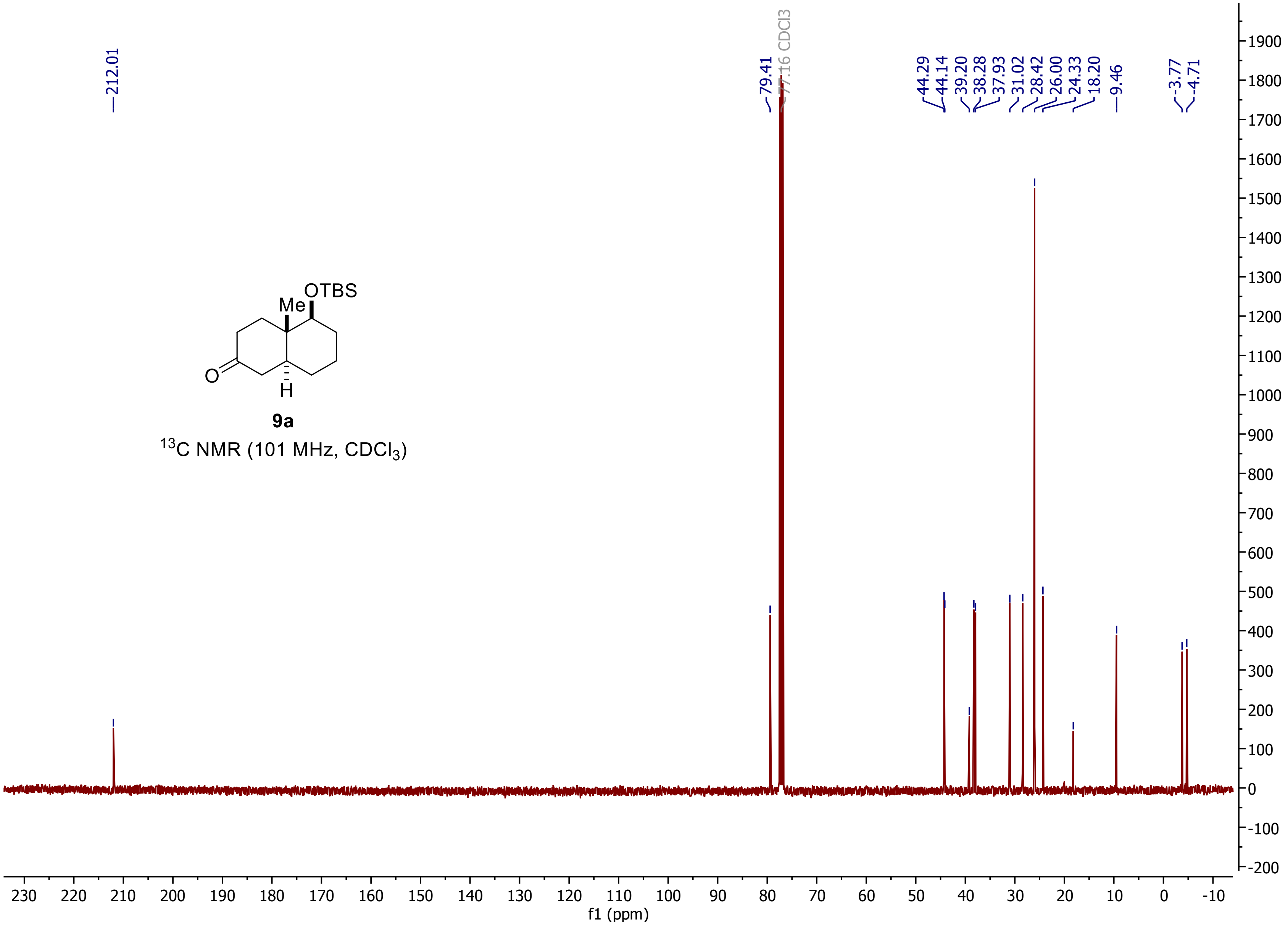

212.01
79.41
77.16 CDCl3
44.29
44.14
39.20
38.28
37.93
31.02
28.42
26.00
24.33
18.20
9.46
-3.77
-4.71
OTBS
Me
O
H
9a
^{13}C NMR (101 MHz, $CDCl_3$)
f1 (ppm)

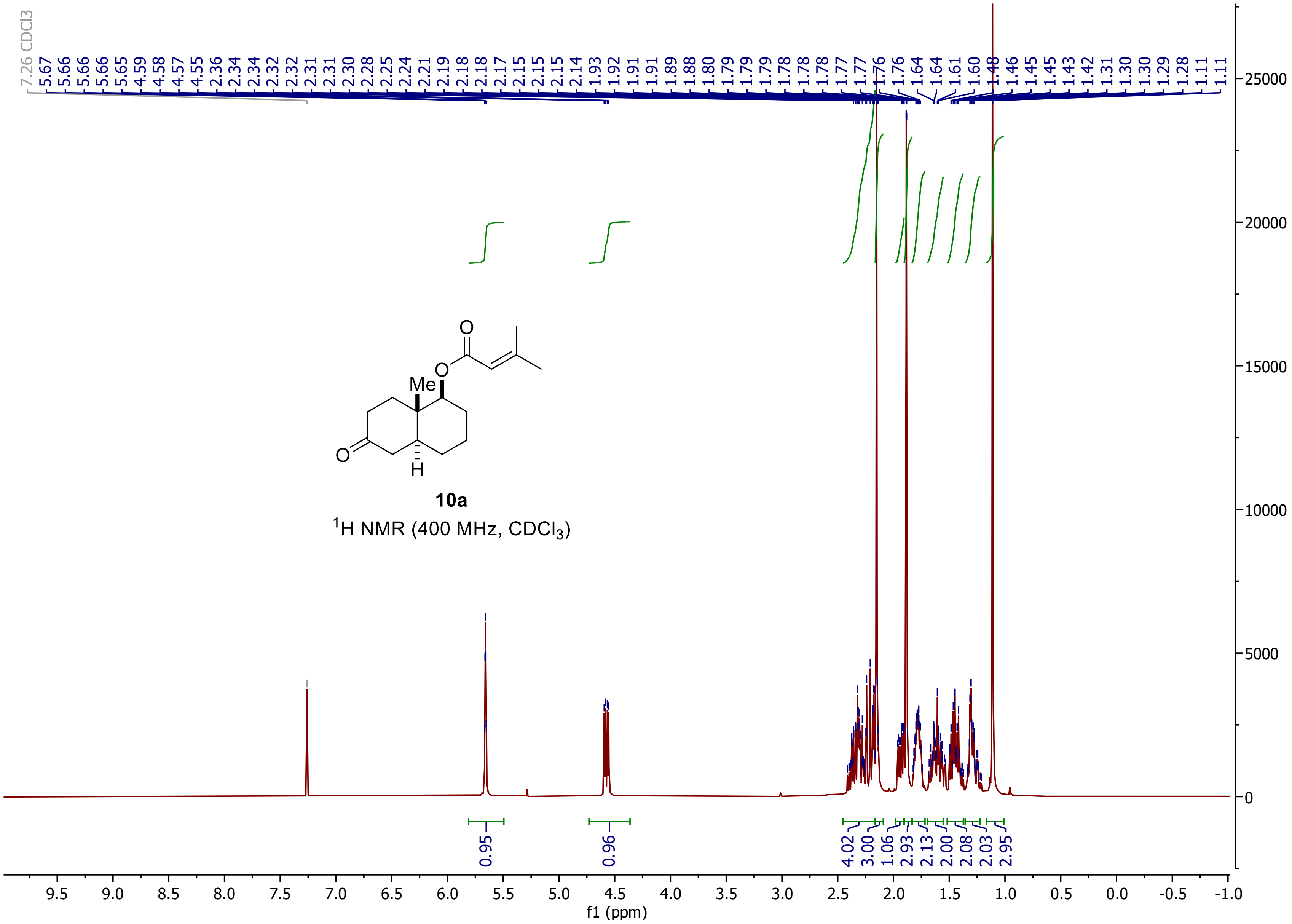

10a

^{1}H NMR (400 MHz, $CDCl_3$)

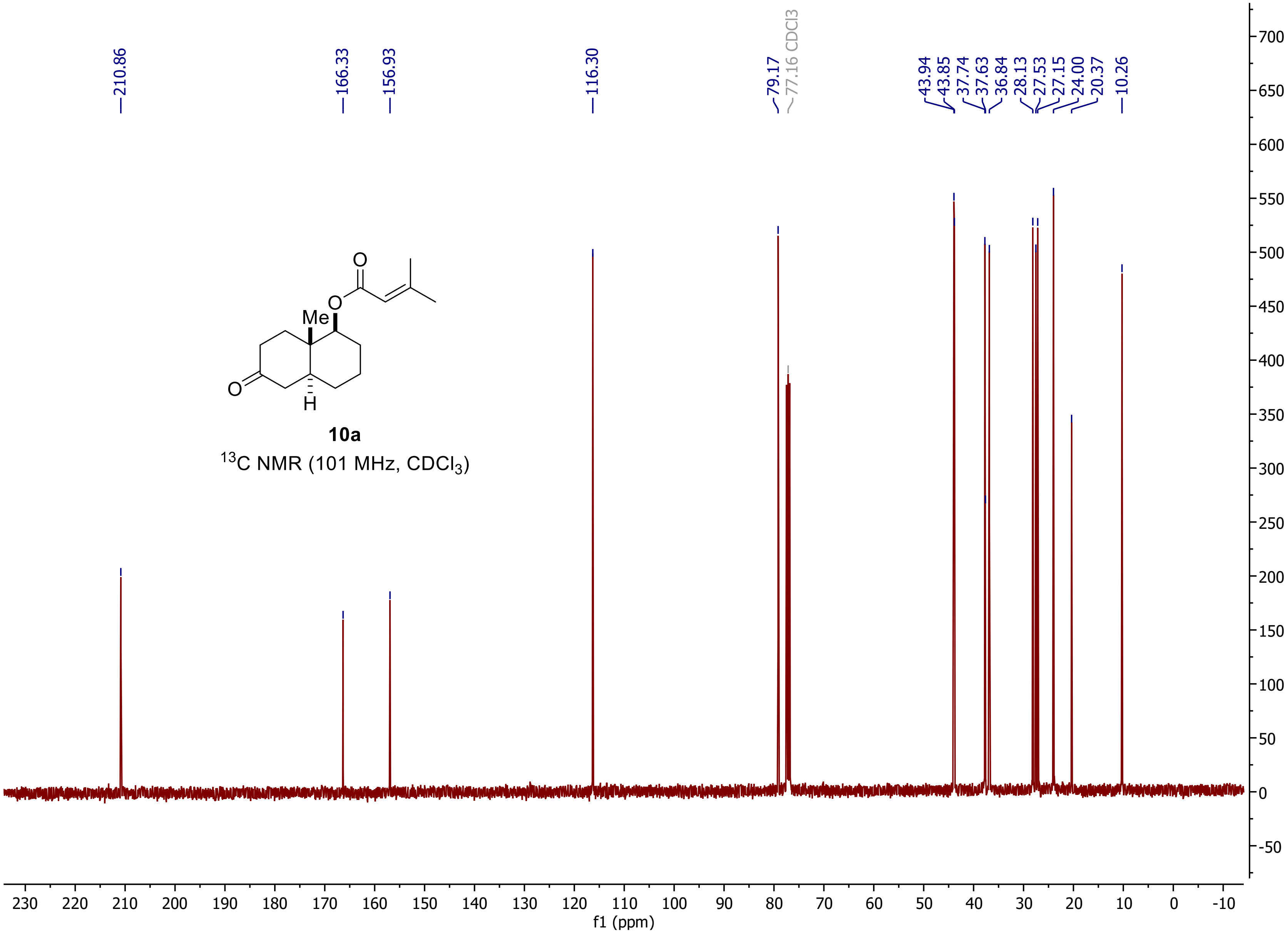
210.86
166.33
156.93
116.30
79.17
77.16 CDCl3
43.94
43.85
37.74
37.63
36.84
28.13
27.53
27.15
24.00
20.37
10.26
Me
O
H
10a
13C NMR (101 MHz, CDCl3)
f1 (ppm)

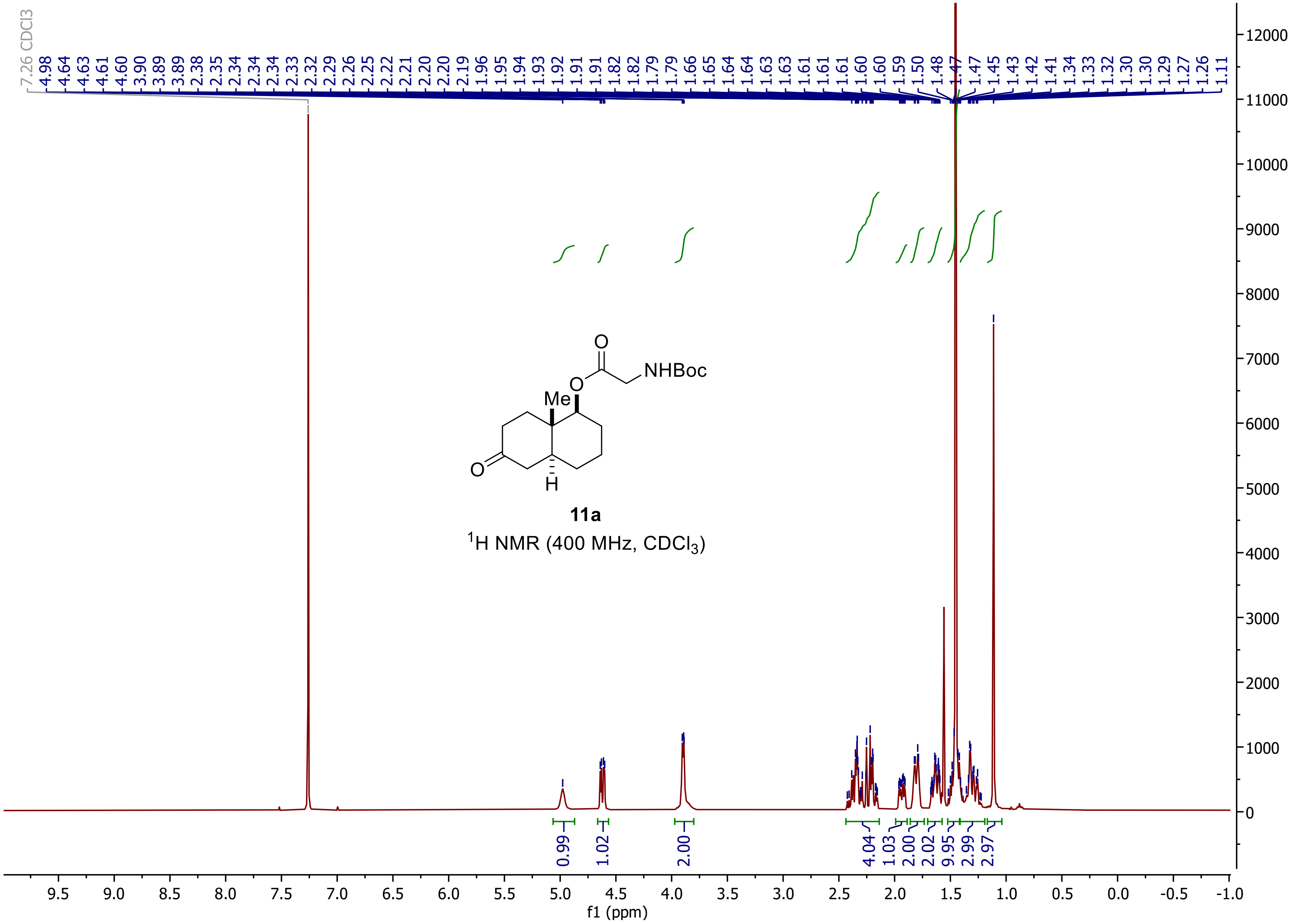
7.26 CDCl3
4.98 4.64 4.63 4.61 4.60 3.90 3.89 3.89 2.38 2.35 2.34 2.34 2.34 2.33 2.32 2.29 2.26 2.25 2.22 2.21 2.20 2.20 2.19 1.96 1.95 1.94 1.93 1.92 1.91 1.91 1.82 1.82 1.79 1.79 1.66 1.65 1.64 1.64 1.63 1.63 1.61 1.61 1.61 1.60 1.60 1.59 1.50 1.48 1.47 1.47 1.45 1.43 1.42 1.41 1.34 1.33 1.32 1.30 1.30 1.29 1.27 1.26 1.11
O
NHBoc
O
Me
O
H
11a
^{1}H NMR (400 MHz, $CDCl_3$)
0.99 1.02 2.00 4.04 1.03 2.00 2.02 9.95 2.99 2.97
f1 (ppm)

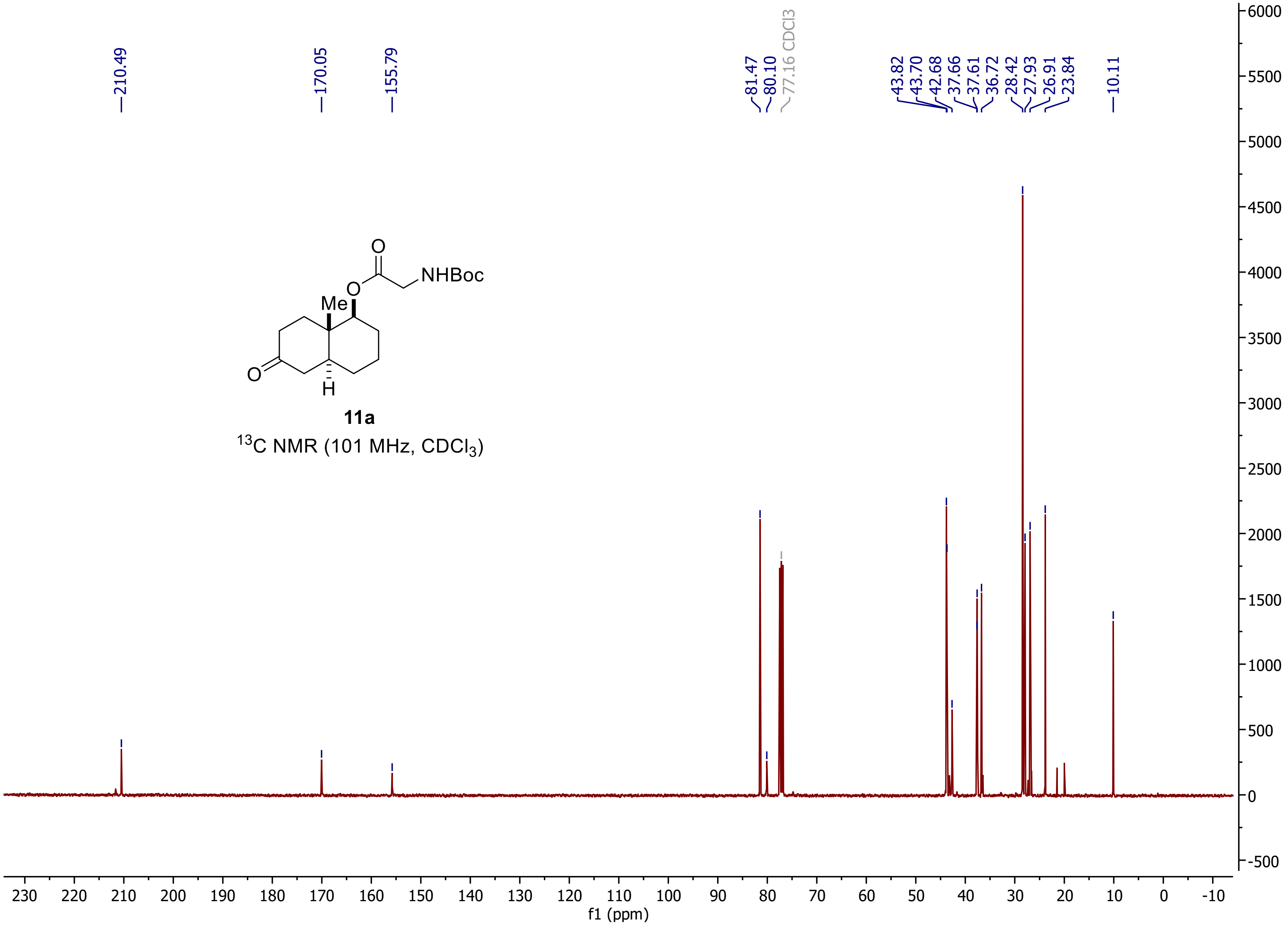
210.49
170.05
155.79
81.47
80.10
77.16 CDCl3
43.82
43.70
42.68
37.66
37.61
36.72
28.42
27.93
26.91
23.84
10.11
NHBoc
Me
O
H
11a
^{13}C NMR (101 MHz, $CDCl_3$)
f1 (ppm)

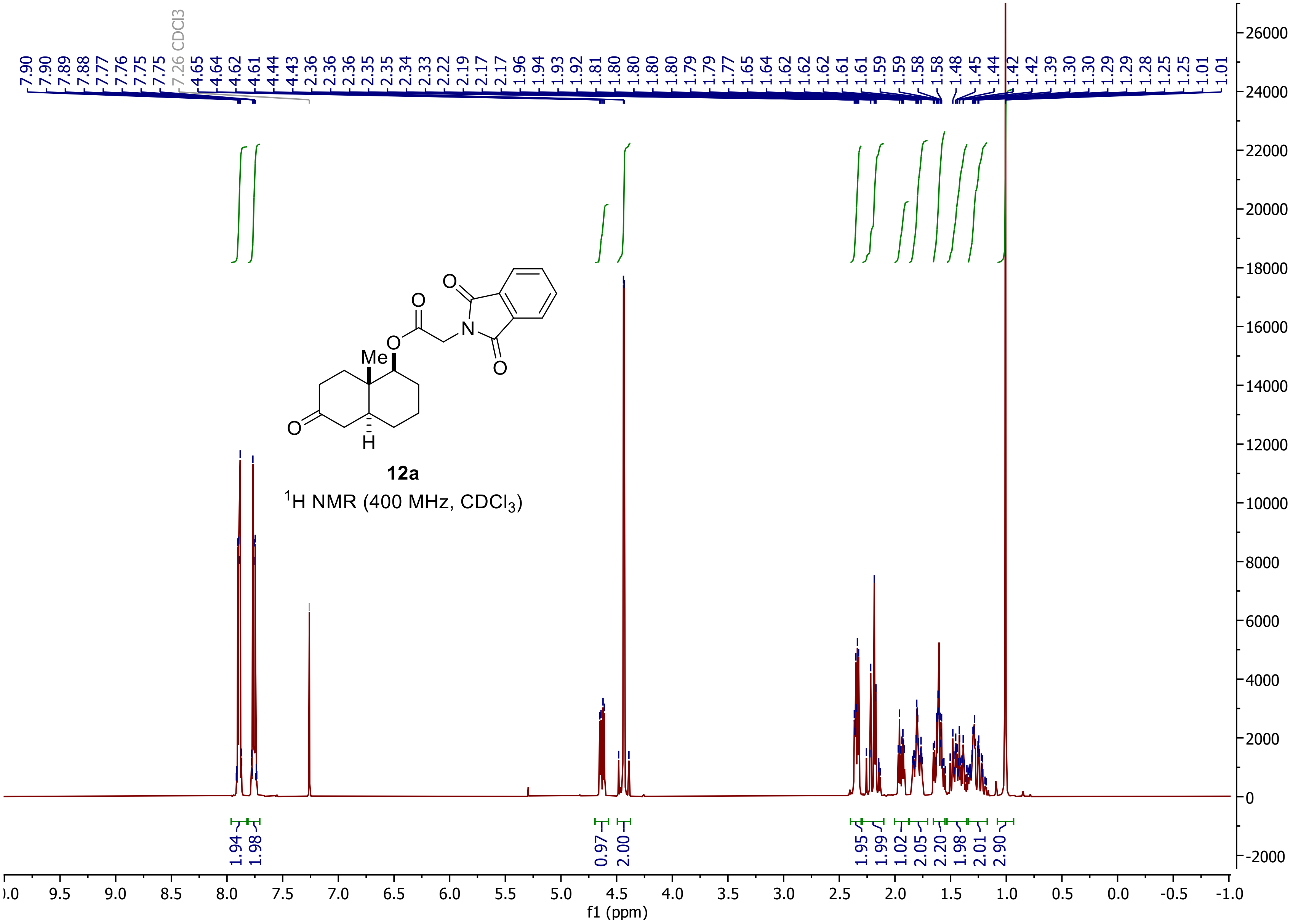

7.90 7.90 7.89 7.88 7.77 7.76 7.75 7.75 7.26 CDCl3 4.65 4.64 4.62 4.61 4.44 4.43 2.36 2.36 2.36 2.35 2.35 2.34 2.33 2.22 2.19 2.17 2.17 1.96 1.94 1.93 1.92 1.82 1.81 1.80 1.80 1.80 1.80 1.79 1.79 1.77 1.65 1.64 1.62 1.62 1.62 1.61 1.61 1.59 1.59 1.58 1.58 1.48 1.45 1.44 1.42 1.42 1.39 1.30 1.30 1.29 1.29 1.28 1.25 1.25 1.01 1.01
O
O
O
N
O
Me
O
H
12a
^{1}H NMR (400 MHz, $CDCl_3$)
1.94 1.98 0.97 2.00 1.95 1.99 1.02 2.05 2.20 1.98 2.01 2.90
f1 (ppm)

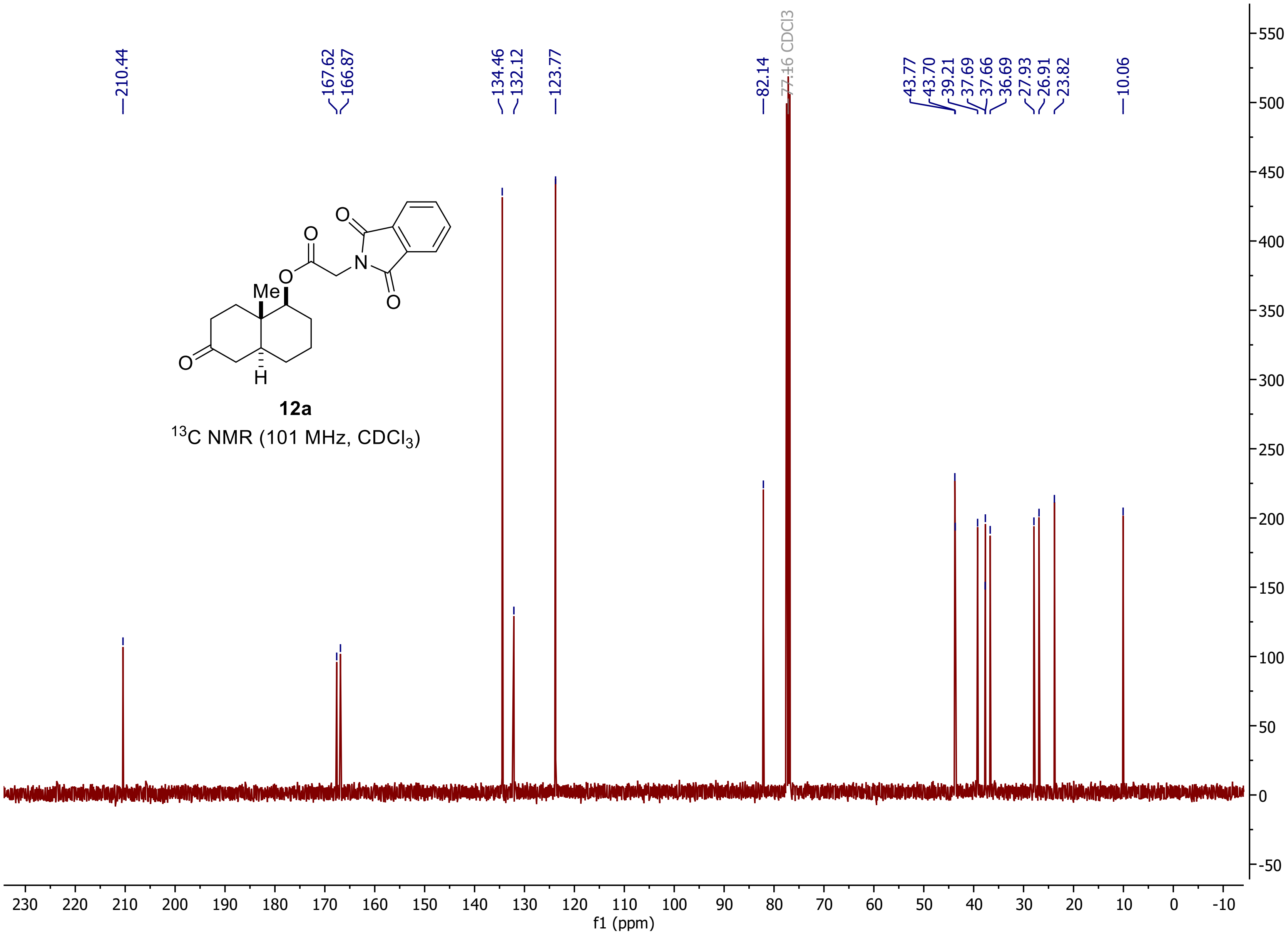

210.44
167.62
166.87
134.46
132.12
123.77
82.14
77.16 CDCl3
43.77
43.70
39.21
37.69
37.66
36.69
27.93
26.91
23.82
10.06
O
O
O
N
O
O
Me
O
H
12a
^{13}C NMR (101 MHz, $CDCl_3$)
f1 (ppm)

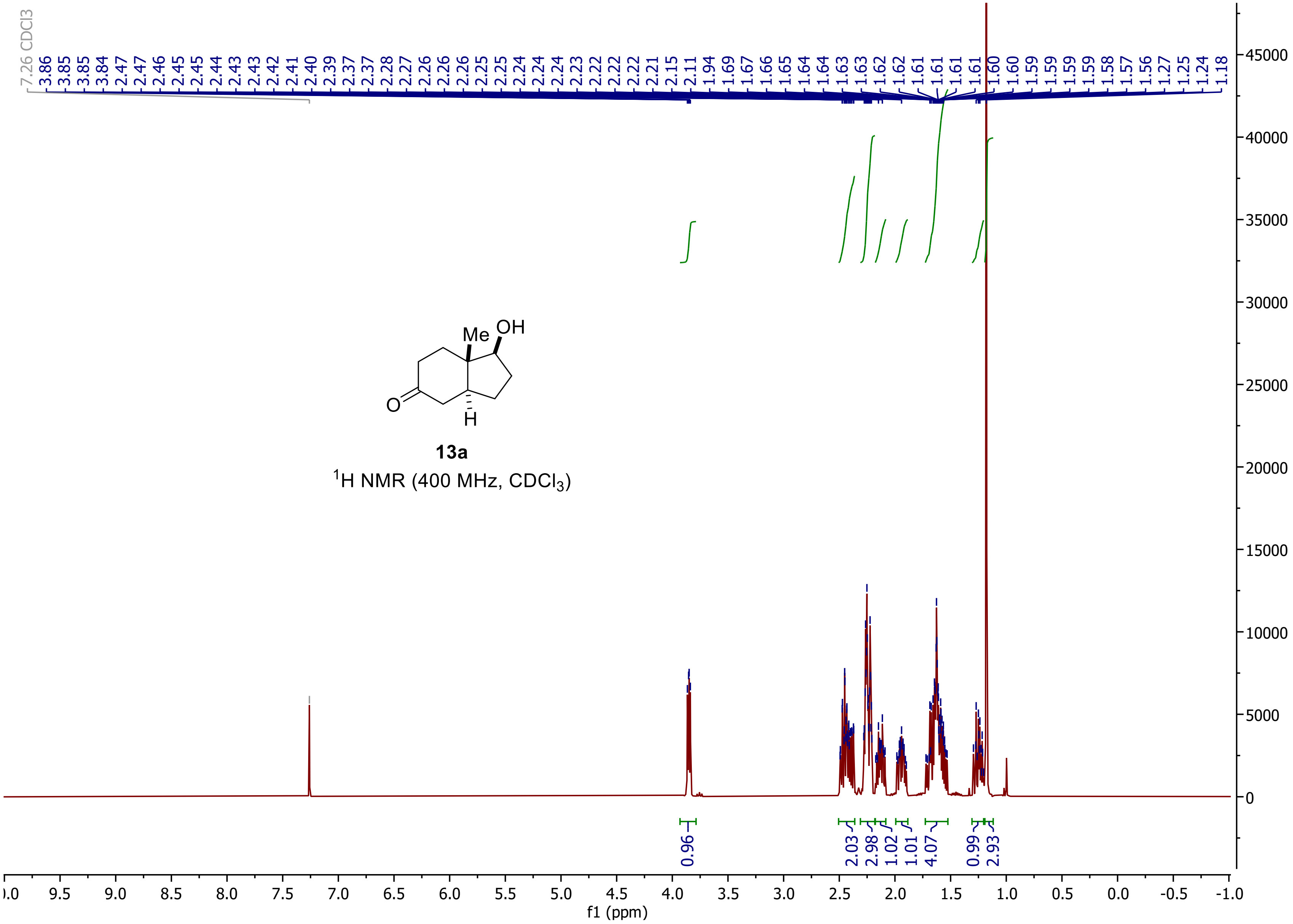
7.26 CDCl3
3.86 3.85 3.85 3.84 2.47 2.47 2.46 2.45 2.45 2.44 2.43 2.43 2.42 2.41 2.40 2.39 2.37 2.37 2.28 2.27 2.26 2.26 2.26 2.25 2.25 2.24 2.24 2.24 2.23 2.22 2.22 2.22 2.21 2.15 2.11 1.94 1.69 1.67 1.66 1.65 1.64 1.64 1.63 1.63 1.62 1.62 1.61 1.61 1.61 1.61 1.60 1.60 1.59 1.59 1.59 1.58 1.57 1.56 1.27 1.25 1.24 1.18
Me
OH
O
H
13a
^{1}H NMR (400 MHz, CDCl$_3$)
0.96
2.03
2.98
1.02
1.01
4.07
0.99
2.93
f1 (ppm)

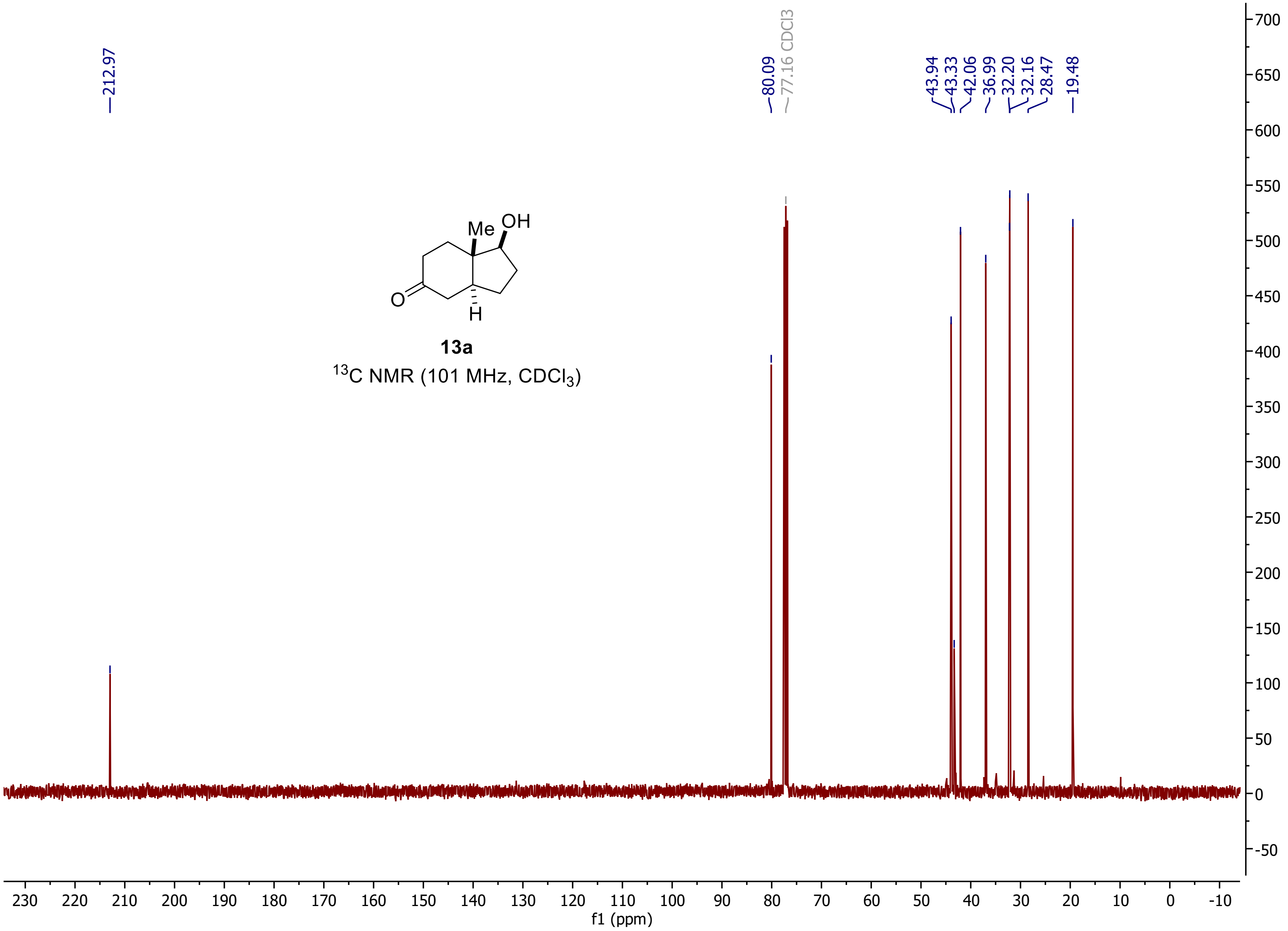

13a

^{13}C NMR (101 MHz, $CDCl_3$)

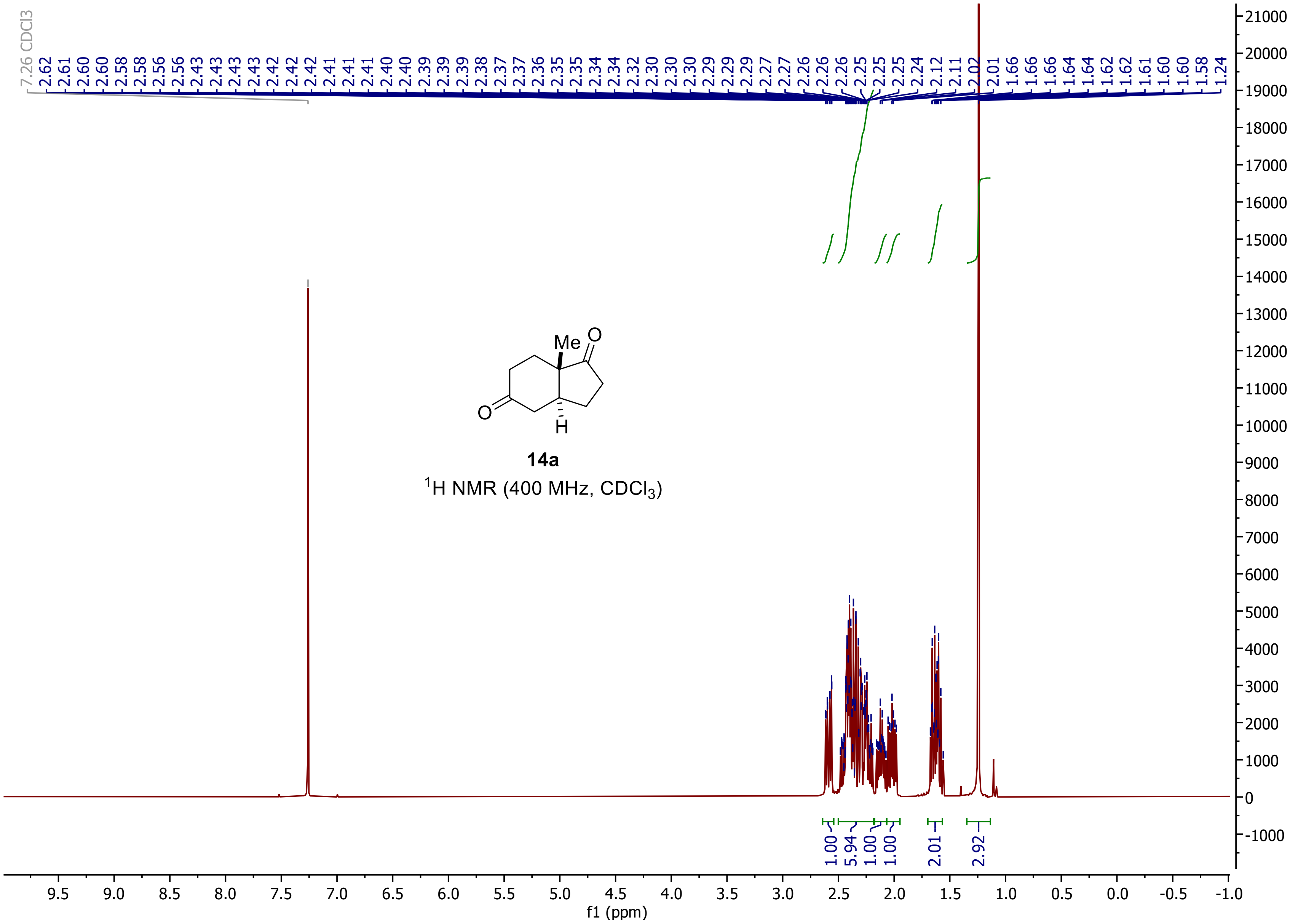

14a

^{1}H NMR (400 MHz, $CDCl_3$)

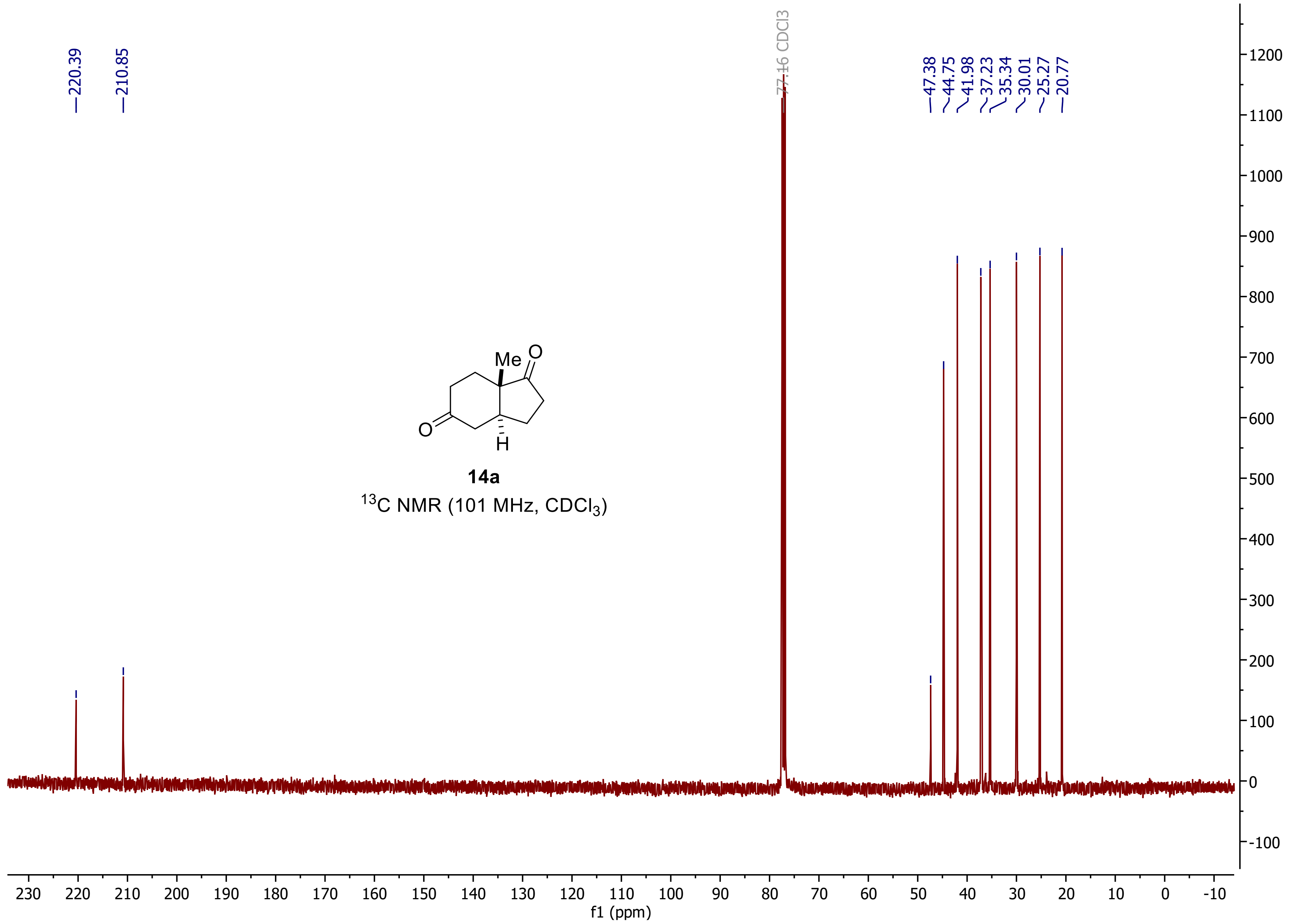
220.39
210.85
77.16 CDCl3
47.38
44.75
41.98
37.23
35.34
30.01
25.27
20.77
Me
O
O
H
14a
13C NMR (101 MHz, CDCl3)
f1 (ppm)

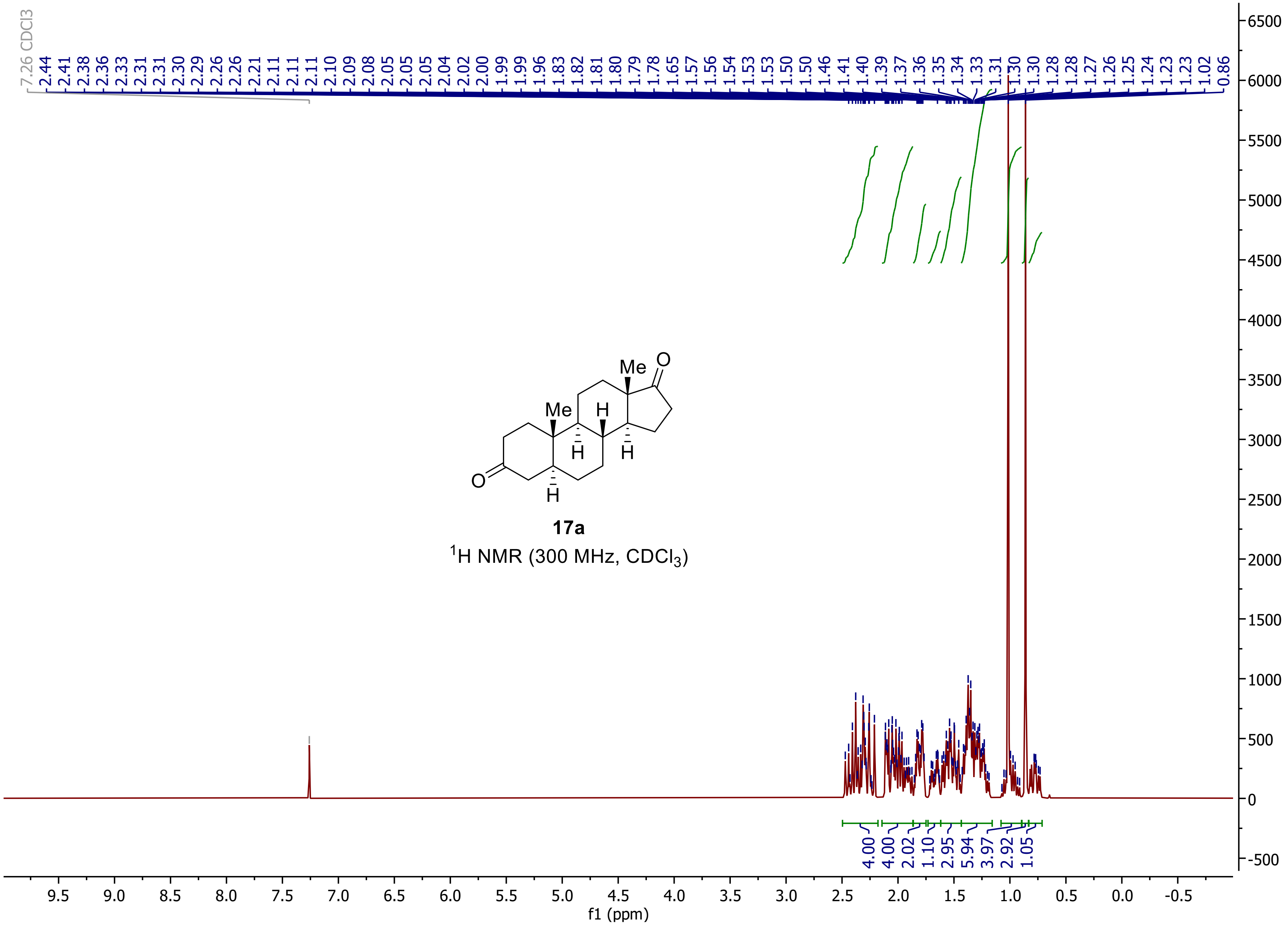
7.26 CDCl3
2.44 2.41 2.38 2.36 2.33 2.31 2.31 2.30 2.29 2.26 2.26 2.21 2.11 2.11 2.11 2.10 2.09 2.08 2.05 2.05 2.05 2.04 2.02 2.00 1.99 1.99 1.96 1.83 1.82 1.81 1.80 1.79 1.78 1.65 1.57 1.56 1.54 1.53 1.53 1.50 1.50 1.46 1.41 1.40 1.39 1.37 1.36 1.35 1.34 1.33 1.31 1.30 1.30 1.28 1.28 1.27 1.26 1.25 1.24 1.23 1.23 1.02 0.86
Me
O
H
17a
^{1}H NMR (300 MHz, CDCl$_3$)
4.00 4.00 2.02 1.10 2.95 5.94 3.97 2.92 1.05
f1 (ppm)

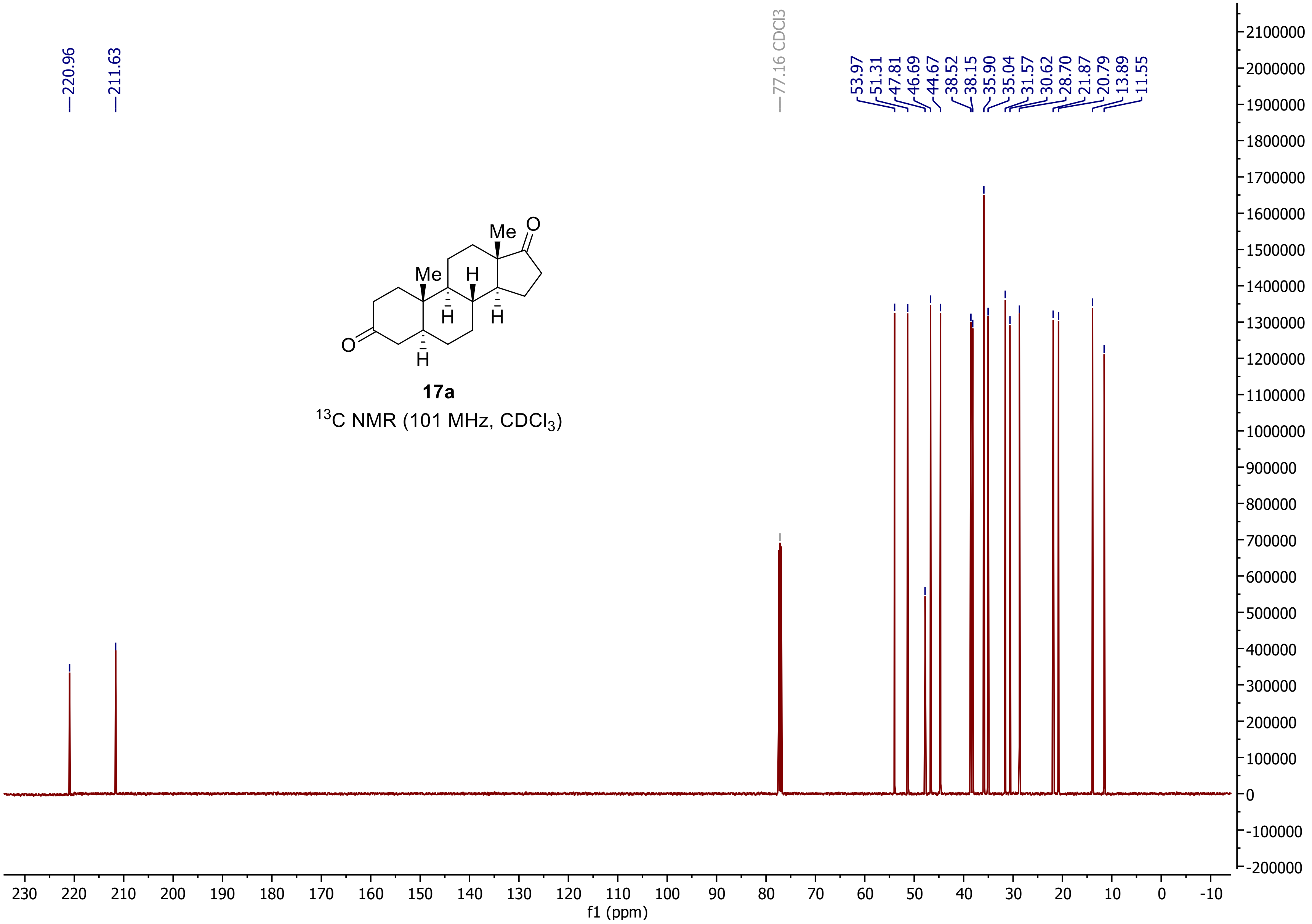

17a

^{13}C NMR (101 MHz, $CDCl_3$)

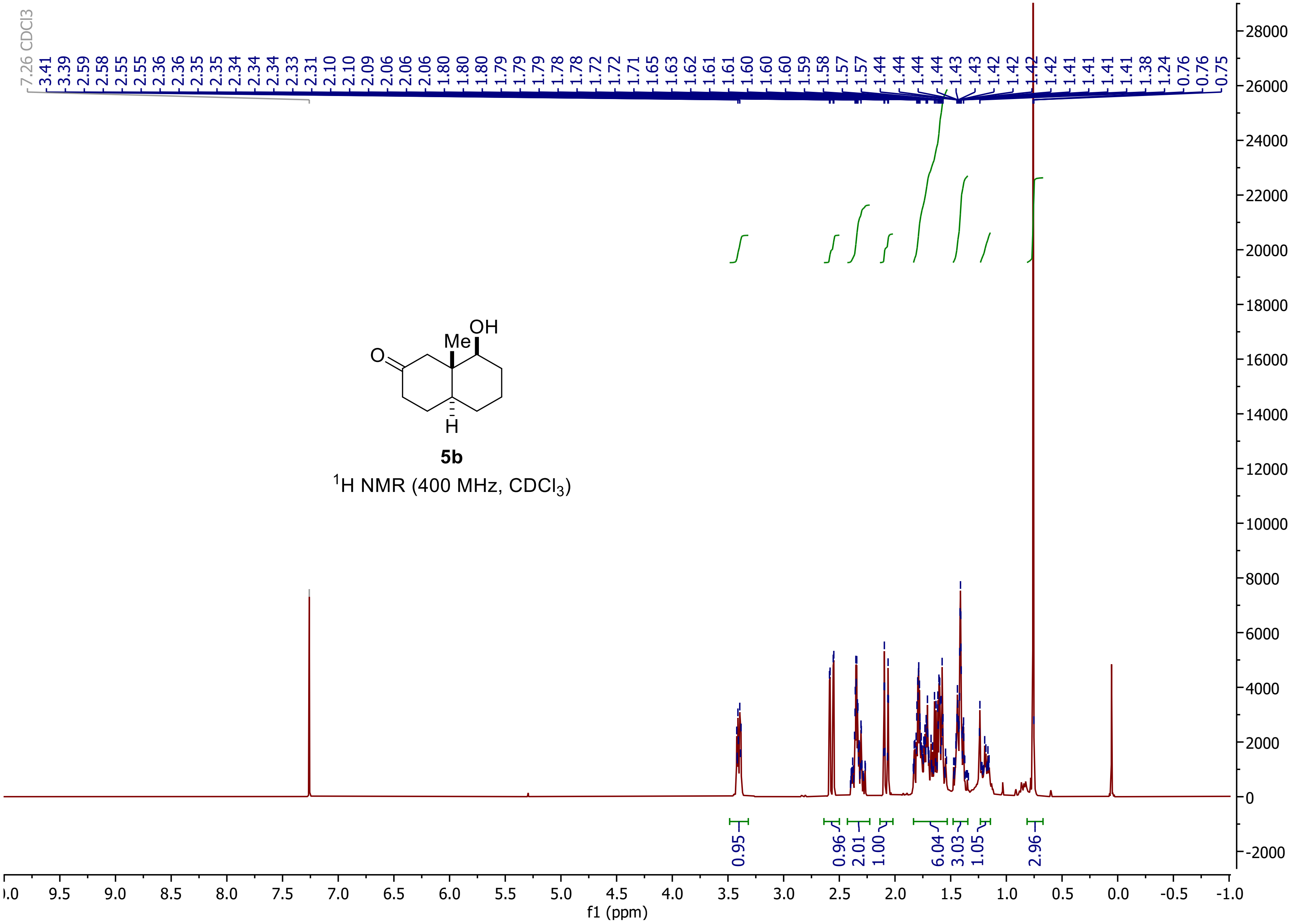

5b

^{1}H NMR (400 MHz, $CDCl_3$)

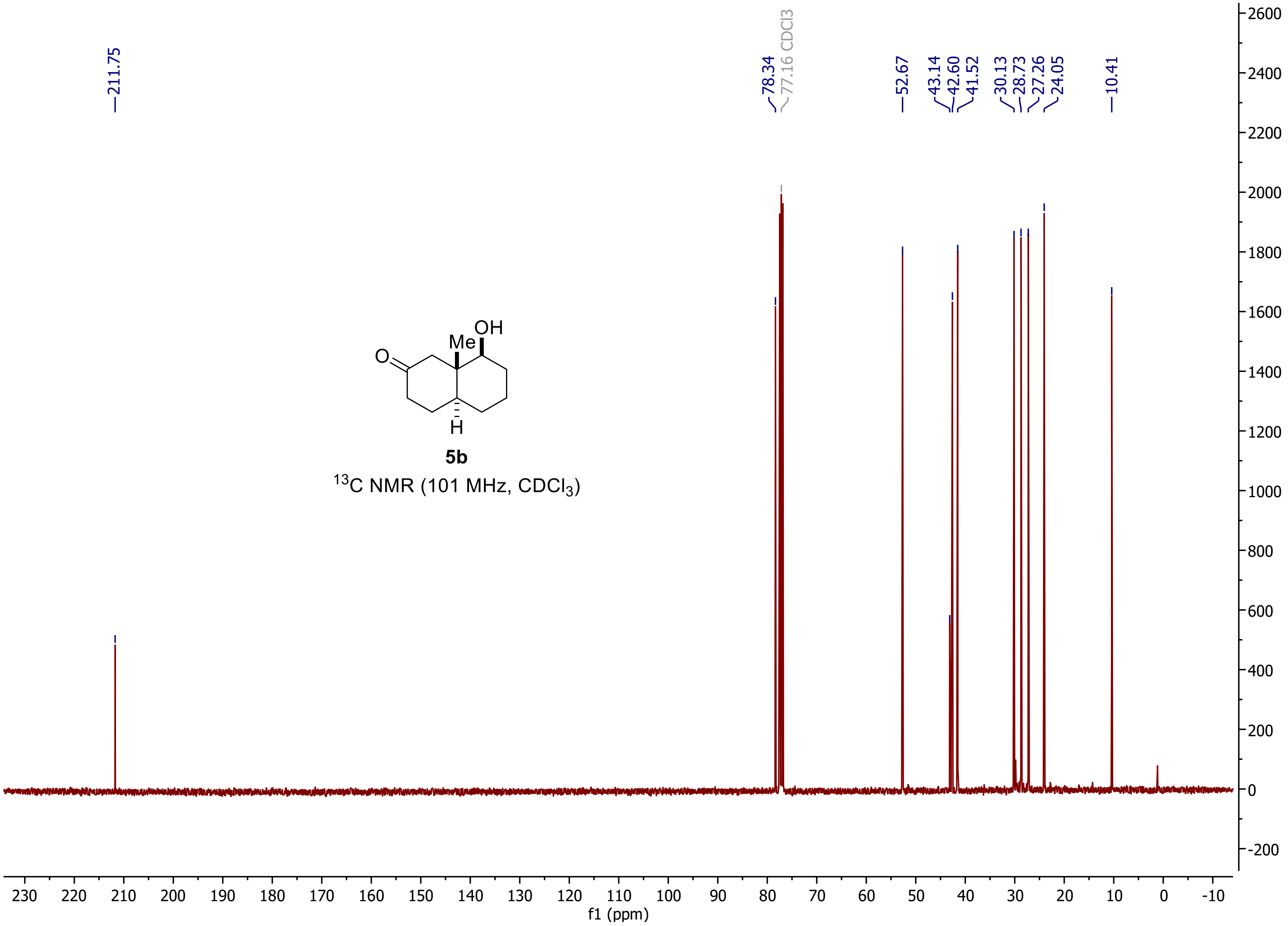

5b

^{13}C NMR (101 MHz, $CDCl_3$)

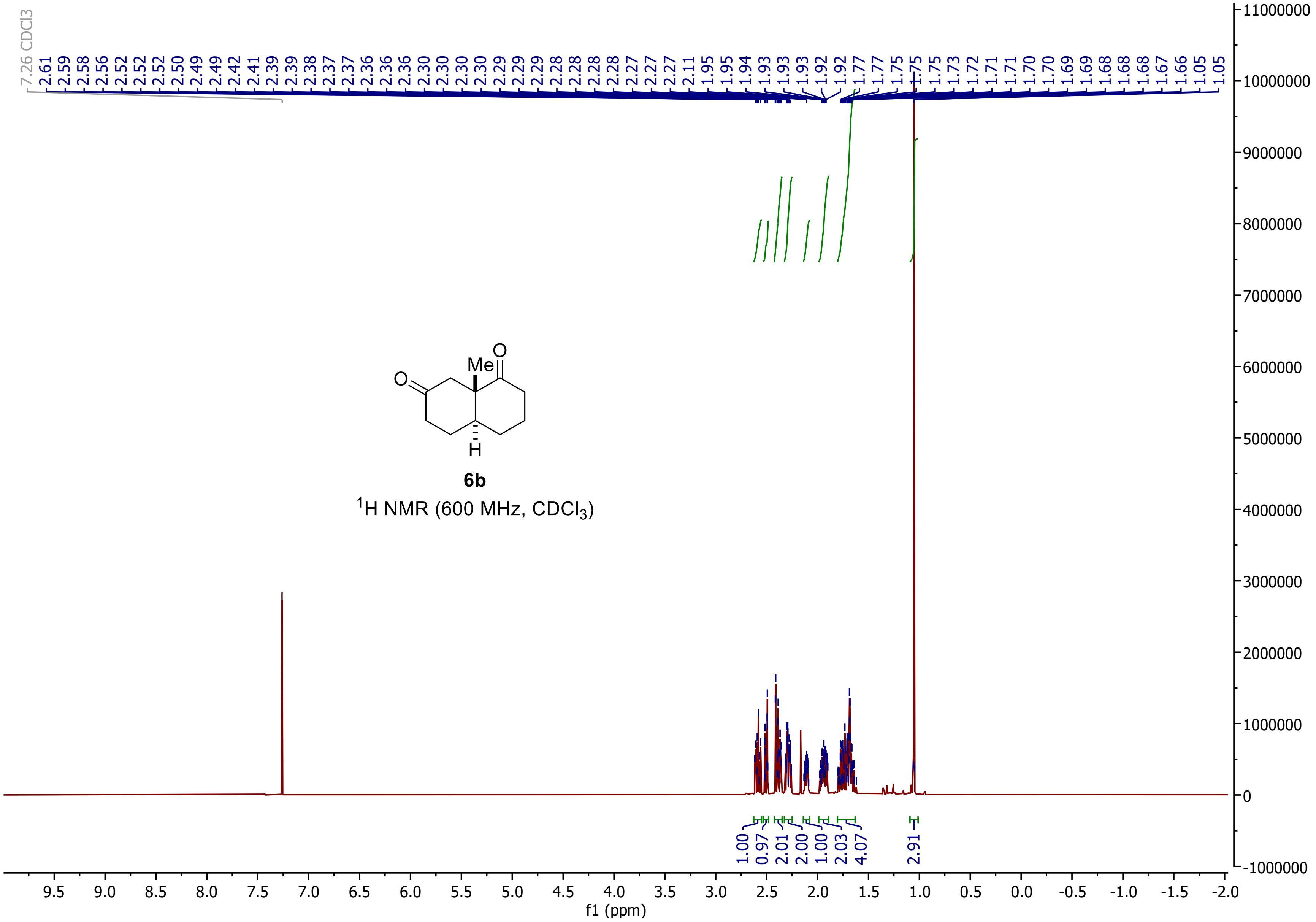

6b

^1H NMR (600 MHz, $CDCl_3$)

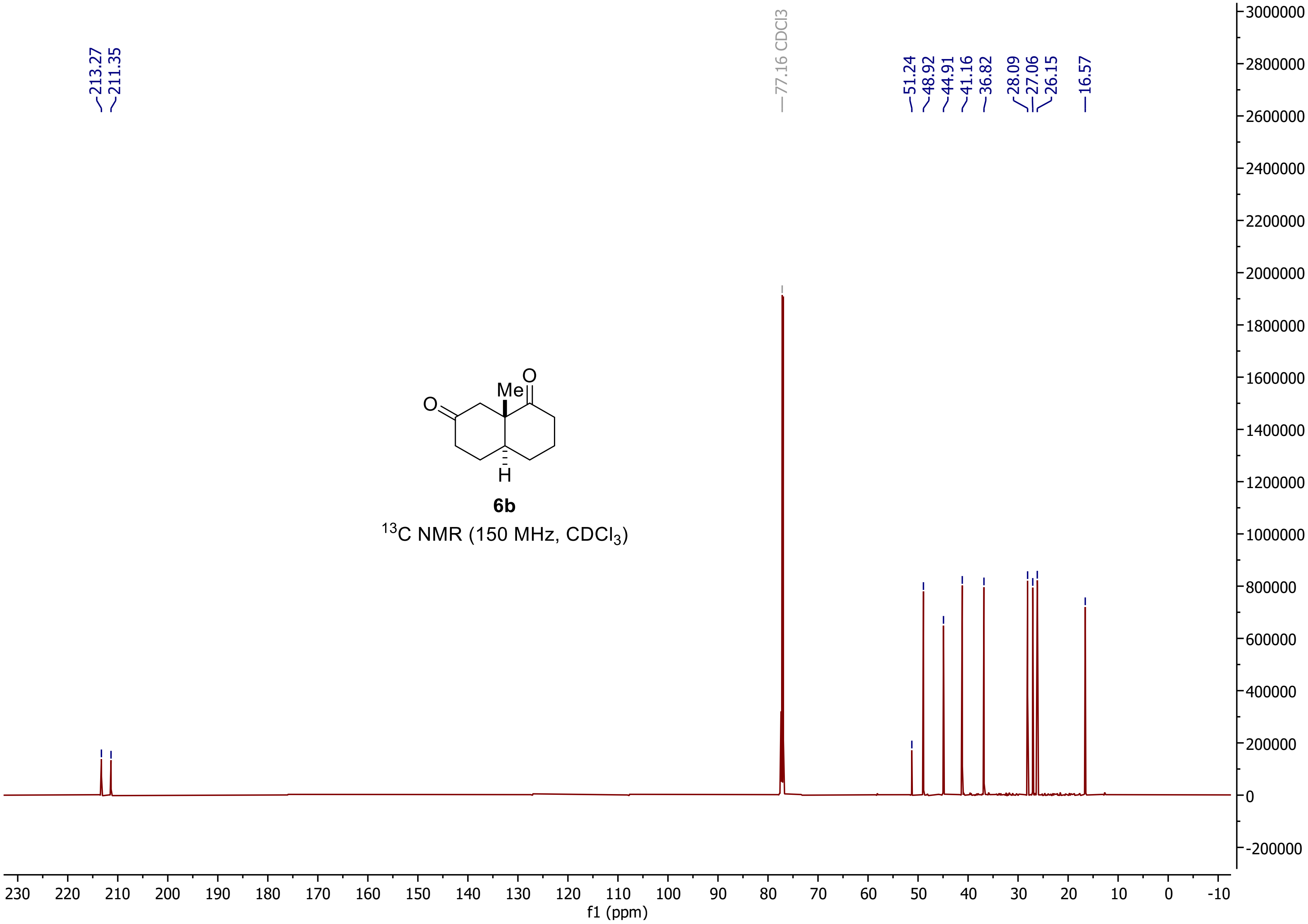

213.27
211.35
77.16 CDCl3
51.24
48.92
44.91
41.16
36.82
28.09
27.06
26.15
16.57
Me
O
H
6b
^{13}C NMR (150 MHz, $CDCl_3$)
f1 (ppm)

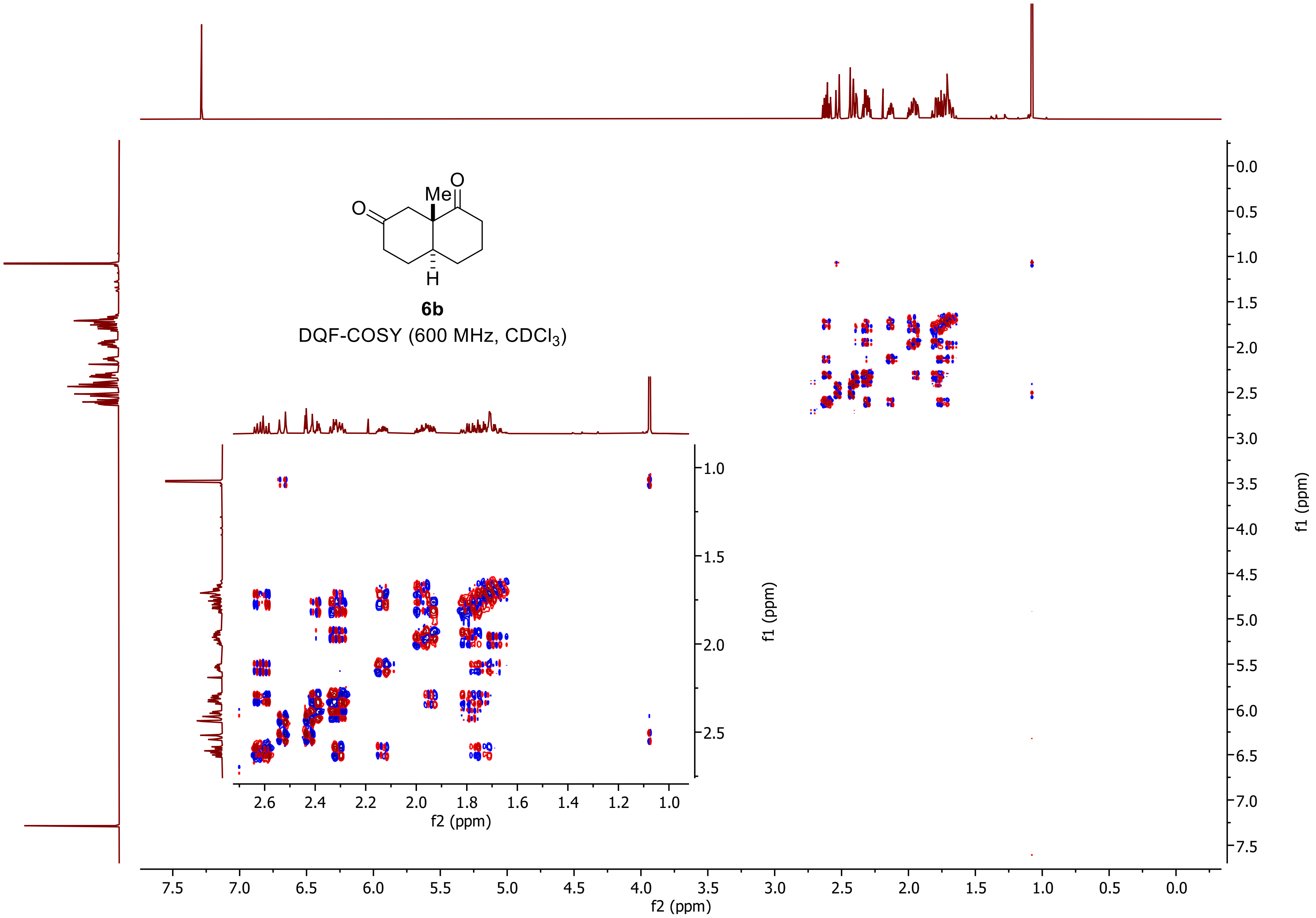
Me
O
O
H
6b
DQF-COSY (600 MHz, CDCl3)
f2 (ppm)
f1 (ppm)

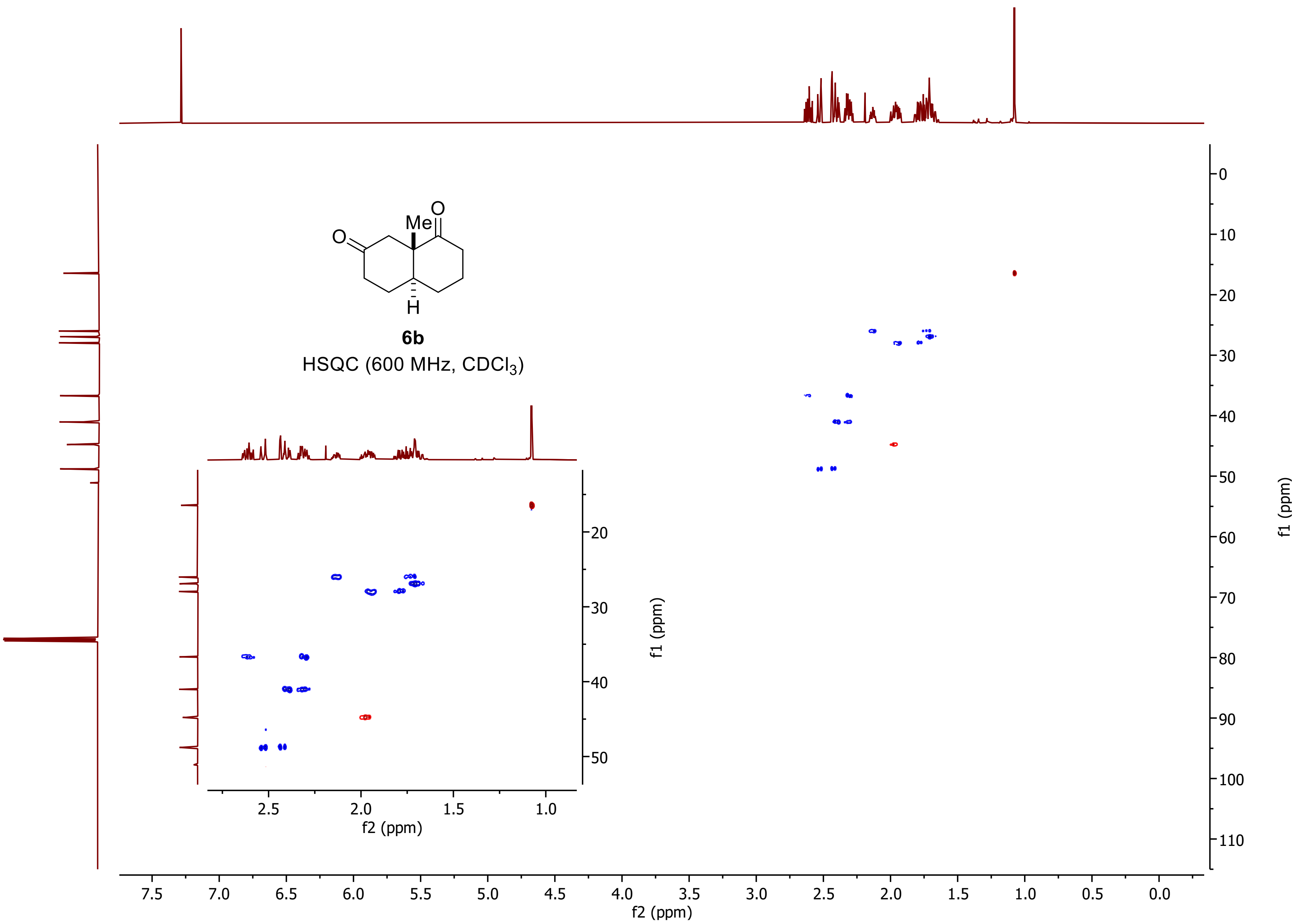

Me
O
O
H
6b
HSQC (600 MHz, CDCl3)
f2 (ppm)
f1 (ppm)

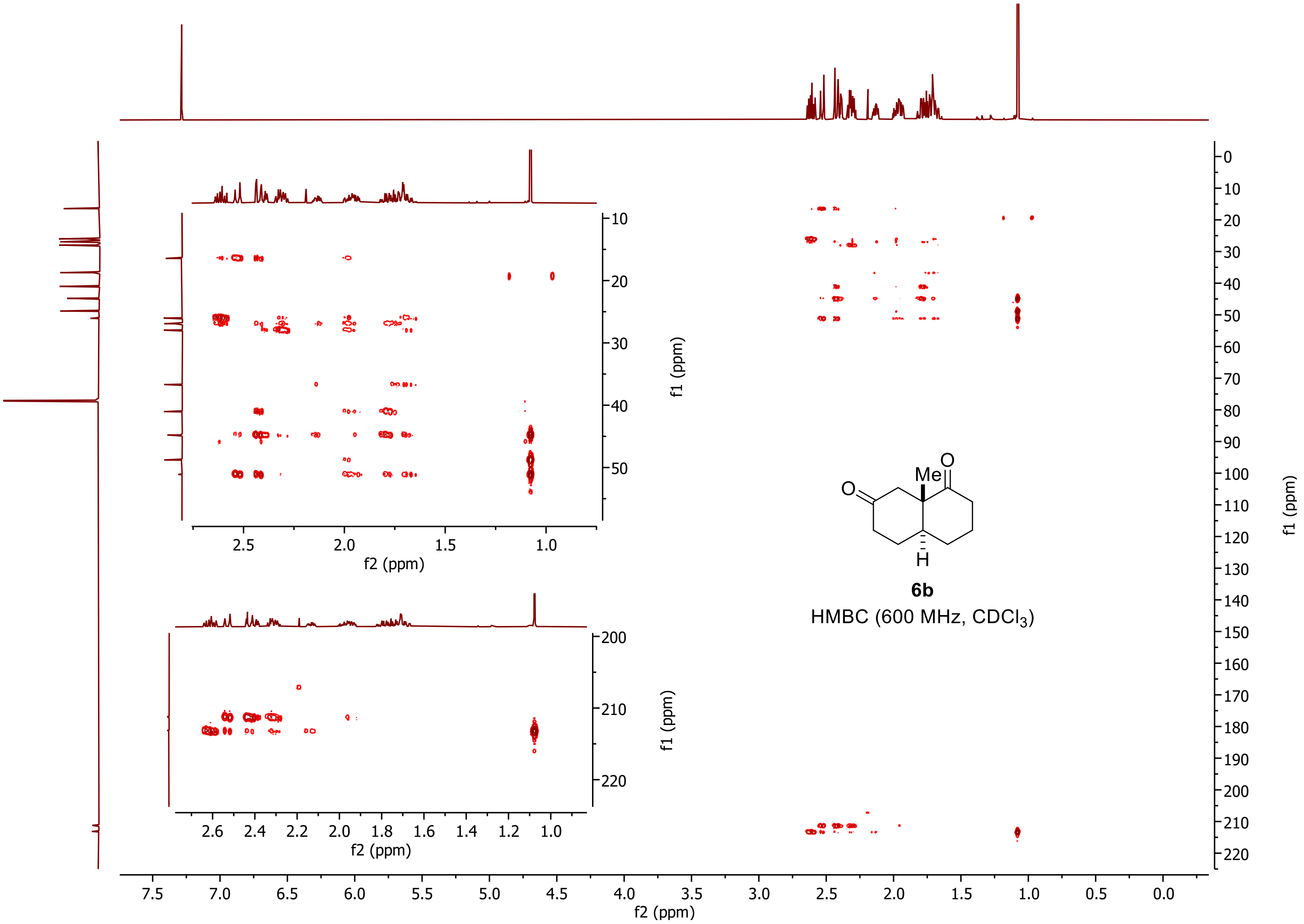
f2 (ppm)
f1 (ppm)
O
Me
O
H
6b
HMBC (600 MHz, CDCl3)

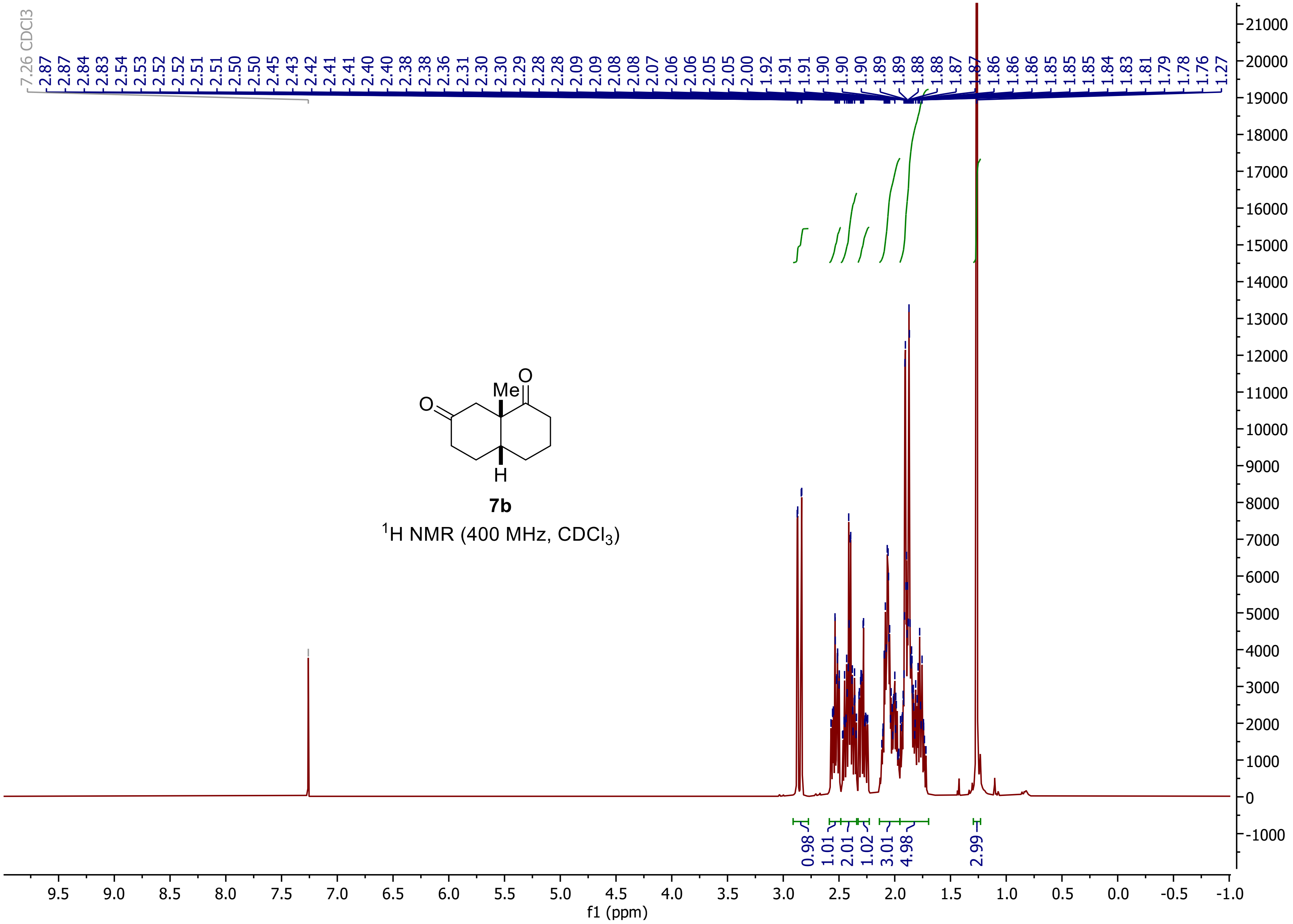
7.26 CDCl3
2.87
2.87
2.84
2.83
2.54
2.53
2.52
2.52
2.51
2.51
2.50
2.50
2.45
2.43
2.42
2.41
2.41
2.40
2.40
2.38
2.38
2.36
2.31
2.30
2.30
2.29
2.28
2.28
2.09
2.09
2.08
2.08
2.07
2.06
2.06
2.05
2.05
2.00
1.92
1.91
1.91
1.90
1.90
1.90
1.89
1.89
1.88
1.88
1.87
1.87
1.86
1.86
1.86
1.85
1.85
1.84
1.83
1.81
1.79
1.78
1.76
1.27
Me
O
O
H
7b
1H NMR (400 MHz, CDCl3)
0.98
1.01
2.01
1.02
3.01
4.98
2.99
f1 (ppm)

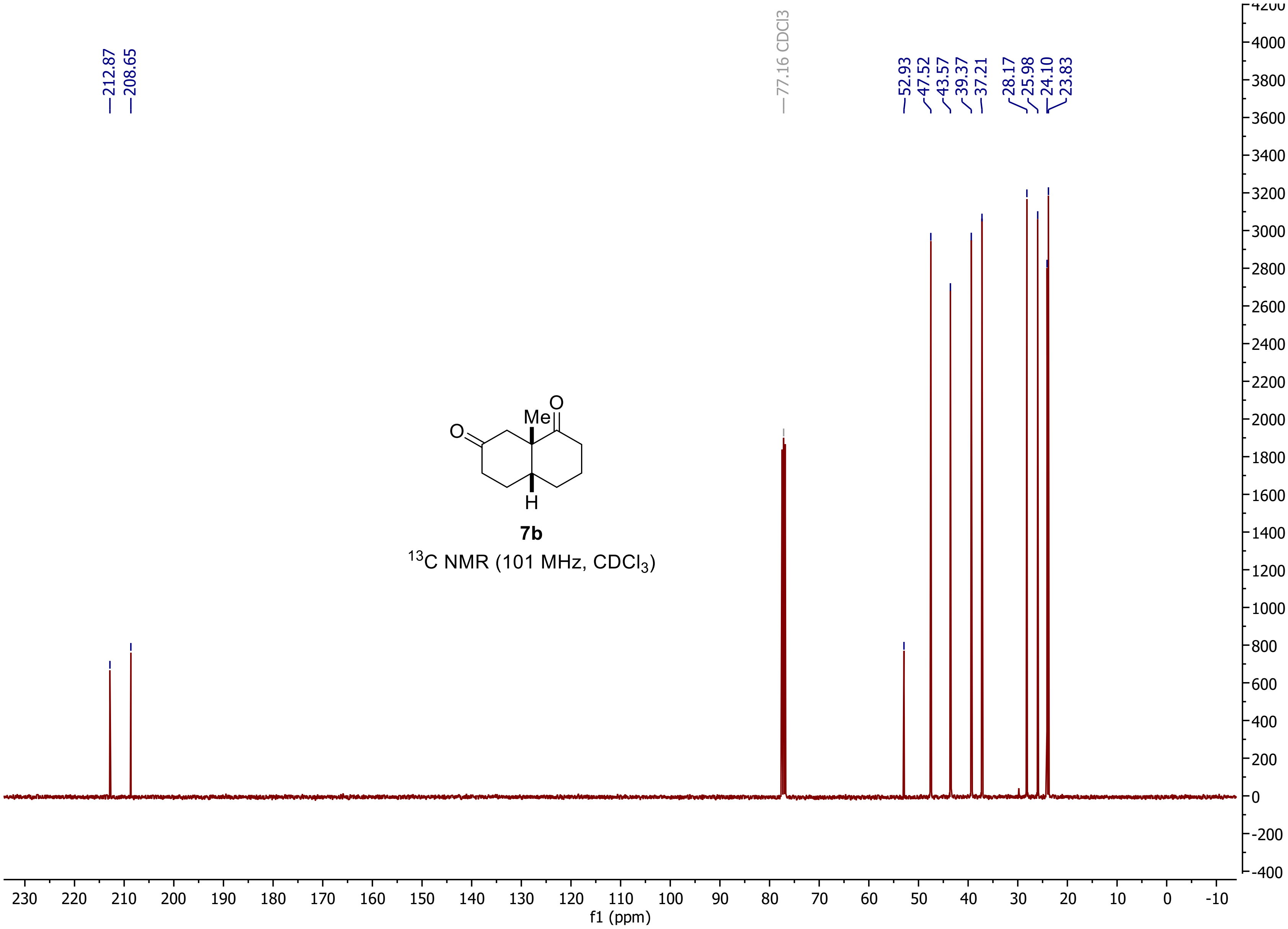
212.87
208.65
77.16 CDCl3
52.93
47.52
43.57
39.37
37.21
28.17
25.98
24.10
23.83
Me
O
O
H
7b
^{13}C NMR (101 MHz, CDCl$_3$)
f1 (ppm)

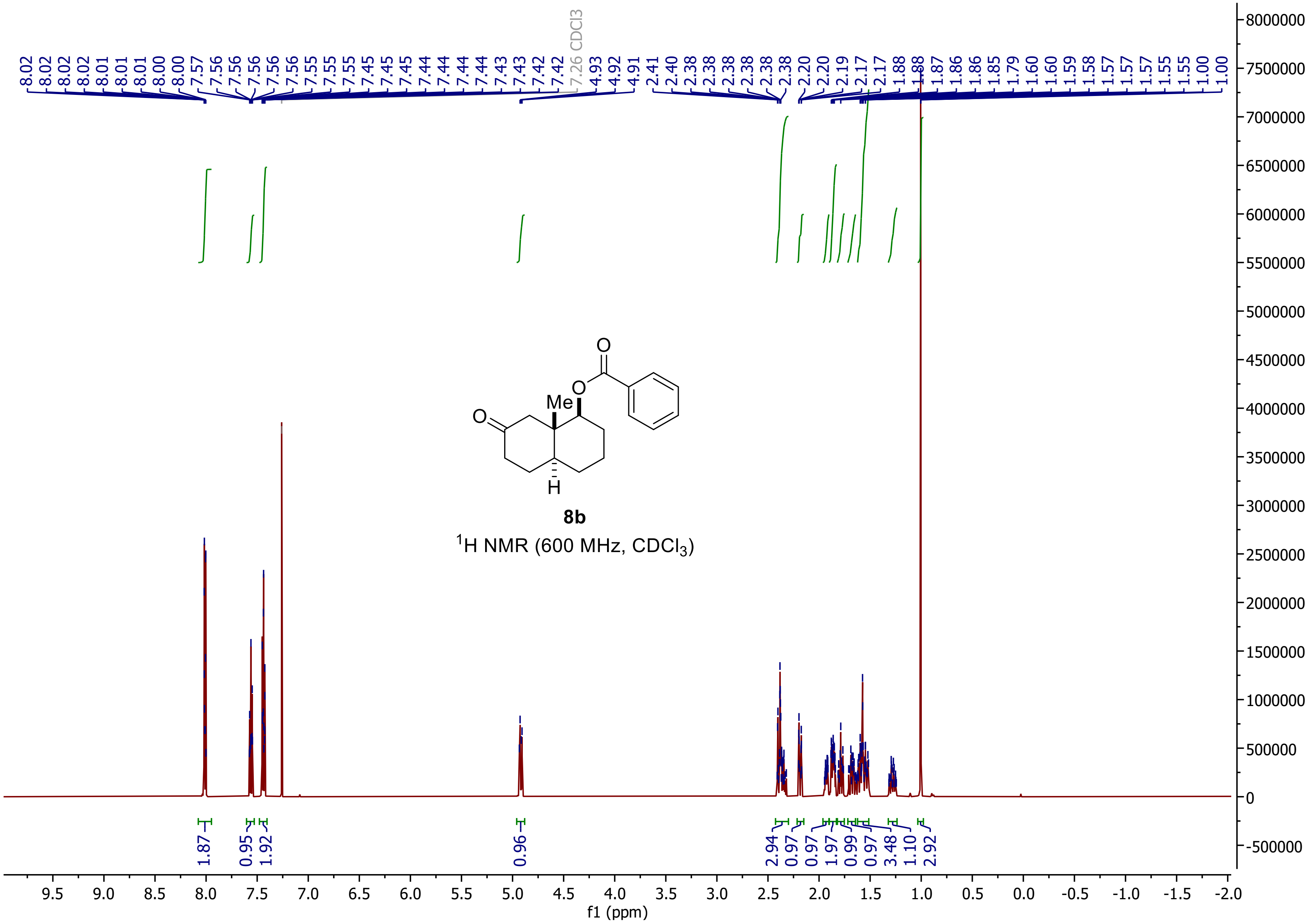
8b
1H NMR (600 MHz, CDCl3)
7.26 CDCl3
f1 (ppm)

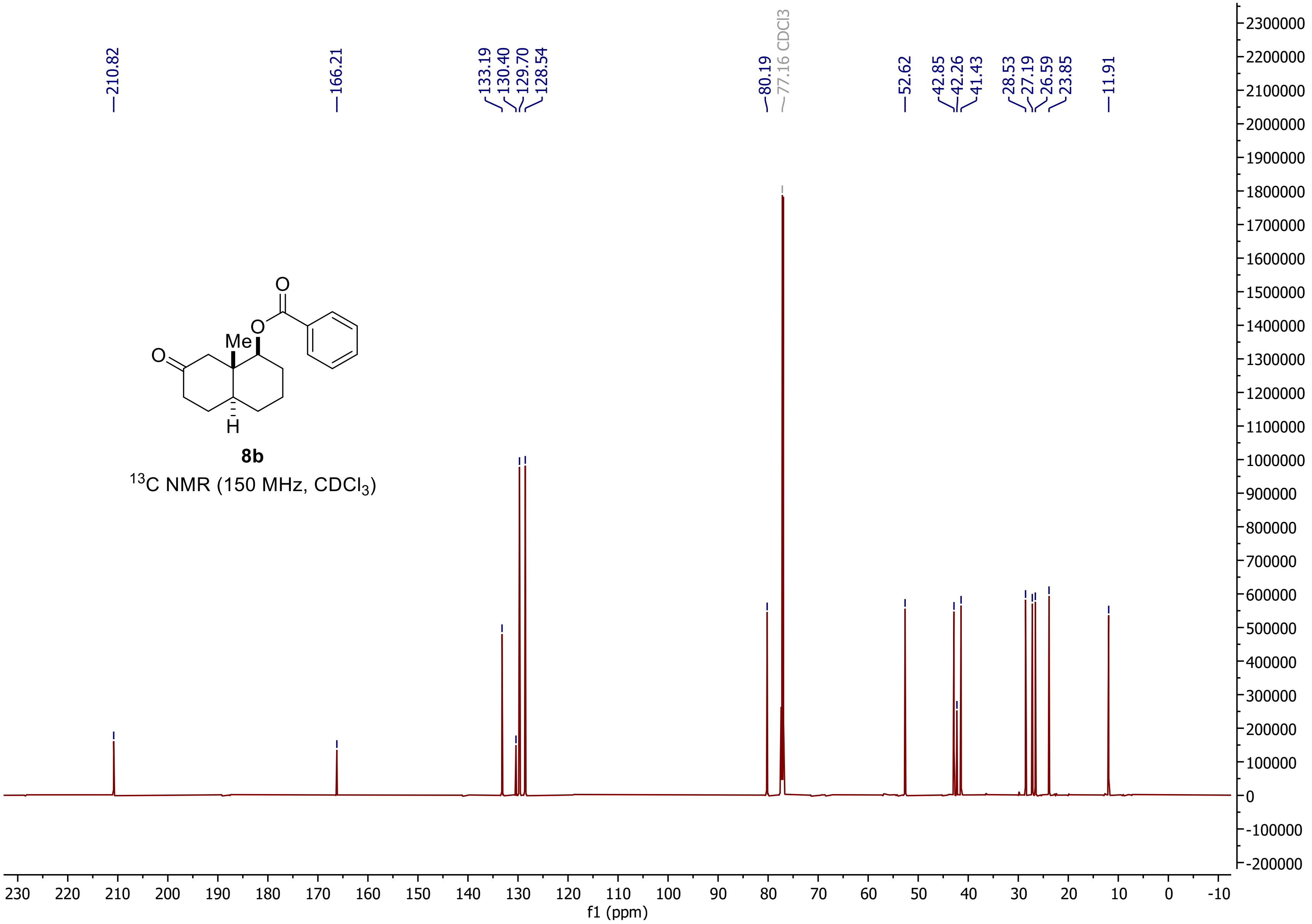
210.82
166.21
133.19
130.40
129.70
128.54
80.19
77.16 CDCl3
52.62
42.85
42.26
41.43
28.53
27.19
26.59
23.85
11.91
O
O
Me
O
H
8b
13C NMR (150 MHz, CDCl3)
f1 (ppm)

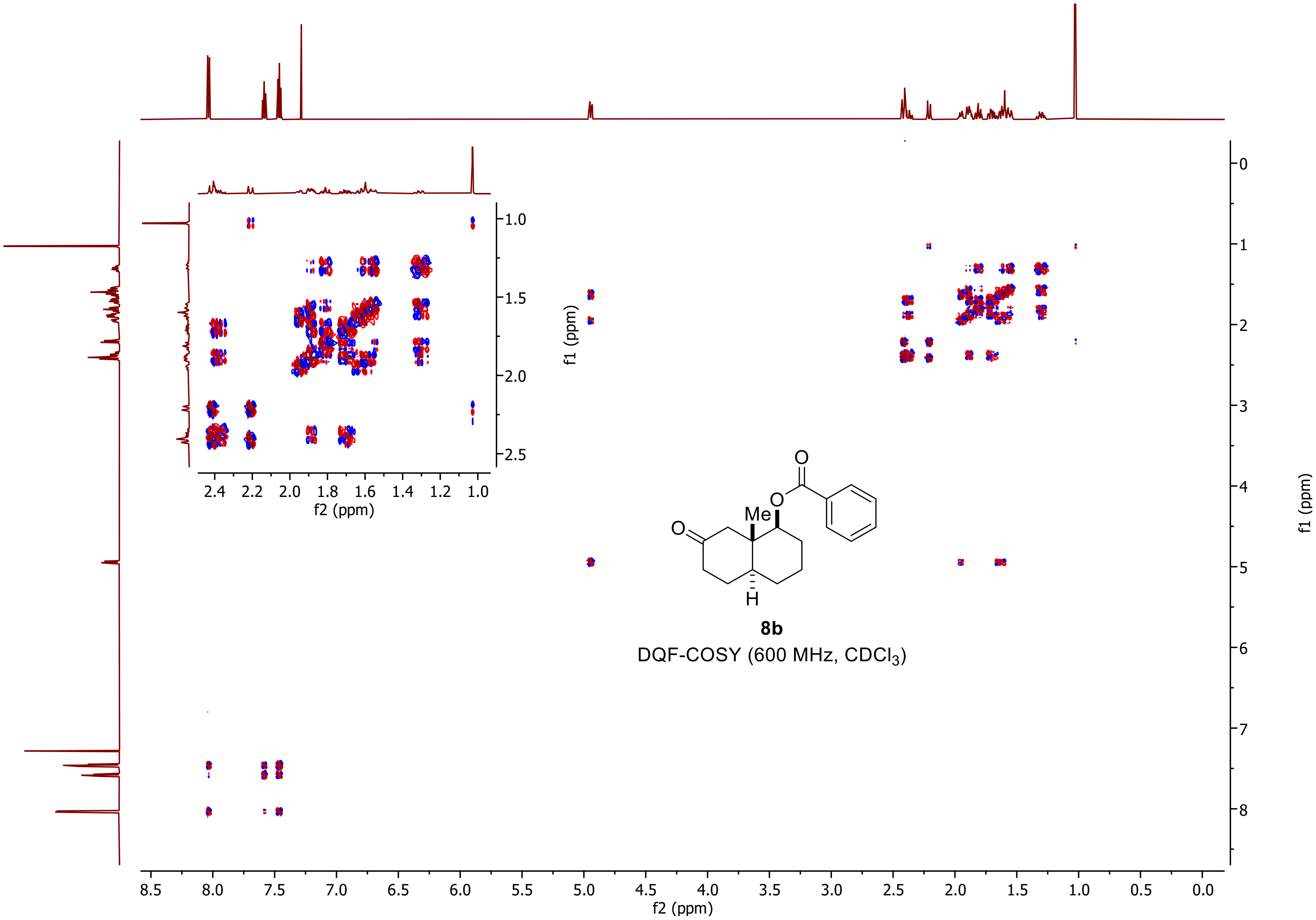

O
Me
O
O
H
8b
DQF-COSY (600 MHz, $CDCl_3$)
f2 (ppm)
f1 (ppm)

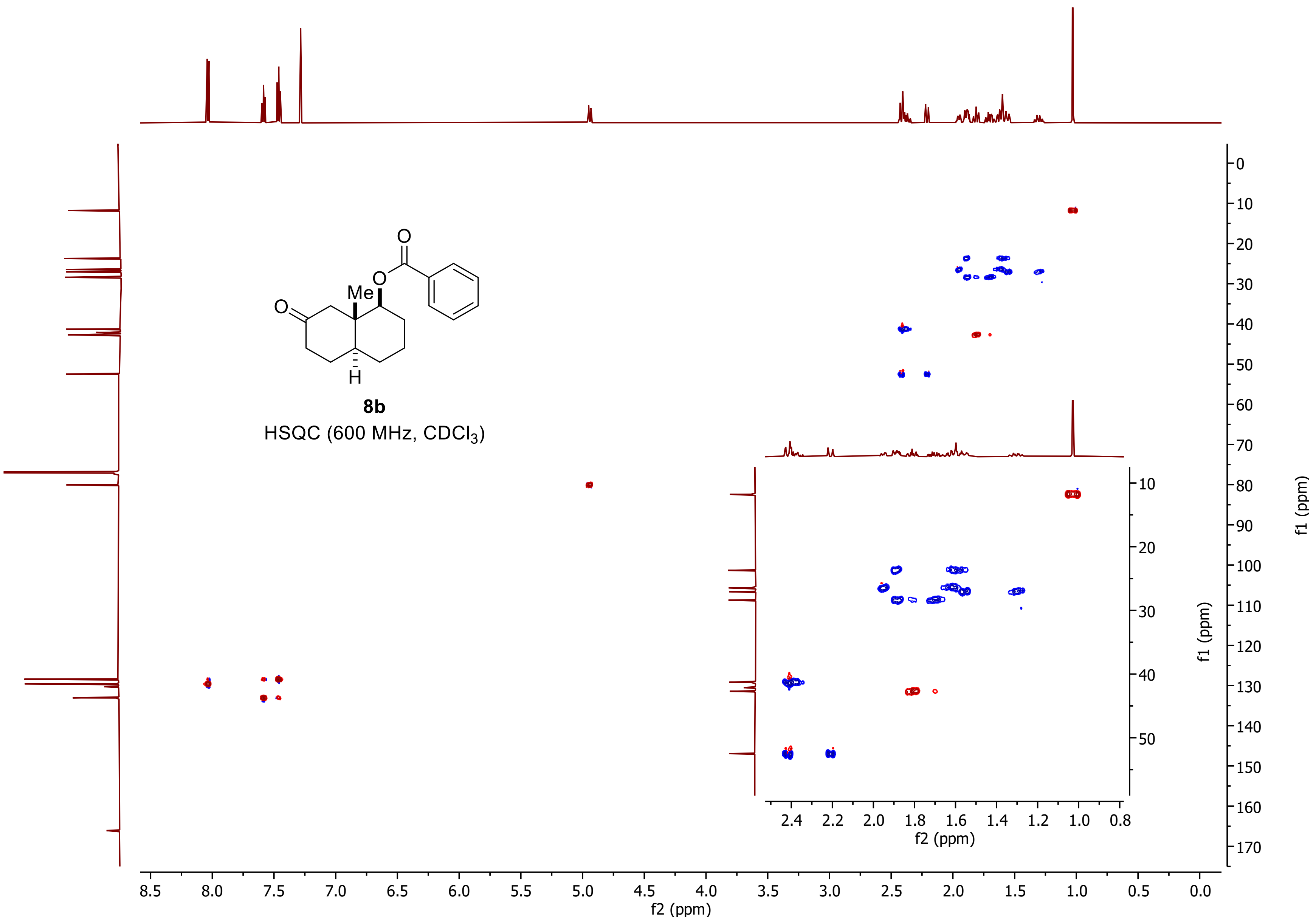
O
Me
O
O
H
8b
HSQC (600 MHz, CDCl3)
f1 (ppm)
f2 (ppm)

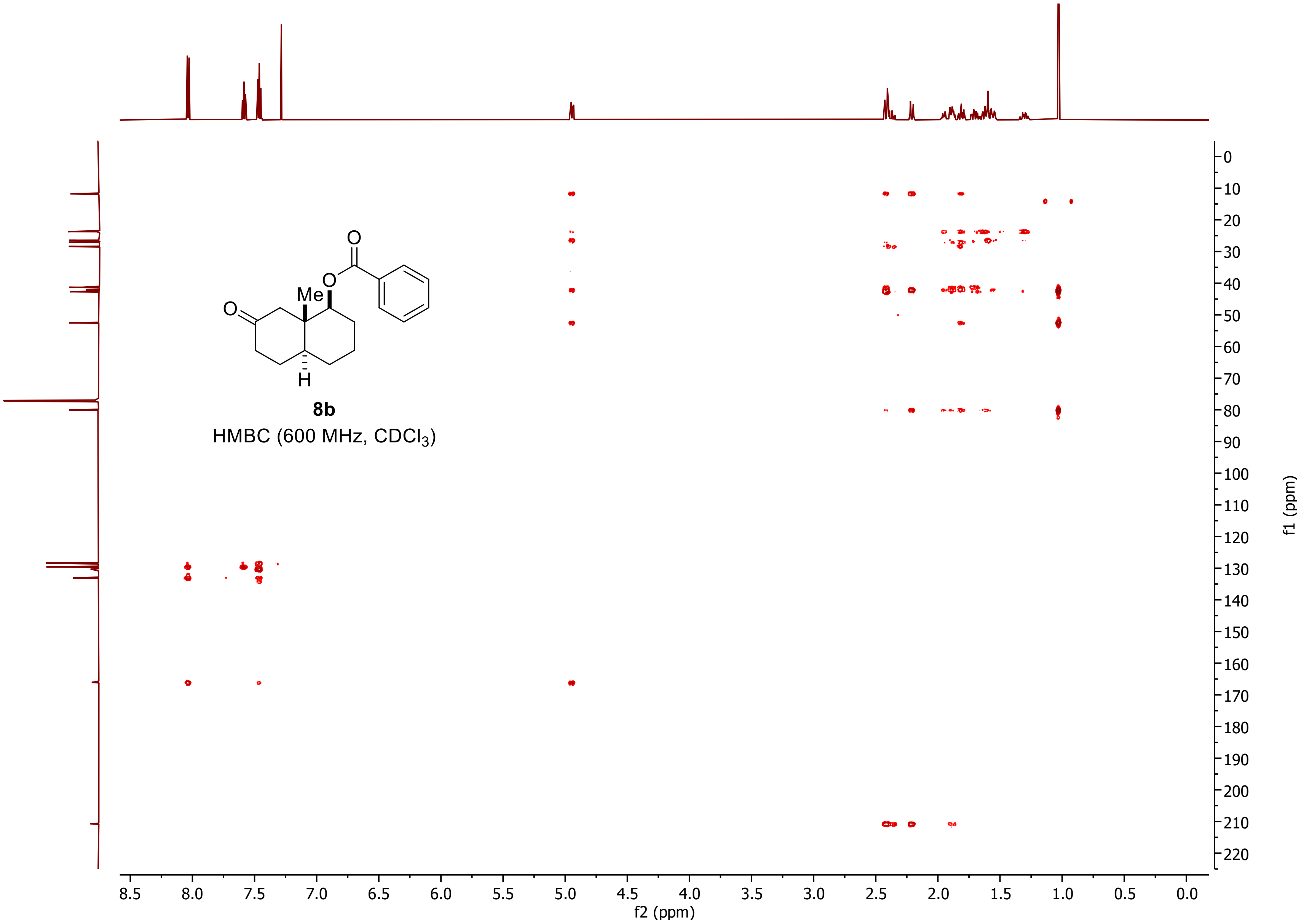
8b
HMBC (600 MHz, $CDCl_3$)
Me
O
H
f2 (ppm)
f1 (ppm)

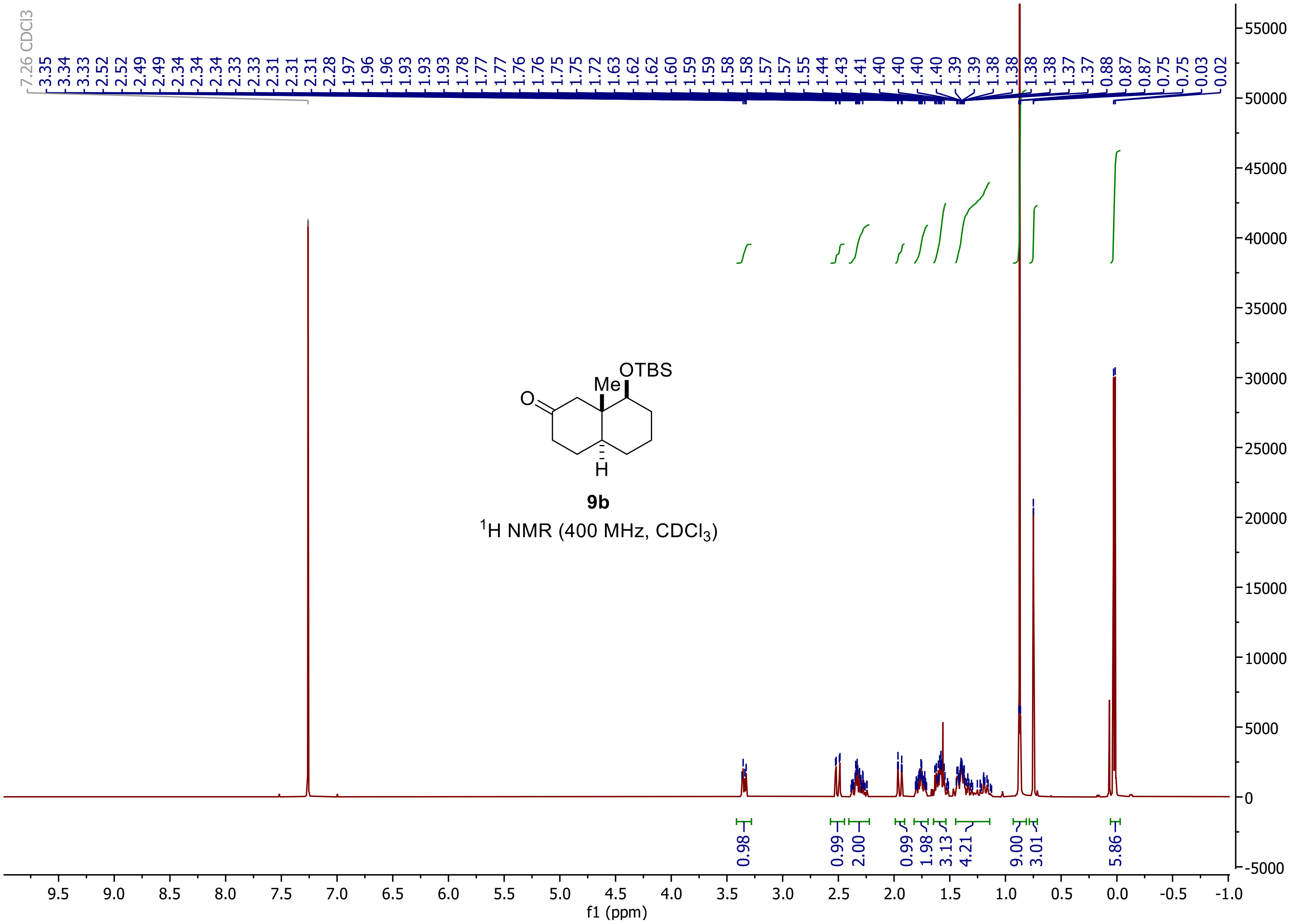

OTBS
Me
O
H
9b
¹H NMR (400 MHz, CDCl3)
f1 (ppm)

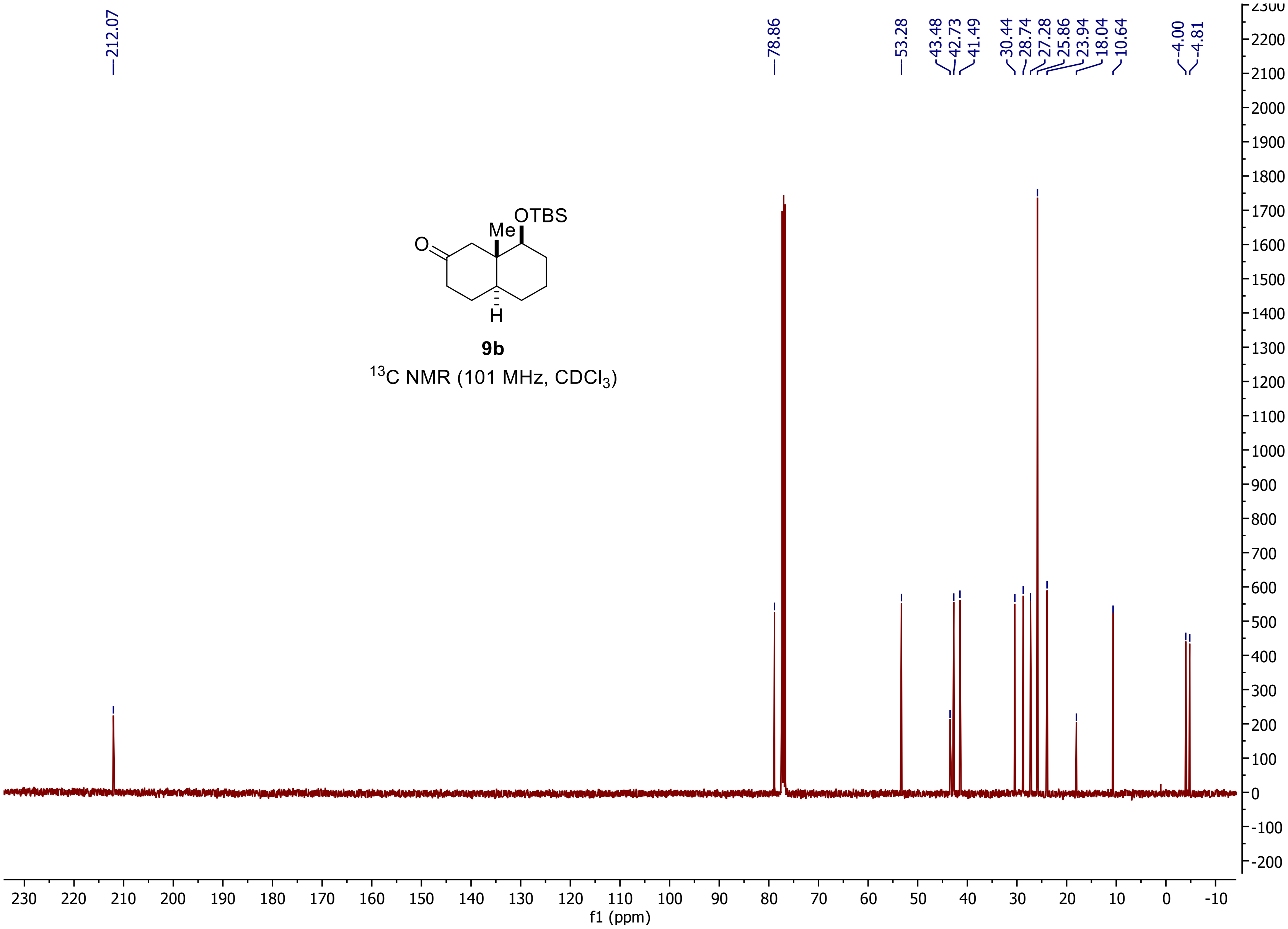
212.07
78.86
53.28
43.48
42.73
41.49
30.44
28.74
27.28
25.86
23.94
18.04
10.64
-4.00
-4.81
OTBS
Me
O
H
9b
^{13}C NMR (101 MHz, CDCl$_3$)
f1 (ppm)

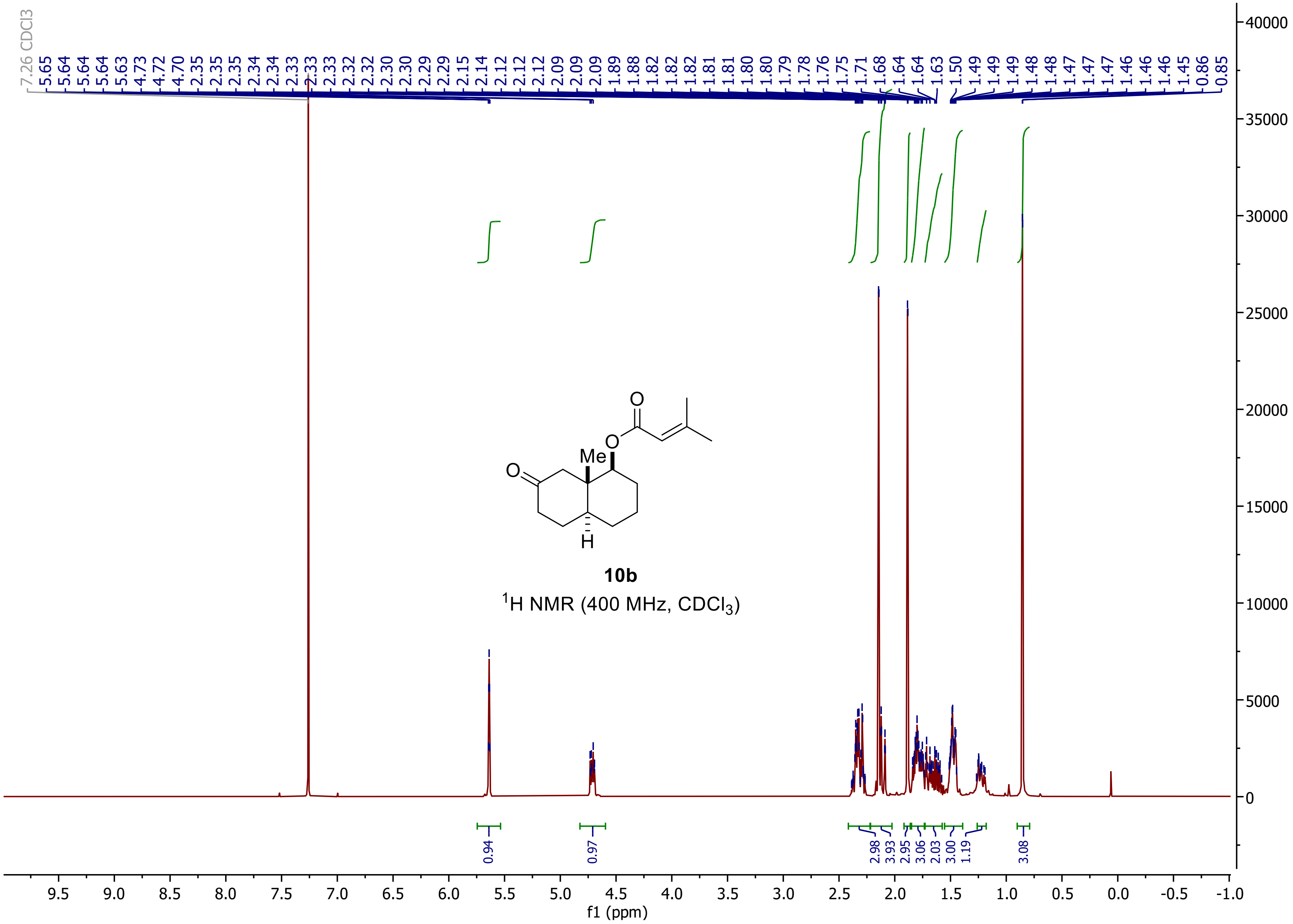
7.26 CDCl3
5.65
5.64
5.64
5.64
5.63
4.73
4.72
4.70
2.35
2.35
2.35
2.34
2.34
2.33
2.33
2.33
2.32
2.32
2.30
2.30
2.29
2.29
2.15
2.14
2.12
2.12
2.12
2.09
2.09
2.09
1.89
1.88
1.82
1.82
1.82
1.81
1.81
1.80
1.80
1.79
1.78
1.76
1.75
1.71
1.68
1.64
1.64
1.63
1.50
1.49
1.49
1.49
1.48
1.48
1.47
1.47
1.47
1.46
1.46
1.46
1.45
0.86
0.85
10b
1H NMR (400 MHz, CDCl3)
0.94
0.97
2.98
3.93
2.95
3.06
2.03
3.00
1.19
3.08
f1 (ppm)

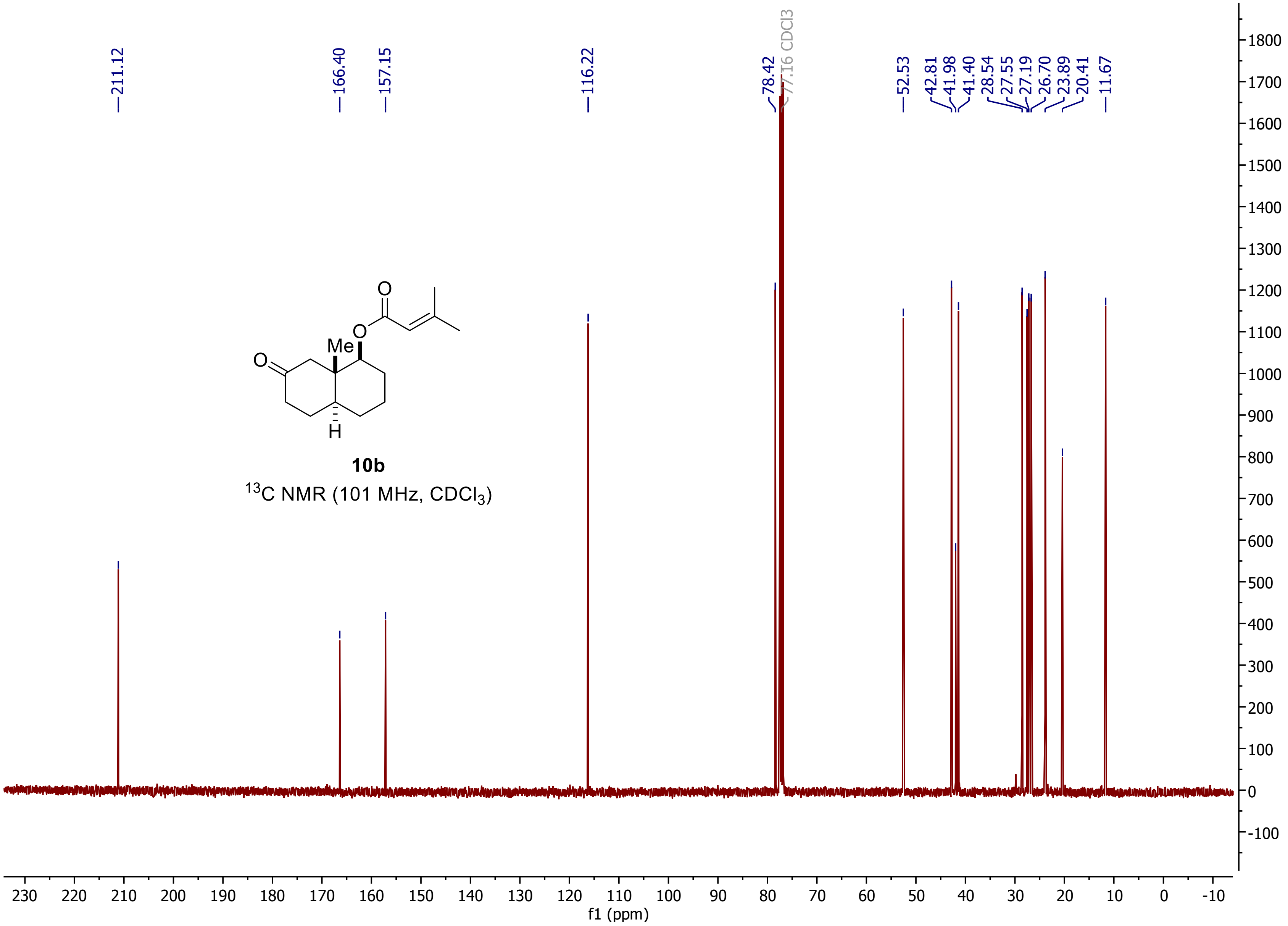

211.12
166.40
157.15
116.22
78.42
77.16 CDCl3
52.53
42.81
41.98
41.40
28.54
27.55
27.19
26.70
23.89
20.41
11.67
O
O
Me
O
H
10b
^{13}C NMR (101 MHz, $CDCl_3$)
f1 (ppm)

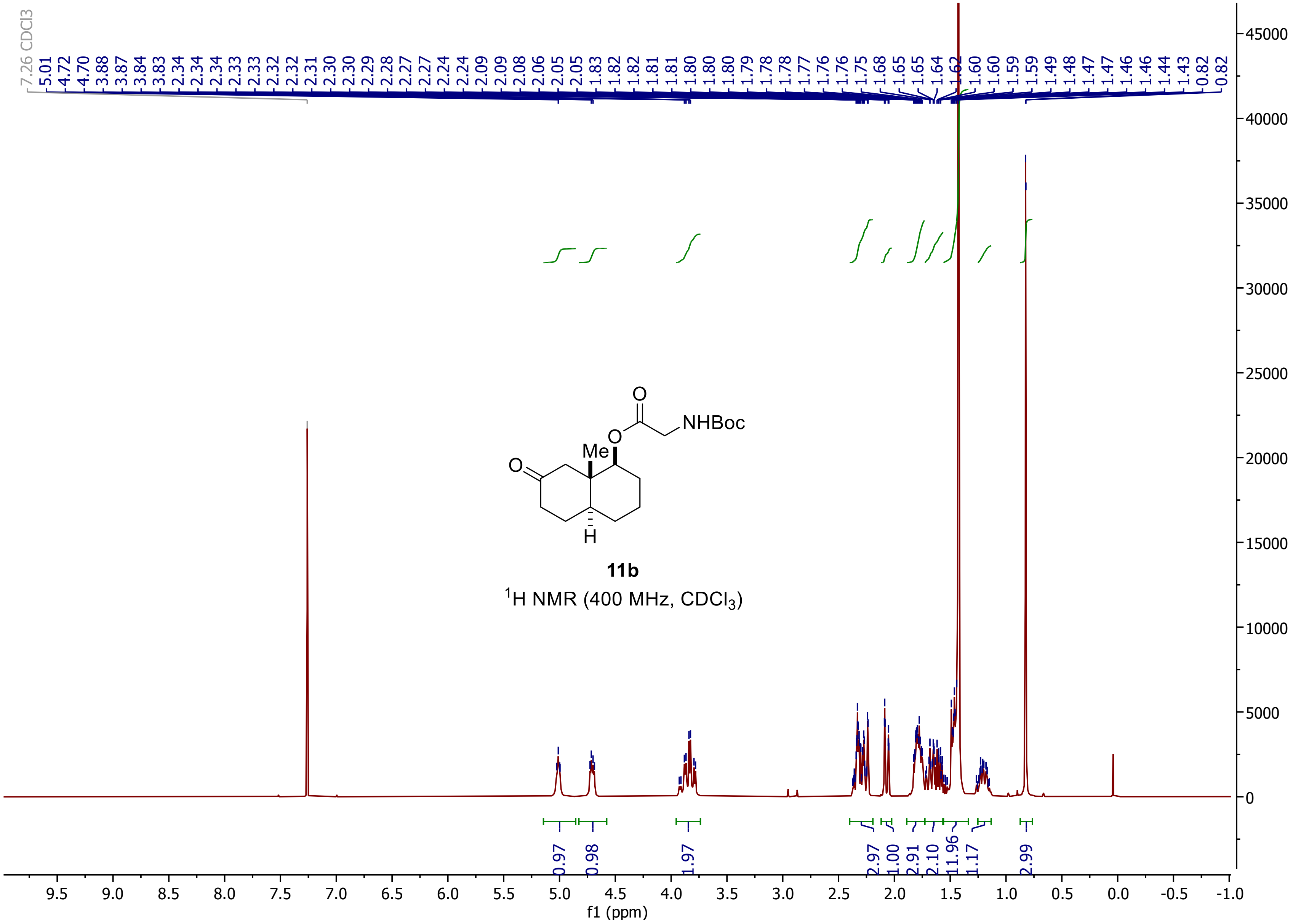

7.26 CDCl3
5.01
4.72
4.70
3.88
3.87
3.84
3.83
2.34
2.34
2.34
2.33
2.33
2.32
2.32
2.31
2.30
2.30
2.29
2.28
2.27
2.27
2.24
2.24
2.09
2.09
2.08
2.06
2.05
2.05
1.83
1.82
1.82
1.81
1.81
1.80
1.80
1.80
1.79
1.78
1.78
1.77
1.76
1.76
1.75
1.68
1.65
1.65
1.64
1.62
1.60
1.60
1.59
1.59
1.49
1.48
1.47
1.47
1.46
1.46
1.44
1.43
0.82
0.82
NHBoc
O
Me
H
11b
^{1}H NMR (400 MHz, CDCl$_3$)
0.97
0.98
1.97
2.97
1.00
2.91
2.10
11.96
1.17
2.99
f1 (ppm)

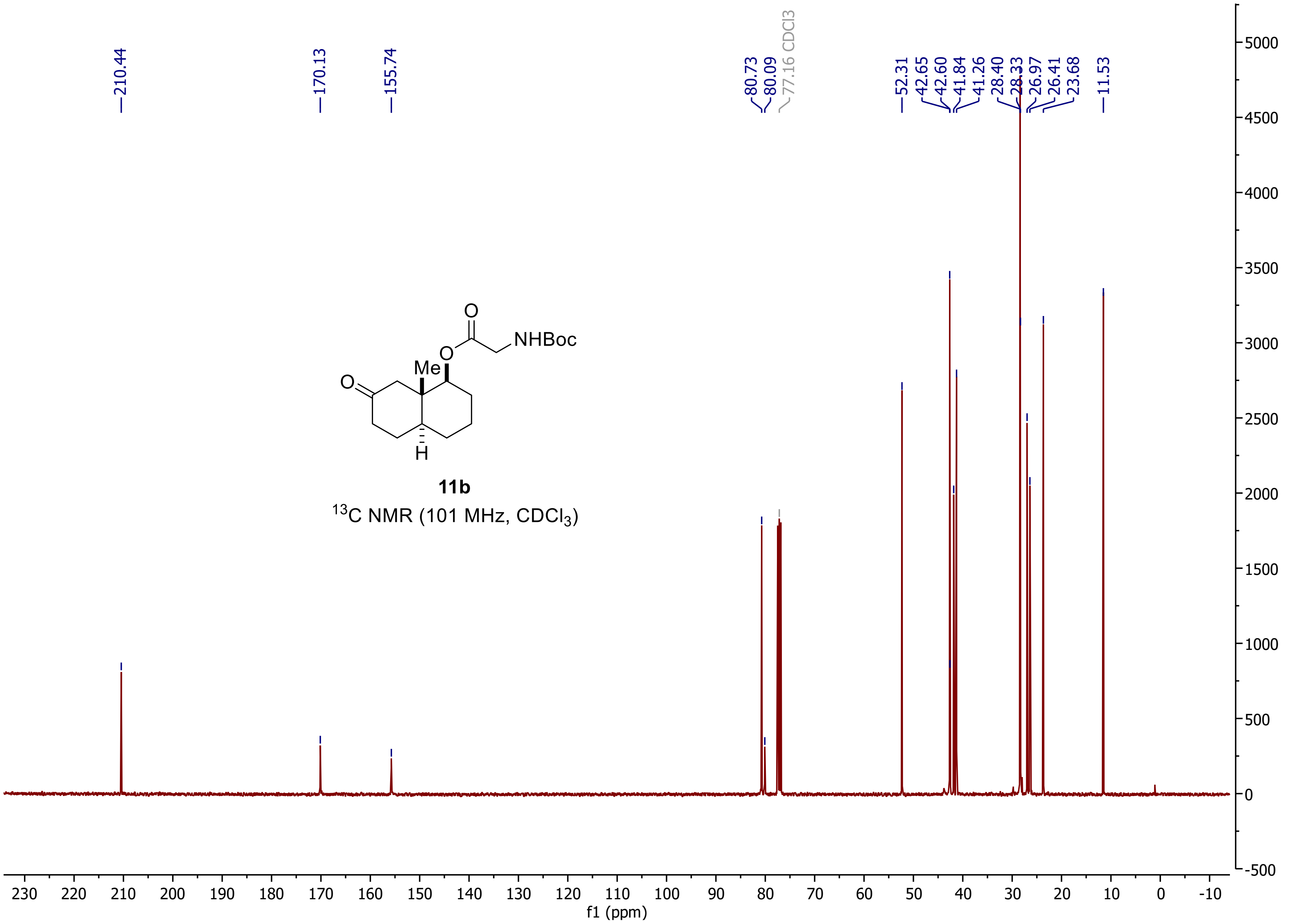
210.44
170.13
155.74
80.73
80.09
77.16 CDCl3
52.31
42.65
42.60
41.84
41.26
28.40
28.33
26.97
26.41
23.68
11.53
O
NHBoc
O
Me
O
H
11b
^{13}C NMR (101 MHz, CDCl$_3$)
f1 (ppm)

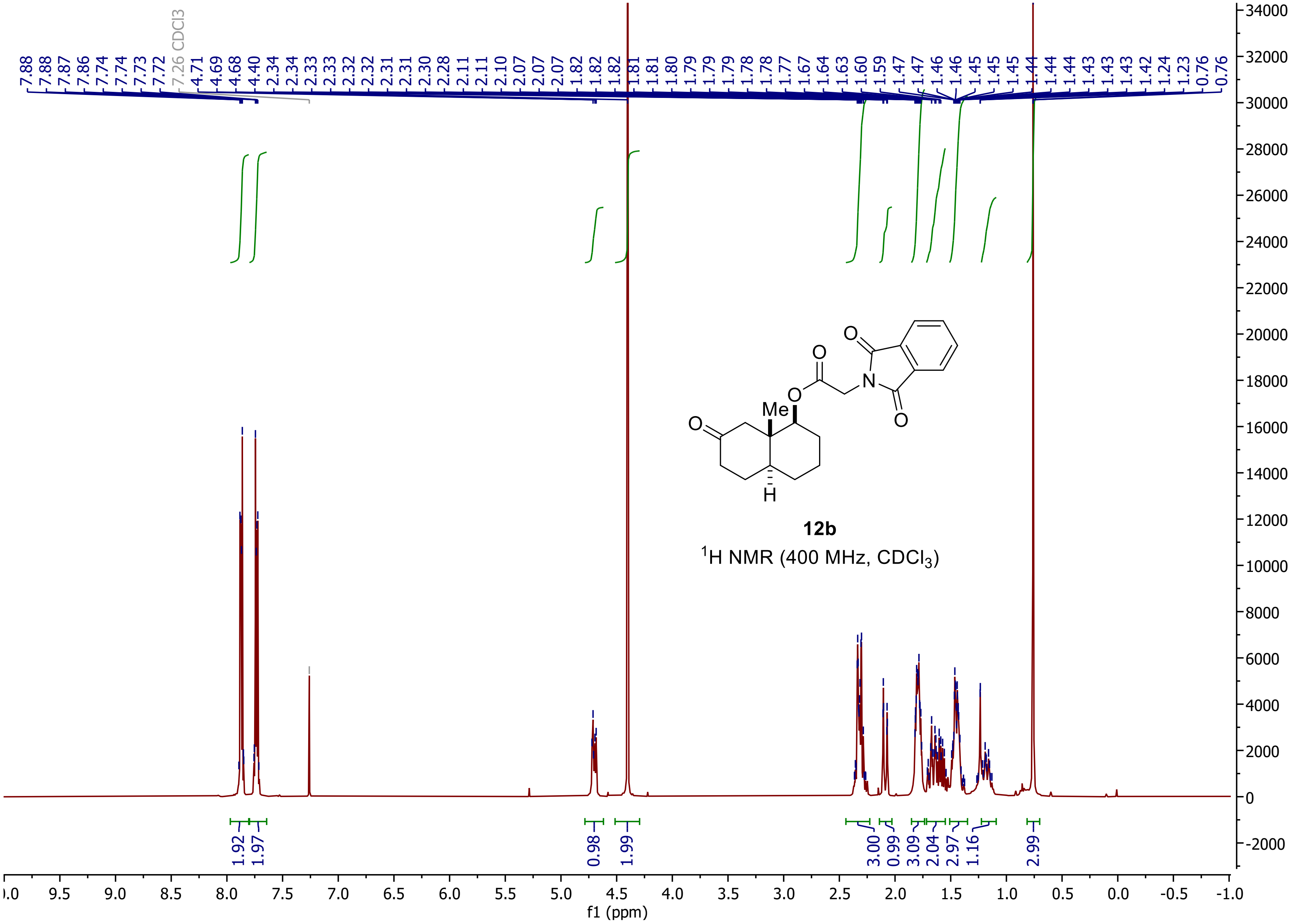

12b
^{1}H NMR (400 MHz, CDCl$_3$)
7.88 7.88 7.87 7.86 7.74 7.74 7.73 7.72 7.26 CDCl3 4.71 4.69 4.68 4.40 2.34 2.34 2.33 2.33 2.32 2.32 2.31 2.31 2.30 2.28 2.11 2.11 2.10 2.07 2.07 2.07 1.82 1.82 1.82 1.81 1.81 1.80 1.79 1.79 1.79 1.78 1.78 1.77 1.67 1.64 1.63 1.60 1.59 1.47 1.47 1.46 1.46 1.45 1.45 1.45 1.44 1.44 1.44 1.43 1.43 1.43 1.42 1.24 1.23 0.76 0.76
1.92 1.97 0.98 1.99 3.00 0.99 3.09 2.04 2.97 1.16 2.99
f1 (ppm)

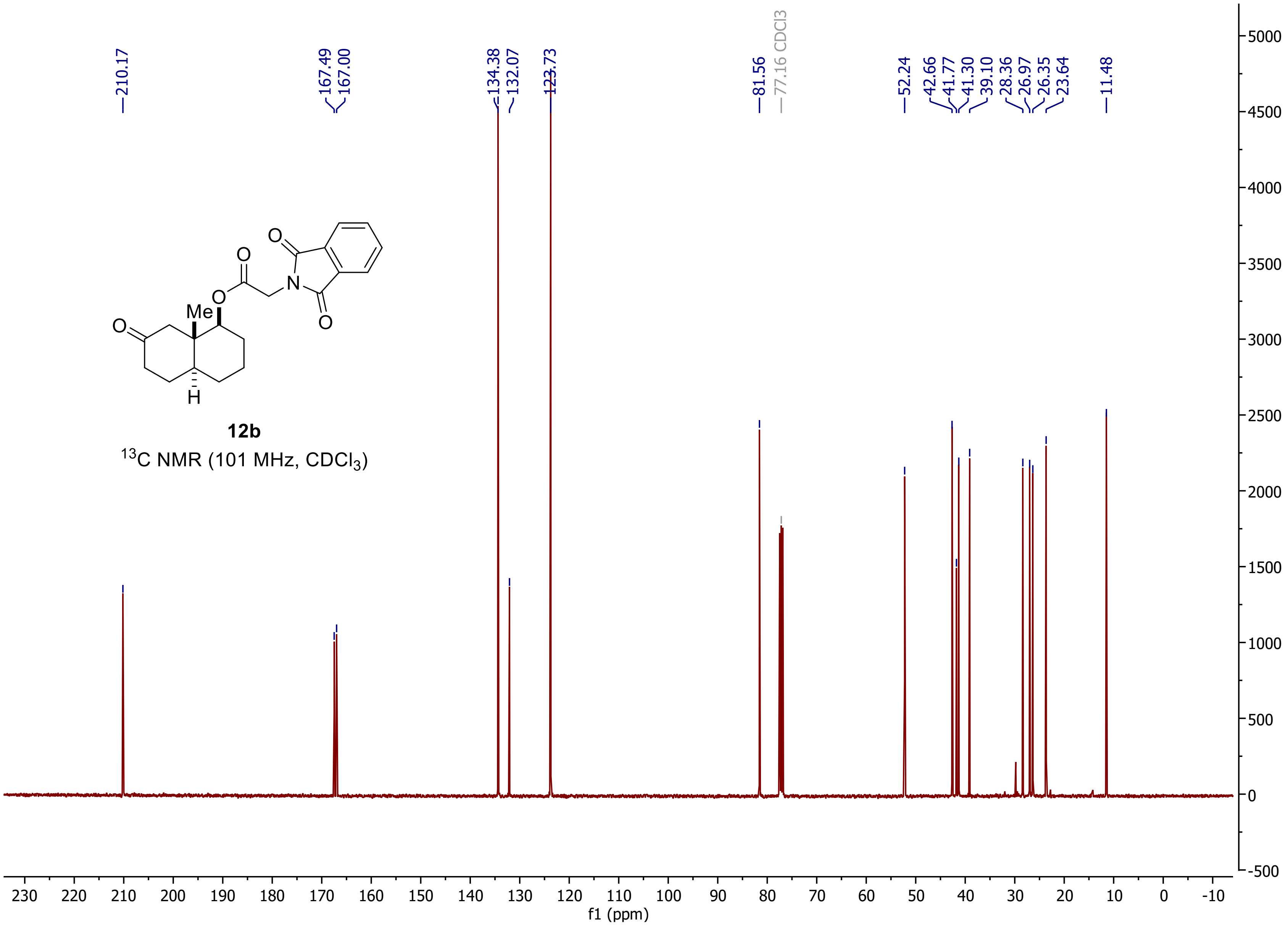
210.17
167.49
167.00
134.38
132.07
123.73
81.56
77.16 CDCl3
52.24
42.66
41.77
41.30
39.10
28.36
26.97
26.35
23.64
11.48
12b
^{13}C NMR (101 MHz, $CDCl_3$)
f1 (ppm)

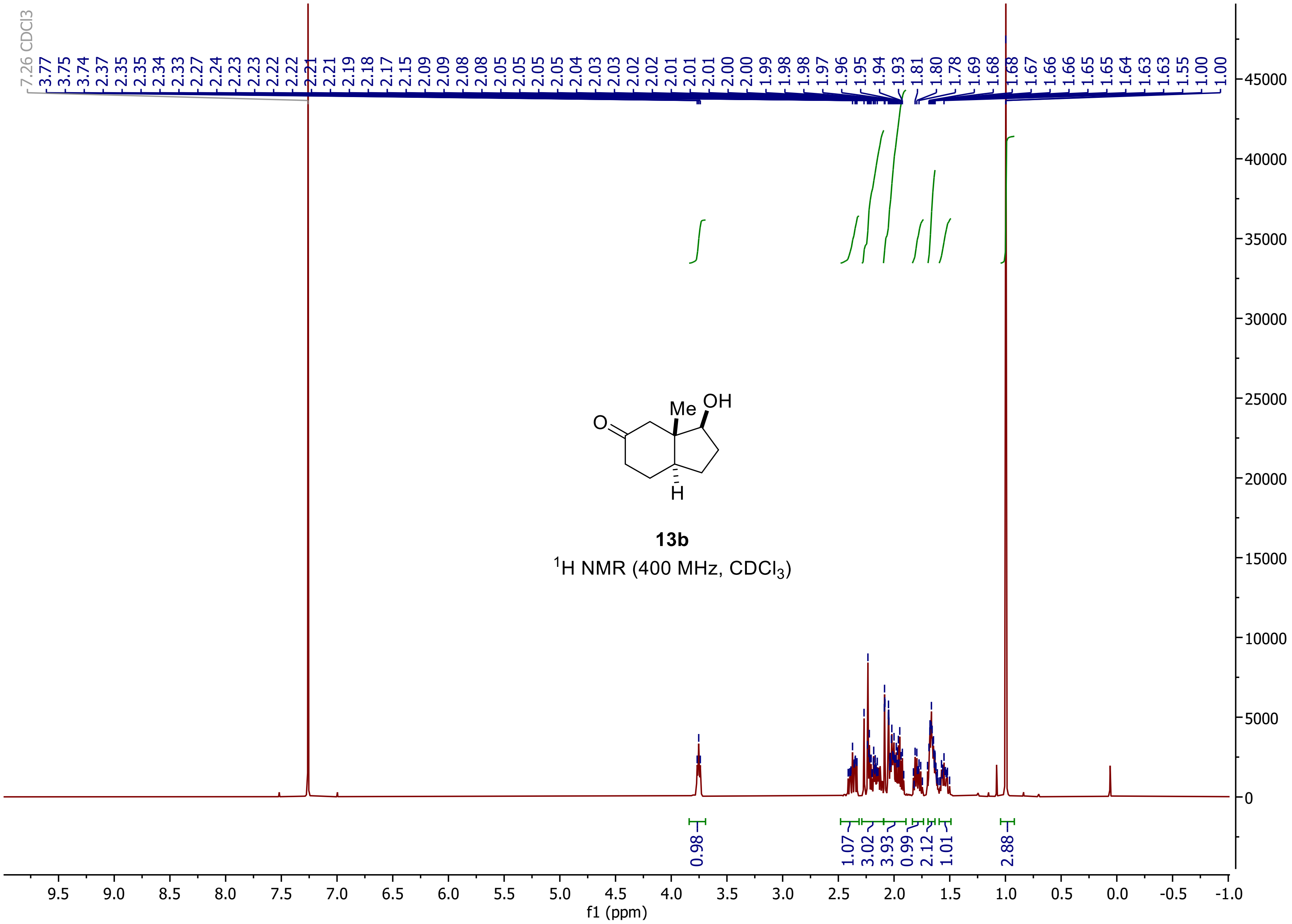
13b
^{1}H NMR (400 MHz, $CDCl_3$)
f1 (ppm)

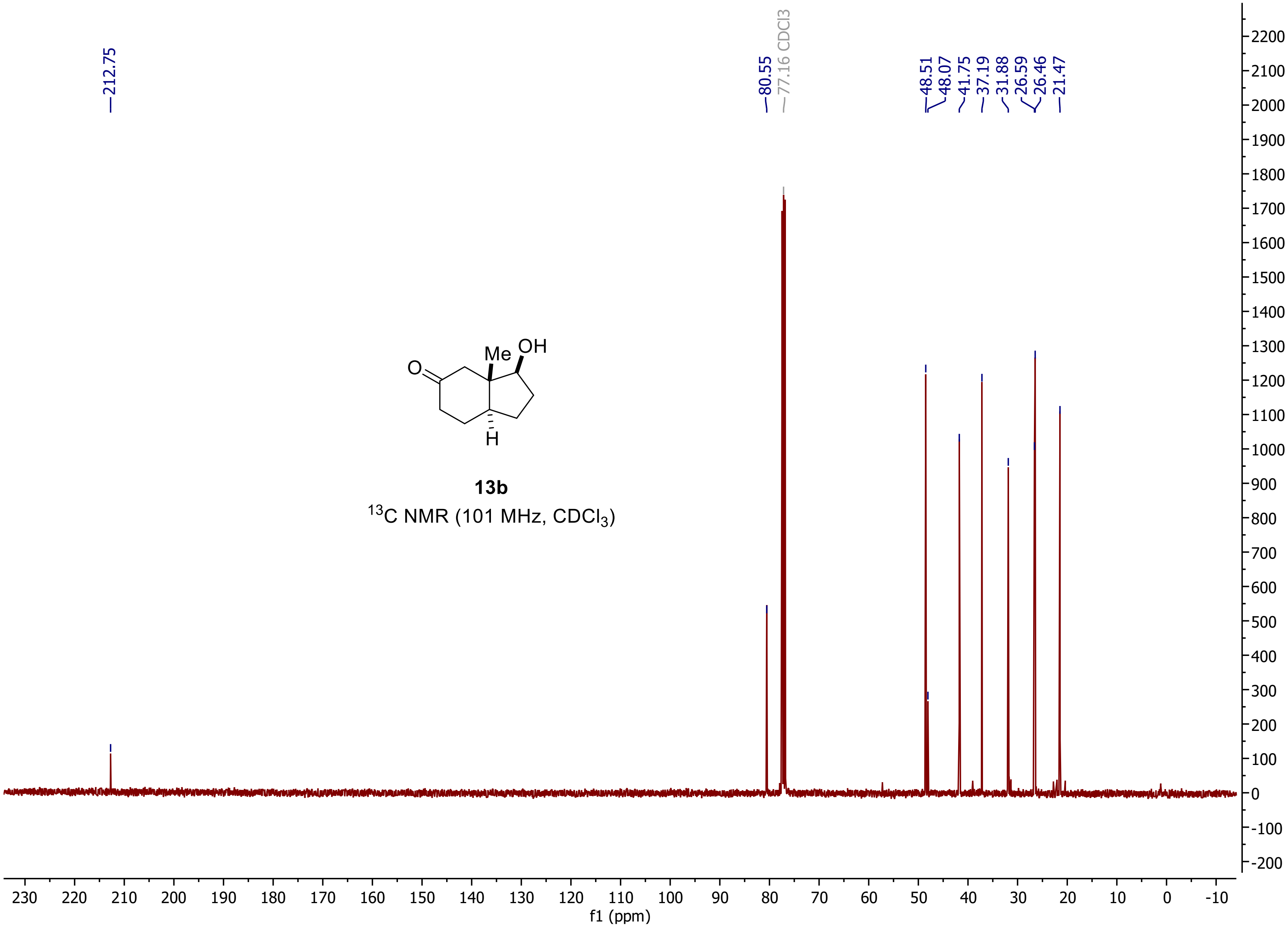
212.75
80.55
77.16 CDCl3
48.51
48.07
41.75
37.19
31.88
26.59
26.46
21.47
O
Me
OH
H
13b
13C NMR (101 MHz, CDCl3)
f1 (ppm)

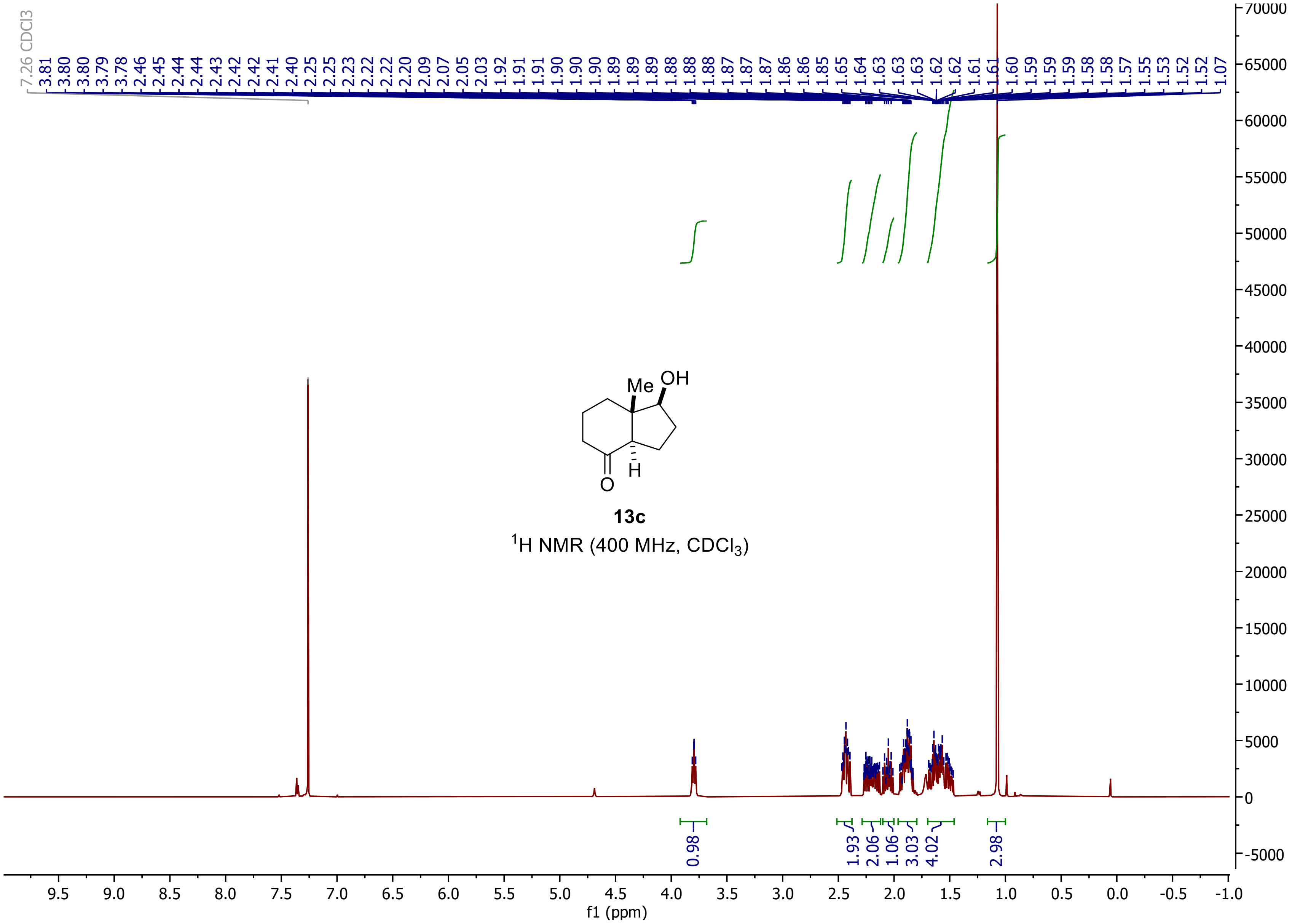

7.26 CDCl3
3.81
3.80
3.80
3.79
3.78
2.46
2.45
2.44
2.44
2.43
2.42
2.42
2.41
2.40
2.25
2.25
2.23
2.22
2.22
2.20
2.09
2.07
2.05
2.03
1.92
1.91
1.91
1.90
1.90
1.90
1.89
1.89
1.89
1.88
1.88
1.88
1.87
1.87
1.87
1.86
1.86
1.85
1.65
1.64
1.63
1.63
1.63
1.62
1.62
1.61
1.61
1.60
1.59
1.59
1.59
1.58
1.58
1.57
1.55
1.53
1.52
1.52
1.07
Me
OH
H
O
13c
^{1}H NMR (400 MHz, $CDCl_3$)
0.98
1.93
2.06
1.06
3.03
4.02
2.98
f1 (ppm)

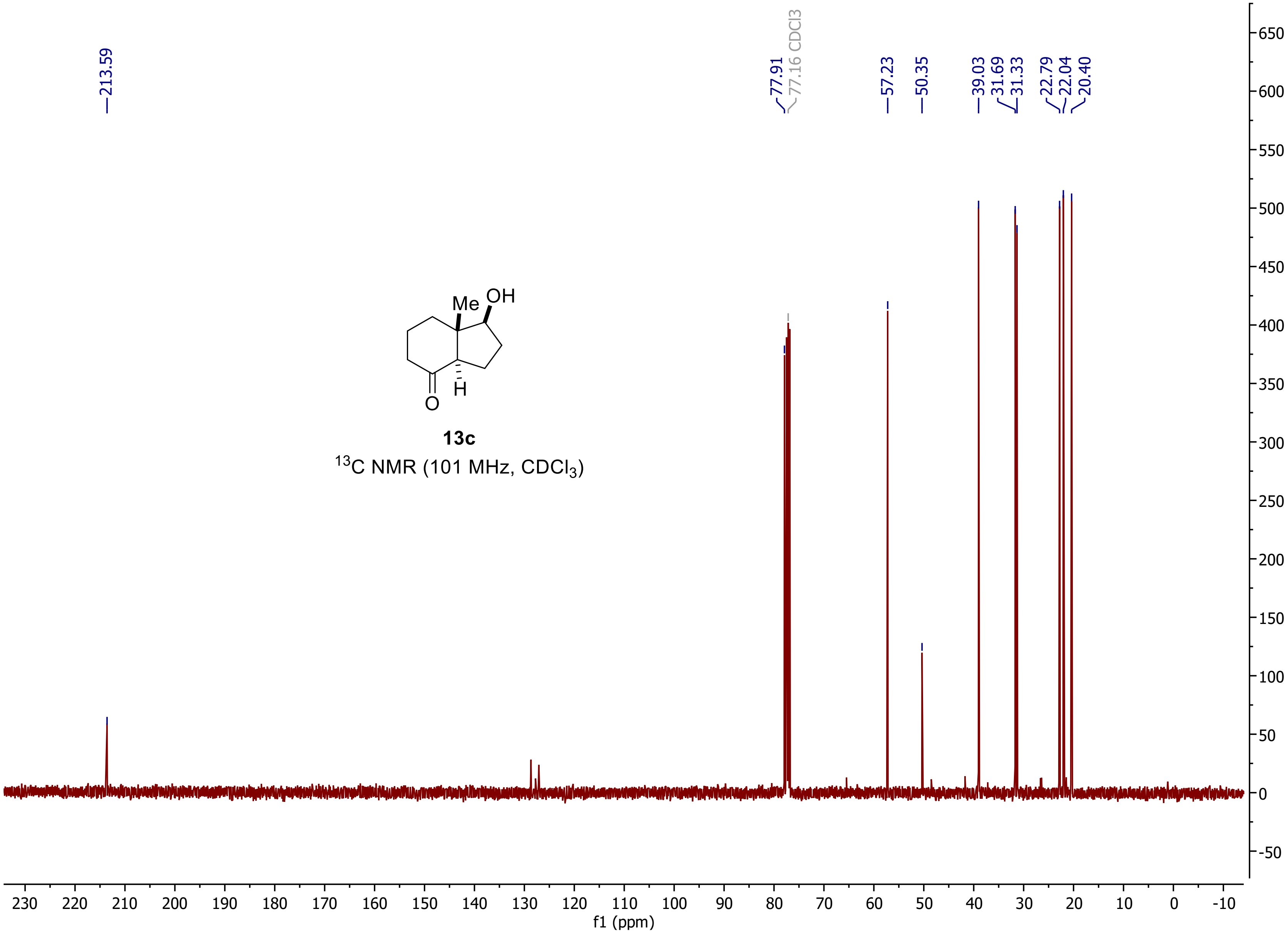

213.59
77.91
77.16 CDCl3
57.23
50.35
39.03
31.69
31.33
22.79
22.04
20.40
Me
OH
H
O
13c
13C NMR (101 MHz, CDCl3)
f1 (ppm)

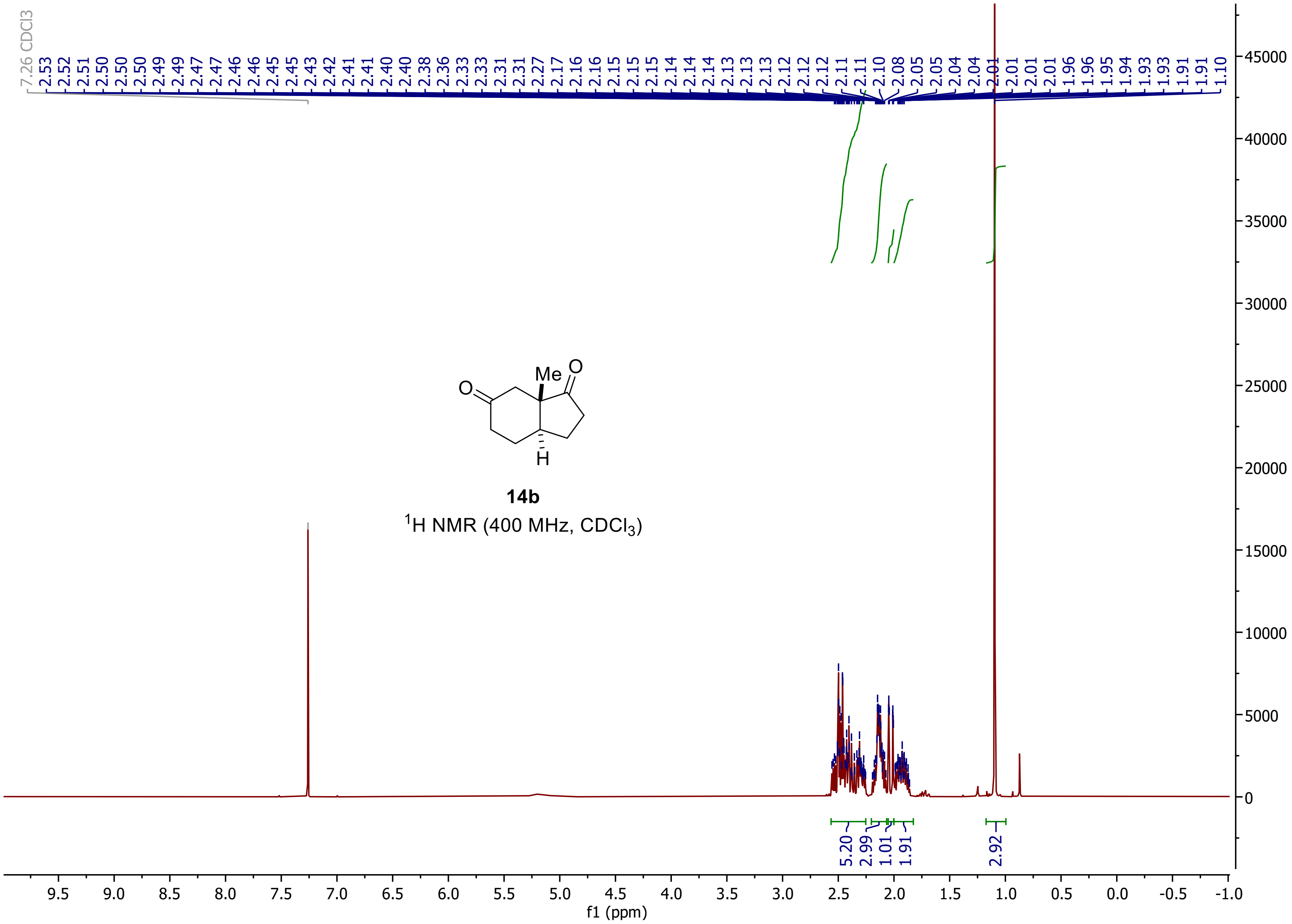

14b

^1H NMR (400 MHz, $CDCl_3$)

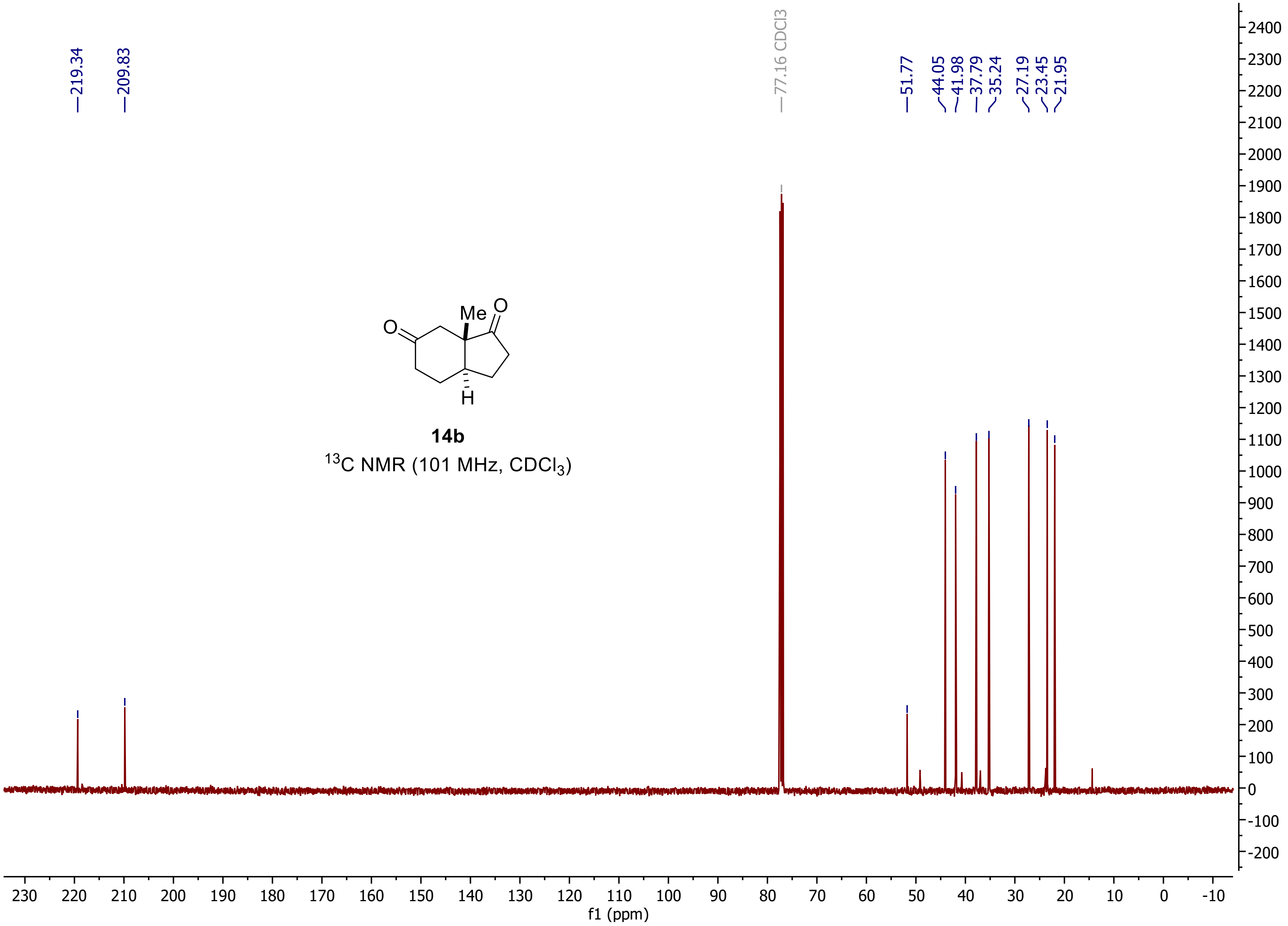

14b

^{13}C NMR (101 MHz, $CDCl_3$)

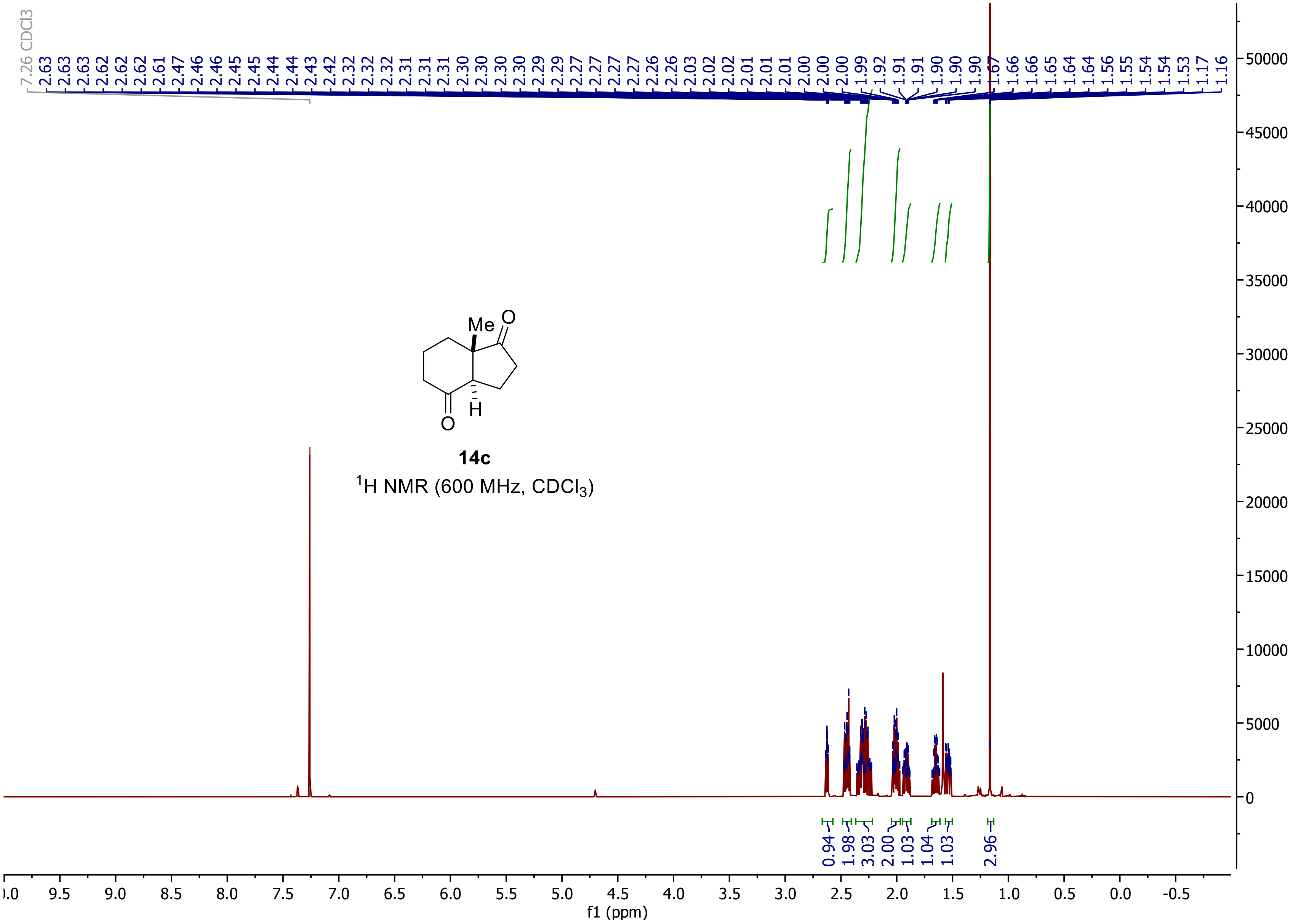

14c

^{1}H NMR (600 MHz, $CDCl_3$)

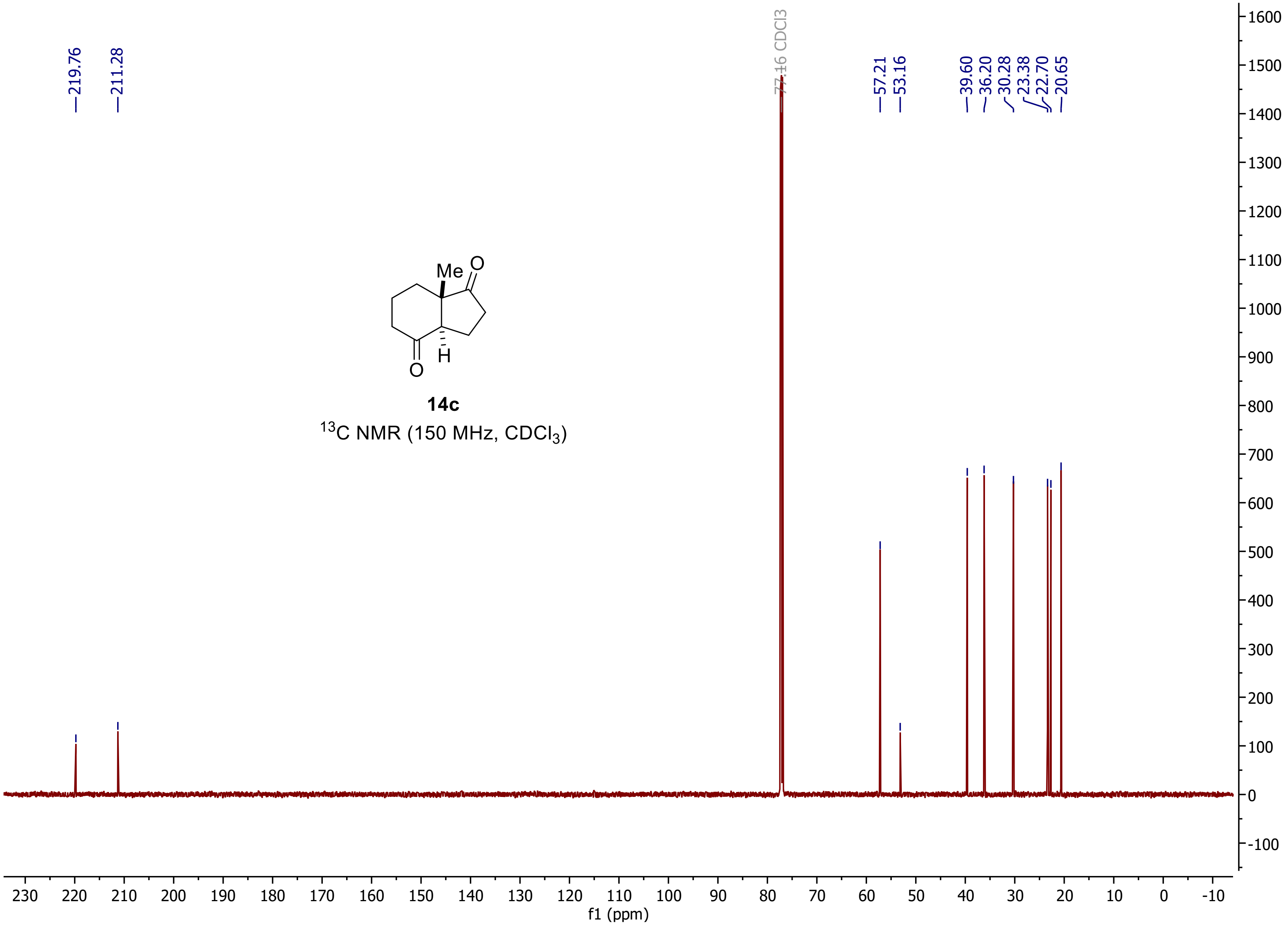

219.76
211.28
77.16 CDCl3
57.21
53.16
39.60
36.20
30.28
23.38
22.70
20.65
Me
O
O
H
14c
^{13}C NMR (150 MHz, $CDCl_3$)
f1 (ppm)

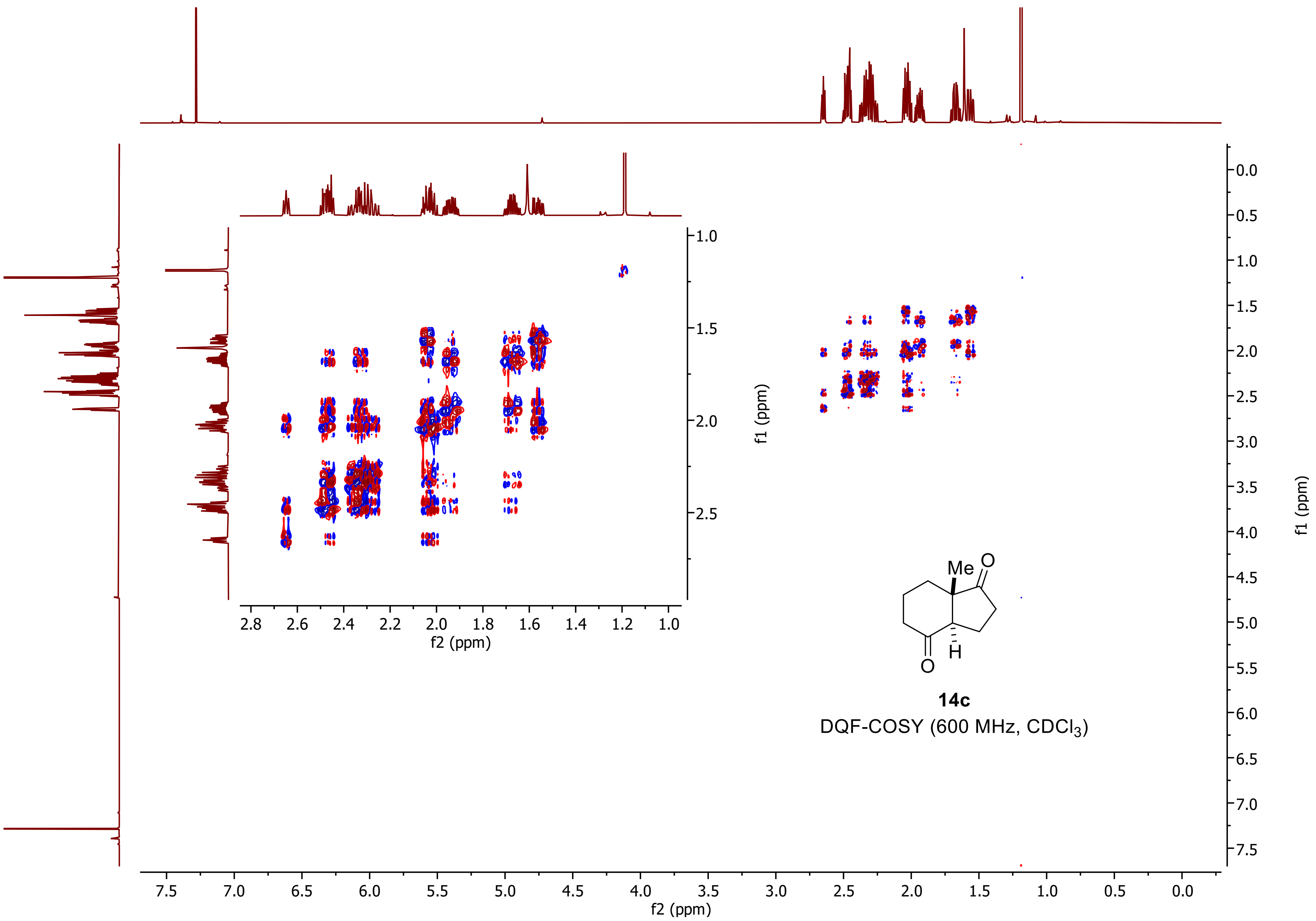

14c
DQF-COSY (600 MHz, $CDCl_3$)

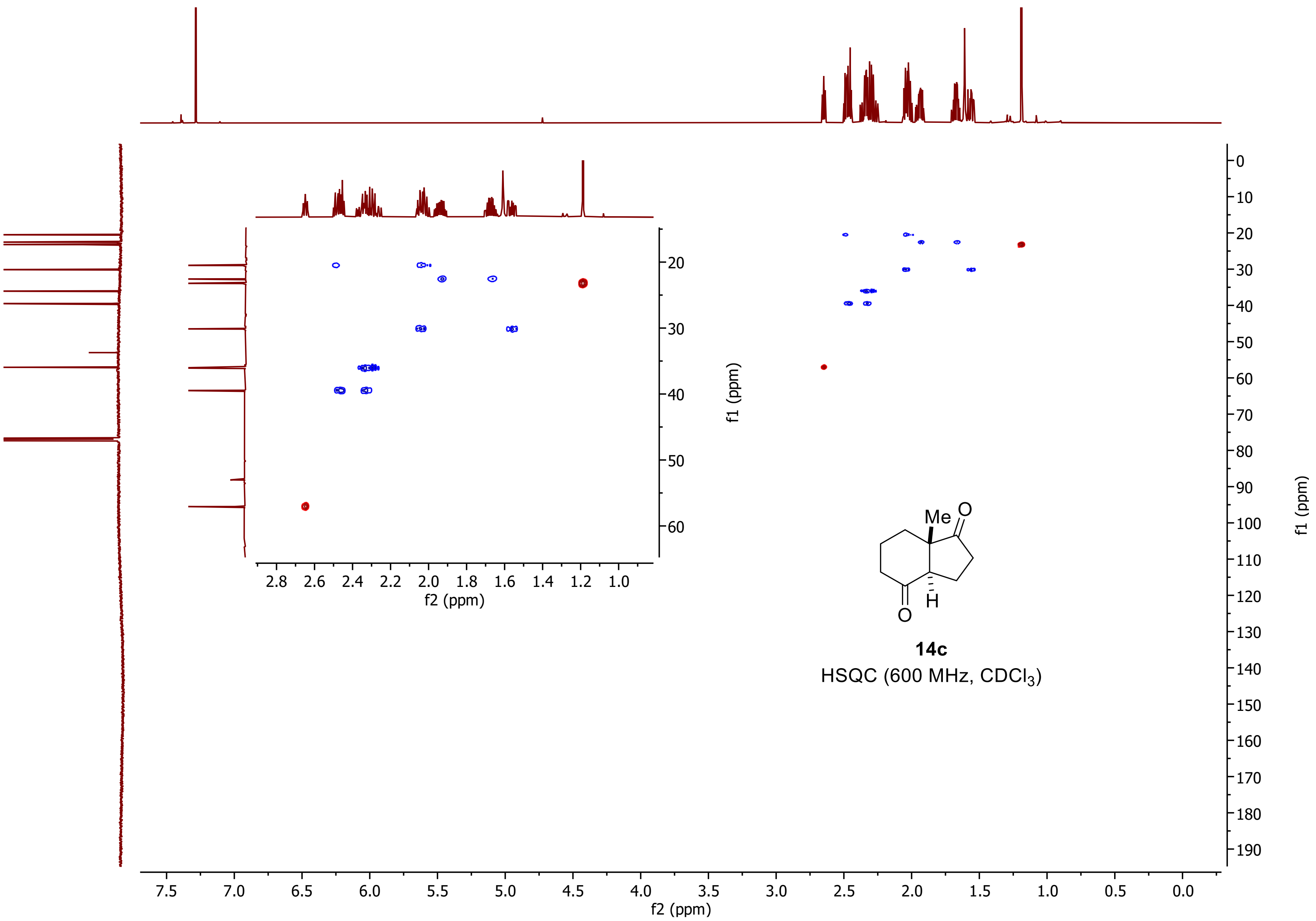

14c

HSQC (600 MHz, $CDCl_3$)

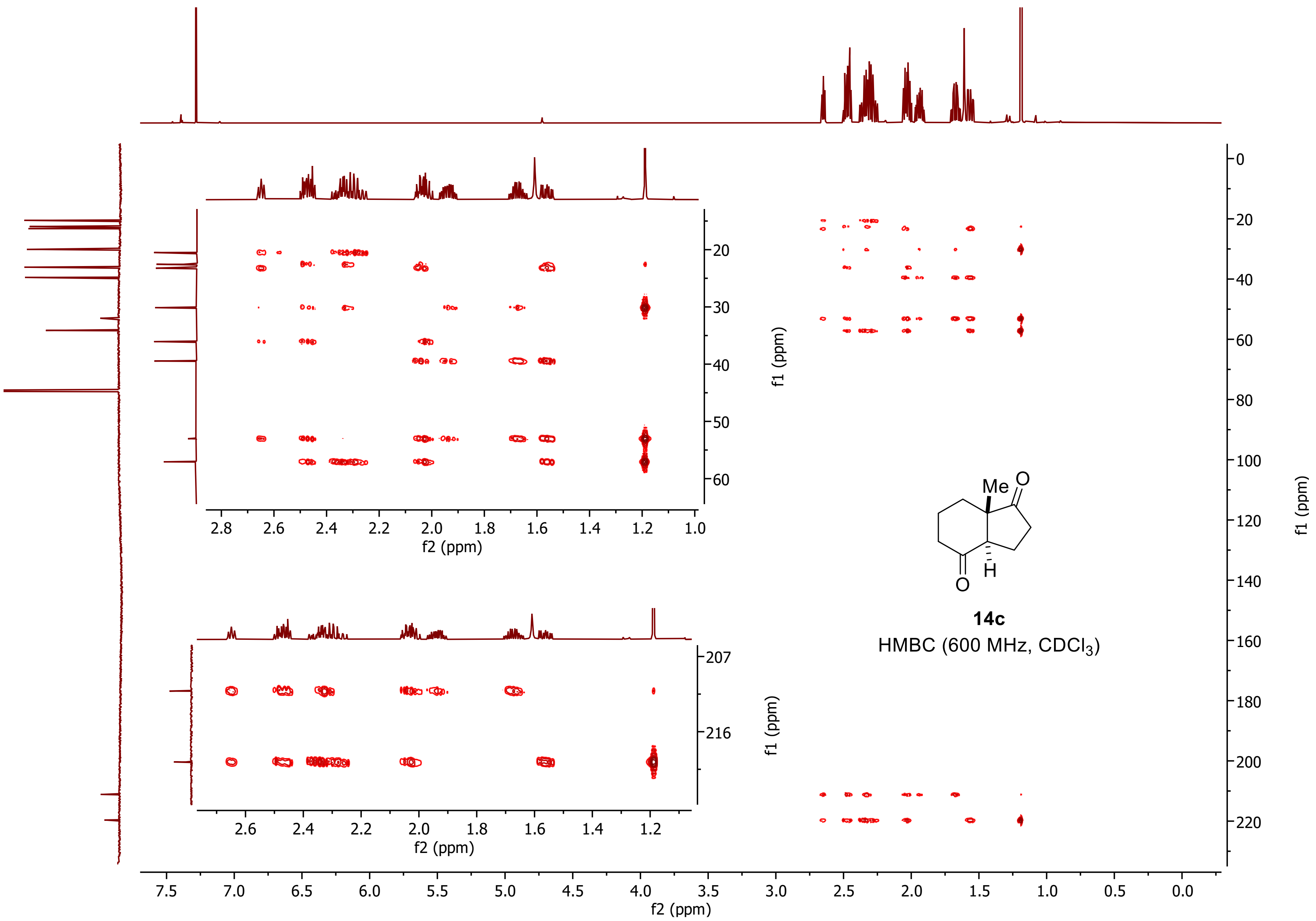
Me
O
H
O
14c
HMBC (600 MHz, $CDCl_3$)
f2 (ppm)
f1 (ppm)

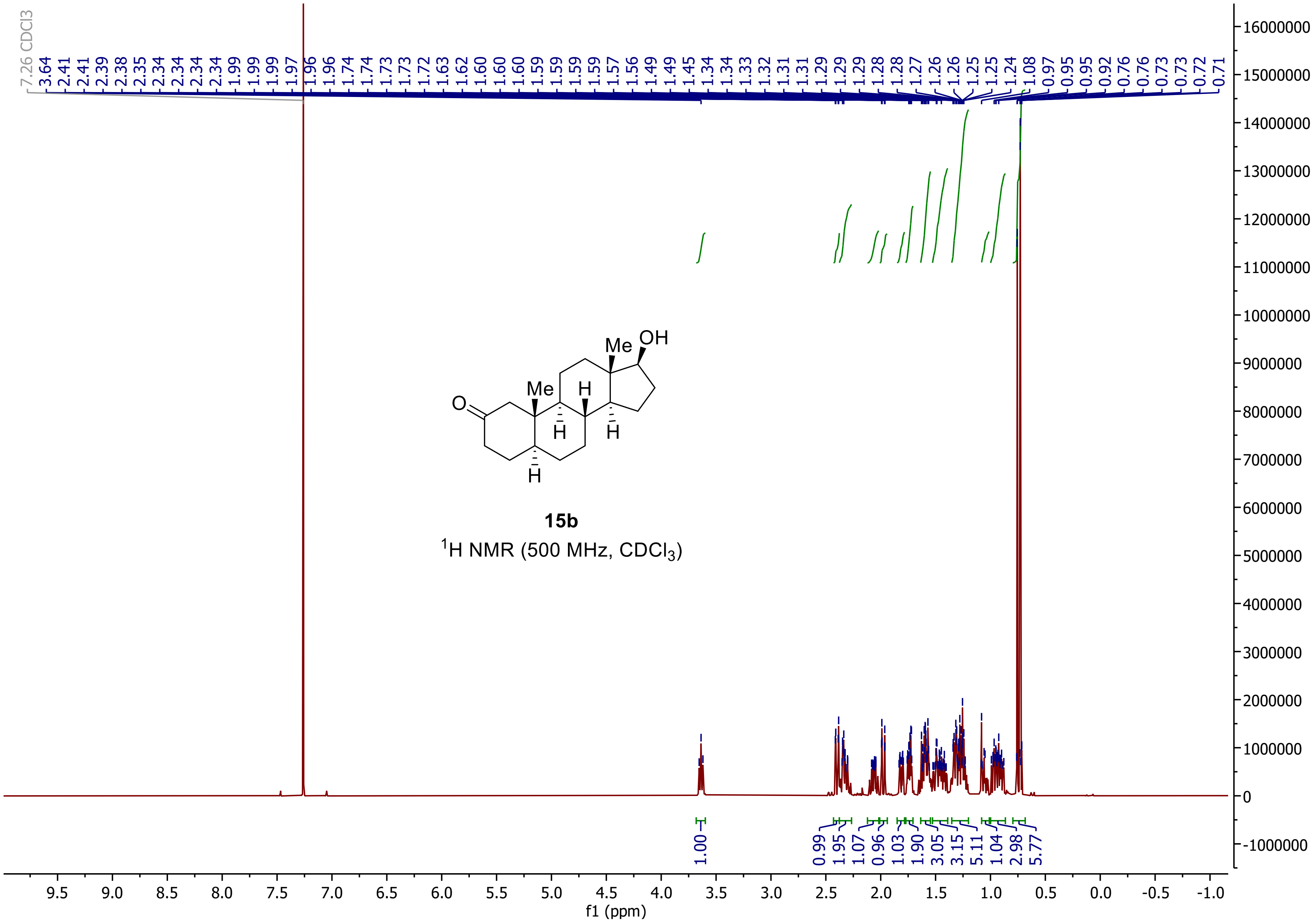

15b

^{1}H NMR (500 MHz, $CDCl_3$)

15b

^{13}C NMR (126 MHz, $CDCl_3$)

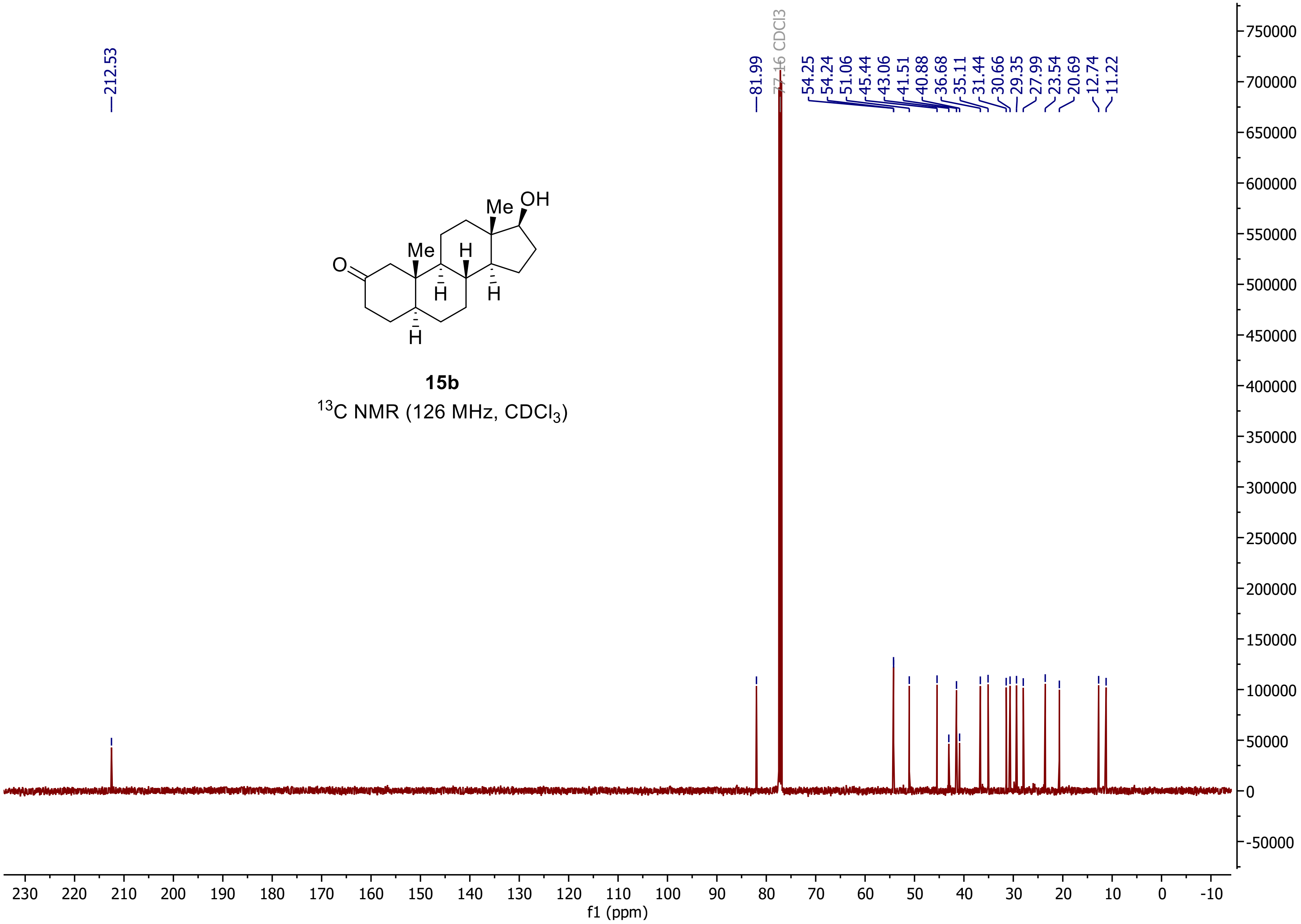

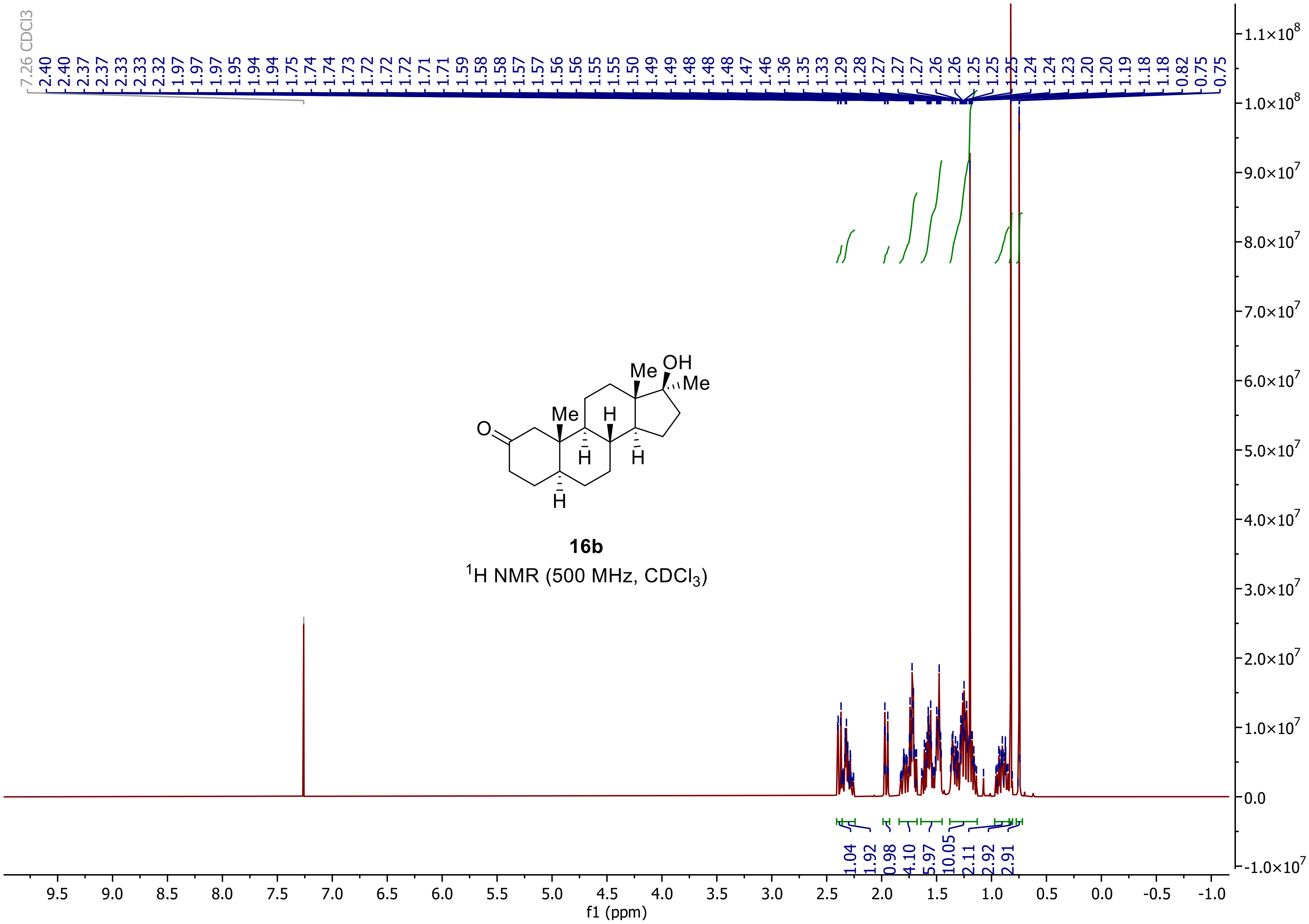

7.26 CDCl3
2.40 2.40 2.37 2.37 2.33 2.33 2.32 1.97 1.97 1.97 1.95 1.94 1.94 1.75 1.74 1.74 1.73 1.72 1.72 1.72 1.71 1.71 1.59 1.58 1.58 1.57 1.57 1.56 1.56 1.55 1.55 1.50 1.49 1.49 1.48 1.48 1.48 1.47 1.46 1.36 1.35 1.33 1.29 1.28 1.27 1.27 1.27 1.26 1.26 1.25 1.25 1.25 1.24 1.24 1.23 1.20 1.20 1.19 1.18 1.18 0.82 0.75 0.75
Me
OH
Me
Me
H
H
H
O
H
16b
1H NMR (500 MHz, CDCl3)
1.04 1.92 0.98 4.10 5.97 10.05 2.11 2.92 2.91
f1 (ppm)

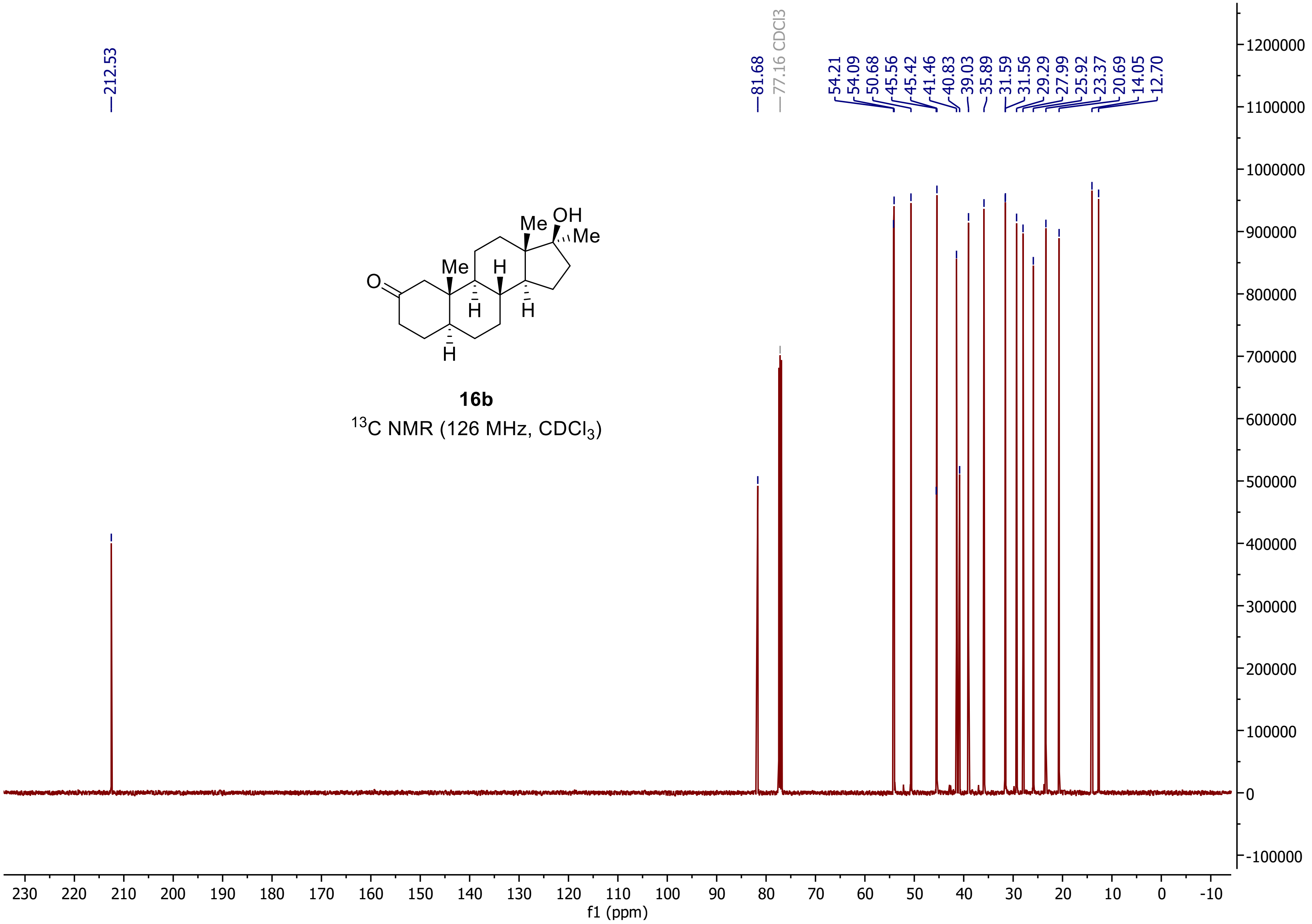
212.53
81.68
77.16 CDCl3
54.21
54.09
50.68
45.56
45.42
41.46
40.83
39.03
35.89
31.59
31.56
29.29
27.99
25.92
23.37
20.69
14.05
12.70
Me
OH
Me
Me
H
O
H
H
H
16b
13C NMR (126 MHz, CDCl3)
f1 (ppm)

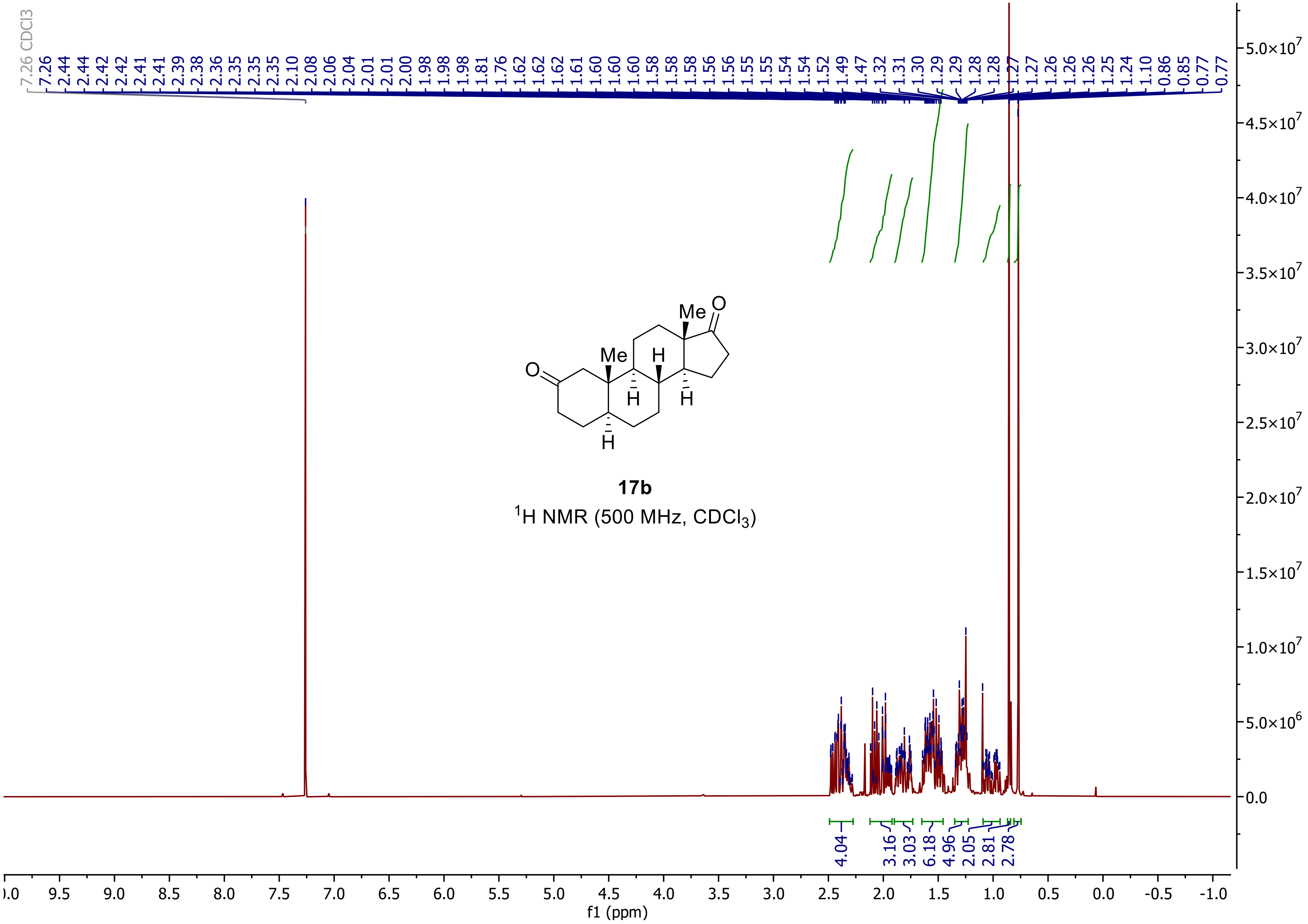

17b

^{1}H NMR (500 MHz, $CDCl_3$)

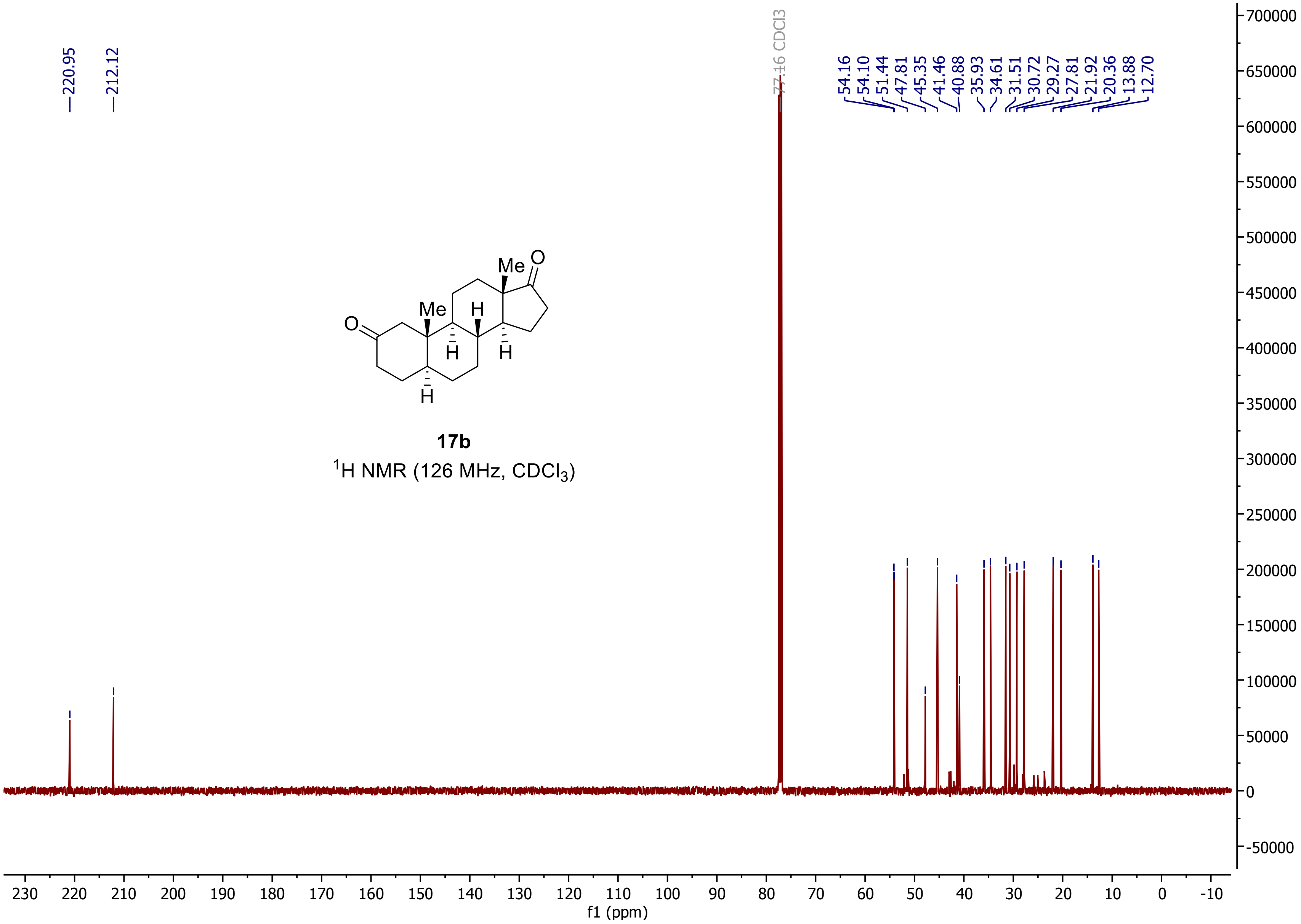
220.95
212.12
77.16 CDCl3
54.16
54.10
51.44
47.81
45.35
41.46
40.88
35.93
34.61
31.51
30.72
29.27
27.81
21.92
20.36
13.88
12.70
Me
O
Me
H
O
H
H
H
17b
^{1}H NMR (126 MHz, $CDCl_3$)
700000
650000
600000
550000
500000
450000
400000
350000
300000
250000
200000
150000
100000
50000
0
-50000
230 220 210 200 190 180 170 160 150 140 130 120 110 100 90 80 70 60 50 40 30 20 10 0 -10
f1 (ppm)

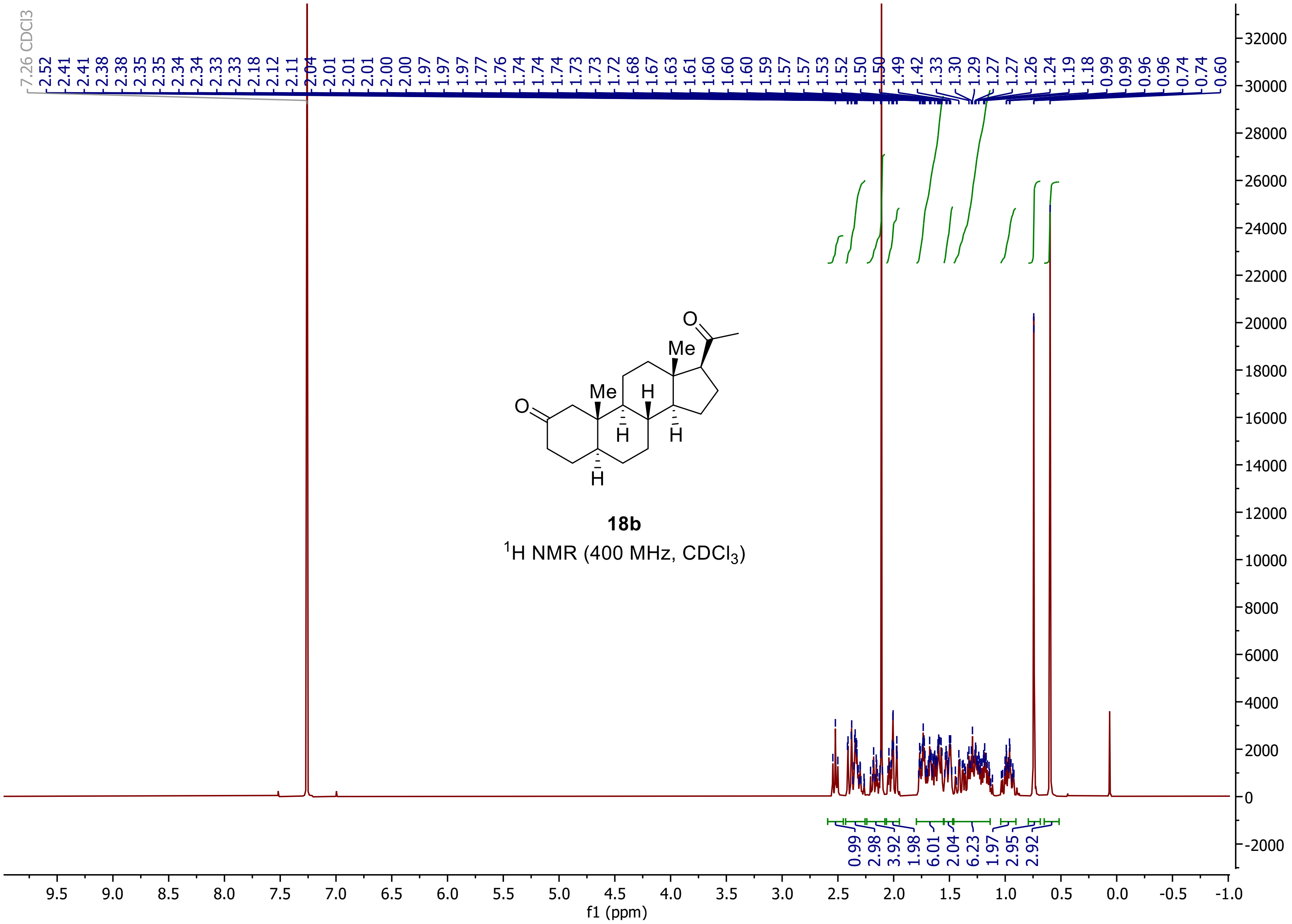
O
Me
Me
H
O
H
H
H
18b
1H NMR (400 MHz, CDCl3)
7.26 CDCl3
f1 (ppm)

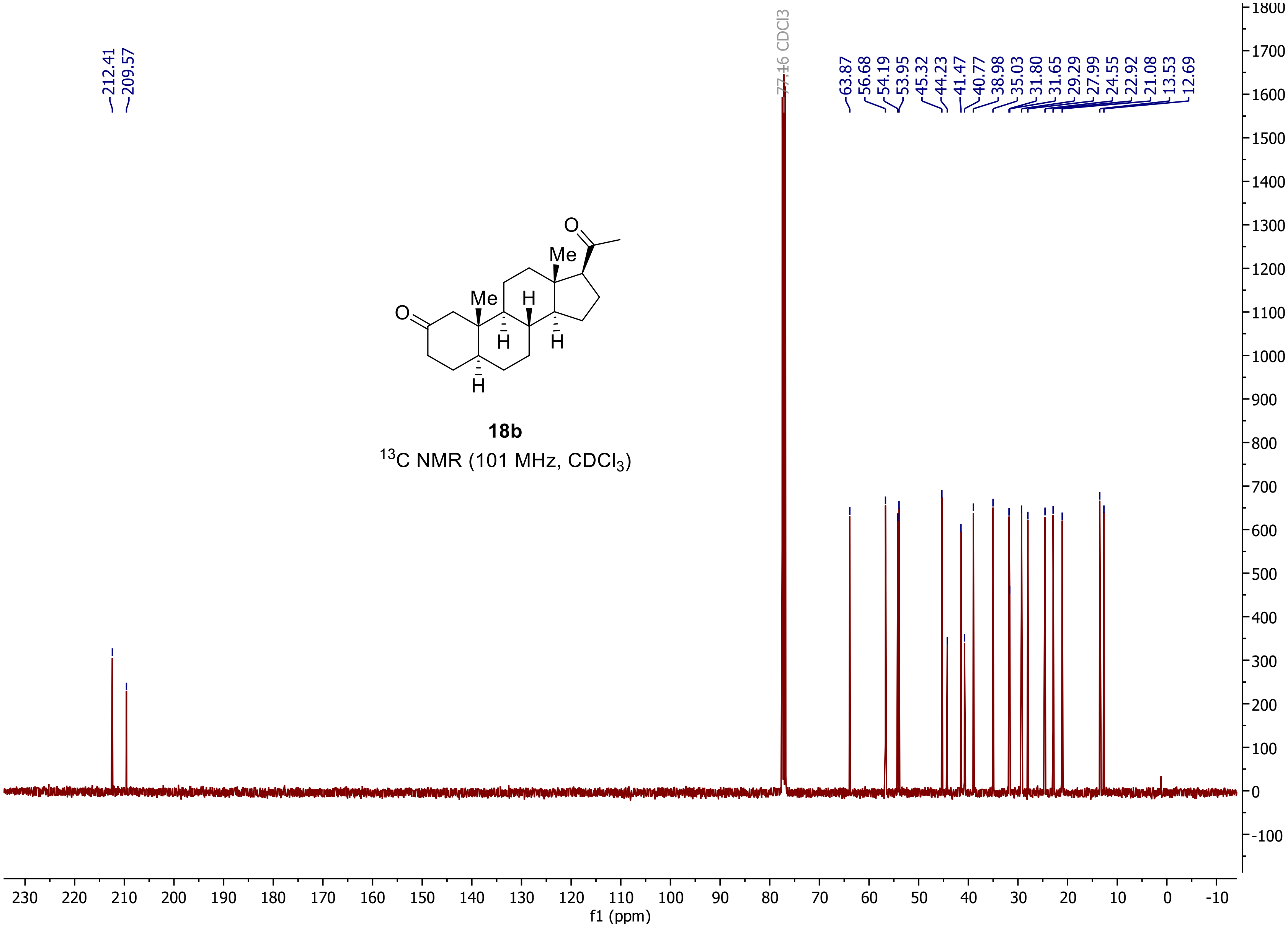

18b

^{13}C NMR (101 MHz, $CDCl_3$)
